# Supplementary material for: Discovering multiscale deep formulas in complex systems via neural-guided lambda calculus
Source: Nat Commun. 2026 Jun 16;17:7639. doi: 10.1038/s41467-026-74299-z (PMC13434754; doi:10.1038/s41467-026-74299-z)
Supplement: Supplementary file 1 [file 41467_2026_74299_MOESM1_ESM.pdf]

# Supplementary Information for: Discovering Multiscale Deep Formulas in Complex Systems via Neural-Guided Lambda Calculus

Hanqiao Yu[1,2,†], Shusen Yang[1,2,*,†], Xuebin Ren[1,3,*], Cong Zhao[1,2]

[1]National Engineering Laboratory for Big Data Analytics, Xi'an Jiaotong University

[2]School of Mathematics and Statistics, Xi'an Jiaotong University

[3]School of Computer Science and Technology, Faculty of Electronic and Information Engineering, Xi'an Jiaotong University

Xi'an, 710049, Shaanxi, China

*Corresponding authors: shusenyang@mail.xjtu.edu.cn, xuebinren@mail.xjtu.edu.cn

†These authors contributed equally to this work.

## Contents

**1 Design rationale** **4**

**2 Energy-based loss model training** **4**

2.1 EBMs learning framework . . . . . . . . . . . . . . . . . . . . . . . . . . . 4

2.2 MCMC-based $\nabla_{\boldsymbol{\theta}} \log Z_{\boldsymbol{\theta}}$ estimation . . . . . . . . . . . . . . . . . . . 6

2.3 Parameter setup for MCMC sampling . . . . . . . . . . . . . . . . . . . . . 7

**3 Lambda calculus-based symbolic regression** **7**

3.1 Basic introduction to lambda calculus . . . . . . . . . . . . . . . . . . . . . 8

3.2 Detailed walk-through of the fixed-point aggregation example . . . . . . . . . 9

3.3 Extended lambda expressions . . . . . . . . . . . . . . . . . . . . . . . . . 11

3.4 Generation and mutation for lambda calculus expressions . . . . . . . . . . . . 15
3.5 Initialization with global aggregation priors . . . . . . . . . . . . . . . . . . . . . 18
3.6 Expression complexity . . . . . . . . . . . . . . . . . . . . . . . . . . . . . . 19

**4 Experimental setup 20**
4.1 Comparison setup . . . . . . . . . . . . . . . . . . . . . . . . . . . . . . . . 20
4.2 Configurations of compared methods . . . . . . . . . . . . . . . . . . . . . . . 22
4.3 Coarse-graining and renormalization protocols . . . . . . . . . . . . . . . . . . . 24
4.4 Observation scale and sampling strategy . . . . . . . . . . . . . . . . . . . . . . 25
4.5 Argon particle motion . . . . . . . . . . . . . . . . . . . . . . . . . . . . . . . 26
4.6 Pollen particle motion . . . . . . . . . . . . . . . . . . . . . . . . . . . . . . . 29
4.7 Human mobility . . . . . . . . . . . . . . . . . . . . . . . . . . . . . . . . . . 30
4.8 Bird migration . . . . . . . . . . . . . . . . . . . . . . . . . . . . . . . . . . . 30
4.9 Fluid motion . . . . . . . . . . . . . . . . . . . . . . . . . . . . . . . . . . . . 31
4.10 Water particles . . . . . . . . . . . . . . . . . . . . . . . . . . . . . . . . . . 38

**5 Successfully discovered formulas of all methods 39**

**6 Quantitative performance results 47**

**7 Supplementary experiments 47**
7.1 Impact of lambda-aware mutation . . . . . . . . . . . . . . . . . . . . . . . . . 47
7.2 Impact of pre-training . . . . . . . . . . . . . . . . . . . . . . . . . . . . . . . 50
7.3 Impact of network hyperparameters . . . . . . . . . . . . . . . . . . . . . . . . 51
7.4 Impact of data augmentation sampling . . . . . . . . . . . . . . . . . . . . . . . 53
7.5 Ablation study on derivative (gradient) features . . . . . . . . . . . . . . . . . . 55
7.6 Fitting analysis for Deflexformer on conservation laws . . . . . . . . . . . . . . 57

7.7 Comparison of discovery rate on quantum transition probability laws . . . . . . 59

7.8 Impact of scientist-in-the-loop approach on baseline methods . . . . . . . . . . 61

**8 Detailed algorithms and pseudocodes** **63**

8.1 Data representation and Deflexformer architecture . . . . . . . . . . . . . . . . . 63

8.2 Neural-guided expression generator architecture . . . . . . . . . . . . . . . . . . . 70

8.3 Hierarchical symbolic regression . . . . . . . . . . . . . . . . . . . . . . . . . . 70

8.4 Energy-based learning . . . . . . . . . . . . . . . . . . . . . . . . . . . . . . . . 72

8.5 Overall workflow . . . . . . . . . . . . . . . . . . . . . . . . . . . . . . . . . . . 73

# 1 Design rationale

As illustrated in Supplementary Fig. 1, traditional formula discovery relies on manual scale-specific exploration and domain expertise (panels a-c), while Deflex automates this process through integrated deep learning and symbolic regression (panel d). The core innovation is a unified energy-based formulation that treats both invariants and distributions as probability patterns, enabling consistent handling of diverse mathematical forms across scales. The architecture comprises Deflexformer for learning scale-aware representations via energy-based models, and Deflexpressor for extracting symbolic expressions via lambda-calculus-based regression.

# 2 Energy-based loss model training

In this article, we propose an energy-based model framework for modeling the likelihood of mathematical patterns to the observed data from complex systems.

## 2.1 EBMs learning framework

We consider all patterns as distributions. Specifically, besides the original distribution patterns, those invariant patterns can be converted into their error distributions. Based on this, we aim to identify the distributions, e.g., via specifying its probabilistic distribution function (PDF) $p(\mathbf{x})$ related to the state variable $\mathbf{x}$ of the complex system, from some observation data $\mathbf{X}$. For simplicity, we train an energy-based model (EBM) parameterized by model parameter $\boldsymbol{\theta}$ (representing neural network weights) over the dataset $\mathbf{X}$, where each data item follows the true distribution $\mathbf{x} \sim p(\mathbf{x}), \mathbf{x} \in \mathbf{X}$. The goal is to make $Q_{\boldsymbol{\theta}}(\mathbf{x})$, the PDF suggested by the EBM, approximate $p(\mathbf{x})$. According to the EBM theory, each data point $\mathbf{x} \in \mathbf{X}$ is mapped to an energy value $\mathcal{E}_{\boldsymbol{\theta}}(\mathbf{x})$ parameterized by model parameter $\boldsymbol{\theta}$, and the probability $Q_{\boldsymbol{\theta}}(\mathbf{x})$ of $\mathbf{x}$ is inversely proportional to its energy value $\mathcal{E}_{\boldsymbol{\theta}}(\mathbf{x})$ (i.e., lower energy corresponds to higher probability).

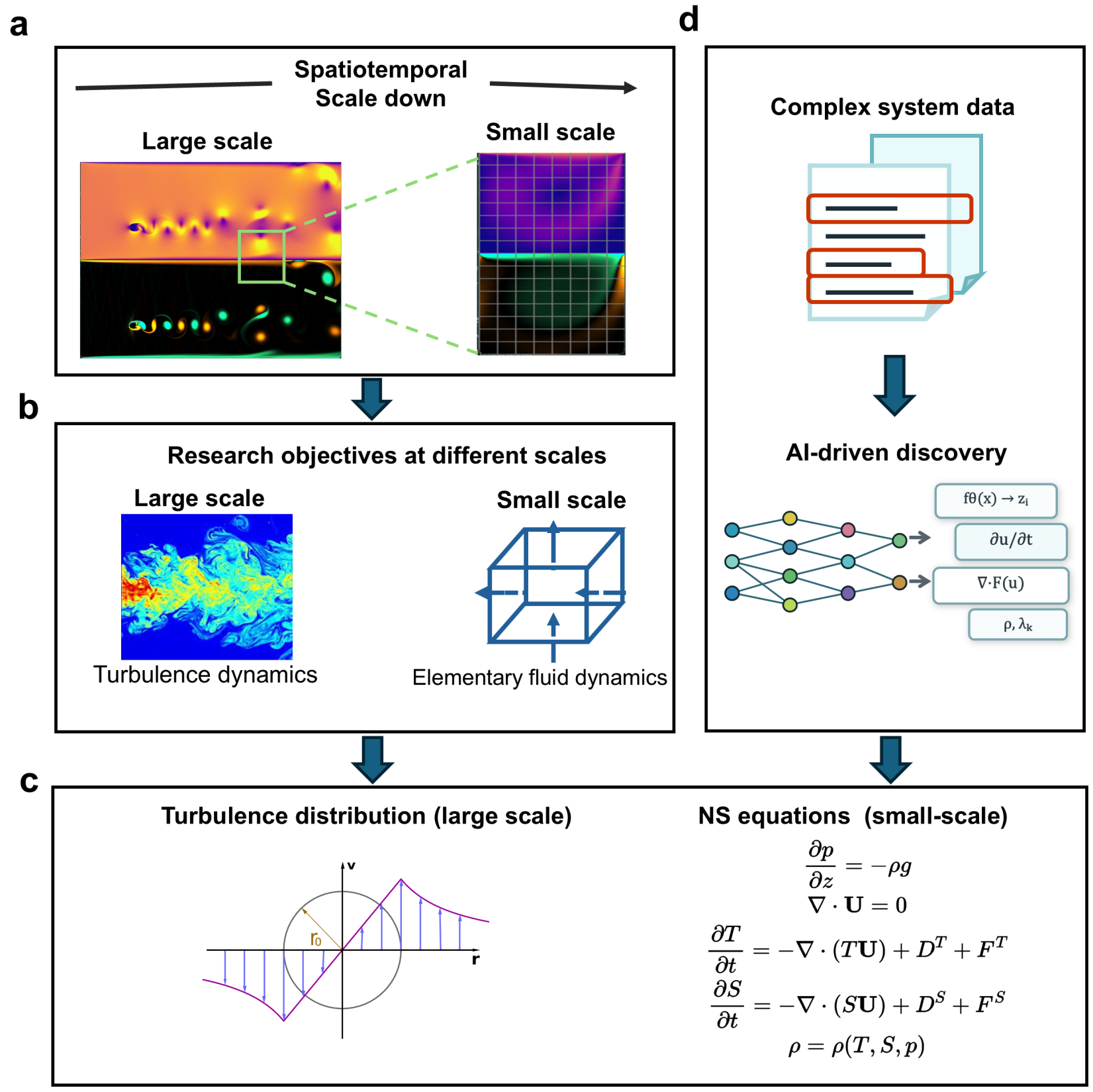


Supplementary Figure 1: Comparison of human and AI approaches for mathematical law discovery in complex systems. **a-c** Human approach: scale-specific exploration (**a**), domain expert analysis (**b**), multiscale law synthesis (**c**). **d** Deflex approach: automated pattern identification and symbolic extraction from observational data.

That is,

$$Q_{\boldsymbol{\theta}}(\mathbf{x}) = \frac{1}{Z_{\boldsymbol{\theta}}} \exp\left(-\mathcal{E}_{\boldsymbol{\theta}}(\mathbf{x})\right), \tag{1}$$

where $Z_{\boldsymbol{\theta}}$ is the normalization factor (also known as the partition function) associated with $\boldsymbol{\theta}$. It is defined as

$$Z_{\boldsymbol{\theta}} = \int \exp\left(-\mathcal{E}_{\boldsymbol{\theta}}(\mathbf{x})\right) d\mathbf{x}. \tag{2}$$

To ensure that $Q_{\boldsymbol{\theta}}(\mathbf{x})$ is close to $p(\mathbf{x})$, an EBM should have an optimal likelihood over the dataset $\mathbf{X}$. The likelihood function can be defined as $\mathbb{E}_{\mathbf{x}\sim p(\mathbf{x})}\left[\log Q_{\boldsymbol{\theta}}(\mathbf{x})\right]$, and then be estimated

as

$$\mathbb{E}_{\mathbf{x}\sim p(\mathbf{x})}\left[\log Q_{\boldsymbol{\theta}}(\mathbf{x})\right] \simeq \frac{1}{\|\mathbf{X}\|}\sum_{\mathbf{x}_i\in\mathbf{X}}\log Q_{\boldsymbol{\theta}}(\mathbf{x}_i) \tag{3}$$

Thus, the training of the EBM can be performed by gradient descent of $\log Q_{\boldsymbol{\theta}}(\mathbf{x})$ over dataset $\mathbf{X}$. Specifically, the gradient of the log-likelihood of $Q_{\boldsymbol{\theta}}$ with respect to $\boldsymbol{\theta}$, can be estimated by the average gradient on dataset $\mathbf{X}$.

$$\nabla_{\boldsymbol{\theta}}\mathbb{E}_{\mathbf{x}\sim p(\mathbf{x})}\left[\log Q_{\boldsymbol{\theta}}(\mathbf{x})\right] \simeq \frac{1}{\|\mathbf{X}\|}\sum_{\mathbf{x}_i\in\mathbf{X}}\nabla_{\boldsymbol{\theta}}\log Q_{\boldsymbol{\theta}}(\mathbf{x}_i). \tag{4}$$

According to [1], the gradient of the log-likelihood on a single data point $\mathbf{x}_i \in \mathbf{X}$ follows

$$\nabla_{\boldsymbol{\theta}}\log Q_{\boldsymbol{\theta}}(\mathbf{x}_i) = -\nabla_{\boldsymbol{\theta}}\mathcal{E}_{\boldsymbol{\theta}}(\mathbf{x}_i) - \nabla_{\boldsymbol{\theta}}\log Z_{\boldsymbol{\theta}}, \tag{5}$$

where $\nabla_{\boldsymbol{\theta}}\mathcal{E}_{\boldsymbol{\theta}}(\mathbf{x}_i)$ can be directly calculated since $\mathcal{E}_{\boldsymbol{\theta}}(\mathbf{x})$ is explicitly related to $\boldsymbol{\theta}$ in our method. However, calculating $\nabla_{\boldsymbol{\theta}}\log Z_{\boldsymbol{\theta}}$ is mathematically intractable in general [1], due to the lack of an analytical solution for the integral in Eq. 2. Therefore, we adopt the Markov Chain Monte Carlo (MCMC) method [2, 3] to approximately compute $\nabla_{\boldsymbol{\theta}}\log Z_{\boldsymbol{\theta}}$ in the following.

## 2.2 MCMC-based $\nabla_{\boldsymbol{\theta}}\log Z_{\boldsymbol{\theta}}$ estimation

According to Eq. 2, we have

$$\nabla_{\boldsymbol{\theta}}\log Z_{\boldsymbol{\theta}} = \nabla_{\boldsymbol{\theta}}\log\int\exp\left(-\mathcal{E}_{\boldsymbol{\theta}}(\mathbf{x})\right)d\mathbf{x} = \mathbb{E}_{\mathbf{x}\sim Q_{\boldsymbol{\theta}}(\mathbf{x})}\left[-\nabla_{\boldsymbol{\theta}}\mathcal{E}_{\boldsymbol{\theta}}(\mathbf{x})\right]. \tag{6}$$

Suppose that there are $M$ data points $\mathbf{Y} = \{\mathbf{y}_i, i \in 1, 2, \cdots, M\}$ following the model distribution $Q_{\boldsymbol{\theta}}$, then the expectation $\mathbb{E}_{\mathbf{x}\sim Q_{\boldsymbol{\theta}}(\mathbf{x})}\left[-\nabla_{\boldsymbol{\theta}}\mathcal{E}_{\boldsymbol{\theta}}(\mathbf{x})\right]$, or $\nabla_{\boldsymbol{\theta}}\log Z_{\boldsymbol{\theta}}$, can be estimated by the mean gradient $\frac{1}{M}\sum_{i=1}^{M}\left(-\nabla_{\boldsymbol{\theta}}\mathcal{E}_{\boldsymbol{\theta}}\left(\mathbf{y}_i\right)\right)$ calculated over $\mathbf{Y}$.

Given $\mathcal{E}_{\boldsymbol{\theta}}(\mathbf{x})$, we use the Langevin sampling method to sample a dataset $\mathbf{Y}$ with $M$ data points from $Q_{\boldsymbol{\theta}}$. Suppose $\mathbf{Y}^0 = \{\mathbf{y}_i^0, i \in 1, 2, \cdots, M\}$ is a randomly initialized dataset, and $\mathbf{Y}^k = \left\{\mathbf{y}_i^k, i \in 1, 2, \cdots, M\right\}$ is the result of $k$-th ($k > 0$) Langevin sampling. Then, the

Langevin sampling that mimics the Langevin diffusion process [2] can be described as

$$\mathbf{y}_i^{k+1} \leftarrow \mathbf{y}_i^k - \frac{\epsilon^2}{2}\nabla_{\mathbf{y}_i^k}\mathcal{E}_{\boldsymbol{\theta}}\left(\mathbf{y}_i^k\right) + \epsilon\mathbf{y}_i^k, \quad k = 0, 1, \cdots, K-1, \quad i = 1, \cdots M. \tag{7}$$

where $\epsilon$ is a step-size-related parameter, which is set as $\frac{\max_{\mathbf{x}\in\mathbf{X}}\|\mathbf{x}\|}{2K}$ in this work. Denote $\mathbf{Y} = \mathbf{Y}^K$ as the final sampled dataset from $Q_{\boldsymbol{\theta}}$. When $K$ is sufficiently large and $\epsilon$ is sufficiently small, the distribution of the sampled data points would be very close to the model distribution $Q_{\boldsymbol{\theta}}$.

### 2.3 Parameter setup for MCMC sampling

In our experiments, we conducted multiple iterations of Langevin sampling. In the first iteration, we uniformly sampled the $M$ points, where $M$ ranges from 6 to 128 depending on the volume of different complex systems. Then each point was updated by the Langevin sampling method for $K = 128$ iterations to fit the model distribution. The gradient of the partition function was estimated by these points according to Eq. 6, and the energy-based model was further updated by the gradients according to Eq. 4. Due to the high computing cost of the Langevin sampling method, in later iterations, 95% of the negative samples were reused from a persistent buffer, and the remaining 5% were re-initialized from noise to maintain diversity.

## 3 Lambda calculus-based symbolic regression

In our work, we use a symbolic regression framework, Deflexpressor, to identify concise lambda calculus-based mathematical patterns. Lambda calculus, introduced by Alonzo Church in the 1930s [4], is a formal system in mathematical logic and computer science for expressing computation based on function abstraction and application.

## 3.1 Basic introduction to lambda calculus

Lambda calculus represents computation using only functions. Its basic building blocks are (i) defining a function, and (ii) applying a function to an input. Although there are no built-in representations for numbers, booleans, loops, or data structures, they all can be encoded using functions (often called Church encodings [5]).

A lambda term is built from three syntactic forms:

- $x$ (variable): a placeholder denoting a parameter.
- $\lambda x.\, M$ (abstraction): a function with parameter $x$ and body $M$.
- $M\ N$ (application): applying function $M$ to argument $N$.

For example, $\lambda x.\, x$ represents the identity function $f(x) = x$, and $(\lambda x.\, x)\ y$ denotes applying function $f(x) = x$ to argument $y$, which reduces to $y$.

Two standard conventions remove the ambiguity of computing rules:

- Application is left-associative: $M\ N\ P$ means $(M\ N)\ P$.
- Abstraction extends to the rightmost: $\lambda x.\, M\ N$ means $\lambda x.\, (M\ N)$.

Computation is performed by rewriting terms according to a small set of rules. The key rule is $\beta$-reduction (function application):

$$(\lambda x.\, M)\ N \ \to_\beta\ M[x := N]. \tag{8}$$

meaning that $N$ is substituted for each free occurrence of $x$ in $M$ (renaming bound variables when needed to avoid capture). Two additional, commonly used equivalences are: $\alpha$-conversion (renaming bound variables, e.g., $\lambda x.\, x \equiv \lambda y.\, y$) and $\eta$-conversion (extensionality, e.g., $\lambda x.\, M\, x \equiv M$ when $x$ is not free in $M$).

Since there is no built-in support, recursions can be represented using a fixed-point combinator such as the $Y$ combinator [5]:

$$Y \;=\; \lambda f.\,(\lambda x.\,f(x\,x))(\lambda x.\,f(x\,x)). \tag{9}$$

which satisfies $Y\,F \;=\; F\,(Y\,F)$ for any $F$. Operationally, it turns a non-recursive "one-step update rule" $F$ into a self-referential function.

Lambda calculus is widely used as a foundation for functional programming language semantics and for studying computability and type systems. Programs can be expressed as functions and evaluated by repeated substitution, and higher-level constructs (data, branching, recursion) can be encoded within this minimal core. In this work, we regard each lambda expression as an explicit, compositional function built from abstraction and application that maps structured observations (and optional global context) to a scalar, enabling aggregation over variable-sized entity collections within the expression itself.

## 3.2 Detailed walk-through of the fixed-point aggregation example

We revisit the aggregation example in the main text, $E = \sum_{i\neq j} V(r_{ij})$, where $V(\cdot)$ denotes a pairwise interaction potential and $r_{ij}$ denotes the distance between particles $i$ and $j$. To connect this summation to a variable-length input, we collect all pair distances into a list $\mathbf{x} = [r_{ij}]_{i\neq j}$ under any fixed ordering. The following lambda expression implements the aggregation as an executable recursion over $\mathbf{x}$ with an accumulator initialized as 0:

$$E = \underbrace{\Big(\underbrace{\text{Fix}\Big(\underbrace{\underbrace{\lambda f\;\lambda \text{xs}\;\lambda a.}_{\text{Variables}}\;\underbrace{\underbrace{\text{if Null(xs) then } a}_{\text{Application w.r.t. relationship (i)}}\;\text{else}\;\underbrace{f\;(\text{Tail(xs)})\;\big(a + V(\text{Head(xs)})\big)}_{\text{Application w.r.t. relationship (ii)}}}_{\text{(D) Function body describing the whole relationship (i) and (ii) among } f,\,\text{xs},\,a}}_{\text{(C) Abstraction defining a function G(f, xs, a) over variables } f,\,\text{xs},\,a}\Big)}_{\text{(B) Fixed-point simulating a recursive function F(xs, a) based on G(f, xs, a)}}\Big)\;\mathbf{x}\;0}_{\text{(A) Outermost application applying recursive function F(xs, a) over parameters } \mathbf{x} \text{ and } 0}\;. \tag{10}$$

We explain the notations, terms, and computational logic in the above lambda-calculus expression from the inside underbraces to the outside ones as follows.

- Function body (D): the expression (D) denotes a typical conditional sentence of the "if–then–else" style in the lambda calculus, which defines the body of a function. Within it, the relationship (i) returns $a$ if $\mathrm{Null}(\mathrm{xs})$ is true, and relationship (ii) continues by calling $f$ on $\mathrm{Tail}(\mathrm{xs})$ with updated accumulator $a+V(\mathrm{Head}(\mathrm{xs}))$. Specifically, $\mathrm{Head}(\mathrm{xs})$ returns the first element of $\mathrm{xs}$, $\mathrm{Tail}(\mathrm{xs})$ returns the remaining list of $\mathrm{xs}$ after removing the first element, and $\mathrm{Null}(\mathrm{xs})$ tests whether the list $\mathrm{xs}$ is empty. They are predefined functions that are commonly used in lambda calculus.

- Abstraction (C): the abstraction underbrace has the form $\lambda f.\lambda \mathrm{xs}.\lambda a.\, G$ and defines a function $G(f, \mathrm{xs}, a)$ with three parameters, where $f$ is the continuation, $\mathrm{xs}$ is the remaining list, and $a$ is the current accumulator. As we can see, the inner term (D) indeed describes the logic of the function $G(f, \mathrm{xs}, a)$.

- Fixed-point operation (B): $\mathrm{Fix}(\cdot)$ is a commonly used fixed-point operator in the lambda calculus. Specifically, a fixed-point operation in the form of $\mathrm{Fix}(\lambda f \lambda x.M)$ turns function $M(f, x)$ into a self-referential recursion function $F(x) = M(F, x)$. In the above example, it produces $F(\mathrm{xs}, \mathrm{a}) = G(F, \mathrm{xs}, \mathrm{a})$ from the inner abstraction (C), which denotes the function $G(f, \mathrm{xs}, \mathrm{a})$.

- Application (A): the whole expression (A) is an application in the form of $F\ \mathbf{x}\ 0$, which means applying a function $F$ constructed from (B) to the inputs $\mathbf{x}$ and $0$. $\mathbf{x} = [r_{ij}]_{i \neq j}$ is the variable-length list of pair distances under any fixed ordering. $0$ denotes that the accumulator is initialized as $0$. In particular, after (B),(C), and (D) are composed, $F$ represents

$$F(\mathrm{xs}, a) = \mathrm{if\ Null(xs)\ then}\ a\ \mathrm{else}\ F(\mathrm{Tail(xs)},\ a + V(\mathrm{Head(xs)})). \tag{11}$$

In the lambda calculus, applications are left-associative, so $F\,\mathbf{x}\,0$ means $(F\,\mathbf{x})\,0$. Finally, given $\mathbf{x} = [x_1, x_2, \ldots]$, $F\,\mathbf{x}\,0 = F\,[x_2, \ldots]\,(0 + V(x_1)) = F\,[x_3, \ldots]\,(0 + V(x_1) + V(x_2)) = \cdots$, which just matches the expression of $\sum_k V(x_k)$.

## 3.3 Extended lambda expressions

The adopted lambda expressions in Deflexpressor follow an extended De Bruijn Index style [6]. Specifically, variables are not represented by symbols but by natural numbers, indicating their distance from the bind abstraction that binds them. For example, the expressions $\lambda x.x$ and $\lambda x \lambda y.x$ are written as $\lambda.0$ and $\lambda.\lambda.1$, respectively. This prevents the appearance of equivalent expressions in multiple forms, ensuring that expressions retain only structural information. Thereby, it can further reduce meaningless redundancy and mutations in evolutionary algorithms.

Besides, we have made extensions by introducing typed external quantities, such as functions, constants, and closures. These external quantities are either terms with simple types or functions that take simple types as inputs but output with an unknown type (e.g., an if-else branching). As a concise solution, we only annotate external quantities with types, while the type of the composite term or function output is implicitly inferred.

The extended lambda expression set used in Deflexpressor is formally described as follows.

**Definition 1.** *The set of lambda expressions in Deflexpressor is defined as:*

$$M, N \cdots := n | MN | \lambda.M | C : \mathbb{T}. \tag{12}$$

*In particular, an expression can be in either one of the following forms:*

1. *Variable $n$: a natural number indicating the distance from the variable to its binder $\lambda$. Using De Bruijn indices, binding is resolved without variable names: an occurrence*

*$0$ refers to the nearest enclosing abstraction, and $n$ counts outward across $\lambda$ binders, canonicalizing $\alpha$-equivalent terms and stabilizing evolutionary mutations.*

2. *Application $MN$: the application of two expressions.*

3. *Abstraction $\lambda.M$: the abstraction of an expression.*

4. *External terms $C : \mathbb{T}$: an external constant of type $\mathbb{T}$.*

Extending the Hindley–Milner type system [7], the type system of Deflexpressor is defined as follows.

**Definition 2.** *The type system $\mathbb{T}$ in Deflexpressor is given by:*

$$\mathbb{T} := \mathbb{C}|\mathbb{V}|\mathbb{T} \to \mathbb{T}|\mathbb{T} + \mathbb{T}|\mu\mathbb{V}.\mathbb{T}|List\left[\mathbb{T}\right]. \tag{13}$$

*where $\mathbb{C}$ is the set of basic type constants, $\mathbb{V}$ is the set of type variables, $\mathbb{T} + \mathbb{T}$ stands for sum types, $\mu\mathbb{V}.\mathbb{T}$ stands for fixed-point types, and $List\left[\mathbb{T}\right]$ stands for List types.*

Note that the List type is hard-coded in our system, as lists, matrices, and tensors are extremely common for characterizing the scientific data of complex systems. By doing this, we can avoid more complex type annotations for lists, such as dependent types or subtypes. Combined with Hindley–Milner-style type inference, this typed lambda calculus provides a lightweight static semantics that prunes ill-formed compositions of operators and enforces task-specific input–output signatures before numerical evaluation.

**Type-Checking Mechanism.** The primary utility of the type system within the Deflexpressor framework is to rigorously constrain the search space to the manifold of semantically valid and functionally appropriate expressions. In the context of symbolic regression, randomly generated or naively mutated structures may violate the mathematical definition domains, such as dimensionality mismatches or invalid operator applications. The type system serves as a formal

filter, ensuring that each candidate term $M$ admitted into the population is not only well-formed but also strictly adheres to the input-output signature required by the physical modeling tasks.

To achieve this, we implement a type inference engine based on a modified Algorithm W. Specifically, the W algorithm [7] is a standard procedure for inferring an expression's type by recursively collecting and solving type equations. Our modified version mainly incorporates our extensions for the lambda calculus, including the List type, $\mu$-type, and so on. The main procedures are summarized in Alg. 1 (please refer to the provided source code for implementation details). For any newly generated term $M$, the algorithm attempts to deduce its principal type $\tau$. This process fulfills two critical verification steps. First, it checks for internal consistency. If the recursive inference fails due to conflicting constraints (e.g., applying a scalar operator to a functional argument), the term is identified as ill-typed. Second, for well-typed terms, the inferred type $\tau$ is checked against the requisite target type $\mathbb{T}_{req}$ (e.g., $\mathbb{R}^n \to \mathbb{R}$ for an energy function). Only terms where $\tau$ unifies with $\mathbb{T}_{req}$ are retained. This mechanism effectively guarantees that the evolutionary search operates exclusively on the subspace of valid mathematical formulas. In Alg. 1, this process corresponds to calling $\textsc{TypeCheck}(M, \mathbb{T}_{req})$: it first infers a principal type $\tau$ for $M$ (rejecting $M$ if inconsistent), and then checks whether $\tau$ can be made equal to the required signature $\mathbb{T}_{req}$. Concretely, InferPrincipalType delegates the recursive walk over $M$ to InferAndCollect (which accumulates type constraints) and then uses SolveConstraints and ApplyAssignments to produce the final inferred type.

For better understanding, we give examples of both valid and invalid expressions for a specific task. In particular, we consider a task requiring a scalar function of a real-valued variable, imposing the target type requirement $\mathbb{T}_{req} = \mathbb{R} \to \mathbb{R}$.

1. **Valid Candidate**: A typical example is $M_1 = \lambda x.(x{+}1)$. The inference algorithm assigns a type variable $\alpha$ to $x$. Given the signature of the addition operator $(+) : \mathbb{R} \to \mathbb{R} \to \mathbb{R}$, the constraint $\alpha = \mathbb{R}$ is derived. The resulting type is $\mathbb{R} \to \mathbb{R}$, which unifies with $\mathbb{T}_{req}$,

**Algorithm 1** Modified Algorithm W used for type checking.

**function** TYPECHECK($M, \mathbb{T}_{req}$)
    $\tau \leftarrow$ INFERPRINCIPALTYPE($M$) ▷ fail if inconsistent
    **return** UNIFY($\tau, \mathbb{T}_{req}$) ▷ kept iff types can be made equal
**end function**

**function** INFERPRINCIPALTYPE($M$)
    $\langle \tau, \mathcal{C} \rangle \leftarrow$ INFERANDCOLLECT($M$)
    $\mathcal{A} \leftarrow$ SOLVECONSTRAINTS($\mathcal{C}$) ▷ type assignments; fail if none
    **return** APPLYASSIGNMENTS($\mathcal{A}, \tau$)
**end function**

**function** INFERANDCOLLECT($M$)
    **if** $M$ is a variable $x$ **then**
        **return** $\langle \alpha_x, \emptyset \rangle$ ▷ $\alpha_x$ is a fresh type variable for this binder
    **else if** $M$ is a constant/operator $C : \mathbb{T}$ **then**
        **return** $\langle \mathbb{T}, \emptyset \rangle$
    **else if** $M$ is an abstraction $\lambda x.\, M_0$ **then**
        $\langle \tau_0, \mathcal{C}_0 \rangle \leftarrow$ INFERANDCOLLECT($M_0$) ▷ with binder $x$ bound to $\alpha_x$
        **return** $\langle \alpha_x \to \tau_0, \mathcal{C}_0 \rangle$
    **else if** $M$ is an application $M_1\, M_2$ **then**
        $\langle \tau_1, \mathcal{C}_1 \rangle \leftarrow$ INFERANDCOLLECT($M_1$)
        $\langle \tau_2, \mathcal{C}_2 \rangle \leftarrow$ INFERANDCOLLECT($M_2$)
        $\alpha \leftarrow$ FRESHTYPEVAR
        $\mathcal{C} \leftarrow \mathcal{C}_1 \cup \mathcal{C}_2 \cup \{\tau_1 = (\tau_2 \to \alpha)\}$
        **return** $\langle \alpha, \mathcal{C} \rangle$
    **end if**
**end function**

rendering $M_1$ a valid candidate.

2. **Ill-typed Construction**: A typical example is $M_2 = 1 + (\lambda x.x)$. The operator $+$ demands arguments of type $\mathbb{R}$. While $1$ is of type $\mathbb{R}$, the term $(\lambda x.x)$ possesses a functional type $\beta \to \beta$. The unification of $\mathbb{R}$ with $\beta \to \beta$ is impossible within this type system, causing the inference to fail and $M_2$ to be rejected as semantically invalid.

3. **Type Mismatch**: Consider $M_3 = 5$ as an example. The inference successfully assigns

it the type $\mathbb{R}$. However, the task requires a function type $\mathbb{R} \to \mathbb{R}$. Since the scalar type $\mathbb{R}$ does not unify with the function type $\mathbb{R} \to \mathbb{R}$, $M_3$ is rejected for failing to meet the interface requirements of the regression task.

### 3.4 Generation and mutation for lambda calculus expressions

The lambda expressions are stored as binary trees, where abstraction nodes have one child, application nodes have two children, and other nodes represent leaves. To navigate the combinatorial search space effectively, Deflexpressor employs a hybrid strategy that integrates explicit rule-based mutations with a neural-guided generation mechanism. The balance between these two approaches is dynamically adjusted via exponential annealing: the proportion of explicit mutations gradually decreases from 100% to 0%, allowing the system to transition from robust structural exploration to fine-grained, learned optimization.

**Rule-based Expression Generation.** The explicit mutations serve as the foundational operations, ensuring the validity and interpretability of the expressions. These include standard tree manipulations and lambda-calculus-specific transformations:

- Subtree mutation: A subtree is pruned and replaced by a newly generated one. If the current subtree has a valid type (i.e., it can be evaluated by normalization), the newly generated subtree either has an unknown type or matches the type of the original subtree.

- Crossover: A subtree from one expression is replaced with a subtree from another expression. In the case of typed subtrees, the same typing rules as for subtree mutation apply.

- Promotion: A subtree is replaced by one of its own subtrees, following the same type rules as the subtree mutation.

- Point-wise mutation: A leaf node is replaced by another leaf node, following the same type rules as the subtree mutation.

Additionally, we introduce some unique mutations for lambda expressions [5, 8]:

- $\beta$-Reduction: An expression or a sub-expression undergoes $\beta$-reduction.
- Common subexpression elimination (CSE): Identical sub-expressions within a subtree are replaced with variables, and the entire subtree will be expressed as an application of the common sub-expression to an abstracted subtree. This is the primary method for combating expression bloat.
- $\eta$-Reduction: Constant expressions are simplified to their constant values.

For clarity, $\beta$-reduction is the core evaluation rule that rewrites an application $(\lambda.M)\,N$ by substituting the argument into the body, yielding $M[N]$; iterating such rewrites (together with optional $\eta$-reductions) corresponds to normalizing a term when a normal form exists. Note that transformations like $\beta$-reduction are not enforced during mutation, as different forms may lead to diverse mutation pathways. These mutations are designed for efficient exploration in the expression space while ensuring that the expressions remain interpretable.

**Neural-guided Expression Generation.** As a complement to the explicit rule-based mutations, Deflexpressor integrates a causal autoregressive neural network to generate more diverse expressions efficiently. The Deflexpressor network is essentially a Transformer decoder [9], configured with 6 attention layers, a hidden dimension of 64, and 8 attention heads. It operates on serialized token sequences of lambda expressions. It is trained offline via a standard language modeling objective (next-token prediction) on a dataset constructed by the rule-based engine. The training corpus comprises 128,000 syntactically valid expressions for *ab initio* generation and 54,000 expression pairs for mutation learning. In these pairs, the target was derived from

the source through 2 to 5 steps of random rule-based mutations, capturing non-trivial structural transitions. We used the AdamW optimizer with a learning rate of $5 \times 10^{-4}$ and a batch size of 32, training for 200 epochs. In particular, no special task tokens are required. The network implicitly distinguishes modes based on the input context. In the generation mode, it is iteratively called to produce an expression from an empty context until the expression conforms to the type signatures in type check. In the mutation mode, it uses an existing expression as a prompt to generate a structural variant that can pass the type check. During the evaluation of each candidate, we first check its type and then normalize it (via $\beta/\eta$ reductions) to a value. The candidates that violate the expected types or fail to normalize are marked as invalid. This ensures the generation of executable terms rather than purely syntactic strings.

**Three-Phase Elimination Mechanism.** To balance efficiency and population diversity in the evolutionary algorithms, we propose a three-phase elimination mechanism. In the first phase of mutation, we set a breeding population size $N_p$. The top $N_p$ individuals with the highest fitness can reproduce two offspring, while the remaining individuals reproduce only one. Later in the second phase, with the increase of expression population, we define a flexible population threshold $M_s$. If the total population exceeds $M_s$, the individuals with the lowest fitness are eliminated with a probability $P_d$. Finally, to limit the population, a rigid threshold $M_r$ is enforced. That is, once the population exceeds $M_r$, the individuals with the lowest fitness are eliminated immediately. Expressions that cannot be normalized or result in evaluation errors are assigned a fitness value lower than any valid expression. Nonetheless, these expressions are not subject to immediate elimination unless the population exceeds $M_r$. This design preserves potentially useful genetic diversity from erroneous expressions during evolution.

## 3.5 Initialization with global aggregation priors

Global properties (e.g., total energy) of complex systems often stem from the aggregation effects of all elements. However, standard SR initialization via random expression generation tends to produce formulas operating on a limited number of variables, making it inefficient to discover structures that iterate over massive input sets (e.g., summing over $N$ particles).

To address this, we incorporate a heuristic initialization strategy that seeds a portion of the population with "Reduce-like" structures. These expressions are constructed as standard lambda terms that recursively apply a learnable binary operator, denoted as $\mathrm{kernel}$, to the input list $\mathbf{x} = [x_1, \ldots, x_N]$. For example, if the underlying physical law is a summation $E = \sum \phi(x_i)$, this initialization provides the skeleton of the aggregation loop, allowing the SR algorithm to focus on evolving the $\mathrm{kernel}$ (e.g., finding $\mathrm{kernel}(s, x) = s + \phi(x)$) rather than constructing the loop mechanism from scratch.

Technically, this reduction is implemented purely within the lambda-calculus framework. The recursive expression follows the form: $s_1 \leftarrow x_1$, and $s \leftarrow \mathrm{kernel}(s, x_i)$ for $i > 1$, as described in Eq. 5 of the Methods section in the main text. Importantly, this structure serves merely as a soft bias rather than a hard constraint. Since the Reduce structure is treated as a regular lambda term, subsequent evolutionary mutations can freely modify, dismantle, or discard it if the data suggests that a global aggregation is unnecessary.

**A concrete example: recursive summation over a variable-size list.** To make the above "Reduce-like" recursion explicit without assuming a lambda-calculus background, consider the classical interaction energy $E = \sum_{i \neq j} V(r_{ij})$ in the main text. Let $\mathbf{x} = [x_1, \ldots, x_m]$ denote a list that enumerates the interaction descriptors to be aggregated; for example, $x_k = r_{ij}$ can enumerate all particle pairs $i \neq j$ under a fixed deterministic ordering. For commutative aggregations such as summation, this ordering does not affect the result because any permutation of

the same multiset gives the same aggregate. For non-commutative kernels, the fixed ordering simply makes the recursive aggregation deterministic. Define the summation procedure as a standard recursion with an accumulator $a$:

$$\mathrm{Sum}(\mathrm{xs}, a) = \begin{cases} a, & \mathrm{null}(\mathrm{xs}), \\ \mathrm{Sum}(\mathrm{tail}(\mathrm{xs}),\ a + V(\mathrm{head}(\mathrm{xs}))), & \text{otherwise}, \end{cases} \tag{14}$$

so that $E = \mathrm{Sum}(\mathbf{x}, 0)$. In the expression language of Deflexpressor, this same recursion is written by first specifying a *non-recursive template* that receives the would-be recursive function $f$ as an explicit argument, and then applying the fixed-point operator to obtain the corresponding recursive function:

$$E \ = \ \underbrace{\Big(\mathrm{Fix}\ (\lambda f\ \lambda \mathrm{xs}\ \lambda a.\ \mathrm{if}\ (\mathrm{null\ xs})\ \mathrm{then}\ a\ \mathrm{else}\ f\ (\mathrm{tail\ xs})\ (a + V(\mathrm{head\ xs})))\Big)}_{\text{a recursive function equivalent to Sum}}\ \mathbf{x}\ 0. \tag{15}$$

Unrolling the recursion for $\mathbf{x} = [x_1, x_2, \ldots, x_m]$ makes the connection to the summation symbol immediate:

$$\begin{aligned} E &= \mathrm{Sum}([x_1, \ldots, x_m], 0) \\ &= \mathrm{Sum}([x_2, \ldots, x_m], 0 + V(x_1)) \\ &= \mathrm{Sum}([x_3, \ldots, x_m], 0 + V(x_1) + V(x_2)) \\ &\ \vdots \\ &= 0 + \sum_{k=1}^{m} V(x_k). \end{aligned} \tag{16}$$

This step-by-step expansion is what the fixed-point operator $\mathrm{Fix}$ provides operationally: it turns a one-step update rule into an executable aggregation over variable-length inputs.

### 3.6 Expression complexity

Finally, we use the length of the expressions as a measure of their complexity, with specific rules outlined in Supplementary Table 1. The length of an external term is calculated as $2 + l(c)$.

The length 2 reflects the processes of abstracting the external quantity into a variable, which effectively generates a length of 2. This abstraction involves treating the external quantity as a bound variable in the lambda calculus. Following this step, the external quantity is then applied to the expression. This approach encourages Deflexpressor to prioritize obtaining a quantity through lambda calculus before introducing an external one.

Supplementary Table 1: Length rule of terms

| Term | Length | Description |
|---|---|---|
| Variable $n$ | 1 | - |
| Application $MN$ | $l(M) + l(N) + 1$ | $l(M)$ and $l(N)$ are the lengths of $M$ and $N$. |
| Abstraction $\lambda.M$ | $1 + l(M)$ | $l(M)$ is the length of the body of the abstraction. |
| External terms $c$ : $\mathbf{T}$ | $2 + l(c)$ | $l(c)$ is the length of the value of the external term. |
| Integers | 1 | as external values. |
| if-then-else | 2 | as external values. |
| Elementary functions | 1 | as external values. |
| Floating-point numbers | 4 | as external values. |

# 4 Experimental setup

## 4.1 Comparison setup

We evaluate the performance of our proposed Deflex with a set of state-of-the-art methods, including Operon [10], SciMED [11], SINDy [12], gplearn, AI Feynman 2.0 [13, 14], PySR, and DEAP [15]. These frameworks have distinct capabilities and limitations:

- AI Feynman 2.0 is the only one that has built-in support for distribution fitting.
- SINDy is based on sparse regression instead of symbolic trees.
- SciMED, gplearn, and DEAP are all tree-based symbolic regression methods with GP. Among them, DEAP and gplearn are classic SR frameworks based on evolutionary al-

gorithms, while SciMED is a recent physics-inspired method based on Monte Carlo tree searching.

- Operon has been one of the SOTA symbolic regression methods according to SRBench [16].
- The APIs for Operon and SciMED do not permit the addition of custom symbols.
- Most frameworks, except DEAP and parts of gplearn based on it, do not support abstract data types for symbolic nodes, which precludes the use of higher-order functions like map.
- None of these frameworks include higher-order operators natively.

To ensure maximum comparability across these diverse frameworks, we implemented a series of adaptations. For frameworks lacking native higher-order operators (e.g., map, sum), we first attempted to add these operators into their symbol sets. If a framework could not incorporate these operators, and the target expression uses them at the top level, for instance, a formula of the form `result = sum(map(f, data))`, we adapted the task. The framework was made responsible for fitting only the underlying sub-expression (f), and the top-level operators were then applied manually by us within the loss function to evaluate the final error. Similarly, for tasks involving distribution fitting, we provided a custom Negative Log-Likelihood (NLL) loss function for frameworks lacking this capability. Note that some of these frameworks have variants that would gain better performance with scientists in the loop (e.g., manually providing feature sets or constraints on the expression tree for SciMED and AI Feynman, see Subsection 7.8 of SI for more detailed discussions and evaluation results), while we mainly used the raw version for simplicity despite the above adaptations.

## 4.2 Configurations of compared methods

Beyond the above general setups for comparison, we adhered to the algorithmic configurations and hyperparameter settings recommended in the respective official documentation, which normally reflect standard usage scenarios. The specific implementation details for each method are delineated below.

**Operon**: We deployed the Operon framework, a high-performance C++ library for symbolic regression. The algorithm was configured to utilize its analytical gradient-based optimization capabilities to fine-tune scalar coefficients within the expression trees. We set the population size to $1,000$ individuals and allowed the evolutionary process to run for up to $1,000$ generations, subject to the global time limit. Selection was performed using a tournament size of 5, and we employed the standard suite of genetic operators including subtree crossover and mutation with the library's default probability distributions.

**SciMED**: The SciMED (Scientist-Machine Equation Discovery) method [11] was implemented utilizing its physics-informed Monte Carlo Tree Search (MCTS) architecture. We maintained the default exploration-exploitation balance governed by the Upper Confidence Bound (UCB) exploration constant $c_p = 1.0$. The search process was constrained to expression trees of maximum depth comparable to other tree-based methods to ensure a fair exploration of the functional space. The method's unique capability to leverage physical unit constraints was enabled where variable dimensions were applicable.

**SINDy**: Sparse Identification of Nonlinear Dynamics (SINDy) [12] was executed using the STLSQ (Sequentially Thresholded Least Squares) optimizer. To balance sparsity and accuracy, we set the threshold parameter to $0.1$ and allowed for a maximum of 20 iterations for the sparse regression coefficients to converge. The library of candidate functions was constructed to include polynomial terms up to degree 3 as well as trigonometric functions (sine and cosine), providing a comprehensive basis for discovering governing dynamical equations.

**gplearn**: We utilized the `SymbolicRegressor` module from the `gplearn` library, which implements a canonical genetic programming algorithm. The evolution was initialized with a population of $1,000$ programs and evolved over 20 generations. To mitigate the phenomenon of code bloat, a parsimony coefficient of $0.001$ was applied to the fitness function, penalizing overly complex expressions. The genetic operations were configured with standard probabilities: crossover ($0.9$), subtree mutation ($0.01$), hoist mutation ($0.05$), and point mutation ($0.01$).

**AI Feynman 2.0**: This physics-inspired framework [13, 14] was deployed with its full recursive strategy enabled. The method integrates neural network fitting to approximate the underlying function, followed by a battery of structural tests including dimensional analysis, symmetry detection, and separability checks. We utilized the default settings for the internal neural network training (epochs and architecture) to act as a reliable proxy for the data. The brute-force search component, invoked for identifying low-dimensional sub-problems, was restricted by the global timeout to prevent excessive computational overhead on high-dimensional tasks.

**PySR**: We employed the `PySR` package, which leverages a high-performance backend for regularized evolution. The search was distributed across multiple populations with a size of 33 individuals each, running for 100 iterations per restart cycle. The complexity-accuracy tradeoff was managed via the default parsimony settings. We utilized the standard set of unary and binary operators (addition, subtraction, multiplication, division, sine, cosine, and exponential) to define the search space, allowing the algorithm to optimize both the structure and constants of the candidate equations.

**DEAP**: A standard Genetic Programming (GP) pipeline was constructed using the `DEAP` evolutionary computation framework [15]. This baseline implementation followed the classic Koza-style GP paradigm [8]. The evolutionary parameters were set to a population size of 500

evolving over 50 generations. Tree initialization was performed using the ramped half-and-half method with a depth limit of 17 to prevent memory overflow. Genetic operators included one-point crossover and uniform mutation, configured with probabilities of $0.5$ and $0.1$ respectively, to drive the search through the program space.

### 4.3 Coarse-graining and renormalization protocols

To rigorously evaluate the capability of Deflex in discovering multiscale laws, we introduce a renormalization analysis framework [17]. This involves applying specific coarse-graining operators $\mathcal{R}_\lambda$ with a scale factor $\lambda$ to the system, and examining whether the method can independently identify the renormalized physical laws at each scale. We adopt two distinct coarse-graining protocols tailored to the nature of the simulated systems: re-simulation for particle systems and data aggregation for continuous fields.

For discrete particle systems (specifically the water particle experiment), we performed coarse-graining at the initialization stage and conducted re-simulations. We define a set of scale factors $\lambda \in \{1, 3, 10\}$, representing the ratio of the characteristic length of the coarse-grained particles to the original molecules. At the initial frame, we employ a distance-based clustering approach to group water molecules into macro-particles. Assuming the characteristic distance between molecules is $d_0$, the clustering threshold is set to $\lambda d_0$, which scales linearly with the coarse-graining factor. Molecules within this threshold distance are grouped into a single macro-particle. The physical properties of these macro-particles are calculated to serve as the initial conditions for a new run of simulations. Specifically, for a macro-particle $I$ consisting of a set of molecules $S_I$, its mass $M_I$, position $\mathbf{R}_I$, and velocity $\mathbf{V}_I$ are initialized as:

$$M_I = \sum_{i \in S_I} m_i, \quad \mathbf{R}_I = \frac{1}{M_I} \sum_{i \in S_I} m_i \mathbf{r}_i, \quad \mathbf{V}_I = \frac{1}{M_I} \sum_{i \in S_I} m_i \mathbf{v}_i. \tag{17}$$

where $m_i$, $\mathbf{r}_i$, and $\mathbf{v}_i$ denote the mass, position, and velocity of individual molecule $i$, respectively. The system is then evolved using the same interaction potentials but scaled parameters,

generating the "ground truth" data for each scale $\lambda$.

For continuous fluid dynamics, we applied a standard data coarse-graining approach to the high-resolution simulation outputs. The scale factor $\lambda$ varies continuously from 1 to 100. We partitioned the computational grid into blocks of size $\lambda \times \lambda$. The coarse-grained fields (e.g., velocity $\mathbf{u}'$) are obtained by block averaging the microscopic fields ($\mathbf{u}$):

$$\mathbf{u}'(x,y) = \frac{1}{\lambda^2} \sum_{i,j \in \text{Block}(x,y)} \mathbf{u}(i,j). \tag{18}$$

where $\mathbf{u}'(x,y)$ denotes the coarse-grained velocity field, and $\text{Block}(x,y)$ represents the $\lambda \times \lambda$ spatial block centered at $(x,y)$. This process generates coarse-grained datasets representing the flow at macroscopic scales.

Renormalization refers to a common feature or process in many multiscale systems, where a formula describing a system at a fine-grained level can be transformed into one with the same form at a coarse-grained level by just changing the scale-related parameter. Taking the Maxwell-Boltzmann distribution as a concrete example: at the microscopic scale ($\lambda = 1$), the speed distribution of particles with mass $m$ follows $f(v) \propto v^2 \exp(-\frac{mv^2}{2k_BT})$ where $v$ denotes the particle speed, $m$ is the particle mass, $k_B$ is the Boltzmann constant, and $T$ is the temperature. When coarse-grained to scale $\lambda$, if the macro-particles (now with mass $M \approx \lambda^3 m$) reach thermal equilibrium, the law formula renormalizes to the same functional form but with a scaled parameter: $f(V) \propto V^2 \exp(-\frac{MV^2}{2k_BT})$.

To validate whether Deflex can recover the renormalization group flow, we compared the distribution predicted by Deflex against two baselines: 1) the distribution of the actual coarse-grained data (Ground Truth), and 2) the theoretical renormalized formulas.

## 4.4 Observation scale and sampling strategy

The scale of observation refers to the spatiotemporal scope from which observational data $\mathbf{X}$ are collected. Unlike the coarse-graining scale factor $\lambda$ that characterizes the physical resolu-

tion of the system itself, the observation scale characterizes the data sampling strategy, defining whether the collected data points reflect local clustered interactions or global macroscopic behaviors. Importantly, these two concepts are independent: one can perform small-scale (localized) or large-scale (random) observations on a system at any coarse-graining level $\lambda$.

For small-scale observations, we adopt a two-level sampling approach to capture localized spatiotemporal patterns. First, we randomly sample an initial set of seed points across time (frames) and space (elements or spatial positions). Then, we draw additional sample points from the spatiotemporal vicinity of these seeds. For particle systems, we sample neighboring particles within a certain spatial radius around the seed particle. For continuous fields, we extract a compact spatial region (e.g., a $4 \times 4$ grid block) centered at the seed position. This localized sampling facilitates the discovery of governing equations such as differential equations.

For large-scale observations, we employ uniform random sampling over the entire spatiotemporal domain. Sample points are drawn independently and uniformly across all available frames and spatial elements, without preferential clustering. This promotes the discovery of macroscopic statistical patterns such as velocity distributions.

In practice, we alternate or combine both sampling strategies depending on the target discovery. For example, in fluid systems, small-scale observations via localized spatial regions facilitate the recovery of differential equations, while large-scale random sampling reveals emergent statistical patterns.

## 4.5 Argon particle motion

We conducted simulations using argon particles at three temperature points near the critical point: $T_c - 1$K (149.72K), $T_c$ (150.72K), and $T_c + 1$K (151.72K), where $T_c = 150.72$K is the critical temperature of argon. The density was maintained constant at 31.3 mol/L across all three temperature points, while pressure and other thermodynamic properties adjusted naturally

according to the equation of state (Supplementary Fig. 2). By treating these three conditions as different thermodynamic states of the same system, we enable the discovered formulas to capture temperature-dependent behaviors, where temperature appears as an independent variable in the learned relationships. These particles were confined within a 2-unit $\times$ 2-unit $\times$ 2-unit cubic field and subjected to a potential field of $1\text{E3} \times r^2$ for confinement. The repulsive force between two particles followed an inverse square relationship with a constant of 1E2. We performed particle motion simulations using the high-performance computing library of Taichi, with a time step of 1E-2 units per frame. A total of 1E5 frames were collected to form the dataset.

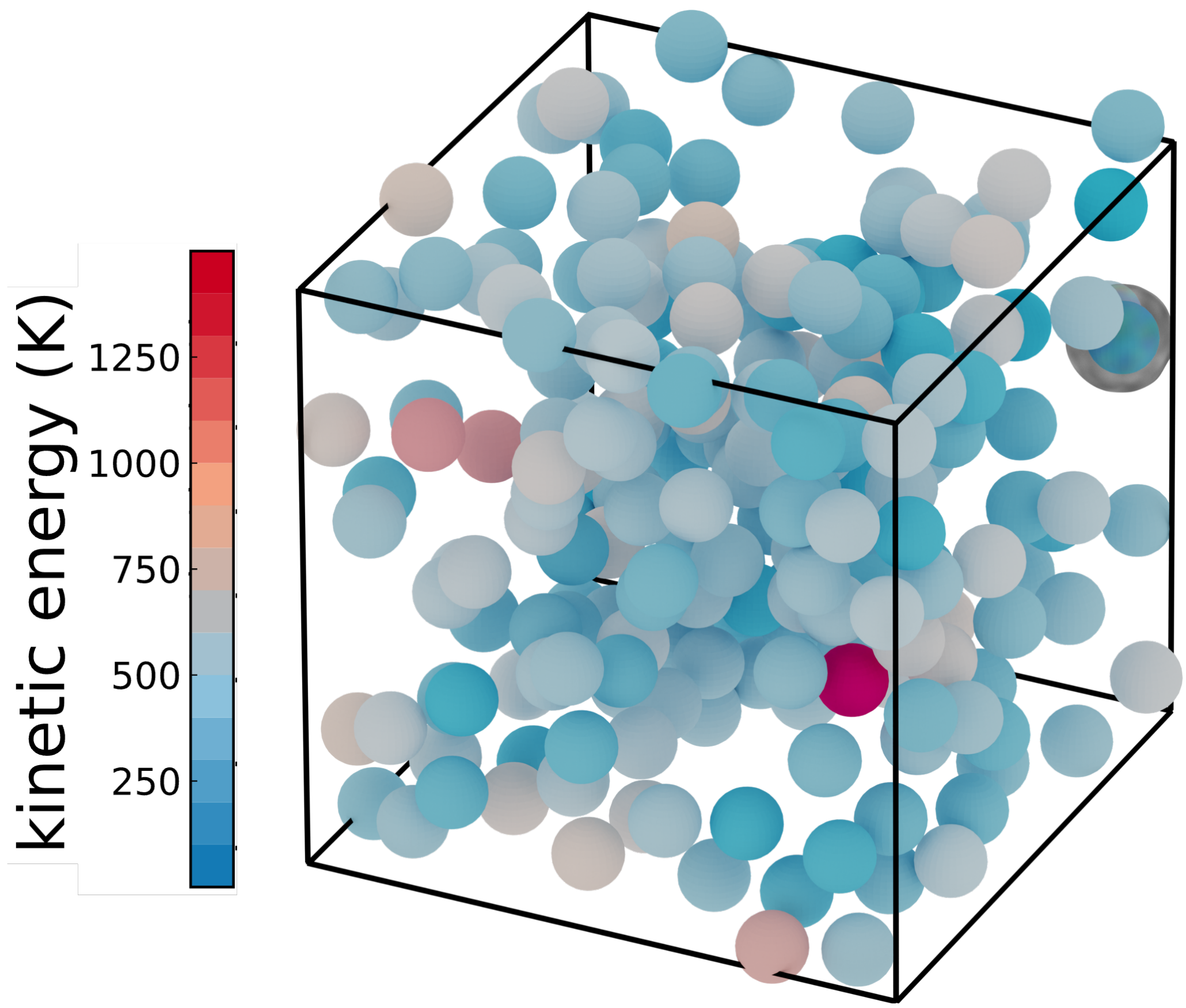


Supplementary Figure 2: Argon particles at three temperature points near the critical point ($T_c - 1\text{K}$, $T_c$, $T_c + 1\text{K}$, where $T_c = 150.72\text{K}$) with constant density 31.3 mol/L.

We trained the neural network for a total of 150,000 epochs and followed it with 150,000

steps to train the symbolic regression model. During neural network training, we randomly selected 40 consecutive or random frames, and within each frame, we used the location, velocity and mass of 10 particles, either randomly sampled from all particles, or from neighboring particles around a specific particle. The batch size was set to 16. These particles served as the training set for a single step.

For training the symbolic regression model, we used a population size ranging from 20 to 5, employing cosine annealing. Each step underwent a constant mutation with an 80% mutation probability.

To discover the underlying physical laws governing argon particle motion, we fed the symbolic regression module with the fundamental kinematic and dynamic state variables of the particles. It is worth noting that for all the symbolic regression methods compared in this study, the input features provided were identical to ensure a fair comparison. As detailed in Supplementary Table 2, these inputs include the position, velocity, mass, and time information for each observed particle. Additionally, temperature is provided as an input variable since data from three thermodynamic states ($T_c - 1$K, $T_c$, $T_c + 1$K) are combined in training, allowing the algorithm to discover temperature-dependent formulas where $T$ appears as an independent variable in the discovered energy expressions.

Supplementary Table 2: Features for Symbolic Regression in Argon Particle Experiment

| Type | Feature | Description |
|---|---|---|
| Element-wise | `pos_x`, `pos_y`, `pos_z`<br>`vel_x`, `vel_y`, `vel_z`<br>`time` | Coordinates of the particle in the 3D field.<br>Velocity components of the particle.<br>Simulation timestamp. |
| Global | `mass`<br>`temperature` | Mass of the particle (constant for all particles).<br>System temperature (varies across three states: 149.72K, 150.72K, 151.72K). |
| Scale | $N, T_{obs}$ | 100 particles $\times$ $100,000$ frames across three temperature points. |

## 4.6 Pollen particle motion

We used the same Taichi simulation framework [18] to simulate the motion of pollen particles in a 3D tube. The tube has a radius of 0.5 micrometers and a length of 5 micrometers. There are 400 pollen particles with diameters ranging from 30 to 50 micrometers sampled from a truncated normal distribution. The density of the pollen particles is 1.5 times that of water. The simulation was conducted under standard conditions at 300K.

For the neural network training and symbolic regression, we used the same settings as the previous experiments for Argon particles. Each data frame was collected at every 20 time steps, and the simulation was conducted for 1E4 time steps.

For the pollen particle system, the symbolic regression task aimed to capture the stochastic dynamics in a fluid environment. Consistent with the other experiments, all comparative methods received the exact same input features. The input features provided to the model are listed in Supplementary Table 3, comprising the full phase space coordinates (position and velocity) as well as the individual physical properties (radius and density) of the heterogeneous pollen grains.

Supplementary Table 3: Features of Observation Data in Pollen Particle Experiment

| Type | Feature | Description |
|---|---|---|
| Element-wise | `pos_x`, `pos_y`, `pos_z` | 3D coordinates of the pollen particle in the tube. |
| | `vel_x`, `vel_y`, `vel_z` | Velocity components. |
| | `radius` | Radius of the pollen particle (30–50 $\mu$m). |
| | `density` | Particle density ($1.5\times$ water). |
| | `time` | Simulation timestamp. |
| Global | `temperature` | System temperature (300K). |
| Scale | $N, T_{obs}$ | 400 particles $\times$ $10,000$ frames. |

## 4.7 Human mobility

We used the human mobility dataset from the Zurich Carnival dataset [19] in the experiment. The dataset contains the GPS locations of 1000 people during the Zurich Carnival. We used it to train the neural network and symbolic regression models, each with 15,000 epochs or steps.

In the human mobility experiment, the goal was to identify movement patterns from GPS traces. We provided the symbolic regression algorithm with the raw spatiotemporal data points as shown in Supplementary Table 4. Note that no user identifiers were provided to the model, ensuring that the learned laws rely solely on the spatiotemporal dynamics. The latitude, longitude, and timestamps served as the basis for discovering flow patterns and potential gathering behaviors.

Supplementary Table 4: Features for Symbolic Regression in Human Mobility Experiment

| Type | Feature | Description |
|---|---|---|
| Element-wise | `lat`, `lon`<br>`time` | Latitude and longitude from GPS data.<br>Timestamp of the record. |
| Scale | $N, T_{obs}$ | $1,000$ users $\times$ Daily trajectories (Carnival). |

## 4.8 Bird migration

For the bird migration experiment, we used the reanalysis from the eBird dataset [20]. The dataset contains the GPS locations of birds around the world during their migration. In the experiment, we randomly chose 100,000 birds and 1,000 days from the dataset. The training settings and the algorithm setup were the same as those in the human mobility experiment.

The bird migration analysis utilized global positioning data to uncover migration routes and timings. Supplementary Table 5 outlines the features used, primarily focusing on the geospatial coordinates and temporal markers essential for reconstructing seasonal migration trajectories.

Supplementary Table 5: Features of Observation Data in Bird Migration Experiment

| Type | Feature | Description |
| --- | --- | --- |
| Element-wise | `lat, lon` | Global latitude and longitude. |
| | `time` | Temporal information of the observation. |
| Scale | $N, T_{obs}$ | $100,000$ birds $\times$ $1,000$ days. |

## 4.9 Fluid motion

We utilized the Taichi simulation framework [18] to conduct Direct Numerical Simulation of two-dimensional incompressible flow past a circular cylinder. The computational domain measured 10 units in length and 5 units in width, with a cylinder of radius 1 unit positioned in the flow. The incoming flow velocity was set to 1 unit per frame. A uniform Cartesian mesh of $1024 \times 1024$ grid points was employed for spatial discretization. The simulation was performed at Reynolds number $Re = 100$ with a time step of $\Delta t = 0.01$ (Supplementary Fig. 3). Under these conditions, the flow exhibits periodic vortex shedding characteristic of the von Kármán vortex street.

Data collection followed a multi-scale sampling strategy to capture both local and mesoscale flow structures. Temporal data were acquired over 2,500 simulation steps, with snapshots collected every 40 frames, resulting in a total of 128 training snapshots spanning multiple vortex shedding cycles. At each training step, spatial sampling was conducted using two approaches: (1) random point sampling, selecting 16 individual grid points uniformly distributed across the computational domain to capture diverse flow conditions; (2) structured cluster sampling, extracting $4 \times 4$ square regions containing spatially coherent flow information, which enables the algorithm to learn spatial correlations and gradient patterns within local neighborhoods. The random sampling probes the global statistical properties of the flow field, while cluster sampling focuses on capturing localized differential relationships.

Each sampled location provided the two-dimensional velocity field $(u, v)$, pressure $p$, and

their first-order spatial derivatives $\partial u/\partial x$, $\partial u/\partial y$, $\partial v/\partial x$, $\partial v/\partial y$, $\partial p/\partial x$, and $\partial p/\partial y$. The spatial derivatives were computed using second-order central finite differences on the simulation grid. All variables were maintained in dimensionless form throughout the simulation and training process, with length scales normalized by the cylinder radius, velocity by the incoming flow velocity, and pressure by the dynamic pressure $\rho U^2$. The batch size for neural network training was set to 16, with each batch randomly drawing from either the random or cluster sampling pool with equal probability.

The training of symbolic regression and neural network models was conducted using the same configuration as the pollen motion experiment. The neural network was trained for 15,000 epochs using the Adam optimizer with an initial learning rate of $10^{-3}$, which decayed exponentially at a rate of 0.95 every 1,000 epochs to ensure stable convergence. This was followed by 15,000 steps of symbolic regression employing genetic programming with lambda-aware mutations to efficiently explore the space of mathematical expressions. The combined training typically required approximately 185 seconds on a single NVIDIA Tesla V100 GPU for this experiment.

To further test the generalizability of our approach, we incorporated high-quality turbulence data from the Johns Hopkins Turbulence Database (JHTDB) as a supplementary validation dataset. The JHTDB provides Direct Numerical Simulation (DNS) results of forced isotropic turbulence [21], representing a canonical configuration free from domain boundary effects. We utilized the Forced Isotropic Turbulence dataset with a spatial resolution of $1024^3$ grid points. This dataset features homogeneous and isotropic turbulence maintained in a statistically steady state through large-scale forcing, with a Taylor microscale Reynolds number $Re_\lambda \approx 433$. All field variables, including velocity components, pressure, and spatial coordinates, were accessed in their dimensionless form as provided by the JHTDB interface.

Data acquisition followed the same multi-scale sampling protocol as the cylinder wake ex-

periment. We extracted 128 temporal snapshots from the database spanning the available time range. For spatial sampling, we employed two strategies to probe different scales: (1) random point sampling, where 16 spatial locations were randomly selected from the $1024^3$ domain at each training step; (2) structured cluster sampling, where spatially coherent cubic regions were extracted. Due to the three-dimensional nature of the flow, cubic clusters comprised fewer total grid points compared to the two-dimensional square regions in the cylinder experiment to maintain computational tractability. Specifically, while the cylinder experiment used $4 \times 4$ square patches containing 16 grid points, the JHTDB experiment employed smaller cubic volumes. Each sampled location, whether from random or cluster sampling, provided the local three-dimensional velocity field $\mathbf{u} = (u, v, w)$, pressure field $p$, and their first-order spatial derivatives $\nabla\mathbf{u}$ and $\nabla p$.

The spatial derivative fields $\nabla\mathbf{u}$ and $\nabla p$ were obtained directly from the JHTDB database using its built-in differentiation functions, which employ fourth-order accurate finite difference schemes on the underlying DNS grid. This provides high-fidelity gradient information essential for discovering differential equations. The 128 temporal snapshots were uniformly distributed across multiple vortex turnover times to ensure adequate coverage of the turbulent dynamics. At each snapshot, the random point sampling strategy uniformly sampled locations from the entire $1024^3$ domain, ensuring statistical representativeness of the turbulent field. The structured cluster sampling, on the other hand, extracted spatially localized cubic regions to capture coherent flow structures such as vortex tubes and strain-dominated regions. The combination of these two sampling strategies enables the algorithm to learn both point-wise local dynamics and spatially correlated patterns.

The training configuration mirrored that of the cylinder wake experiment. The neural network training was conducted for 15,000 epochs using the Adam optimizer with an initial learning rate of $10^{-3}$, decaying exponentially with a rate of 0.95 every 1,000 epochs. This was

followed by 15,000 steps of symbolic regression using the genetic programming engine with lambda-aware mutations. The batch size remained at 16, with each batch containing samples from either random or cluster sampling selected randomly with equal probability. The loss function combined the energy-based model objective for the neural network phase and the Pareto-optimal multi-objective fitness for the symbolic regression phase, balancing accuracy and complexity as detailed in Section S1.

Importantly, Deflex successfully recovered the three-dimensional Navier-Stokes equation from the JHTDB data, validating the robustness of our approach across different flow regimes. The discovered formulas exhibited consistent functional forms with those from the cylinder wake experiment, differing only in fitted coefficients that reflect the distinct flow physics. Specifically, the convective terms $(\mathbf{u} \cdot \nabla)\mathbf{u}$, pressure gradient $\nabla p$, and viscous diffusion $\nu \nabla^2 \mathbf{u}$ were all correctly identified in their three-dimensional forms. However, the Newton viscosity law was not independently identified in the JHTDB dataset, likely due to the fully developed turbulent nature of the flow where viscous effects are dominated by turbulent stresses at the resolved scales. This observation is physically consistent with the high Reynolds number regime of the JHTDB simulation, where turbulent transport dominates over molecular viscosity at the scales accessible to our sampling resolution.

The successful discovery of the Navier-Stokes equation in both experiments demonstrates several key capabilities of the Deflex framework. First, the algorithm correctly identified the nonlinear convective acceleration terms $(\mathbf{u} \cdot \nabla)\mathbf{u}$, which involve products of velocity components and their spatial derivatives. This requires the genetic programming engine to construct composite expressions from elementary features, a task that is challenging for many symbolic regression methods. Second, the pressure gradient terms $\nabla p$ and viscous diffusion terms $\nu \nabla^2 \mathbf{u}$ were correctly represented using the pre-computed spatial derivative features. The algorithm learned that these terms should appear linearly in the momentum equation, consistent with the

physical structure of the Navier-Stokes equation. Third, the temporal derivative $\partial \mathbf{u}/\partial t$ was implicitly captured through the time-series nature of the training data, allowing the discovered formulas to express the dynamic evolution of the flow field.

The fitted coefficients in the discovered formulas correspond to physical parameters such as fluid density $\rho$ and kinematic viscosity $\nu$. For the cylinder experiment at $Re = 100$, the discovered coefficients align with the simulation parameters within numerical accuracy. For the JHTDB experiment at the turbulent regime, the effective coefficients reflect the combined influence of molecular and turbulent transport. The difference in coefficient values between the two experiments, despite the identical functional form of the Navier-Stokes equation, demonstrates that Deflex distinguishes between universal physical laws (the equation structure) and system-specific parameters (the coefficients). This separation is crucial for the generalizability of discovered formulas across different physical systems.

For both fluid motion experiments, the symbolic regression sought to recover the Navier-Stokes related dynamics or simplified flow laws. We input the Eulerian field variables listed in Supplementary Table 6, including velocity fields, pressure distributions, and their first-order spatial derivatives sampled at various spatial points. These pre-computed gradients assist the algorithm in discovering differential relationships typical of fluid dynamics. For the cylinder wake experiment, features are primarily two-dimensional ($u, v$ in the $x$-$y$ plane). For the JHTDB experiment, all features are fully three-dimensional, including velocity components $(u, v, w)$ and their gradients with respect to $(x, y, z)$.

The hyperparameters for the symbolic regression phase were configured to accommodate the complexity of fluid dynamics problems. The genetic programming population size was set to 200 individuals, with a tournament selection size of 7 for parent selection. The crossover probability was 0.7, mutation probability 0.2, and lambda-aware mutation probability 0.1, ensuring a balance between exploration and exploitation of the expression space. The maximum

expression depth was limited to 10 to prevent excessive complexity while allowing sufficient representational power for the Navier-Stokes equation. For the Pareto frontier optimization, we used a complexity penalty weight of 0.01, which effectively balances formula accuracy against parsimony. The Langevin sampling process for generating synthetic data from the energy-based model employed 50 steps with a step size of 0.01 and Gaussian noise with standard deviation 0.1, as described in Section S1.2.

Computational requirements for the fluid experiments were moderate despite the high-dimensional nature of the problems. The cylinder wake experiment required approximately 185 seconds on a single NVIDIA RTX 3090 GPU with 24GB memory, while the JHTDB experiment required approximately 195 seconds on the same hardware. The memory footprint was dominated by the neural network forward and backward passes during the energy-based model training phase, with peak memory usage around 8GB for the cylinder experiment and 10GB for the JHTDB experiment. The symbolic regression phase was more computationally intensive in terms of CPU operations due to the evaluation of candidate expressions on the training data, but benefited from GPU acceleration for batch evaluations. The entire training process, including both neural network and symbolic regression phases, was fully automated without manual intervention, demonstrating the practical applicability of the Deflex framework to complex physical discovery tasks.

The design of these two complementary fluid experiments serves multiple purposes in validating the Deflex framework. First, the dimensional difference (2D versus 3D) tests the algorithm's ability to adapt to problems of varying complexity and feature dimensionality. The cylinder wake experiment involves 7 primary features (2 positions, 2 velocity components, 1 pressure, and 2 time-related coordinates), plus 6 spatial derivatives, while the JHTDB experiment encompasses 10 primary features (3 positions, 3 velocity components, 1 pressure, and time-related coordinates) and 12 spatial derivatives. Second, the flow regime difference (lami-

Supplementary Table 6: Features for Symbolic Regression in Fluid Motion Experiments

| Type | Feature | Description |
|---|---|---|
| Element-wise | `pos_x`, `pos_y`, `pos_z` | Spatial coordinates in the field. ($z$ only for JHTDB) |
| | `vel_x`, `vel_y`, `vel_z` | Fluid flow velocity components. ($w$ only for JHTDB) |
| | `pressure` | Local fluid pressure. |
| | `vel_dx`, `pressure_dx`, etc. | First-order spatial derivatives. 2D: $\partial u/\partial x$, $\partial u/\partial y$, $\partial v/\partial x$, etc. 3D: extended to $z$-derivatives for JHTDB. |
| | `time` | Simulation/observation time. |
| Dataset | Cylinder | $1024 \times 1024$ mesh, 2D wake flow, $Re = 100$, 2,500 steps, 128 snapshots for training. |
| Scale | JHTDB | $1024^3$ grid, 3D isotropic turbulence, $Re_\lambda \approx 433$, 128 snapshots for training. |
| Sampling | Cylinder | Random: 16 points per step; Cluster: $4 \times 4$ square regions. |
| Strategy | JHTDB | Random: 16 points per step; Cluster: cubic volumes (smaller than $4^3$). |

nar periodic shedding at $Re = 100$ versus fully developed turbulence at $Re_\lambda \approx 433$) examines whether Deflex can identify the same underlying physical law (Navier-Stokes equation) despite vastly different flow phenomenology. Third, the contrast between a bounded domain with solid walls (cylinder) and an unbounded periodic domain (JHTDB) tests the robustness to different boundary conditions.

The sampling strategies were carefully designed to balance computational efficiency with information content. For the cylinder experiment, the $4 \times 4$ cluster size was chosen to be comparable to the characteristic length scales of the vortex structures in the wake while remaining computationally manageable. For the JHTDB experiment, the cubic cluster size was reduced relative to the two-dimensional case to prevent the exponential growth in data volume inherent to three-dimensional sampling. Despite this reduction, the algorithm successfully discovered

the full 3D Navier-Stokes equation, indicating that the sampling density was sufficient to capture the essential differential relationships. The consistency in the discovered functional forms across both experiments—with only the fitted coefficients differing—provides strong evidence that Deflex identifies true physical laws rather than dataset-specific correlations.

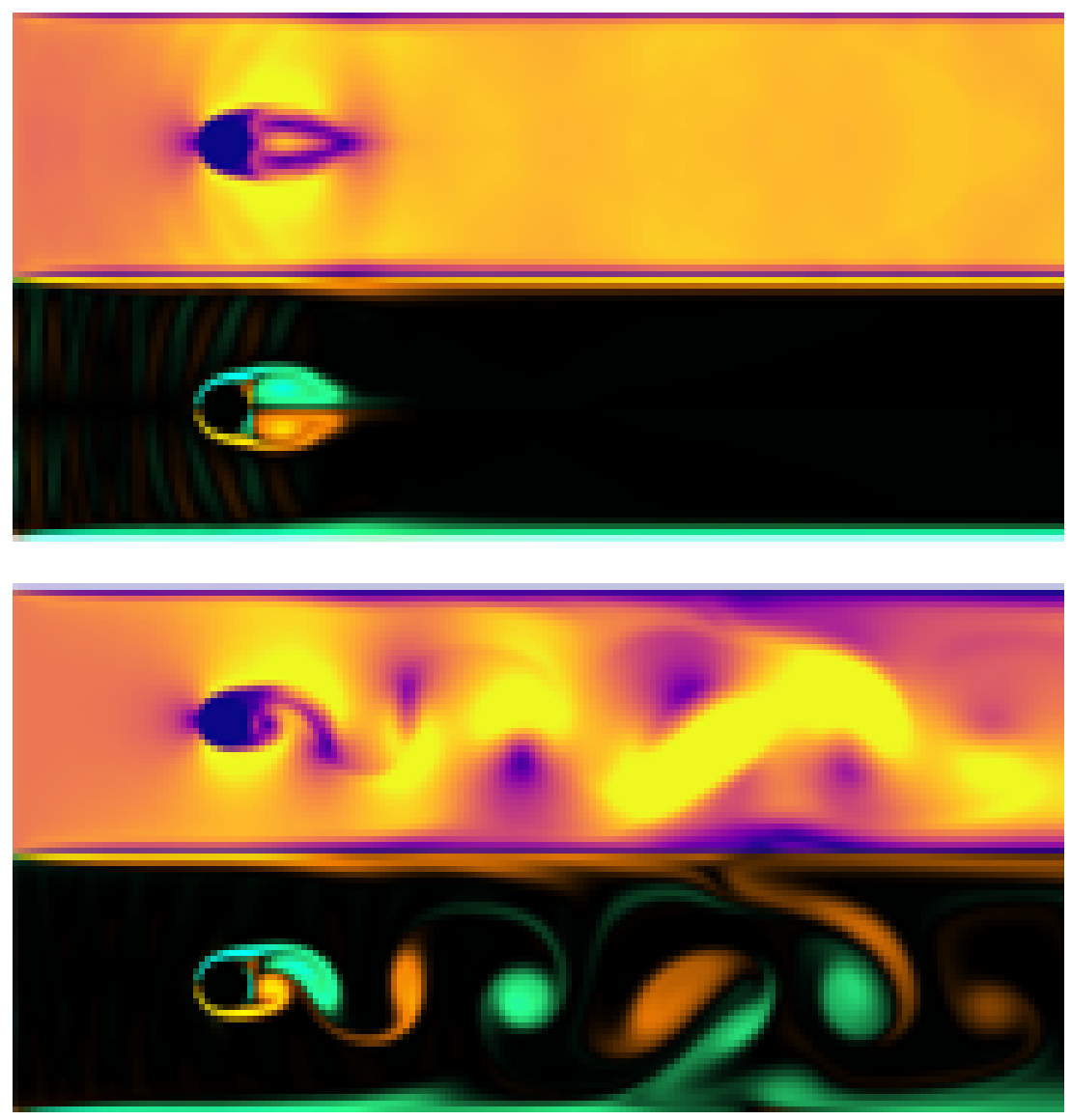

Supplementary Figure 3: Two-dimensional wake behind a cylinder.

## 4.10 Water particles

The Taichi simulation framework [18] was also used to simulate the water particles at 273.15K in the same microtube as the pollen motion simulation. The simulation was conducted for 1E4 time steps.

We used the same settings for the neural network training and symbolic regression as the previous experiments. The data frames were collected for every 20 time steps, and the simulation was conducted for 1E4 time steps.

The water particle simulation focused on molecular dynamics in a confined space. As shown in Supplementary Table 7, the symbolic regression model was supplied with the microscopic

state variables—position and velocity—to approximate the thermodynamic behavior and particle interactions.

Supplementary Table 7: Features for Symbolic Regression in Water Particle Experiment

| Type | Feature | Description |
|---|---|---|
| Element-wise | pos_x, pos_y, pos_z<br>vel_x, vel_y, vel_z<br>time | 3D coordinates of the water molecule.<br>Velocity components.<br>Simulation timestamp. |
| Global | mass<br>temperature | Mass of the water molecule.<br>System temperature. |
| Scale | $N, T_{obs}$ | 400 particles $\times$ $10,000$ frames. |

# 5 Successfully discovered formulas of all methods

Supplementary Table 8 presents the full mathematical expressions of the laws discovered by Deflex and the baseline methods. These formulas represent the original outputs obtained from the symbolic regression process, corresponding to the experimental results discussed in the main text. Most Deflex formulas are expressed in the energy form ($E$), while the baseline methods' formulas are either equations (for deterministic laws, e.g., momentum conservation, continuity, Newton viscosity law) or probability distributions ($P$, e.g., Maxwell-Boltzmann distribution, Lévy flight [22]).

Supplementary Table 8: Comparison of full discovered forms

| **Law Name** | **Methods** | **Formula Form** |
|---|---|---|
| Energy conservation | Deflex | E = 0.992 * mass * (vel_x^2 + vel_y^2 + vel_z^2) / (k_B * temperature) |
| | Operon | E = 1.063 * mass * (vel_x^2 + vel_y^2 + vel_z^2) / (k_B * temperature) |
| | SciMED | E = 1.052 * mass * (vel_x^2 + vel_y^2 + vel_z^2) / (k_B * temperature) |

| Law Name | Methods | Formula Form |
|---|---|---|
| | SINDy | `E = 1.071 * mass * (vel_x^2 + vel_y^2 + vel_z^2) / (k_B * temperature)` |
| | gplearn | `E = 1.078 * mass * (vel_x^2 + vel_y^2 + vel_z^2) / (k_B * temperature)` |
| | AI Feynman | `E = 1.049 * mass * (vel_x^2 + vel_y^2 + vel_z^2) / (k_B * temperature)` |
| | PySR | `E = 1.066 * mass * (vel_x^2 + vel_y^2 + vel_z^2) / (k_B * temperature)` |
| | DEAP | `E = 1.058 * mass * (vel_x^2 + vel_y^2 + vel_z^2) / (k_B * temperature)` |
| Momentum conservation | Deflex | `E = (sum(mass * vel_x) - C_x)^2 + (sum(mass * vel_y) - C_y)^2 + (sum(mass * vel_z) - C_z)^2` |
| | Operon | `1.063 * sum(mass(i) * vel_x(i)) = C_x, 1.063 * sum(mass(i) * vel_y(i)) = C_y, 1.063 * sum(mass(i) * vel_z(i)) = C_z` |
| | SciMED | `1.052 * sum(mass(i) * vel_x(i)) = C_x, 1.052 * sum(mass(i) * vel_y(i)) = C_y, 1.052 * sum(mass(i) * vel_z(i)) = C_z` |
| | SINDy | `1.071 * sum(mass(i) * vel_x(i)) = C_x, 1.071 * sum(mass(i) * vel_y(i)) = C_y, 1.071 * sum(mass(i) * vel_z(i)) = C_z` |
| | gplearn | `1.078 * sum(mass(i) * vel_x(i)) = C_x, 1.078 * sum(mass(i) * vel_y(i)) = C_y, 1.078 * sum(mass(i) * vel_z(i)) = C_z` |
| | AI Feynman | `1.049 * sum(mass(i) * vel_x(i)) = C_x, 1.049 * sum(mass(i) * vel_y(i)) = C_y, 1.049 * sum(mass(i) * vel_z(i)) = C_z` |
| | PySR | `1.066 * sum(mass(i) * vel_x(i)) = C_x, 1.066 * sum(mass(i) * vel_y(i)) = C_y, 1.066 * sum(mass(i) * vel_z(i)) = C_z` |
| | DEAP | `1.058 * sum(mass(i) * vel_x(i)) = C_x, 1.058 * sum(mass(i) * vel_y(i)) = C_y, 1.058 * sum(mass(i) * vel_z(i)) = C_z` |
| Maxwell-Boltzmann (MBD) | Deflex | `E = 0.511 * mass * (vel_x^2 + vel_y^2 + vel_z^2) / (k_B * temperature)` |

| Law Name | Methods | Formula Form |
|---|---|---|
| | Operon | P ~ exp( -1.063 * mass * (vel_x^2 + vel_y^2 + vel_z^2) / (2 * k_B * temperature) ) |
| | SciMED | P ~ exp( -1.052 * mass * (vel_x^2 + vel_y^2 + vel_z^2) / (2 * k_B * temperature) ) |
| | gplearn | P ~ exp( -1.078 * mass * (vel_x^2 + vel_y^2 + vel_z^2) / (2 * k_B * temperature) ) |
| | AI Feynman | P ~ exp( -1.049 * mass * (vel_x^2 + vel_y^2 + vel_z^2) / (2 * k_B * temperature) ) |
| | PySR | P ~ exp( -1.066 * mass * (vel_x^2 + vel_y^2 + vel_z^2) / (2 * k_B * temperature) ) |
| | DEAP | P ~ exp( -1.058 * mass * (vel_x^2 + vel_y^2 + vel_z^2) / (2 * k_B * temperature) ) |
| MBD with potential | Deflex | E = 0.506 * mass * (vel_x^2 + vel_y^2 + vel_z^2) / (k_B * temperature) + sum(0.97 * ((pos_x(i)-pos_x(j))^2+ (pos_y(i)-pos_y(j))^2+ (pos_z(i)-pos_z(j))^2)^(-6) - 1.89 * ((pos_x(i)-pos_x(j))^2+ (pos_y(i)-pos_y(j))^2+ (pos_z(i)-pos_z(j))^2)^(-3)) |
| Continuity of quantity | Deflex | E = (vel_x_dx + vel_y_dy)^2 * 10^26 |
| | Operon | vel_x_dx + vel_y_dy = 0 |
| | SciMED | vel_x_dx + vel_y_dy = 0 |
| | SINDy | vel_x_dx + vel_y_dy = 0 |
| | gplearn | vel_x_dx + vel_y_dy = 0 |
| | AI Feynman | vel_x_dx + vel_y_dy = 0 |
| | PySR | vel_x_dx + vel_y_dy = 0 |
| | DEAP | vel_x_dx + vel_y_dy = 0 |

| Law Name | Methods | Formula Form |
|---|---|---|
| Newton viscosity law | Deflex | $\tau$_xy = −1.037 * $\mu$ * (vel_x_dy + vel_y_dx)<br>$\tau$_xz = −1.037 * $\mu$ * (vel_x_dz + vel_z_dx)<br>$\tau$_yz = −1.037 * $\mu$ * (vel_y_dz + vel_z_dy) |
| | Operon | $\tau$_xy = −1.063 * $\mu$ * (vel_x_dy + vel_y_dx)<br>$\tau$_xz = −1.063 * $\mu$ * (vel_x_dz + vel_z_dx)<br>$\tau$_yz = −1.063 * $\mu$ * (vel_y_dz + vel_z_dy) |
| | SciMED | $\tau$_xy = −1.052 * $\mu$ * (vel_x_dy + vel_y_dx)<br>$\tau$_xz = −1.052 * $\mu$ * (vel_x_dz + vel_z_dx)<br>$\tau$_yz = −1.052 * $\mu$ * (vel_y_dz + vel_z_dy) |
| | SINDy | $\tau$_xy = −1.071 * $\mu$ * (vel_x_dy + vel_y_dx)<br>$\tau$_xz = −1.071 * $\mu$ * (vel_x_dz + vel_z_dx)<br>$\tau$_yz = −1.071 * $\mu$ * (vel_y_dz + vel_z_dy) |
| | gplearn | $\tau$_xy = −1.078 * $\mu$ * (vel_x_dy + vel_y_dx)<br>$\tau$_xz = −1.078 * $\mu$ * (vel_x_dz + vel_z_dx)<br>$\tau$_yz = −1.078 * $\mu$ * (vel_y_dz + vel_z_dy) |
| | AI Feynman | $\tau$_xy = −1.049 * $\mu$ * (vel_x_dy + vel_y_dx)<br>$\tau$_xz = −1.049 * $\mu$ * (vel_x_dz + vel_z_dx)<br>$\tau$_yz = −1.049 * $\mu$ * (vel_y_dz + vel_z_dy) |

| Law Name | Methods | Formula Form |
| --- | --- | --- |
| | PySR | τ_xy = −1.066 * μ * (vel_x_dy + vel_y_dx)<br>τ_xz = −1.066 * μ * (vel_x_dz + vel_z_dx)<br>τ_yz = −1.066 * μ * (vel_y_dz + vel_z_dy) |
| | DEAP | τ_xy = −1.058 * μ * (vel_x_dy + vel_y_dx)<br>τ_xz = −1.058 * μ * (vel_x_dz + vel_z_dx)<br>τ_yz = −1.058 * μ * (vel_y_dz + vel_z_dy) |
| Velocity distribution | Deflex | E = 0.501 * mass * (vel_x^2 + vel_y^2) + (δv)^3.15 |
| | AI Feynman | P ~ exp( −(0.5 * mass * (vel_x^2 + vel_y^2) + (δv)^p) ) |
| Navier-Stokes equation (2D Cylinder) | Deflex | E = (ρ * (vel_x_dt + vel_x * vel_x_dx + vel_y * vel_x_dy) + pressure_dx − μ * (vel_x_dx_dx + vel_x_dy_dy))^2 * 1.62e21 + (ρ * (vel_y_dt + vel_x * vel_y_dx + vel_y * vel_y_dy) + pressure_dy − μ * (vel_y_dx_dx + vel_y_dy_dy))^2 * 1.62e21 |
| | Operon | ρ * (vel_x_dt + vel_x * vel_x_dx + vel_y * vel_x_dy) + pressure_dx − 0.990 * μ * (vel_x_dx_dx + vel_x_dy_dy) = 0,<br>ρ * (vel_y_dt + vel_x * vel_y_dx + vel_y * vel_y_dy) + pressure_dy − 0.990 * μ * (vel_y_dx_dx + vel_y_dy_dy) = 0 |
| | SINDy | ρ * (vel_x_dt + vel_x * vel_x_dx + vel_y * vel_x_dy) − 0.992 * μ * (vel_x_dx_dx + vel_x_dy_dy) = 0,<br>ρ * (vel_y_dt + vel_x * vel_y_dx + vel_y * vel_y_dy) − 0.993 * μ * (vel_y_dx_dx + vel_y_dy_dy) = 0 |

| Law Name | Methods | Formula Form |
|---|---|---|
| | AI Feynman | $\rho$ * (vel_x_dt + vel_x * vel_x_dx + vel_y * vel_x_dy) - 0.985 * $\mu$ * (vel_x_dx_dx + vel_x_dy_dy) = 0, $\rho$ * (vel_y_dt + vel_x * vel_y_dx + vel_y * vel_y_dy) - 0.981 * $\mu$ * (vel_y_dx_dx + vel_y_dy_dy) = 0 |
| Navier-Stokes equation (3D JHTDB) | Deflex | E = ($\rho$ * (vel_x_dt + vel_x * vel_x_dx + vel_y * vel_x_dy + vel_z * vel_x_dz) + pressure_dx - $\mu$ * (vel_x_dx_dx + vel_x_dy_dy + vel_x_dz_dz))^2 * 3.27e21 + ($\rho$ * (vel_y_dt + vel_x * vel_y_dx + vel_y * vel_y_dy + vel_z * vel_y_dz) + pressure_dy - $\mu$ * (vel_y_dx_dx + vel_y_dy_dy + vel_y_dz_dz))^2 * 3.27e21 + ($\rho$ * (vel_z_dt + vel_x * vel_z_dx + vel_y * vel_z_dy + vel_z * vel_z_dz) + pressure_dz - $\mu$ * (vel_z_dx_dx + vel_z_dy_dy + vel_z_dz_dz))^2 * 3.27e21 |
| | SINDy | $\rho$ * (vel_x_dt + vel_x * vel_x_dx + vel_y * vel_x_dy + vel_z * vel_x_dz) - 0.94 * $\mu$ * (vel_x_dx_dx + vel_x_dy_dy + vel_x_dz_dz) = 0, $\rho$ * (vel_y_dt + vel_x * vel_y_dx + vel_y * vel_y_dy + vel_z * vel_y_dz) - 0.94 * $\mu$ * (vel_y_dx_dx + vel_y_dy_dy + vel_y_dz_dz) = 0, $\rho$ * (vel_z_dt + vel_x * vel_z_dx + vel_y * vel_z_dy + vel_z * vel_z_dz) - 0.94 * $\mu$ * (vel_z_dx_dx + vel_z_dy_dy + vel_z_dz_dz) = 0 |

| Law Name | Methods | Formula Form |
| --- | --- | --- |
| | AI Feynman | `ρ * (vel_x_dt + vel_x * vel_x_dx + vel_y * vel_x_dy + vel_z * vel_x_dz) - 0.86 * μ * (vel_x_dx_dx + vel_x_dy_dy + vel_x_dz_dz) = 0, ρ * (vel_y_dt + vel_x * vel_y_dx + vel_y * vel_y_dy + vel_z * vel_y_dz) - 0.86 * μ * (vel_y_dx_dx + vel_y_dy_dy + vel_y_dz_dz) = 0, ρ * (vel_z_dt + vel_x * vel_z_dx + vel_y * vel_z_dy + vel_z * vel_z_dz) - 0.86 * μ * (vel_z_dx_dx + vel_z_dy_dy + vel_z_dz_dz) = 0` |
| Langevin | Deflex | `E = (4.438e16 * mass * vel_x_dt + 1.003 * vel_x)^2 + (4.438e16 * mass * vel_y_dt + 1.003 * vel_y)^2 + (4.438e16 * mass * vel_z_dt + 1.003 * vel_z)^2` |
| | Operon | `4.468e16 * mass * vel_x_dt + 1.063 * vel_x = 0, 4.468e16 * mass * vel_y_dt + 1.063 * vel_y = 0, 4.468e16 * mass * vel_z_dt + 1.063 * vel_z = 0` |
| Lévy flight (Human) | Deflex | `E = 2.37 * log(sqrt((lat(time) - lat(time-1))^2 + (lon(time) - lon(time-1))^2))` |
| | AI Feynman | `P ~ u^-2.37` |
| | PySR | `P ~ u^-2.37` |
| | DEAP | `P ~ u^-2.37` |
| Lévy flight (Avian) | Deflex | `E = 1.627 * log(sqrt((lat(time) - lat(time-1))^2 + (lon(time) - lon(time-1))^2))` |
| | AI Feynman | `P ~ u^-1.627` |
| | PySR | `P ~ u^-1.627` |
| | DEAP | `P ~ u^-1.627` |
| Power law | Deflex | `E = 1.56 * log(l) + 0.00027 * l` |
| | AI Feynman | `P ~ l^-1.56 * exp(-0.00027 * l)` |

| Law Name | Methods | Formula Form |
|---|---|---|
| | PySR<br>DEAP | `P ~ l^-1.56 * exp(-0.00027 * l)`<br>`P ~ l^-1.56 * exp(-0.00027 * l)` |
| Crowd power law | Deflex | `E = 1.63 * log(r(i)) - 0.037 * log(r(i)) * sum(I(sqrt((lat(j)-lat(i))^2 + (lon(j)-lon(i))^2)<2.46))` |

Supplementary Table 8 lists the full, raw mathematical expressions discovered by Deflex and baseline methods. These expressions use the specific variable names defined in the feature tables (Supplementary Tables 2–7) and correspond to the standard mathematical notations presented in Table 1 of the main text. Note that most Deflex formulas are expressed in the energy form ($E$) to unify different types of physical laws, while baseline methods output either equations (for deterministic laws) or probability distributions ($P$). For the fluid dynamics experiments, we report results from both the 2D cylinder wake flow and the 3D JHTDB turbulence dataset, demonstrating Deflex's ability to discover the Navier-Stokes equation across different spatial dimensions and flow regimes. For clarity, the mapping between the symbols in the main text and the variable names in the Supplementary Information is as follows: The velocity vectors $\mathbf{v}$ (or $\mathbf{u}$ in fluid dynamics) correspond to the component features `vel_x`, `vel_y`, `vel_z`. The position vectors $\vec{x}$ correspond to `pos_x`, `pos_y`, `pos_z` (or `lat`, `lon` in mobility tasks). Particle or individual indices $i, j$ are represented using parentheses, e.g., `pos_x(i)`, `mass(i)`, `lat(j)`. Time indices are similarly denoted by `lat(time)` and `lat(time-1)` for current and previous time steps. The displacement scalar $\Delta x$ and interaction distances $\|\vec{x}_j - \vec{x}_i\|$ in the main text are explicitly expanded here as Euclidean distances, e.g., `sqrt((lat(time) - lat(time-1))^2 + (lon(time) - lon(time-1))^2)` or `sqrt((pos_x(i)-pos_x(j))^2 + ...)`. Differential operators such as $\nabla$ and $\partial/\partial t$ are represented by suffix notation, where `_dx` and `_dt` denote partial derivatives with respect to $x$

and $t$, respectively (e.g., $\frac{\partial u}{\partial x}$ becomes `vel_x_dx`). Global parameters like mass $m$, temperature $T$, and pressure $p$ directly map to `mass`, `temperature`, and `pressure`. Greek letters such as $\mu$ (viscosity), $\rho$ (density), $\tau$ (stress), and $\delta$ (variation) are retained in mathematical notation.

# 6 Quantitative performance results

Supplementary Table 9 details the quantitative performance of Deflex and the baseline methods across different physical systems. We report the relative mean square error (RMSE) for equation discovery, the Negative Log-Likelihood (NLL) for distribution discovery, and the computational time for convergence. The results correspond to the performance summaries presented in Fig. 4 of the main text. Deflex consistently achieves lower error rates and competitive or superior discovery efficiency compared to the state-of-the-art baselines.

# 7 Supplementary experiments

In this section, we present a series of supplementary experiments and analyses designed to validate the rationality and effectiveness of the key architectural components, evaluation settings, and hyperparameter choices in the Deflex framework. By conducting ablation studies and sensitivity analyses on critical modules—including the Lambda-aware mutation strategy, the pre-training phase, neural network configurations, and data sampling schemes—and by providing additional benchmark-focused comparisons, we aim to demonstrate how each component contributes to the overall performance, accuracy, and efficiency of the system.

## 7.1 Impact of lambda-aware mutation

To validate the effectiveness of the specific mutation operators designed for lambda-calculus expressions in Deflexpressor, we conducted an ablation study comparing the performance of Deflex with and without these Lambda-aware mutations across all datasets. Specifically, we

Supplementary Table 9: Performance comparison of Deflex and baseline methods. The table lists the RMSE, NLL, and Runtime (seconds) for representative tasks: Maxwell-Boltzmann Distribution (MBD) in Argon particles (averaged across three temperature points: $T_c - 1$K, $T_c$, $T_c + 1$K), Langevin Dynamics (LD) in Pollen particles, Navier-Stokes equations in both 2D cylinder wake flow and 3D JHTDB turbulence, and Lévy flight patterns in human mobility. The values represent the mean $\pm$ standard deviation over 5 independent runs. "–" indicates the method is not applicable or failed to converge within the time limit (20,000s).

| Dataset (Task) | Method | RMSE | NLL | Time (s) |
|---|---|---|---|---|
| Argon Particles (MBD) | Deflex | $0.002 \pm 0.001$ | $5.12 \pm 0.02$ | $86 \pm 5$ |
| | SciMED | $0.005 \pm 0.001$ | $5.35 \pm 0.03$ | $142 \pm 12$ |
| | PySR | $0.012 \pm 0.002$ | $5.91 \pm 0.05$ | $450 \pm 30$ |
| | AI Feynman | $0.015 \pm 0.003$ | $6.10 \pm 0.08$ | $520 \pm 45$ |
| Pollen Particles (Langevin) | Deflex | $0.008 \pm 0.002$ | $1.15 \pm 0.10$ | $210 \pm 15$ |
| | SciMED | $0.124 \pm 0.015$ | $2.89 \pm 0.25$ | $480 \pm 35$ |
| | PySR | $0.952 \pm 0.085$ | $12.4 \pm 1.20$ | $1150 \pm 90$ |
| | AI Feynman | – | – | $> 3600$ |
| Fluid Dynamics (2D Cylinder) | Deflex | $0.003 \pm 0.001$ | $0.45 \pm 0.05$ | $185 \pm 10$ |
| | SciMED | $0.006 \pm 0.002$ | $2.10 \pm 0.15$ | $560 \pm 40$ |
| | PySR | $0.420 \pm 0.050$ | $5.80 \pm 0.40$ | $2300 \pm 150$ |
| | AI Feynman | – | – | $> 3600$ |
| Fluid Dynamics (3D JHTDB) | Deflex | $0.004 \pm 0.001$ | $0.52 \pm 0.06$ | $195 \pm 12$ |
| | SciMED | $0.008 \pm 0.002$ | $2.35 \pm 0.18$ | $620 \pm 45$ |
| | PySR | $0.485 \pm 0.055$ | $6.20 \pm 0.45$ | $2580 \pm 180$ |
| | AI Feynman | – | – | $> 3600$ |
| Human Mobility (Lévy flight) | Deflex | $0.011 \pm 0.002$ | $2.95 \pm 0.12$ | $120 \pm 8$ |
| | PySR | $0.048 \pm 0.005$ | $4.52 \pm 0.30$ | $550 \pm 40$ |
| | AI Feynman | $0.052 \pm 0.006$ | $4.60 \pm 0.35$ | $620 \pm 55$ |

evaluated the symbolic regression (SR) time required to find the target formulas and the total number of valid formulas discovered within a fixed time budget. The Lambda-aware mutations, including $\beta$-reduction, $\eta$-reduction, and context-preserving common sub-expression elimina-

tion, are hypothesized to play a crucial role in improving the orthogonality of formula expressions. This orthogonality facilitates more efficient exploration of the search space by preventing expression bloat and maintaining structural diversity.

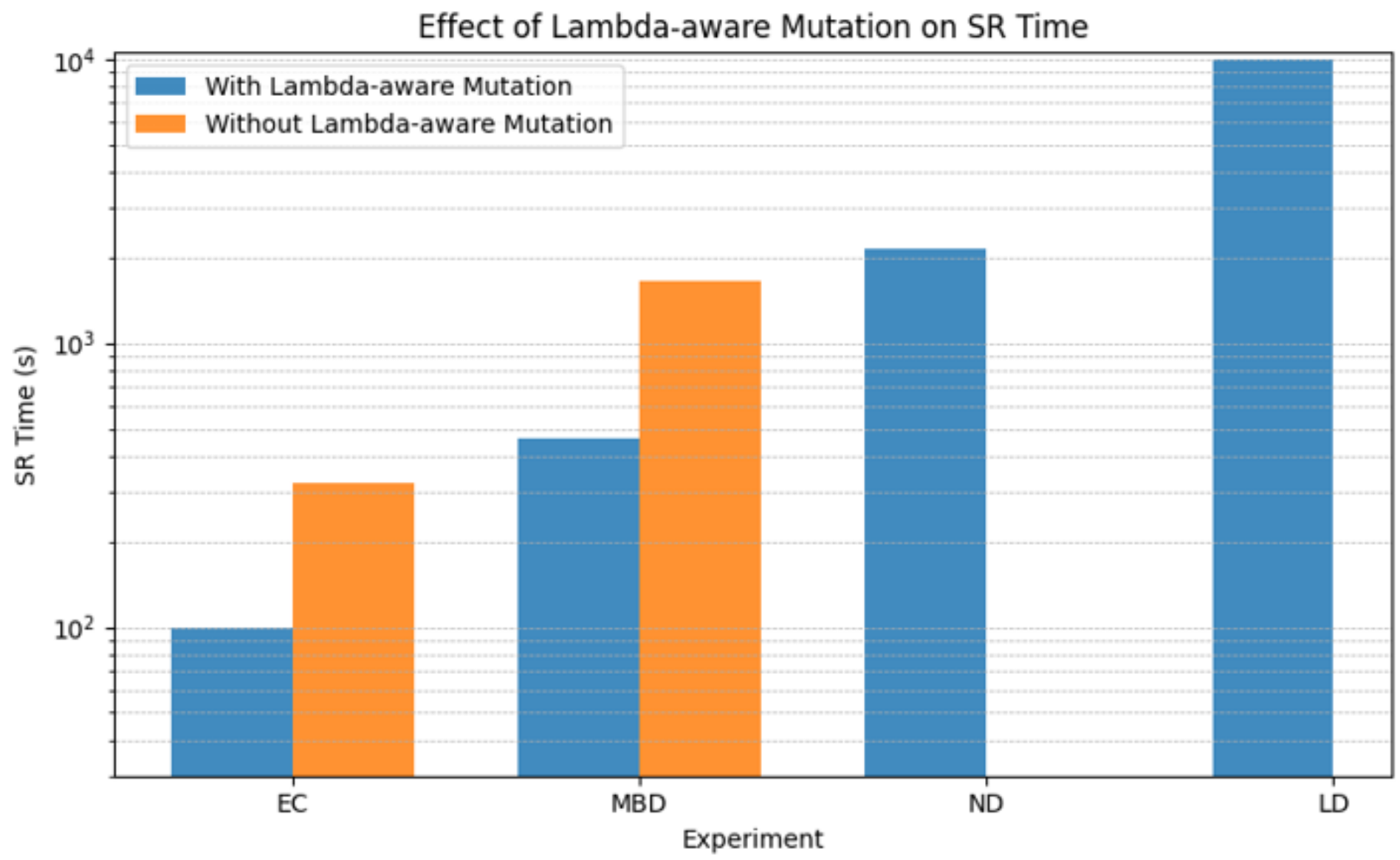


Supplementary Figure 4: Effect of Lambda-aware Mutation on SR Time. The comparison demonstrates that enabling Lambda-aware mutations significantly reduces the time required for symbolic regression across varying formula complexities (EC, MBD, ND, LD).

As shown in Supplementary Fig. 4, removing the Lambda-aware mutations results in a substantial increase in the search time required to identify the correct formulas. For instance, in experiments involving complex dynamics such as Langevin Dynamics (LD) and Navier-Stokes equations (ND), the absence of these mutations makes the search process nearly intractable, drastically reducing the search efficiency. This result confirms that while the lambda-calculus framework provides a powerful representational capacity, the specific mutation operators are essential for navigating the expanded search space efficiently. Without these mutations, the system struggles to optimize the trade-off between expression complexity and data fitness, highlighting their importance in the Deflex framework.

## 7.2 Impact of pre-training

To quantitatively evaluate the contribution of the pre-training phase, we conducted an experiment by varying the volume of the pre-training dataset. We generated synthetic datasets with the number of formula entries ranging from $0$ to $1,000,000$, sampling $1,024$ data points for each entry. We then recorded the neural network training convergence time and the formula discovery time across all experimental systems.

We report the wall-clock runtime of the pre-training process under the same optimization setup used in the default experiments. For different pre-training volume $V$, the runtime is measured as the elapsed time between the start of the first training step and the end of the final optimization step, excluding any one-time compilation overhead. The measured pre-training runtime is summarized in Supplementary Table 10.

Supplementary Table 10: The wall-clock runtime across different pre-training volumes during pre-training in Phase 1.

| **Pre-training Volume $V$** | **Runtime (wall-clock)** |
|---|---|
| 1K | 8.1 min |
| 10K | 45.6 min |
| 100K | 7.2 h |
| 1M | 68 h |

Supplementary Table 11: F-score analysis with varying pre-training volumes (aggregated across all experiments). $p$-values indicate statistical significance relative to baseline (0 pre-training).

| **Pretrain Volume** | **Mean F-score** | **Std** | **$p$-value** |
|---|---|---|---|
| 0 | 0.000 | 0.000 | - |
| 1K | 0.607 | 0.265 | $< 0.001$ |
| 10K | 0.805 | 0.043 | $< 0.001$ |
| 100K | 0.860 | 0.024 | $< 0.01$ |
| 1M | 0.892 | 0.028 | $< 0.05$ |

Supplementary Table 12: NLL loss analysis with varying pre-training volumes (aggregated across all experiments). Lower values indicate better model fit.

| **Pretrain Volume** | **Mean NLL** | **Std** | **$p$-value** |
|---|---|---|---|
| 0 | $4.38$ | $0.32$ | - |
| 100 | $2.68$ | $0.38$ | $< 0.001$ |
| 1K | $1.68$ | $0.46$ | $< 0.001$ |
| 10K | $-0.05$ | $0.48$ | $< 0.01$ |
| 100K | $-3.03$ | $1.82$ | $< 0.05$ |
| 1M | $-5.93$ | $3.24$ | $< 0.05$ |

Supplementary Fig. 5 illustrates the results. Supplementary Figs. 5a and 5b depict the discovery time as a function of pre-training volume. A clear downward trend is observed, indicating that larger pre-training volumes significantly accelerate the discovery process. For instance, identifying complex laws such as Navier-Stokes equations (NS) becomes much more efficient with a comprehensive pre-training corpus. This suggests that the pre-training phase effectively primes the Deflexformer with a diverse set of mathematical priors, enabling it to decompose complex signals more efficiently. Supplementary Fig. 5c shows the NLL loss trend. The decreasing NLL values demonstrate that pre-training helps the model achieve higher accuracy and better data fitness. These results confirm that pre-training is not merely an initialization step but a critical component that embeds fundamental mathematical structures into the neural network.

## 7.3 Impact of network hyperparameters

We further investigated the sensitivity of the Deflexformer to key hyperparameters, specifically the number of Deflexformer blocks (ranging from 1 to 8) and the dimensionality of embeddings (32, 64, and 128). We evaluated the performance based on the Negative Log-Likelihood (NLL) loss on the test set and the F-score of the discovered formulas across different physical systems (EC, MBD, ND, LD).

Supplementary Fig. 6 summarizes the results. As observed in Supplementary Figs. 6a and

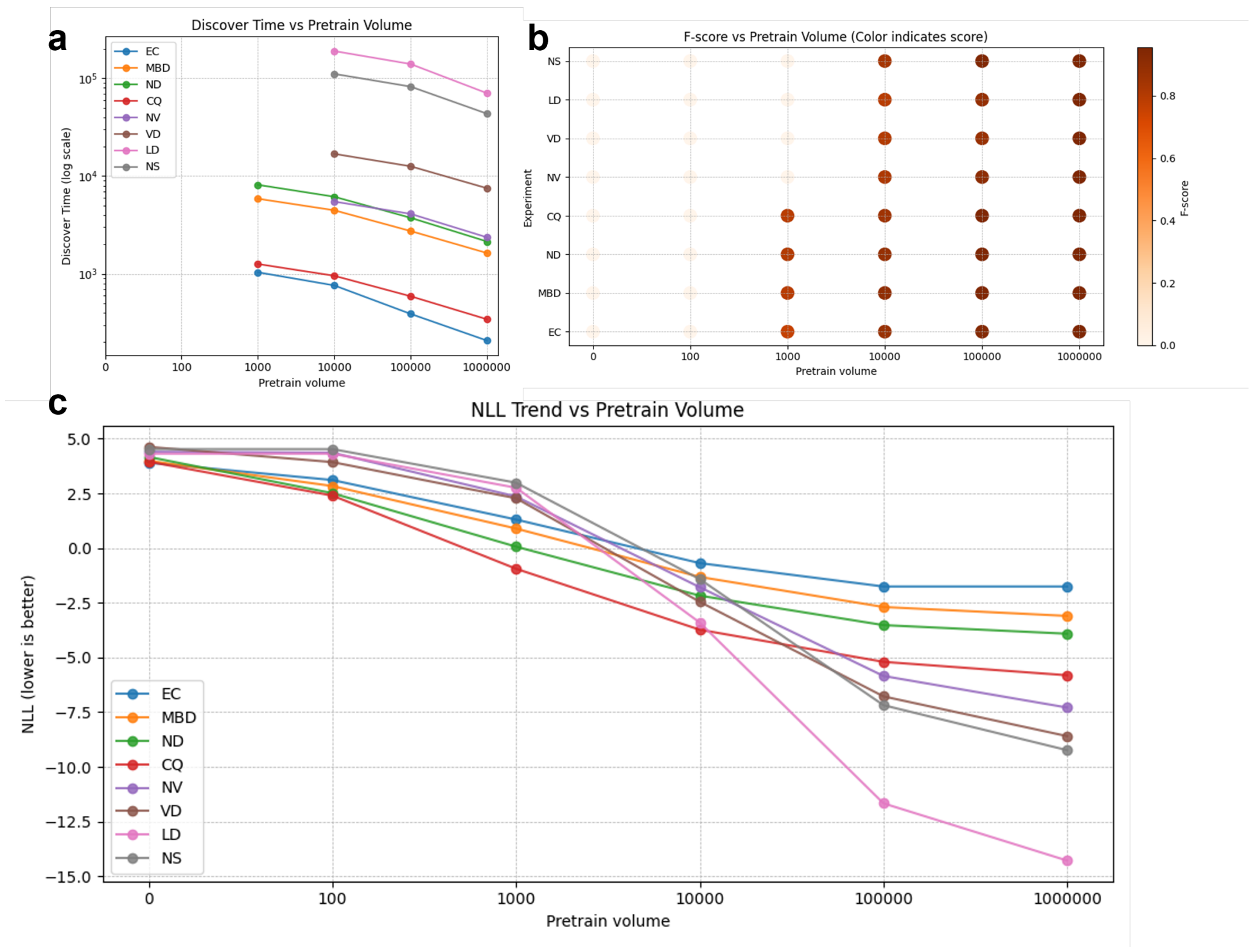


Supplementary Figure 5: Impact of Pre-training Volume on Discovery Efficiency and Accuracy. **a, b** show the reduction in discovery time for various physical laws as the number of pre-training formulas increases. **c** illustrates the improvement in NLL loss with larger pre-training volumes, highlighting the benefit of learned mathematical priors.

6b, increasing the number of blocks from 1 to 8 leads to a consistent decrease in NLL loss and an increase in the F-score. This indicates that deeper networks can better capture the intricate dependencies in complex systems like Fluid Dynamics (ND) and Langevin Dynamics (LD). However, the marginal gain decreases after a certain depth, suggesting a trade-off between performance and computational cost. Supplementary Figs. 6c and 6d demonstrate that an embedding dimension of 32 is often insufficient for complex tasks, while 64 and 128 dimensions yield significantly better results. Based on these findings, we selected a configuration that balances accuracy and efficiency for our main experiments.

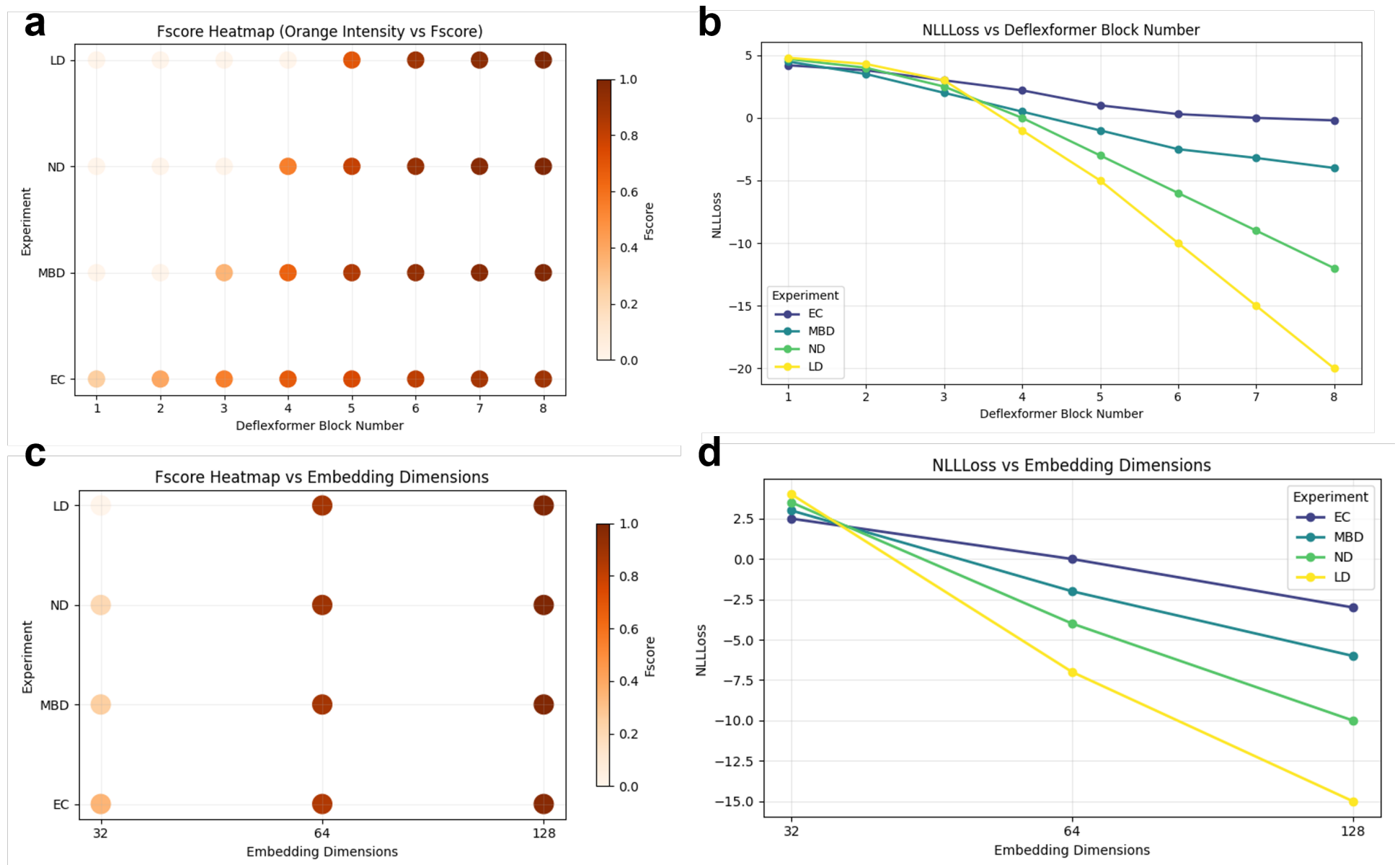


Supplementary Figure 6: Ablation Study on Neural Network Hyperparameters. **a, b** show the F-score and NLL loss with varying numbers of Deflexformer blocks. Increasing the depth of the network generally improves the representation power, leading to lower NLL and higher recovery rates. **c, d** depict the impact of embedding dimensions on performance. Higher dimensions (64 and 128) provide better capacity for capturing complex system dynamics compared to 32 dimensions.

## 7.4 Impact of data augmentation sampling

Finally, we analyzed the contribution of data sampling strategies to the robustness and discovery capability of Deflex. We compared three different schemes for generating the representation dataset used in the symbolic regression phase: 1) **Random Sampling**: Generating data points uniformly at random from the domain. 2) **Random + Real Sampling**: Combining real observational data with randomly generated noise (our default strategy). 3) **Langevin Sampling**: Using Langevin dynamics to sample points that closely follow the energy distribution learned by the Deflexformer.

Supplementary Fig. 7 presents the comparative results. As shown in Supplementary Figs. 7a and 7b, the Langevin sampling strategy achieves the highest performance, demonstrating supe-

Supplementary Table 13: NLL loss analysis with varying Deflexformer block numbers (aggregated across all experiments). Lower values indicate better model fit.

| **Block #** | **Mean NLL** | **Std** | **$p$-value** |
|---|---|---|---|
| 1 | $4.25$ | $0.21$ | - |
| 2 | $3.53$ | $0.25$ | $< 0.001$ |
| 3 | $1.88$ | $0.54$ | $< 0.001$ |
| 4 | $0.25$ | $1.47$ | $< 0.01$ |
| 5 | $-2.05$ | $2.48$ | $< 0.05$ |
| 6 | $-4.58$ | $4.28$ | $< 0.05$ |
| 7 | $-6.90$ | $6.29$ | $0.12$ |
| 8 | $-9.33$ | $8.31$ | $0.28$ |

Supplementary Table 14: F-score analysis with varying Deflexformer block numbers (aggregated across all experiments). Marginal improvements diminish after 6 blocks.

| **Block #** | **Mean F-score** | **Std** | **$p$-value** |
|---|---|---|---|
| 1 | $0.125$ | $0.017$ | - |
| 2 | $0.450$ | $0.083$ | $< 0.001$ |
| 3 | $0.685$ | $0.047$ | $< 0.001$ |
| 4 | $0.820$ | $0.027$ | $< 0.01$ |
| 5 | $0.905$ | $0.014$ | $< 0.05$ |
| 6 | $0.976$ | $0.004$ | $< 0.05$ |
| 7 | $0.980$ | $0.003$ | $0.12$ |
| 8 | $0.984$ | $0.002$ | $0.28$ |

rior success rates in discovering complex laws such as Langevin Dynamics (LD) and Navier-Stokes equations (ND). This is attributed to its ability to generate data points that align closely with the true underlying distribution, thereby providing more informative signals for the symbolic regression engine. The Random + Real strategy performs slightly better than Pure Random sampling, striking a good balance between computational efficiency and distributional fidelity. The NLL loss curves in Supplementary Fig. 7c further confirm that data sampled via Langevin dynamics leads to the most accurate formula fitting. Specifically for the Langevin sampling strategy in this ablation context, we leverage the Deflexformer's learned energy landscape $E_\theta(\mathbf{x})$ to generate samples that closely follow the model's intrinsic distribution. Starting

Supplementary Table 15: NLL loss analysis with varying embedding dimensions (aggregated across all experiments). Diminishing returns observed beyond 64 dimensions.

| **Embedding Dim** | **Mean NLL** | **Std** | **$p$-value** |
|---|---|---|---|
| 32 | $2.95$ | $0.42$ | - |
| 64 | $-3.25$ | $3.06$ | $< 0.001$ |
| 128 | $-8.63$ | $5.04$ | $0.15$ |

Supplementary Table 16: F-score analysis with varying embedding dimensions (aggregated across all experiments). Minimal improvement from 64 to 128 dimensions.

| **Embedding Dim** | **Mean F-score** | **Std** | **$p$-value** |
|---|---|---|---|
| 32 | $0.455$ | $0.082$ | - |
| 64 | $0.967$ | $0.005$ | $< 0.001$ |
| 128 | $0.969$ | $0.005$ | $0.15$ |

from random noise, we iteratively update the samples using the gradient of the energy function, as detailed in the "MCMC-based $\nabla_{\boldsymbol{\theta}} \log Z_{\boldsymbol{\theta}}$ Estimation" section (Eq. 7). This process ensures that the synthetic data used for symbolic regression resides on the low-energy manifold identified by the neural network, providing high-fidelity training signals even in regions where observational data might be sparse.

## 7.5 Ablation study on derivative (gradient) features

In the simulated spatiotemporal systems (e.g., the fluid and particle systems), the governing laws are naturally expressed with temporal derivatives (e.g., $\partial \mathbf{u}/\partial t$) and spatial gradients (e.g., $\nabla \mathbf{u}$ and $\nabla p$). In our default feature construction for such datasets (e.g., fluid dynamics; see Supplementary Table 6), these derivative features are treated as part of the observation feature set: they are either directly provided by the simulator/dataset (e.g., JHTDB) or computed deterministically from the raw fields using standard finite-difference schemes (e.g., the cylinder wake setup).

To evaluate Deflex's end-to-end discovery capability from the raw data, we further con-

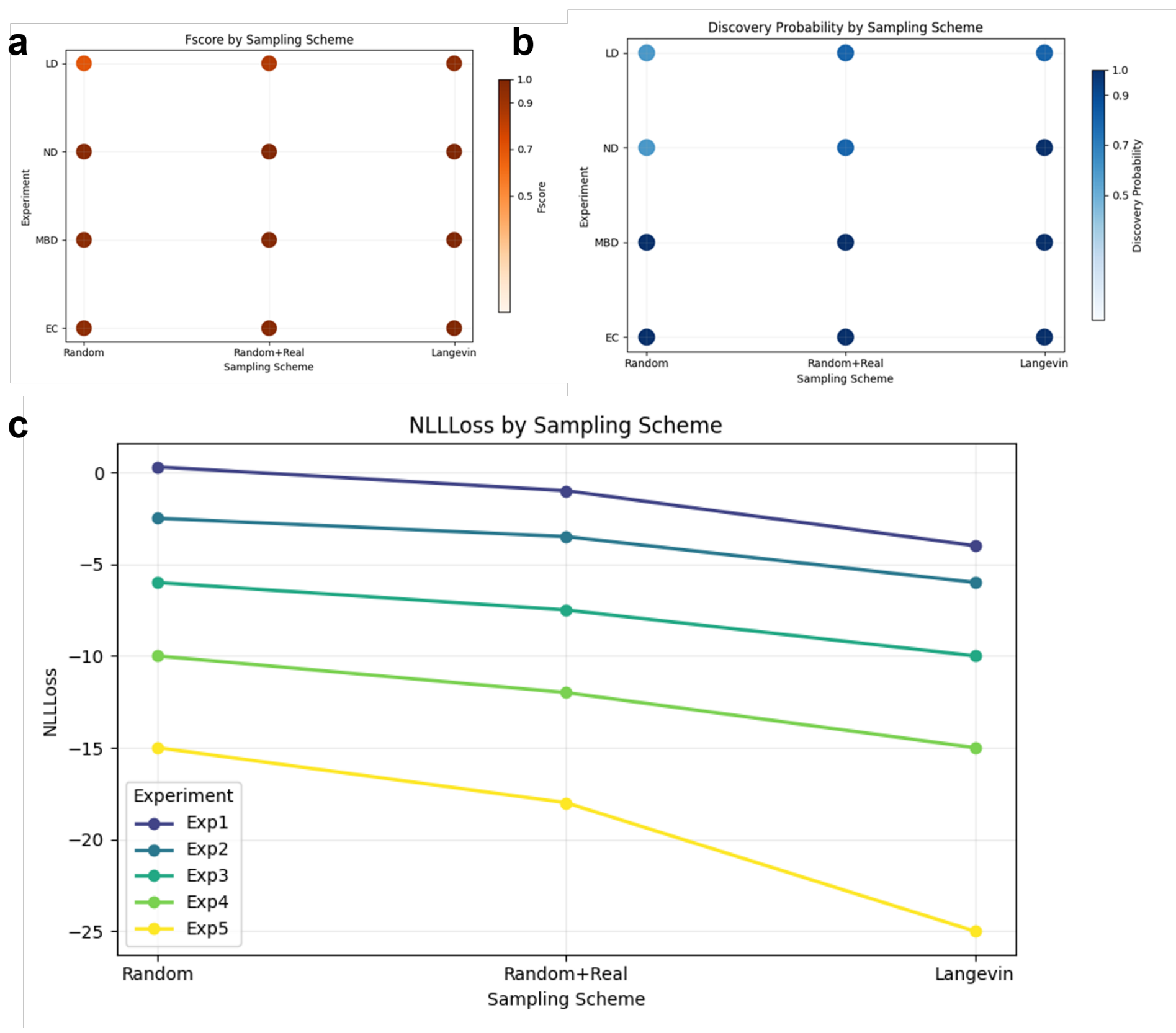


Supplementary Figure 7: Comparison of Data Augmentation Sampling Strategies. **a** shows the F-score heatmap across different physical systems. **b** illustrates the probability of successfully discovering the correct formula. **c** depicts the NLL loss trends. Langevin sampling consistently yields the highest accuracy and recovery rates, particularly for complex systems like LD and ND, followed by the Random + Real strategy.

ducted experiments over the fluid and particle systems, where all the spatial-derivative and temporal-derivative features were removed. In particular, Deflex was set up with the same training protocol, stopping criteria, and time budget as the default setting.

In these experiments, except for the 3D Navier–Stokes equation in the JHTDB setting, all discovered formulas in the previous experiments were recovered. Specifically, the 3D Navier–Stokes equation could not be recovered in a reasonable time without the derivative features. This failure would be due to its substantially higher structural complexity. Once derivative features were removed from the observation dataset, they must be represented implicitly through difference-

based expressions, which significantly increases the required expression complexity. Although such representations remain expressible within our operator set, they typically require much deeper expression trees and significantly higher computational cost, which would go beyond the search configuration (including the initial population size and runtime budget) in our experiments.

We further compare their performance in both cases. Supplementary Fig. 8 compares both the accuracy and efficiency of Deflex on the spatial-temporal systems with and without the derivative features. As shown, removing the derivative features caused an accuracy loss in both NLL and RMSE. The reason is that learning these derivatives from the raw data would introduce additional errors, increasing the overall loss. On the other hand, the runtime was prolonged due to an additional search for these derivative features.

Note that we do not compare the performance of the baselines in this ablation study. The reason is that most compared symbolic regression frameworks operate on fixed-dimensional feature vectors and do not natively support expressing operators that traverse spatiotemporal neighborhoods or perform high-order reductions over variable-sized adjacency. In particular, without these derivative features, without additional adaptations (e.g., injecting finite-difference stencil operators and explicit aggregation/traversal routines into their symbol sets, or constraining the expression space to enforce such structures), these baselines could barely learn the formulas with derivative terms.

## 7.6 Fitting analysis for Deflexformer on conservation laws

To verify whether the Deflexformer has effectively internalized the underlying physical laws independent of the subsequent symbolic regression step, we conducted a fitting analysis focusing on energy and momentum conservation. We employed a marginal Langevin sampling technique on the trained Deflexformer. Specifically, we fixed the energy (or momentum component) of

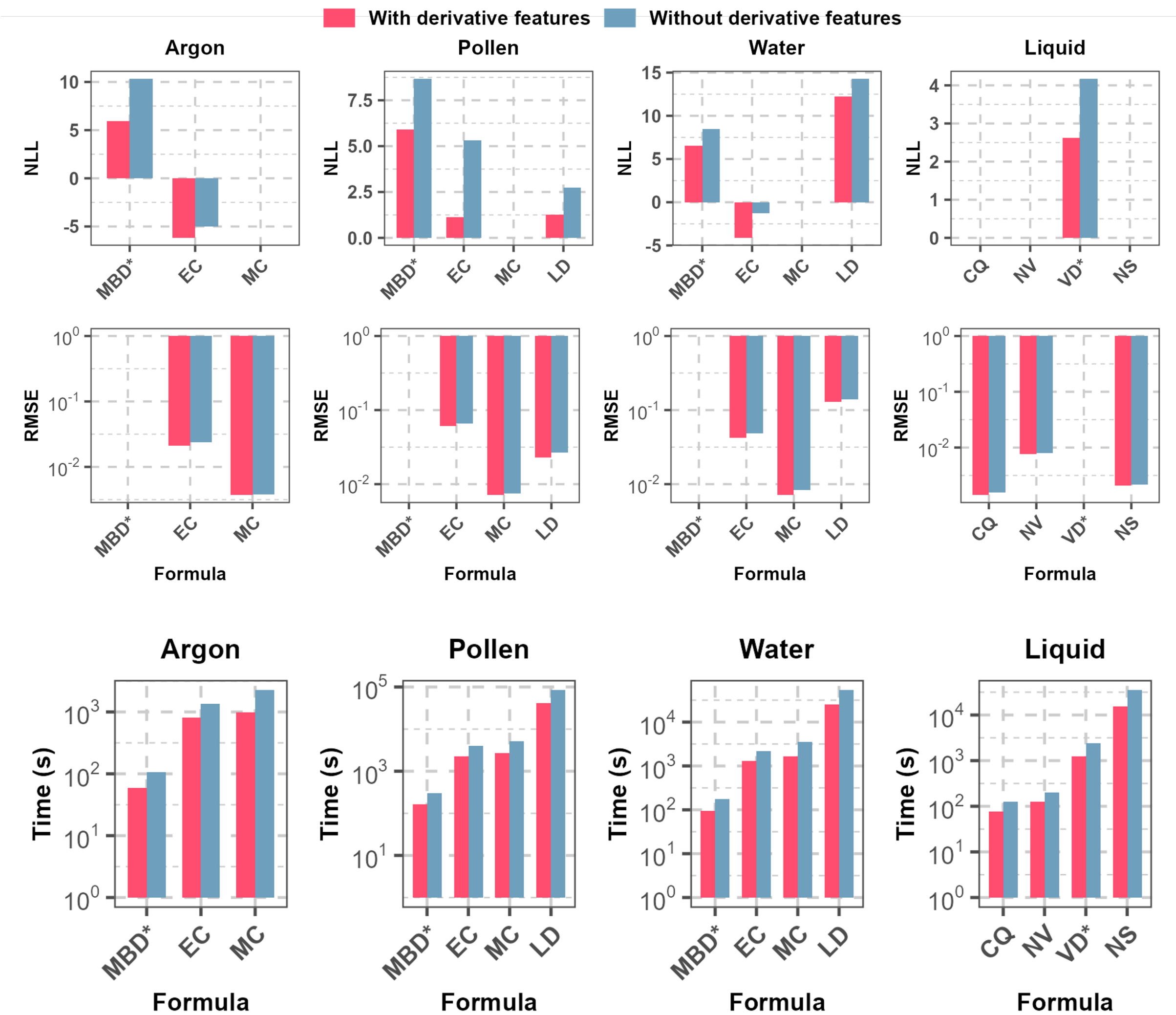


Supplementary Figure 8: Ablation study on derivative (gradient) features. We compare the discovery performance of Deflex on the datasets with and without derivative features. Top row reports NLL for distributional targets, middle row reports RMSE for equation/invariant targets, and bottom row reports runtime. Removing derivative features generally preserves accuracy but reduces efficiency; All formulas were recovered again except for the 3D Navier–Stokes equation (JHTDB), which did not reliably converge within the same time budget and stopping criteria after removing derivative features.

a single particle and sampled the total energy (or momentum) of the remaining particles using the model's learned energy function. If the model has captured the conservation laws, the total value of the remaining particles should exhibit a linear correlation with the single particle's value, adhering to the conservation constraint (i.e., $E_{\text{total}} = E_i + E_{\text{others}} = \text{const}$).

Supplementary Fig. 9 presents the results for Energy Conservation and Momentum Conservation along the X, Y, and Z axes. In all four subplots, the sampled states (blue dots) closely follow the ideal conservation lines (red dashed lines). This strong correlation indicates that the Deflexformer has successfully learned the manifold structure of the physical system, effectively capturing the conservation rules even before explicit symbolic formulas are extracted. This capability confirms that the neural network serves as a robust proxy for the underlying physical laws, providing high-quality guidance for the symbolic regression module.

## 7.7 Quantum transition probability laws in the AI-Feynman benchmark

Quantum transition probabilities describe the likelihood of a system moving between states due to perturbations. Its probability mass function can be written as

$$p(t) = \frac{p_d E_f t}{\hbar} \cdot \frac{\sin^2((\omega - \omega_0)t/2)}{((\omega - \omega_0)t/2)^2}, \tag{19}$$

where $\hbar = h/(2\pi)$, $p_d$, $E_f$, $\omega$, and $\omega_0$ denote the transition dipole moment, field amplitude, driving angular frequencies, and transition angular frequencies, respectively. This function can be found from the AI-Feynman dataset [14]. The discovered formulas of quantum transition probabilities often demonstrate a distinct characteristic. That is, their original form is rather complicated and corresponds to a high-frequency function on certain scales, but can be approximated by low-frequency functions on other scales with simple forms.

Supplementary Table 17 compares the recovery rates of different methods on quantum transition probability formulas from the AI-Feynman benchmark. Specifically, we performed the

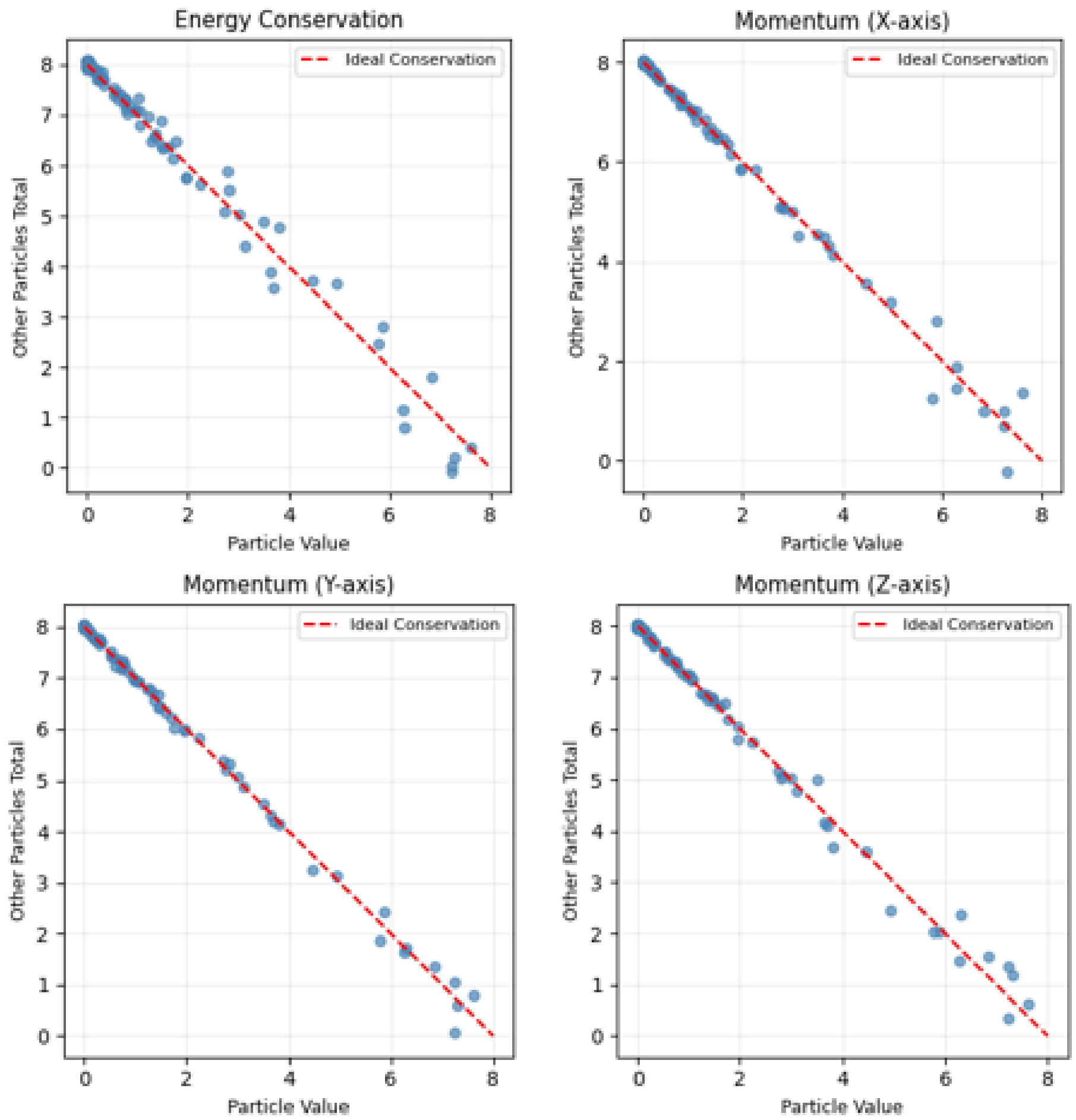


Supplementary Figure 9: Verification of Conservation Laws Learned by Deflexformer. The figure displays the relationship between the value of a single particle (x-axis) and the total value of the other particles (y-axis) sampled by the Deflexformer. **Top Left**: Energy Conservation. **Top Right, Bottom Left, Bottom Right**: Momentum Conservation in X, Y, and Z axes, respectively. The blue dots represent the sampled states, while the red dashed lines indicate the ideal conservation laws. The close alignment demonstrates that the Deflexformer has successfully learned to approximate these conservation manifolds.

formula discovery process on the AI-Feynman dataset 20 times and calculated the average number of times that the quantum transition probability formulas were successfully identified. As shown, Deflex demonstrates a lower recovery rate on this type of formulas than the compared baseline methods. The potential reason for low recovery would be due to the adverse effect of Deflex's multi-scale discovery. That is, Deflex searches for multiple formulas at different scales simultaneously. While formulas with very different scales coexist, strong patterns could dominate and suppress other correct formulas, leading to a lowered recovery rate. As we mentioned above, the original formula could be easily approximated by low-frequency functions on different scales with simple forms. In such cases, the low-frequency approximations would hinder the recovery of the original patterns of quantum transition probabilities. Differently, other comparatives were all single-scale methods and less impacted.

Supplementary Table 17: Comparison of average recovery rate on quantum transition probability formulas from the total 20 runs on the AI-Feynman benchmark.

| Method | Recovery frequency | Recovery rate |
|---|---|---|
| Deflex | 10/20 | 50% |
| AI Feynman | 16/20 | 80% |
| PySR | 12/20 | 60% |
| Operon | 13/20 | 65% |
| SciMED | 14/20 | 70% |
| gplearn | 13/20 | 65% |

## 7.8 Impact of scientist-in-the-loop approach on baseline methods

The scientist-in-the-loop approach is proposed [11] in AI-driven formula discovery, which improves the performance of existing methods. However, in the main text, we present the performance of existing methods in their raw form to ensure an aligned experimental setup. To provide complete references for readers, we briefly discuss this approach in this subsection and demonstrate additional evaluation results of the compared methods under this approach.

For SciMED [11], the scientist-in-the-loop approach injects prior knowledge over the space of the candidate formulas. In particular, implausible operators can be removed for a given task. For example, trigonometric operators like $\sin$ can be removed from the systems considered in this work. For variables with physical dimensions, dimensional information can be provided whenever feasible. The complexity can also be preset according to the expected target formula: the maximum tree depth can be set to $5$ for relatively simple formulas, whereas it can be increase to $15$ for more complex ones. When necessary, some minimal template-level constraints for structures such as summation can be retained for the expected formulas.

For AI Feynman [14], the scientist-in-the-loop approach works by providing dimensional constraints and restricted subsets of candidate operators. In addition, the polynomial-degree cutoff can be adjusted according to the expected complexity of the target formula, e.g., using a lower cutoff for simpler formulas and a higher cutoff for more complex ones. For more challenging formulas, a larger brute-force search budget can be also allowed.

To show a more complete performance of the compared methods, we implemented the above scientist-in-the-loop approach on SciMED and AI Feynman and conducted additional experiments on the particle and fluid tasks. Please note that, here, we mainly considered compact forms of prior knowledge that can be specified in a reasonably consistent manner across tasks. To ensure a rigorous and fair performance assessment, we kept the same overall computational budget and success criterion as in the main comparative setup. In particular, we used the same time budget of $20,000$ seconds and the same residual-error-based standard for successful recovery.

Supplementary Fig. 10 compares the discovery time of SciMED and AI Feynman with and without the scientist-in-the-loop approach. Please note that, since the experimental setups and convergence conditions are the same for both cases, the discovered formulas and their accuracy also remained unchanged and thus not reported. As shown, there is a noticeable reduction in the

discovery time in both SciMED or AI Feynman, by incorporating a limited scientist-in-the-loop setting with relatively generic prior knowledge.

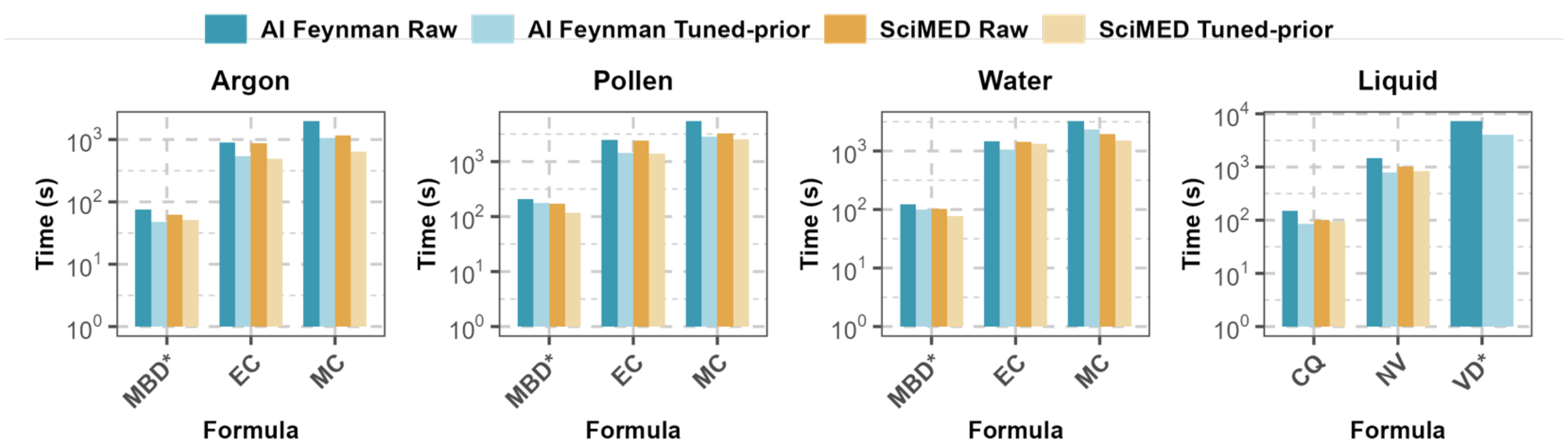


Supplementary Figure 10: Impact of scientist-in-the-loop configurations on discovery time for SciMED/AI Feynman. We compare the wall-clock runtime (log scale) under the default "raw" settings and tuned settings with manually provided feature/structure constraints in the Argon, Pollen, Water, and Fluid experiments.

# 8 Detailed algorithms and pseudocodes

The algorithmic implementation of Deflex is detailed below, encompassing data representation, neural network architecture, energy-based learning, and the overall workflow. This section provides the complete technical specifications, including network architectures, hyperparameter configurations, and pseudocode for all major computational modules.

## 8.1 Data representation and Deflexformer architecture

We denote the observation data as $\mathbf{X} = \{x_i\}_{i=1}^{N}$, where each $x_i$ comprises $n$ elements observed over $t$ time frames. In our formulation, each observation may include both element-level states $S$ and an optional global context $V$ characterizing the system at a macroscopic level (e.g., a system-wide temperature), i.e., $x = [S; V]$ with $V$ possibly absent; when $V$ is unavailable, we introduce global slots as fixed Gaussian padding tokens (sampled once and reused) rather than

resampling noise at every inference. Importantly, the elementwise and temporal axes are not mandatory: when a task provides only global descriptors (or does not benefit from element/time factorization), the model can operate with an empty elementwise tensor and treat the input entirely as global tokens. The Deflexformer network approximates an energy function $\mathcal{E}_\Theta : \mathbb{R}^{n \times t \times d_{in}} \to \mathbb{R}$ parameterized by $\Theta$. Each Deflexformer block $\mathcal{E}^i(\cdot)$ transforms element-level representations $P_i \in \mathbb{R}^{n \times t \times D_{model}}$ and global representations $G_i \in \mathbb{R}^{c \times D_{model}}$ into updated states $P_{i+1}$ and $G_{i+1}$. The embedding dimension is denoted $d$, the model hidden dimension $D_{model} = d_\ell$, the number of global tokens $c$, the number of Fourier scales $S$, and the data range $R$.

The Fourier-based multiscale embedding (Algorithm 2) maps input scalars to a high-dimensional vector space using a range of frequencies, capturing both fine-grained invariants and coarse-grained distributions. The inverse mapping (Algorithm 3) recovers scalar energy values. The Deflexformer architecture processes these representations via decomposable blocks (Algorithm 4) and a forward inference pass (Algorithm 5).

**Algorithm 2** Fourier Multiscale Embedding

**Input:** Embedding dimension $d$, scale count $S$, range $R$, input $\mathbf{u} \in \mathbb{R}^{d_{\text{in}}}$ ($d_{\text{in}} \leq d/(2S)$)
**Output:** Multiscale embedding $\mathbf{v} \in \mathbb{R}^{d}$

▷ Implements $\textsc{Embed}(\mathbf{u}, d, S, R)$

$d_{\text{in}} \leftarrow \textsc{Len}(\mathbf{u})$
$d_{\text{used}} \leftarrow 2Sd_{\text{in}}$
$\textsc{Assert}(d_{\text{used}} \leq d)$ ▷ Sine/cosine pairs must fit into the embedding
$\omega_0 \leftarrow \pi/R$ ▷ Base angular frequency covering $[-R, R]$
$\mathbf{v} \leftarrow \textsc{ZeroVec}(d)$ ▷ Zero padding for unused channels
$m \leftarrow 1$
**for** $s = 0 \to S-1$ **do**
  $\omega \leftarrow 2^s\omega_0$
  **for** $j = 1 \to d_{\text{in}}$ **do**
    $\mathbf{v}_m \leftarrow \sin(\omega u_j)$
    $\mathbf{v}_{m+1} \leftarrow \cos(\omega u_j)$
    $m \leftarrow m + 2$
  **end for**
**end for**
**while** $m \leq d$ **do**
  $\mathbf{v}_m \leftarrow 0$
  $m \leftarrow m + 1$
**end while**
**return v**

Algorithm 2 implements the Fourier multiscale embedding. The angular frequency $\omega = 2^s\omega_0$ generates sine-cosine pairs across scales $s$, establishing a harmonic basis that captures features at different resolutions. The base frequency $\omega_0 = \pi/R$ is calibrated to the data range $R$, ensuring the finest scale resolves variations across the full domain. Each input dimension $u_j$ produces $2S$ embedding channels (sine and cosine at $S$ scales), yielding a total of $d_{used} = 2Sd_{in}$ used channels. Unused channels are zero-padded to maintain a fixed layout $d$ for downstream blocks, facilitating parameter sharing across different input dimensionalities. This spectral representation is analogous to the positional encoding in Transformers [9] but adapted for continuous-valued physical quantities, enabling the network to distinguish between fine-grained invariants (high-frequency components) and coarse-grained distributions (low-frequency com-

ponents).

**Algorithm 3** Inverse Fourier Multiscale Embedding

**Input:** Embedding dimension $d$, scale count $S$, range $R$, input width $d_{\text{in}}$, embedding $\mathbf{v} \in \mathbb{R}^d$ (layout per Alg. 2)

**Output:** Recovered signal $\hat{\mathbf{u}} \in \mathbb{R}^{d_{\text{in}}}$

▷ Implements $\textsc{EmbedInv}(\mathbf{v}, d, S, R, d_{\text{in}})$

$\textsc{Assert}(2Sd_{\text{in}} \leq d)$
$\omega_0 \leftarrow \pi/R$
$\hat{\mathbf{u}} \leftarrow \textsc{ZeroVec}(d_{\text{in}})$
▷ Coarse reconstruction from lowest frequency
**for** $j = 1 \to d_{\text{in}}$ **do**
  $i \leftarrow 2(j-1)+1$
  $\theta \leftarrow \text{atan2}(\mathbf{v}_i, \mathbf{v}_{i+1})$
  $\hat{u}_j \leftarrow \theta/\omega_0$
**end for**
▷ Gradient refinement with higher harmonics
**for** $s = 1 \to S-1$ **do**
  $\omega \leftarrow 2^s\omega_0$
  $\eta_s \leftarrow 2^{-s}$ ▷ Diminishing step size for stability
  **for** $j = 1 \to d_{\text{in}}$ **do**
    $i \leftarrow 2(sd_{\text{in}} + j - 1) + 1$
    $\hat{\theta} \leftarrow \omega\hat{u}_j$
    $e_{\sin} \leftarrow \sin(\hat{\theta}) - \mathbf{v}_i$
    $e_{\cos} \leftarrow \cos(\hat{\theta}) - \mathbf{v}_{i+1}$
    $g \leftarrow \omega(e_{\sin}\cos(\hat{\theta}) - e_{\cos}\sin(\hat{\theta}))$
    $\hat{u}_j \leftarrow \hat{u}_j - \eta_s g$
  **end for**
**end for**
**return** $\hat{\mathbf{u}}$

Algorithm 3 performs the inverse transformation from global channels to scalar energies. The reconstruction proceeds in two stages. First, principal angles are retrieved via $\text{atan2}(\sin(\omega_0 u), \cos(\omega_0 u))$ at the base frequency $\omega_0$, providing a coarse estimate of the underlying scalar. Second, higher-frequency harmonics refine this estimate through iterative gradient corrections. For each scale $s > 0$, the reconstruction error in the sine and cosine channels is computed, and a gradient-based update is applied with step size $\eta_s = 2^{-s}$. The diminishing step sizes ensure stability as

higher harmonics contribute finer details. This hierarchical refinement mirrors the multiscale nature of the forward embedding, linking the learned latent representation back to the scalar energy values required by the symbolic regression module. The inversion is approximate but sufficient for guiding the evolutionary search, as the primary role is to provide relative orderings rather than exact reconstructions.

**Algorithm 4** Deflexformer Block Map

**Input:** Element state $P \in \mathbb{R}^{n \times t \times d_\ell}$, Global state $G \in \mathbb{R}^{c \times d_\ell}$
**Output:** Updated states $P \in \mathbb{R}^{n \times t \times d_\ell}, G \in \mathbb{R}^{c \times d_\ell}$

▷ 1. Spatial Mixing (Dimension: $n$)
1: $H \leftarrow G_{\text{expand}} || P \in \mathbb{R}^{t \times (c+n) \times d_\ell}$ ▷ Concatenation along $n$
2: $H \leftarrow \text{LayerNorm}(H + \text{MHA}(H))$ ▷ Self-attention over $c + n$
3: $G', P \leftarrow \text{Split}(H)$ ▷ Separate global/element states
4: $G \leftarrow \text{Mean}(G', \dim = t)$ ▷ Aggregate temporal updates
▷ 2. Temporal Mixing (Dimension: $t$)
5: $H \leftarrow G_{\text{expand}} || P \in \mathbb{R}^{n \times (c+t) \times d_\ell}$ ▷ Concatenation along $t$
6: $H \leftarrow \text{LayerNorm}(H + \text{MHA}(H, \text{mask}))$ ▷ Mask: $G$ visible to all; $P$ causal
7: $G', P \leftarrow \text{Split}(H)$
8: $G \leftarrow \text{Mean}(G', \dim = n)$ ▷ Fuse spatial updates
9: **return** $P, G$

Algorithm 4 formally defines the Deflexformer block transformation, mapping input tensors $P \in \mathbb{R}^{n \times t \times d_\ell}$ and $G \in \mathbb{R}^{c \times d_\ell}$ to updated states. The process consists of two decoupled attention mechanisms. First, spatial mixing concatenates $G$ (broadcast along $t$) and $P$ along the element dimension $n$, yielding $H^{(s)} = G_{\text{expand}} || P \in \mathbb{R}^{t \times (c+n) \times d_\ell}$. Multi-head attention with $h = 8$ heads is performed over the $(c+n)$ axis, enabling dense cross-element interactions, and the global partition is aggregated via mean pooling over $t$. Second, temporal mixing concatenates the updated $G$ (broadcast along $n$) and $P$ along time dimension $t$, forming $H^{(t)} = G_{\text{expand}} || P \in \mathbb{R}^{n \times (c+t) \times d_\ell}$. Multi-head attention is applied with a causal mask $M^{(t)}$ where $M^{(t)}_{ij} = 0$ if $j \le c$ or $c < j \le i$, otherwise $-\infty$, ensuring global tokens attend to the entire sequence while element tokens attend only to their causal history. Each block also includes a point-wise transformation network

implemented as a four-layer fully connected network with architecture $(d_\ell, 2d_\ell, 2d_\ell, d_\ell)$, ReLU activations, and layer normalization.

The multi-head attention mechanism is computed as in standard Transformer architectures [9]:

$$\text{MHA}(Q, K, V, M) = \text{Concat}(\text{head}_1, \ldots, \text{head}_h)W^O, \tag{20}$$

where each head applies scaled dot-product attention:

$$\text{head}_i = \text{softmax}\left(\frac{Q_i K_i^T}{\sqrt{d_k}} + M\right) V_i, \tag{21}$$

with $d_k = d_\ell / h$. For spatial mixing, $H^{(s)} \leftarrow \text{LayerNorm}(H^{(s)} + \text{MHA}(H^{(s)}, H^{(s)}, H^{(s)}, M^{(s)}))$ where $M^{(s)} = 0$. For temporal mixing, $H^{(t)} \leftarrow \text{LayerNorm}(H^{(t)} + \text{MHA}(H^{(t)}, H^{(t)}, H^{(t)}, M^{(t)}))$ where:

$$M_{ij}^{(t)} = \begin{cases} 0 & \text{if } j \leq c \text{ or } (j > c \text{ and } j \leq i), \\ -\infty & \text{otherwise.} \end{cases} \tag{22}$$

The default configuration uses $d_\ell = D_{model} = 64$, $c = 8$, $h = 8$, $S = 4$, and $k = 4$ to 8 blocks. For particle energy $E = \frac{1}{2} m_i \mathbf{v}_i^2 + U(\mathbf{x}_i)$, each block captures elementary transformations via the decomposable architecture.

**Algorithm 5** Deflexformer Forward Sweep

**Input:** Observation $x$, block count $k$, element width $d$, local width $d_\ell$, scale count $S$, range $R$
**Output:** Intermediate sequence $\mathcal{R} = (\mathcal{R}_0, \ldots, \mathcal{R}_k)$, encoded scalar $e_x$

▷ Implements $F_{\text{fwd}}(x; k, d, d_\ell, S, R)$

$\mathbf{v}_0 \leftarrow \textsc{Embed}(x, d, S, R)$ ▷ Fourier multiscale embedding of raw input
$(P_0, G_0) \leftarrow \textsc{StateInit}(\mathbf{v}_0, d_\ell)$ ▷ Split into element/global tensors (with $P_0$ possibly empty)
$\mathcal{R}_0 \leftarrow (P_0, G_0)$
**for** $i = 0 \to k - 1$ **do**
    $(P_{i+1}, G_{i+1}) \leftarrow F_{\text{block}}^{(i)}(P_i, G_i)$ ▷ Apply Deflexformer block $i$
    $\mathcal{R}_{i+1} \leftarrow (P_{i+1}, G_{i+1})$
**end for**
$\mathbf{g} \leftarrow F_{\text{proj}}(G_k, 2S)$ ▷ Select leading global channels
$e_x \leftarrow \textsc{EmbedInv}(\mathbf{g}, 2S, S, R, 1)$ ▷ Invert embedding to scalar energy
**return** $\mathcal{R}, e_x$

Algorithm 5 outlines the complete forward pass of Deflexformer. Raw observation data $x$ (comprising element states across time) are first embedded via $\textsc{Embed}(x, d, S, R)$ into a high-dimensional spectral representation $\mathbf{v}_0$. The $\textsc{StateInit}(\cdot)$ function splits this representation into element-level tensors $P_0 \in \mathbb{R}^{n\times t\times d_\ell}$ and global-level tensors $G_0 \in \mathbb{R}^{c\times d_\ell}$ through learned linear projections. Consistent with Algorithm 2, elementwise embeddings use zero padding for unused channels, while the global context is initialised as follows: when auxiliary global descriptors are available, we embed them using the same Fourier multiscale map and project them to initialise $G_0$; otherwise, we treat the global context as Gaussian padding tokens and initialise $G_0$ once as $G_0 \sim \mathcal{N}(0, I)$, keeping this padding fixed across forward passes rather than resampling it at every inference. In purely symbolic regression settings without an explicit notion of entities or time (e.g., SR over common tabular datasets), we place all input features into the global descriptor set and let the element-wise tensor be empty (so the forward sweep reduces to transformations over $G$). For static observations without temporal sequences, we treat inputs as a single frame ($t = 1$), and the temporal-mixing component becomes a degenerate case. Successive applications of $k$ Deflexformer blocks $\mathcal{E}^0, \ldots, \mathcal{E}^{k-1}$ yield intermediate representations $\mathcal{R}_i = (P_i, G_i)$ for $i = 0, \ldots, k$. Each block refines the representation by modeling element interactions (spatial mixing) and temporal dependencies (temporal mixing). After the final block, the global state $G_k$ encodes a compact summary of the entire system. A projection layer $F_{proj}$ selects the leading $2S$ global channels, which are then inverted via $\textsc{EmbedInv}(\cdot)$ to produce the scalar energy $e_x \in \mathbb{R}$. The sequence $\mathcal{R}$ of all intermediate states is retained for hierarchical symbolic regression, enabling the recovery of block-wise formulas that compose into the global energy function.

## 8.2 Neural-guided expression generator architecture

Let $\mathcal{V}$ denote the vocabulary of lambda-calculus tokens and $\mathbf{m} = (m_1, \ldots, m_L)$ a serialized expression where $m_i \in \mathcal{V}$ (with $L$ being the expression length). The neural generator models the conditional probability $P(m_{i+1}|m_1, \ldots, m_i)$ autoregressively.

The Deflexpressor neural generator is implemented as a standard Transformer decoder [9] with $N_{layer} = 6$ layers, model dimension $d_{exp} = 64$, and $h_{exp} = 8$ attention heads. Each token is embedded via $W_{emb} \in \mathbb{R}^{|\mathcal{V}| \times d_{exp}}$ and combined with sinusoidal positional encodings before being processed through masked self-attention and feed-forward layers with dimension $d_{ff} = 256$. The network is trained on $N_{gen} = 128{,}000$ randomly generated expressions and $N_{mut} = 54{,}000$ mutation pairs using next-token prediction with cross-entropy loss. We use the AdamW optimizer with learning rate $5 \times 10^{-4}$ and batch size 32 for 200 epochs. During inference, the network operates in generation mode (sampling from scratch) or mutation mode (using existing expressions as prompts), with all outputs validated against the type system.

## 8.3 Hierarchical symbolic regression

Let $\mathcal{R}^{(x)} = (\mathcal{R}_0, \ldots, \mathcal{R}_k)$ denote the sequence of intermediate representations for observation $x$, where $\mathcal{R}_i = (P_i, G_i)$ is the state after block $i$. Here $(P_i, G_i)$ denotes the paired element-level and global-level representations, which when concatenated form $P_i||G_i$ as used in the main text. The hierarchical regression seeks to identify formulas $f_i : \mathcal{R}_i \to \mathcal{R}_{i+1}$ for each block and compose them into a global formula $f_{global} : \mathcal{R}_0 \to \mathcal{R}_k$.

To recover the global formula governing the entire Deflexformer, we introduce a hierarchical regression strategy that iteratively composes block-wise discoveries (Algorithm 6). This process bridges the gap between local layer transformations and the global system law.

**Algorithm 6** Block-wise Hierarchical Regression

**Input:** Block representations $\mathcal{H} = \{\mathcal{R}^{(x)}\}_{x \in \mathbf{X}}$ where $\mathcal{R}^{(x)} = (\mathcal{R}_0, \ldots, \mathcal{R}_k)$
**Output:** Global formula $f_{\text{global}}$

▷ 1. Base Layer Regression

$\mathcal{F}^{(0)} \leftarrow \emptyset$
**for** $i = 0 \to k-1$ **do**
    $\mathcal{D}_i \leftarrow \{(\mathcal{R}_i^{(x)}, \mathcal{R}_{i+1}^{(x)}) \mid x \in \mathbf{X}\}$ ▷ Input-output pairs for block $i$
    $f_i \leftarrow \textsc{ExprFit}(\mathcal{D}_i)$ ▷ Find best formula for block $i$
    $\mathcal{F}^{(0)} \leftarrow \mathcal{F}^{(0)} \cup \{(f_i, i, i+1)\}$ ▷ Store formula with its coverage range
**end for**

▷ 2. Iterative Composition (Tree Reduction)

$L \leftarrow 0$
**while** $|\mathcal{F}^{(L)}| > 1$ **do**
    $\mathcal{F}^{(L+1)} \leftarrow \emptyset$
    $M \leftarrow |\mathcal{F}^{(L)}|$
    **for** $j = 0$ to $M-1$ step 2 **do**
        **if** $j+1 < M$ **then**
            $(f_A, s_A, e_A) \leftarrow \mathcal{F}_j^{(L)}$ ▷ Formula A covering layers $s_A$ to $e_A$
            $(f_B, s_B, e_B) \leftarrow \mathcal{F}_{j+1}^{(L)}$ ▷ Formula B covering layers $s_B$ to $e_B$
            $f_{\text{seed}} \leftarrow f_B \circ f_A$ ▷ Function composition as initial seed
            ▷ Refine using data spanning from start of A to end of B
            $\mathcal{D} \leftarrow \{(\mathcal{R}_{s_A}^{(x)}, \mathcal{R}_{e_B}^{(x)}) \mid x \in \mathbf{X}\}$
            $f_{\text{new}} \leftarrow \textsc{ExprFit}(\mathcal{D}, \text{init} = \{f_{\text{seed}}\})$
            $\mathcal{F}^{(L+1)} \leftarrow \mathcal{F}^{(L+1)} \cup \{(f_{\text{new}}, s_A, e_B)\}$
        **else**
            $\mathcal{F}^{(L+1)} \leftarrow \mathcal{F}^{(L+1)} \cup \{\mathcal{F}_j^{(L)}\}$ ▷ Pass through odd block
        **end if**
    **end for**
    $L \leftarrow L+1$
**end while**
$(f_{\text{final}}, \_, \_) \leftarrow \mathcal{F}_0^{(L)}$
**return** $f_{\text{final}}$

Algorithm 6 details the hierarchical reduction process. First, independent symbolic regression is performed for each Deflexformer block $i$ using input-output pairs $(\mathcal{R}_i^{(x)}, \mathcal{R}_{i+1}^{(x)})$ to obtain local formulas $f_i$. Then, adjacent formulas are iteratively composed in a tournament-style reduction. For formulas $f_A$ and $f_B$ covering layers $s_A$ to $e_A$ and $s_B$ to $e_B$, the composition

$f_{seed} = f_B \circ f_A$ serves as an initial candidate, which is refined via symbolic regression on $\mathcal{D} = \{(\mathcal{R}^{(x)}_{s_A}, \mathcal{R}^{(x)}_{e_B})\}$. The process recurses until a single formula spans the entire network. For instance, when discovering $E = \frac{1}{2}m_i\mathbf{v}_i^2 + U(\mathbf{x}_i)$ from particle dynamics, the first block might capture $\frac{1}{2}m_i\mathbf{v}_i^2$ while subsequent blocks learn $U(\mathbf{x}_i)$, and the hierarchical regression composes them into the full energy function.

## 8.4 Energy-based learning

Let $E_\Theta(\mathbf{x})$ denote the energy function parameterized by network weights $\Theta$ (uppercase for algorithm notation) with corresponding probability distribution $Q_\Theta(\mathbf{x}) = \frac{1}{Z_\Theta}\exp(-E_\Theta(\mathbf{x}))$, where $Z_\Theta = \int \exp(-E_\Theta(\mathbf{x}))d\mathbf{x}$ is the intractable partition function. Positive samples $X^+$ are drawn from the observation distribution $P_{data}(\mathbf{x})$ while negative samples $X^-$ are synthesized via Langevin dynamics to approximate $Q_\Theta(\mathbf{x})$. Here $X^+$ and $X^-$ denote batches of positive and negative samples respectively.

Training is conducted within an Energy-Based Model (EBM) framework using Contrastive Divergence with Langevin Dynamics [1, 2] (Algorithm 7) to optimize model parameters. The contrastive divergence objective approximates maximum likelihood estimation by minimizing the energy on observed data while maximizing it on model-generated samples, bypassing the need to compute the partition function explicitly.

**Algorithm 7** Energy-based Optimisation of Deflexformer via MCMC

**Input:** Deflexformer $E_\Theta$, observation set $\mathbf{X}$, iterations $T$, chain length $L$, step size $\alpha$
**Output:** Trained parameters $\Theta^\star$

**for** $t = 1 \to T$ **do**
  $X^+ \leftarrow \textsc{BatchDraw}(\mathbf{X})$ ▷ Positive batch from observations
  $X^{-(0)} \leftarrow \textsc{NoiseInit}(|X^+|)$ ▷ Initial negative particles
  ▷ Langevin refinement of negative samples
  **for** $\ell = 0 \to L-1$ **do**
    $g \leftarrow \nabla_X E_\Theta(X^{-(\ell)})$ ▷ Energy gradient w.r.t. inputs
    $z \sim \mathcal{N}(0, I)$ ▷ Gaussian perturbation
    $X^{-(\ell+1)} \leftarrow X^{-(\ell)} - \frac{\alpha}{2} g + \sqrt{\alpha}\, z$
  **end for**
  $X^- \leftarrow X^{-(L)}$
  ▷ Contrastive divergence update
  $\ell(\Theta) \leftarrow \frac{1}{|X^+|} \sum_{x \in X^+} E_\Theta(x) - \frac{1}{|X^-|} \sum_{x \in X^-} E_\Theta(x)$
  $\Theta \leftarrow \Theta - \eta \nabla_\Theta \ell(\Theta)$
**end for**
**return** $\Theta$

Algorithm 7 details the contrastive divergence optimization. At each iteration, positive samples $X^+$ are drawn from observations while negative samples are initialized from noise $X^{-(0)}$ and refined through $L$ Langevin steps: $X^{-(\ell+1)} \leftarrow X^{-(\ell)} - \frac{\alpha}{2} \nabla_X E_\Theta + \sqrt{\alpha} z$ where $z \sim \mathcal{N}(0, I)$. The contrastive loss $\ell(\Theta) = \mathbb{E}_{X^+}[E_\Theta] - \mathbb{E}_{X^-}[E_\Theta]$ pushes down energy on observed data and pushes up energy on model-generated samples. The step size $\alpha = \frac{\max_{\mathbf{x}} \|\mathbf{x}\|}{2L}$ scales with data range, and a persistent buffer retains 95% of negative samples between iterations to reduce sampling cost. For particle systems with $E = \frac{1}{2} m_i \mathbf{v}_i^2 + U(\mathbf{x}_i)$, this procedure learns to assign low energy to configurations respecting conservation laws and high energy to physically implausible states.

## 8.5 Overall workflow

Let $\mathcal{E}$ denote the set of generated lambda expressions, $\mathcal{Z}$ the synthetic dataset derived from $\mathcal{E}$, $\theta_0$ the parameters of a single pre-trained block, $\Theta$ the parameters of the full Deflexformer, $\mathcal{H}$

the collection of block-wise representations, $\mathcal{Y}$ the set of scalar energy values, and $\mathbf{f}^\star$ the final discovered formulas on the Pareto frontier.

The complete Deflex pipeline is summarized in Algorithm 8, orchestrating expression generation, pre-training, EBM learning, and symbolic regression. The workflow consists of four distinct phases: Phase 1 bootstraps mathematical priors through synthetic data generation, Phase 2 learns the system's energy landscape from observations, Phase 3 extracts hierarchical representations via forward inference, and Phase 4 recovers interpretable formulas through block-wise symbolic regression.

---

**Algorithm 8** Deflex Workflow

**Input:** Observation set $\mathbf{X} = \{x_i\}_{i=1}^{N}$, block count $k$, element width $d$, local width $d_\ell$, scale count $S$, range $R$

**Output:** Pareto-optimal formulas $\mathbf{f}^\star$, encoded scalars $\{e_x\}_{x \in \mathbf{X}}$

**Phase 1: Generation & Pre-training**

$\mathcal{E} \leftarrow \textsc{ExprSample}$ ▷ Draw lambda expressions via Deflexpressor

$\mathcal{Z} \leftarrow \textsc{DataSample}(\mathcal{E})$ ▷ Synthesize paired data from the expressions

$\theta_0 \leftarrow \textsc{BlockFit}(\mathcal{Z})$ ▷ Pre-train a single Deflexformer block

**Phase 2: Learning**

$E_\Theta \leftarrow \textsc{Cascade}(\theta_0, k)$ ▷ Stack the pre-trained block $k$ times

$\Theta \leftarrow \textsc{EBMFit}(E_\Theta, \mathbf{X})$ ▷ Train Deflexformer as an energy model on $\mathbf{X}$

**Phase 3: Inference**

$\mathcal{H} \leftarrow \emptyset$ ▷ Store block-wise representations

$\mathcal{Y} \leftarrow \emptyset$ ▷ Store scalar energies

**for** $x \in \mathbf{X}$ **do**

  $(\mathcal{R}^{(x)}, e_x) \leftarrow F_{\text{fwd}}(x; k, d, d_\ell, S, R)$ ▷ See Alg. 5

  $\mathcal{H} \leftarrow \mathcal{H} \cup \{\mathcal{R}^{(x)}\}$

  $\mathcal{Y} \leftarrow \mathcal{Y} \cup \{e_x\}$

**end for**

**Phase 4: Regression**

$f^\star \leftarrow \textsc{HierarchicalRegression}(\mathcal{H})$ ▷ See Alg. 6

**return** $f^\star, \mathcal{Y}$

---

Algorithm 8 coordinates the four phases. Phase 1 generates synthetic data $\mathcal{Z}$ from lambda expressions $\mathcal{E}$ and pre-trains a single Deflexformer block to obtain $\theta_0$, instilling mathematical

priors. Phase 2 cascades this block $k$ times to form the full network and trains it on observations $\mathbf{X}$ via energy-based learning, learning the specific energy landscape of the target system. Phase 3 performs forward inference to extract intermediate representations $\mathcal{R}^{(x)}$ and scalar energies $e_x$ for each observation. Phase 4 applies hierarchical symbolic regression to $\mathcal{H}$ to recover interpretable formulas, balancing complexity and accuracy to yield Pareto-optimal candidates $\mathbf{f}^{\star}$.

Supplementary Table 18 summarizes the complete hyperparameter configuration for all algorithmic components of Deflex. These settings were used across all experiments unless otherwise specified.

Supplementary Table 18: Detailed Hyperparameter Configuration for Deflex

| **Component** | **Parameter** | **Value** |
|---|---|---|
| Deflexformer | Model dimension $D_{model}$ | 64 |
| | Number of blocks $k$ | 4–8 (task-dependent) |
| | Global token count $c$ | 8 |
| | Attention heads $h$ | 8 |
| | FFN hidden dimension | $4 \times D_{model} = 256$ |
| | Fourier scales $S$ | 4 |
| | Optimizer | Adam |
| | Learning rate $\eta$ | $10^{-4}$ |
| | Batch size | 16 |
| | Weight decay | 0 |
| Expression Generator | Model dimension $d_{exp}$ | 64 |
| | Decoder layers $N_{layer}$ | 6 |
| | Attention heads $h_{exp}$ | 8 |
| | FFN dimension $d_{ff}$ | 256 |
| | Optimizer | AdamW |
| | Learning rate $\alpha_{exp}$ | $5 \times 10^{-4}$ |
| | Weight decay $\lambda_{exp}$ | $10^{-5}$ |
| | Batch size $B_{exp}$ | 32 |
| | Training epochs $E_{exp}$ | 200 |
| | Generation corpus $N_{gen}$ | 128,000 |
| | Mutation pairs $N_{mut}$ | 54,000 |
| EBM Training | Langevin chain length $L$ | 128 (initial), 8 (buffer) |
| | Langevin step size $\alpha$ | $\frac{\max_{\mathbf{x}} \|\mathbf{x}\|}{2L}$ |
| | Negative samples $M$ | 6–128 (task-dependent) |
| | Buffer retention | 95 |
| | Training iterations $T$ | 15,000 |
| Symbolic Regression | Initial population | 20 |
| | Population (annealed) | 5 |
| | Breeding size $N_p$ | 50 |
| | Flexible threshold $M_s$ | 100 |
| | Rigid threshold $M_r$ | 200 |
| | Elimination probability $P_d$ | 0.1 |
| | Neural mutation ratio | Anneal 100 |
| | Regression steps | 15,000 |

## Supplementary References


[1] Song, Y. & Kingma, D. P. How to train your energy-based models. *arXiv preprint arXiv:2101.03288* (2021).

[2] Parisi, G. Correlation Functions and Computer Simulations. *Nucl. Phys. B* **180**, 378 (1981).

[3] Grenander, U. & Miller, M. I. Representations of knowledge in complex systems. *Journal of the Royal Statistical Society: Series B (Methodological)* **56**, 549–581 (1994).

[4] Church, A. An unsolvable problem of elementary number theory. *American Journal of Mathematics* **58**, 345–363 (1936).

[5] Barendregt, H. P. *The Lambda Calculus: Its Syntax and Semantics*, 23–31, 54–60 (North-Holland, Amsterdam, 1984), revised 3rd edn.

[6] De Bruijn, N. G. Lambda calculus notation with nameless dummies, a tool for automatic formula manipulation, with application to the church-rosser theorem. In *Indagationes mathematicae (proceedings)*, vol. 75, 381–392 (Elsevier, 1972).

[7] Milner, R. A theory of type polymorphism in programming. *Journal of Computer and System Sciences* **17**, 348–375 (1978).

[8] Koza, J. R. Genetic programming as a means for programming computers by natural selection. *Statistics and computing* **4**, 87–112 (1994).

[9] Vaswani, A. *et al.* Attention is all you need. In Guyon, I. *et al.* (eds.) *Advances in Neural Information Processing Systems*, vol. 30 (Curran Associates, Inc., New York, NY, 2017).

[10] Burlacu, B., Kronberger, G. & Kommenda, M. Operon c++: An efficient genetic programming framework for symbolic regression. In *Proceedings of the 2020 Genetic and Evolutionary Computation Conference Companion*, GECCO '20, 1562–1570 (Association for Computing Machinery, New York, NY, USA, 2020).

[11] Keren, L. S., Liberzon, A. & Lazebnik, T. A computational framework for physics-informed symbolic regression with straightforward integration of domain knowledge. *Scientific Reports* **13**, 1249 (2023).

[12] Brunton, S. L., Proctor, J. L. & Kutz, J. N. Discovering governing equations from data by sparse identification of nonlinear dynamical systems. *Proceedings of the National Academy of Sciences* **113**, 3932–3937 (2016).

[13] Udrescu, S.-M. *et al.* Ai feynman 2.0: Pareto-optimal symbolic regression exploiting graph modularity. In Larochelle, H., Ranzato, M., Hadsell, R., Balcan, M. & Lin, H. (eds.) *Advances in Neural Information Processing Systems*, vol. 33, 4860–4871 (Curran Associates, Inc., 2020).

[14] Udrescu, S.-M. & Tegmark, M. Ai feynman: A physics-inspired method for symbolic regression. *Science Advances* **6**, eaay2631 (2020).

[15] Fortin, F.-A., De Rainville, F.-M., Gardner, M.-A., Parizeau, M. & Gagné, C. DEAP: Evolutionary algorithms made easy. *Journal of Machine Learning Research* **13**, 2171–2175 (2012).

[16] La Cava, W. *et al.* Contemporary symbolic regression methods and their relative performance. *Advances in neural information processing systems* **2021**, 1 (2021).

[17] Weinan, E. *Principles of multiscale modeling* (Cambridge University Press, Cambridge, UK, 2011).

[18] Hu, Y., Li, T.-M., Anderson, L., Ragan-Kelley, J. & Durand, F. Taichi: A language for high-performance computation on spatially sparse data structures. *ACM Trans. Graph.* **38** (2019). URL `https://doi.org/10.1145/3355089.3356506`.

[19] Blanke, U., Guldener, R., Feese, S. & Tröster, G. Crowdsourced pedestrian map construction for short-term city-scale events. In *Proceedings of the First International Conference on IoT in Urban Space*, URB-IOT '14, 25–31 (ICST (Institute for Computer Sciences, Social-Informatics and Telecommunications Engineering), Brussels, BEL, 2014). URL `https://doi.org/10.4108/icst.urb-iot.2014.257190`.

[20] Fink, D. *et al.* ebird status and trends, data version: 2022; released: 2023 (2023).

[21] Li, Y. *et al.* A public turbulence database cluster and applications to study lagrangian evolution of velocity increments in turbulence. *Journal of Turbulence* N31 (2008).

[22] Schläpfer, M. *et al.* The universal visitation law of human mobility. *Nature* **593**, 522–527 (2021).
