# Supplementary material for: Discovering multiscale deep formulas in complex systems via neural-guided lambda calculus
Source: Nat Commun. 2026 Jun 16;17:7639. doi: 10.1038/s41467-026-74299-z (PMC13434754; doi:10.1038/s41467-026-74299-z)
Supplement: Supplementary file 2 [file 41467_2026_74299_MOESM2_ESM.pdf]

nature portfolio

Peer Review File

Discovering Multiscale Deep Formulas in Complex Systems via Neural-Guided Lambda Calculus

Corresponding Author: Professor Shusen Yang

This file contains all editorial decision letters in order by version, followed by all author rebuttals in order by version.

Attachments originally included by the reviewers as part of their assessment can be found at the end of this file.

Version 0:

Reviewer comments:

Reviewer #1

(Remarks to the Author)
The study proposed a timely and intresting subject. Nevertheless, the current study has many shortcoming the authors should consider.
1. the title is somewhat generic - there are several papers online starting with this name.
2. the abstract is very generic and does not provide any details on the proposed method nor on the results one can obtain with it, making it unwelcoming.
3. "do not require prior knowledge, but fail to abstract concise math expressions from a large number of state variables in complex systems, due to their lack of compression and formula length reduction." - not true... Even gplearn (which is old already) provides such capabilities. In a more general sense, knowledge-integrated symbolic regression also does not align with this claim.
4. Table 1 shows very basic equations, with expression three of up to 8 nodes (If I computed correctly) - this experiment does not align with the huge promise of a "very deep expression trees". Even AI Feynman (I) shows more complex examples, and it is 6 years old now...
5. The comparison is with only AI Feynman and PySR. I would expect to see at least SINDy and SciMED and maybe gplearn and DEAP as baselines. You can have more options from the recent SRbenchmark paper.
6. "Deflex can also reveal undocumented system relationships from the observation data." - not sure I understand from the results section how you proved this point.
7. "Compared with state-of-the-art, Deflex achieves superior performance in both accuracy" - From Fig. 4 it seems Deflex does not outperform PySR or Ai Feynman in both NLL and RMSE.
8. The last paragraph in the Discussion section overpromises the empirical results presented in the study.
9. For a physics-inspired symbolic regression paper, the related work part is highly limited given the huge number of papers from the last several years and the development of the field.

More spesisfic:
1. First statement - cite?
2. The claim with cites 5-11, this is not true, the authors should check symbolic regression papers from 2023 forward, there are many counter examples.
3. "Such chaos and order emergence also introduces different levels of regularity, requiring us to account for both conservation laws and stochastic behaviors in scientific analysis" - cite?
4. "AI methods show great potential for discovering the governing rules behind the interactions of massive elements at multiple scales in these complex systems" - right, but spesisfic examples?
5. "Furthermore, their search space grows exponentially with the number of variables and expression depth [8, 18], making them computationally prohibitive to identify complex, nonlinear equations in such systems." - you can claim it to any reinforcement learning problem but still, it is working. This is because the search process is more important than the option space size.
6. "However, traditional symbol-tree-based symbolic regressions may fail to learn higher-order mathematical relationships such as summation and mapping among a large volume of elements in complex systems." - why? Even GA-based SR can do it, just define such functions as basic functions...
7. "which effectively addresses the aforementioned challenges for AI-based formula discovery from complex systems." - This

is a really bad practice to compliment yourself in your own work.
8. RMSE is basic; you do not need to state it in Eq. (1).
9. I find Figure 4 hard to read... Add a grid on the y-axis, keep the same colors, do not use shorts (P1) as it is hard to follow.
10. Figure 1 seems redundant to me, but the authors can leave it if they feel it is helpful.

Compliments:
1. I liked figures 2 + 3.
2. The methods section is well written.

(Remarks on code availability)
I was not able to see the code, which further supports my criticism of this study: https://prnt.sc/gMutxu3XL1wG

Reviewer #2

(Remarks to the Author)
The paper tackles the problem of identifying scale-specific formulas in multi-scale complex systems, a current challenge for existing AI-based symbolic regression algorithms. The authors achieve this by developing a novel AI-based framework called Deflex. By treating mathematical formulas as probability distributions within an energy-based model, in combination with lambda calculus, they are able to discover symbolic formulas in six different complex systems. They handle the usual issues related to the exponential explosion in the parameter space by breaking the complex system into smaller, manageable sub-problems. Their experiments show the efficiency of their algorithm in discovering formulas in multi-scale setups, performing better than other state-of-the-art algorithms. They are also able to discover new formulas in these systems, besides the established ones. Moreover, they lay down clear directions of future research for improving their algorithm. I believe that this is quite an original idea, which can have many applications in various fields of research. The experiments are explained clearly, and the obtained results are well interpreted. I believe that this is a very nice paper and should be accepted for publication.

(Remarks on code availability)

Reviewer #3

(Remarks to the Author)
The ideas presented by the authors are very interesting, this approach is a valuable contribution to explainable AI. Nevertheless, before this can be published, the authors definitively need to analyse the performance of the algorithm in further detail and document
* intermediate results (what do we get after each step of the algorithm)
* results on international benchmark data sets
* comparisons with other state of the art algorithms used on the same data sets

(Remarks on code availability)

Version 1:

Reviewer comments:

Reviewer #1

(Remarks to the Author)
I would like to start by complimenting the authors on the detailed revision, which addressed most of my concerns. The only thing I am not sure about is Table 2, which is the main claim of this study. The authors put V on all the equations, while in some this is just not true (for example, D != constant). The last equation is also not V, as there is no baseline, and the latter is not compared numerically. Moreover, I tested the Levy flight (both versions) and SciMED and gplearn was able to do it - which disagrees with the table. SciMED was able to find the Velocity distribution as well. In addition, not sure why it states that SINDy cannot run this equation? D * log(u)? This is as easy as it gets.
Very strange for me - for the "Newton viscosity law", if du/dx is not implicitly provided, all the methods should be N.A. and not V as they do not support it. If du/dx is provided explicitly, this is not a fair comparison. To this end, Velocity distribution for Deflex replaces "p" with "3.15" - sorry, this cannot considered a "V". The SR misses a term means it does not recreate the physics as it should do. Same for "Power law" and "Levy flight".

Moreover, the authors must indicate how many samples there are for each equation, how they sampled, and what features were provided to each model - otherwise, it is impossible to understand the experiments and the results.

By the way, why not provide the equation for all models? It is an important comparison that the readers should be aware of...

(Remarks on code availability)

Reviewer #3

(Remarks to the Author)
The authors did a very good job in improving the paper. Nevertheless, I can still not advise publishing it.
On the one hand, intermediate results are still not documented and discussed, only described superficially. This does not explain how the method works to the reader.
On the other hand, the evaluation section has been extended significantly, but is still very superficial. Statistically sound analysis including cross validation, mean / std / p-values, etc is still missing.

(Remarks on code availability)

Reviewer #4

(Remarks to the Author)
My review is attached.

(Remarks on code availability)

Version 2:

Reviewer comments:

Reviewer #1

(Remarks to the Author)
I would start this review by saying I suggested rejecting the paper the entire process - the experiments were poorly presented, the results seemed like cheating, and the model was described like black magic with "trust us" feeling. However, after several hard review rounds, I changed my mind. This time, I have several final comments which I think are important for the fairness of the claims for potential readers following them, I will recommend acceptance.

1. Proving "du/dt" is a fair methodological approach but missing the point of discovering "du/dt" from the raw "u" signal - the same argument you had for comment 1.1. You can argue this is a scientist in a loop here or some feature engineering pre the SR part. Both options are legitimate, but you must make this decision.
2. For SciMED, one of its main capabilities (which is also shared with AI Feynman) is that the user provides feature sets or constraints on the expression tree. I understand this cannot be fairly provided without shifting the results. However, it is worth stating that SciMED is used in its most "raw" version and one can gain better results with more "Scientist-in-the-loop".
3. In the discussion, as one of the main novelties of this study is the time-series capabilities, it would be nice to have a discussion on how such a capability can be used in different physical settings and implementation for experiment design, so practitioners will learn how to generate data for such models.
4. Most important - the current table with the main results is showing the method with "V" all the time. Find one example where the model does not work and explain why in the discussion. This is how you drive the field forward, where others have an open question to deal with.

(Remarks on code availability)
The code looks fine.

Reviewer #3

(Remarks to the Author)
Thanks for all the work and improvind that paper as recommended by the reviewers.

(Remarks on code availability)

Reviewer #4

(Remarks to the Author)
First, I would like to thank the authors for their extensive response to my questions. I think the many clarifications and inclusion of the Johns Hopkins Database have substantially strengthened the manuscript. I still have some remaining questions and confusion.

Here are some of my new questions now that more information has been supplied in the supplemental material:

1) Table 2 shows that Operon and SINDy both fail to capture the Navier-Stokes equation (NSE), however the supplemental material shows Operon succeeds at this for the 2D cylinder, and your response to comment 4.1 suggests that SINDy was able to find the Navier-Stokes after changing the settings. Table 2 makes it seem like these methods fail at finding the NSE, which is misleading if they work in some scenarios.
2) Why were spatial derivatives provided as inputs for finding the NSE? Why are these functions not simply learned from the data? The state of the system is only defined by the velocity and the pressure, so the spatial derivatives should not need to be supplied. Does the method fail if the spatial derivatives are not included?
3) In Table S8, Deflex learns the incorrect coefficients for energy conservation, Maxwell-Boltzmann, and Newtonian viscosity, whereas all of the other approaches do not get these incorrect coefficients. This appears to be a drawback of Deflex that is never explicitly stated. Why is Deflex the only method that gets these incorrect coefficients?
4) Even with the supplemental material, it is hard to decipher the Lambda Calculus sections. For example, Eq. 4 contains a substantial number of things that are never defined, like "tail" and "head", and from the brief description, I am unable to determine what "Fix" is doing. In the manuscript's current state, I do not think many readers will understand the sections on the Lambda Calculus.

Here are follow-ups on some of the previous responses:

4.1) What do you mean that the expression in [10] is more concise, but may not be Pareto-optimal? The NSE is the same regardless of how it is represented. Also, you wrote that the "...new experimental setups and results have been added in both manuscript...", but I did not see where these new SINDy results were added in the manuscript.
4.3) In Table 1, the Newton viscosity law is presented for all components of the viscous stress tensor, but the supplemental table S8 only shows one component of this tensor. Were the other components found and not presented in the supplemental material (the response to 4.15 says this table is a complete list of all unique formulas discovered)? Part of my confusion is that this is the only equation not presented as an energy with Deflex, and it remains unclear why this is the case.
4.11) The ablation study was useful for understanding the significance of the pretraining, but I have a couple of questions here. How is recovery speed defined? You explain that with a pre-trained model, it takes 5,000 steps for over 80% recovery rate, and from scratch, it requires 20,000 steps. It is unclear from this statement which approach is faster because this does not account for the pretraining time. If you account for the pretraining time, is it still faster to use the pretrained model? And, to clarify, is it correct that the timing in Figure 3 accounts for every step (including the pretraining) in the Deflex process?
4.13) In your response, you said that if no global information exists, it is introduced as noise. Is this in the manuscript or the SI? I could not find it when searching, but I might have just missed it.

Minor Issues:

1. Below Eq. 8 you write "Malkov" instead of "Markov"
2. I do not see where ND is defined in 4d,e,f. Is this supposed to be VD?

(Remarks on code availability)

Version 3:

Reviewer comments:

Reviewer #1

(Remarks to the Author)
The authors addressed all my concerns fully. I believe the paper can be accepted in its current form.

(Remarks on code availability)

Open Access This Peer Review File is licensed under a Creative Commons Attribution 4.0 International License, which permits use, sharing, adaptation, distribution and reproduction in any medium or format, as long as you give appropriate credit to the original author(s) and the source, provide a link to the Creative Commons license, and indicate if changes were made.
In cases where reviewers are anonymous, credit should be given to 'Anonymous Referee' and the source.
The images or other third party material in this Peer Review File are included in the article's Creative Commons license, unless indicated otherwise in a credit line to the material. If material is not included in the article's Creative Commons license and your intended use is not permitted by statutory regulation or exceeds the permitted use, you will need to obtain permission directly from the copyright holder.
To view a copy of this license, visit https://creativecommons.org/licenses/by/4.0/

Response to the Reviewers' Comments

Manuscript #: NCOMMS-25-26170-T

Dear reviewers,

We thank the reviewers for the helpful and insightful comments, which significantly improve the quality of the article. Compared with the original manuscript, considerable modifications have been made in this revision (marked as blue in the revised manuscript and supplementary material), which are summarized as follows:

1. **Extended Literature Review** (*Comments 1.9, 1.10, 1.11*): we have conducted a more thorough literature review on AI-based methods for scientific discovery, including symbolic regression, genetic programming.
2. **Additional Experimental Results** (*Comments 1.4, 1.5, 1.6, 3.2, 3.3*): we have conducted more extensive experiments and discussions to comprehensively evaluate the performance of our method. Specifically, we have added comparisons with ***five more state-of-the-art methods***, ***three more complex discovery tasks***, and an ***additional international benchmark dataset***.
3. **Statement Clarification and Correction** (*Comments 1.3, 1.7, 1.8, 1.12, 1.13, 1.14, 1.15, 1.16*): we have revised all the confusing statements and ambiguous writings pointed out by the reviewers.
4. **Improved Presentation** (*Comments 1.1, 1.2, 1.17, 1.18, 1.19, 3.1*)**:** we have improved many parts of the manuscript, including the title, abstract, tables and figures, to ensure a clear and concise presentation.
5. **Other Modifications** (*Comments 1.20*): we have fixed all other issues pointed out by the reviewers and found by ourselves.

Detailed point-by-point responses to reviewers' comments are as follows:

=======Reviewer #1=======

Comments to the Authors:

The study proposed a timely and interesting subject. Nevertheless, the current study has many shortcoming the authors should consider.

Compliments:

1. *I liked figures 2 + 3.*
2. *The methods section is well written.*

Response to Reviewer #1:

We thank the reviewer for the positive comments regarding the topic importance and recognizing the presentations and readability of the manuscript. We also thank the reviewer for later constructive comments and revision suggestions, which help us significantly improve the quality of our manuscript.

Comment 1.1:

the title is somewhat generic - there are several papers online starting with this name.

Response to Comment 1.1:

We thank the reviewer for this suggestion. The main contribution and novelty of our work lies in the integration of neural guidance with a lambda calculus-based symbolic system for multi-scale discovery of deep formulas in complex systems. To better reflect the specific technical contributions and scope of our work, we have revised the title in the revised manuscript as follows.

Revised Manuscript:

Discovering Multiscale Deep Formulas in Complex Systems via Neural-Guided Lambda Calculus

Original Manuscript:

Multi-scale Deep Formula Discovery in Complex Systems

Comment 1.2:

the abstract is very generic and does not provide any details on the proposed method nor on the results one can obtain with it, making it unwelcoming.

Response to Comment 1.2:

Thank you for this comment. We agree that the abstract could benefit from more specific details about our method's contributions and key results. To respond, we have carefully revised the abstract to summarize the core components/mechanisms of Deflex and their interactions with more details, and highlight Deflex's key advantages over state-of-the-art methods:

Revised Manuscript:

Abstract

A fundamental problem in science is identifying underlying patterns of complex systems into concise mathematical formulas. Current Artificial Intelligence (AI)-based methods have shown great success in single-scale systems, yet are incapable of identifying scale-specific formulas in multiscale complex systems. We design Deflex, an end-to-end AI method to automatically extract formulas with potentially different forms, including invariants and distributions, across different scales in complex systems. Deflex contains two subsystems named Deflexformer and Deflexpressor. Deflexformer utilizes energy models to unify representations of formulas across scales and adopts a self-attention network architecture for efficient layer-wise transformation of massive data. Deflexpressor is a symbolic regression model empowered by Lambda calculus for extracting higher-order mathematical formulas automatically. Deflex first leverages Deflexpressor to generate synthetic data for pre-training Deflexformer, and then uses it to guide automatic formula discovery by decoupling multiscale latent relationships. Deflex is validated in various real-world complex systems, including molecular motion, fluid dynamics, and flocking mobility. Results demonstrate that, compared with state-of-the-art AI methods, Deflex exhibits expanded capability and improved efficiency (by up to 7 times) in uncovering diverse formulas, particularly pioneering automated multiscale discovery. Our work could be a useful tool for scientific discovery in complex systems across distinct disciplines.

Original Manuscript:

Abstract

A fundamental problem in science is identifying underlying patterns of complex systems, and summarizing them into concise mathematical formulas. Current Artificial Intelligence (AI)-based methods have shown great success in single-scale systems, yet are incapable of identifying scale-specific formulas in multi-scale complex systems. We design Deflex, an end-to-end AI method to automatically extract formulas with potentially different forms including invariants and distributions, across different scales in complex systems. Specifically, we design an energy-based deep neural network model to decouple multi-scale latent relations into intermediate representations, from which meaningful invariants and distributions are then extracted by a symbolic lambda calculus system. This enables Deflex to discover deep formulas across massive elements and multiple scales, and makes it possible to uncover multi-scale rules in complex systems. Deflex is validated in various real-world complex systems, including molecular motion, fluid dynamics, and flocking mobility. Results show that, compared with state-of-the-art AI methods, Deflex not only outperforms with higher discovery accuracy and efficiency in various systems, but also fills the gap of automatic discovery of multi-scale formulas in complex systems.
Our work could be a useful tool for scientific discovery in complex systems across distinct disciplines.

Comment 1.3:

"do not require prior knowledge, but fail to abstract concise math expressions from a large number of state variables in complex systems, due to their lack of compression and formula length reduction." - not true... Even gplearn (which is old already) provides such capabilities. In a more general sense, knowledge-integrated symbolic regression also does not align with this claim.

Response to Comment 1.3:

We thank the reviewer for this comment. We agree that many GP frameworks incorporate mechanisms for parsimony, and formula length reduction and symbolic regression is often

integrated with prior knowledge despite. We have revised the discussion about these relevant studies to more clearly highlight the novelties and contributions of our work.

Our intention was to highlight the following specific challenges for applying traditional GP-based SR to the **multi-scale complex systems**, which are the focus of our work.

- **Challenge 1: Computational efficiency for large number of state variables**. While parsimony pressure exists, abstracting truly concise and structurally meaningful mathematical expressions from a large number of state variables in such complex systems remains a significant hurdle. The challenge is not just about formula length, but also about efficiently identifying the correct, often sparse, underlying structure and meaningful interactions amidst high-dimensional observation data. Standard GP approaches, even with parsimony, can find it difficult to navigate this vast search space to converge on the most insightful and generalizable concise expressions without significant tuning or risk of overfitting to noise.
- **Challenge 2: Automatic discovery of higher-order mathematical relationships.** Due to the tree-based representation, it is impossible for traditional GP-based approaches to automatically discover higher-order mathematical relationships, such as summations or mappings over a large volume of elements (as often required in complex systems to describe collective behaviors or aggregate properties). While such operators can be included in the function set of GP, effectively *learning* their appropriate arguments, scope, and overall structure from data, especially when these structures are not known as *a priori* and involve many components, is challenging for standard tree-based representations. This often necessitates substantial prior knowledge to be encoded into the function set or searching strategies to guide the discovery of these specific complex patterns.

Our work aims to address these challenges and achieve automatic discovery of concise , interpretable, and higher-order symbolic structures that accurately capture multi-element interactions in multi-scale complex systems. To respond this comment, based on above discussion, we have revised the manuscript as follows.

Revised Manuscript:

> Unfortunately, no existing AI methods can achieve automatic discovery of multiscale deep formulas in complex systems. While methods like sparse regression can effectively learn mathematical patterns from large datasets [9, 22], their reliance on pre-defined functional forms limits the exploration of unknown regularities. Symbolic regression (SR), based on symbolic trees and genetic programming [2, 8, 9, 23, 24], is a powerful tool for automatically discovering mathematical formulas with unknown forms from data. However, traditional SR methods suffer from low success rates and computational efficiency for identifying complex relationships. Recently, substantial progress has been made in symbolic regression, which has greatly enhanced the discovery of laws in real-world data. For instance, physics-inspired symbolic regression explored to incorporate physical knowledge (e.g., high-order symmetries) as priors [25, 26]. Also, neural network-integrated SR discovers novel patterns from representations learned from deep models (e.g., GNNs) [9]. Besides, many studies have enabled symbolic regression to find complex relationships on benchmark datasets by improving the searching strategies with evolutionary algorithms [8, 27], reinforcement learning [28], or Monte Carlo tree search [26]. Nonetheless, without high-order representations and tailored design, the automatic discovery of the above studies can hardly work for multiscale complex systems.

Original Manuscript:

Unfortunately, no existing AI methods can achieve this. Fixed-form parametric models like sparse regression [9], provide us with remarkable successes in discovering state equations [10, 20], and governing equations [21] in various systems. However, they are inherently limited by their predefined form space, and therefore would fail in complex systems where prior knowledge of potential math patterns may not hold. Genetic programming [22], especially symbolic regression [1, 8, 9, 18] do not require prior knowledge, but fails to abstract concise math expressions from a large number of state variables in complex systems, due to their lack of compression and formula length reduction. Furthermore, their search space grows exponentially with the number of variables and expression depth [8, 18], making them computationally prohibitive to identify complex, nonlinear equations in such systems.

Comment 1.4:

Table 1 shows very basic equations, with expression three of up to 8 nodes (If I computed correctly) - this experiment does not align with the huge promise of a "very deep expression trees". Even AI Feynman (I) shows more complex examples, and it is 6 years old now...

Response to Comment 1.4:

We thank the reviewer for this comment. We recognize that the initial examples were of moderate complexity, even though some of them (e.g., Langevin, Navier-stokes equation, and Vector Navigation) have not been reported by other existing AI methods including AI Feynman. To demonstrate the capabilities of our method for deep expressions, we have added three new rules with higher complexity (up to 23 nodes) via conducting additional experiments. Please note that, the examples in AI Feynman have up to 21 nodes. As far as we know, and based on our experiments, no existing method can discover the newly added rule of Maxwell-Boltzmann distribution (MBD) with potential.

To respond this comment, we have conducted following modifications in the revised manuscript.

1. We have updated Table 1 with three additional rules, including examples with higher complexity.

Revised Manuscript (Page 21):

Table 1 Discovered expressions by Deflex in comparison with the ground truths

Rules		Ground truths and discovered by Deflex
Energy conservation	True	$E = v^2 \cdot m/kT$
	Discovered	$E = 0.992v^2 \cdot m/kT$
Momentum conservation	True	$\sum m_i \mathbf{v}_i = C$
	Discovered	$\sum m_i \mathbf{v}_i = C$
Maxwell-Boltzmann distribution (MBD)	True	$E = \frac{mv^2}{2k_B T}$
	Discovered	$E = \frac{0.511mv^2}{k_B T}$
MBD with potential	True	$E = \frac{mv^2}{2k_B T} + \sum_{j \neq i} \left(\|\vec{x_j} - \vec{x_i}\|^{-12} - 2\|\vec{x_j} - \vec{x_i}\|^{-6} \right)$
	Discovered	$E = \frac{0.506mv^2}{k_B T} + \sum_{j \neq i} \left(0.97\|\vec{x_j} - \vec{x_i}\|^{-12} - 1.89\|\vec{x_j} - \vec{x_i}\|^{-6} \right)$
Continuity of quality	True	$E = \|\nabla \cdot v\| \times \infty$
	Discovered	$E = \|\nabla \cdot v\| \times 10^{26}$
Newton viscosity law	True	$\tau_{ij} = -\mu \left(\frac{\partial u_i}{\partial x_j} + \frac{\partial u_j}{\partial x_i} \right)$
	Discovered	$\tau_{ij} = -1.037\mu \left(\frac{\partial u_i}{\partial x_j} + \frac{\partial u_j}{\partial x_i} \right)$
Velocity distribution	True	$E = \frac{1}{2}mv^2 + (\delta v)^p$
	Discovered	$E = 0.501mv^2 + (\delta v)^{3.15}$
Navier-Stokes equation	True	$E = \|\rho \left(\frac{\partial \mathbf{u}}{\partial t} + \mathbf{u} \cdot \nabla \mathbf{u} \right) + \nabla p - \mu \nabla^2 \mathbf{u}\| \times \infty$
	Discovered	$E = \|\rho \left(\frac{\partial \mathbf{u}}{\partial t} + \mathbf{u} \cdot \nabla \mathbf{u} \right) + \nabla p - \mu \nabla^2 \mathbf{u}\| \times 1.62 \times 10^{21}$
Langevin	True	$E = \|4.468 \times 10^{16} \ \mathrm{m}\dot{v} + v\|^2$
	Discovered	$E = \|4.438 \times 10^{16} m\dot{v} + 1.003v\|^2$
Levy flight (Human)	True	$E = D \log u$
	Discovered	$E = 2.37 \log u$
Levy flight (Avian)	True	$E = D \log u$
	Discovered	$E = 1.627 \log u$
Power law	True	$E = \alpha \log l + \gamma l$
	Discovered	$E = 1.56 \log l + 0.00027l$
Crowd power law	True	Undocumented
	Discovered	$E = 1.63 \log r_i - 0.037 \log r_i \sum_{j \neq i} \mathbb{I} \left(\left\| x_t^{(j)} - x_t^{(i)} \right\| < 2.46 \right)$

Original Manuscript:

Table 1 Discoverred rules compared with ground truth

Rule name	Ground truth	exploration
Energy conservation	$E = v^2 \cdot m/kT$	$E = 0.992v^2 \cdot m/kT^1$
Momentum conservation	$\sum m_i \mathbf{v}_i = C$	$\sum m_i \mathbf{v}_i = C$
Maxwell-Boltzmann distribution	$E = \frac{U(\vec{x})+\frac{1}{2}mv^2}{k_B T}$	$E = \frac{0.511mv^2}{k_B T}$
Continuity of quality [1]	$E = \Vert\nabla \cdot v\Vert \times \infty$	$E = \Vert\nabla \cdot v\Vert \times 10^26$
Newton viscosity law	$\tau_{ij} = -\mu \left(\frac{\partial u_i}{\partial x_j} + \frac{\partial u_j}{\partial x_i}\right)$	$\tau_{ij} = -1.037\mu \left(\frac{\partial u_i}{\partial x_j} + \frac{\partial u_j}{\partial x_i}\right)$
Velocity distribution	$E = \frac{1}{2}mv^2 + (\delta v)^p$	$E = 0.501mv^2 + (\delta v)^3.15$
Navier-Stokes equation	$E = \Vert\text{NSOperator}\mathbf{u}\Vert \times \infty$ [1]	$E = \Vert\text{NSOperator}\mathbf{u}\Vert \times 1.62 \times 10^21$
Langevin	$E = \Vert 4.468 \times 10^{16}\ \text{m}\dot{\boldsymbol{v}} + \boldsymbol{v}\Vert^2$	$E = \Vert 4.438 \times 10^{16} m\dot{v} + 1.003v\Vert^2$
Levy flight (Human)	$E = D \log u$	$E = 2.37 \log u$
Levy flight (Avian)	$E = D \log u$	$E = 1.627 \log u$
Power law	$E = \alpha \log l + \gamma l$	$E = 1.56 \log l + 0.00027l$

[1] Navier-Stokes equation

2. We have added Table 2 in the revised manuscript to summarize the discovery capabilities of the state-of-the-art methods in our experiments.

Revised Manuscript (Page 21)

Table 2 Comparison of methods on discovery capability

Rules	Deflex	Operon	SciMED	SINDy	gplearn	AI Feynman	PySR	DEAP
Energy conservation	✓	✓	✓	✓	✓	✓	✓	✓
Momentum conservation	✓	✓	✓	✓	✓	✓	✓	✓
MBD	✓	✓	✓	N.A.	✓	✓	✓	✓
MBD with potential	✓	N.A.	N.A.	N.A.	✗	N.A.	N.A.	✗
Continuity of quality	✓	✓	✓	✓	✓	✓	✓	✓
Newton viscosity law	✓	✓	✓	✓	✓	✓	✓	✓
Velocity distribution	✓	✗	✗	N.A.	✗	✓	✗	✗
Navier-Stokes equation	✓	✗	✗	✗	✗	✗	✗	✗
Langevin	✓	✗	✗	N.A.	✗	✗	✗	✗
Levy flight (Human)	✓	✗	✗	N.A.	✗	✓	✓	✓
Levy flight (Avian)	✓	✗	✗	N.A.	✗	✓	✓	✓
Power law	✓	✗	✗	N.A.	✗	✓	✗	✗
Crowd power law	✓	N.A.	N.A.	N.A.	✗	N.A.	N.A.	✗

✓ denotes a successful discovery, ✗ denotes a failure, and N.A. denotes an unsupported task (i.e., not applicable).

3. We have added corresponding analysis for both Tables 1 and 2 in the revised manuscript.

Revised Manuscript (Page 6-7):

As shown in Table 1, Deflex successfully identified a series of formulas in these particle motion systems, including the energy conservation, Boltzmann distribution, Maxwell-Boltzmann distribution (MBD), MBD with Lennard-Jones potential, and the Langevin equation. In particular, MBD with Lennard-Jones potential and the Langevin equation cannot be discovered by other compared methods in our experiments (shown in Table 2). And those tree-based symbolic regression method requires manual specification MBD with Lennard-Jones potential identified by Deflex in the dynamics of rare gas particles is a nonlinear equation that consists of up to 23 nodes, demonstrating Deflex's ability to discover complex nonlinear equations.

On the commonly identified Maxwell-Boltzmann distribution, Deflex managed to achieve the least NLL loss than the compared methods in both Argon and Pollen particles (shown in Fig. 4a). Meanwhile, the RMSEs of all compared methods are nearly the same on both energy conservation and momentum conservation in the two systems. This is because the structures of correctly identified formulas are almost identical in these methods, as mentioned before. As shown in Fig. 4b, Deflex incurs significantly less computational time than most of the comparatives. SINDy and Operon are quite close to or sometimes faster than Deflex, but only limited to some lower-order formulas like momentum conservation. This is due to their limitations in formula structures.

Original Manuscript:

As shown in Table 1, Deflex successfully identified the formulas of the energy conservation, Boltzmann distribution, and Maxwell-Boltzmann distribution in both particle motion systems. Langevin dynamic describes the interaction between matters at different scales and it does not hold for rare gas systems. And Deflex managed to manged to achieve higher accuracy than comparative methods, as shown in Fig. 4a. In contrast, PySR and AI Feynman could only discover momentum conservation due to the limited number of elements. Fig. 4b compares the computational effectiveness of Deflex with that of comparative methods in both systems. The computational time of Deflex was significantly lower than that of comparative methods for those formulas discovered in both systems.

Revised Manuscript (Page 7):

As presented in Table 1, Deflex identified multiple formulas, including power law equations [40], Lévy flight patterns [41], and vector navigation laws [42] in human crowds, and the Lévy flight patterns in bird migrations. Besides Deflex, AI Feynman, PySR, and DEAP can also discover the power law distribution and Lévy flight pattern, while only AI Feynman can discover the Power law distribution (shown in Table 2). But no other method can identify the vector navigation law. It is worth noting that Deflex also uncovered an undocumented power law distribution,

$$P\left(\Delta x_t^{(i)}\right) \propto r^{\alpha_0-\gamma \sum_{j\neq i} \mathbb{I}\left(\left\|x_t^{(j)}-x_t^{(i)}\right\|<2.46\right)}. \tag{2}$$

This rule may indicate a trend superimposed on the baseline power-law distribution and describe the collective behavior of human crowds: individuals exhibit a higher probability of making a long-distance move as their neighbors do.

Original Manuscript:

The probabilistic forms of the formulas discovered by Deflex are also presented in Table 1. Deflex successfully identified multiple formulas, including power law equations [30], Lévy flight patterns [31], and vector navigation laws [32], in human crowds and the Lévy flight patterns in bird migrations. Furthermore, we compared the NLL loss based on the real data and the MSE loss based on the parameter estimation of the known formulas. The results show that PySR was unable to identify these formulas (Fig. 4b). Although AI Feynman could uncover the normal version of the Lévy flight pattern, it incurs greater errors in either the NLL loss based on real data and the estimated known formulas (Fig. 4a).

Revised Manuscript (Page 8):

For scale-independent characteristics, Deflex demonstrates similar performance in water particles as on Argon and pollen particles, as shown in Table 1, Figs. 4a and 4b. To validate the cross-scale properties, we conducted coarse-graining of water

As shown in Fig. 4c, Deflex managed to capture the cross-scale emergence of Langevin dynamics in water particles. In contrast, other methods were limited to observing molecular dynamics at smaller scales and the dynamics of ice crystals at larger scales, failing to bridge the transition across scales. It also shows that Deflex detected the scale invariance of the system at the phase transition point of water (i.e., at 273.15K).

Original Manuscript:

As shown in Fig. 4, Deflex managed to capture the cross-scale emergence of Langevin dynamics in water particles. In contrast, other methods were limited to observing molecular dynamics at smaller scales and the dynamics of ice crystals at larger scales, failing to bridge the transition between these regimes. It is also shown that the Deflex detects the scale invariance of at phase transitions for water at 273.15K.

Revised Manuscript (Page 8):

As listed in Table 1, Deflex detected the Navier-Stokes equation of fluid dynamics through small-scale observations, which cannot be discovered by other methods. In larger-scale random observations, Deflex extracted the velocity distribution of the fluid within the vortex flow. These formulas correspond to different manifestations of long-standing laws in physics. For complex distribution formulas like velocity distribution, most compared methods are infeasible (shown in Table 2), and only AI Feynman and Deflex manage to discover.

Original Manuscript:

As shown in Table 1, the patterns we obtained correspond to different manifestations of long-standing laws applied in physics. Also shown in Fig.4a, we obtained the

Comment 1.5:

The comparison is with only AI Feynman and PySR. I would expect to see at least SINDy and SciMED and maybe gplearn and DEAP as baselines. You can have more options from the recent SRbenchmark paper.

Response to Comment 1.5:

We thank the reviewer for this comment, which can provide a comprehensive comparison to validate our method. We have added all the four suggested comparatives SINDy, SciMED, gplearn and DEAP, in the revised manuscript. Besides these suggested ones, we have also included Operon found by ourselves, which is one of the SOTA symbolic regression method.

SINDy is a sparse regression-based, while SciMED, gplearn, and DEAP are all tree-based symbolic regression methods with GP. Among them, DEAP and GPLearn are classic SR frameworks based on evolution algorithm, while SciMED is a recent physics-inspired method based on Monte Carlo tree searching. Operon has been one of the SOTA symbolic regression method according to SRBench. These are all relevant work to us, so we have followed the reviewer' suggestion to undertake a significant expansion of our experimental comparisons in the revised manuscript. However, since these baselines were not designed for multi-scale systems, the comparison experiment were conducted on single-scale tasks where the goal is to discovery the relationships between scale-specific variables. We believe the comparisons are extensive enough now.

To respond this comment, we have conducted the following modifications in the revised manuscript.

1. We have added detailed description of the compared methods in the revised manuscript.

Revised Manuscript (Page 4-5):

2.1 Compared Methods

We benchmarked our approach against several leading AI methods for formula discovery, including state-of-the-art SR method Operon [31], typical physics-inspired SR method SciMED [26], leading sparse regression method SINDy [22], and AI Feynman 2.0 [8], as well as classic SR methods gplearn [32], PySR [33] and DEAP [34] in terms of accuracy, efficiency, and adaptability to multiscale systems.

Different from Deflex with energy models and lambda calculus, existing AI methods (mainly based on symbolic regression) often support limited forms of problems and formulas. To maximize comparability, we made several accommodations for these compared methods. For the comparatives (PySR, AI Feynman, and DEAP) lacking native support for high-order operators (e.g., map or sum), we added these operators

to their symbol set manually. For the rest ones (Operon, SciMED, SINDy, and gplean) that are incompatible with these operators, we provided an alternative. That is, if such an operator was at the top level of the target formula, we applied the method for the sub-expressions only and then handled the top-level operators within the loss function manually. Similarly, for the methods unable to approximate distributions, we provided custom energy-based loss functions. A method was regarded as failing in a task if it still could not discover the rule after these best-effort accommodations.

Original Manuscript:

> We evaluate the effectiveness and efficiency of Deflex in six representative complex systems with different behaviors. Particularly, these systems can be categorized into the simulation experiments, including particle motion, two-phase water particles, and fluid dynamics, and the real-world data of human and bird collective movements. We compare the performance of Deflex with the state-of-the-art AI-based formula discovery algorithms, including AI Feynman 2.0 [8] and PySR [24], in terms of accuracy, efficiency, and adaptability to multi-scale systems.

2. We have updated Fig. 4 in the revised manuscript to include the comparison results with the new comparatives SINDy, SciMED, gplearn, DEAP, and Operon.

Revised Manuscript (Page 25):

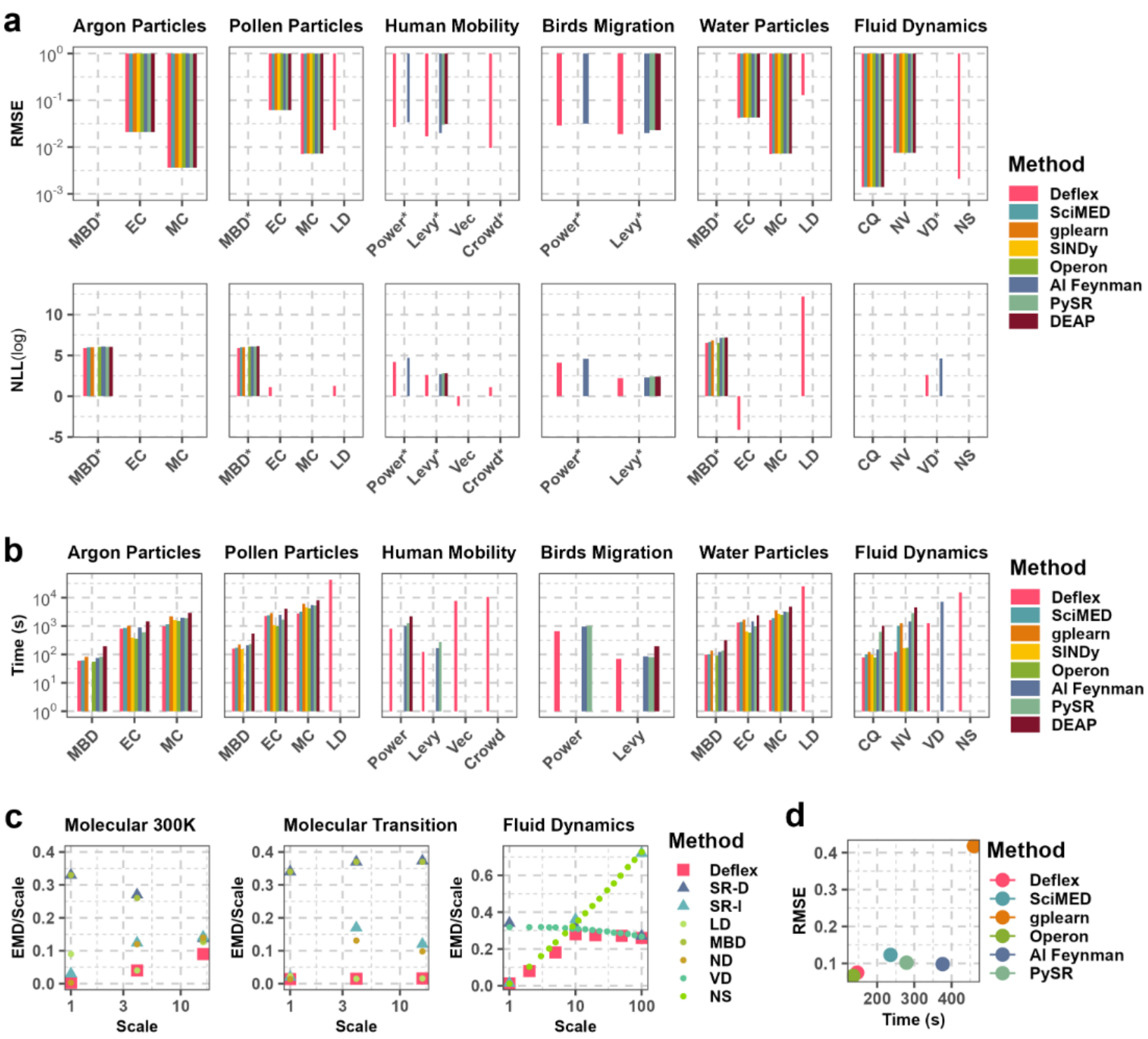

Fig. 4 Experimental results of Deflex in terms of accuracy (**a**), efficiency (**b**), and scalability (**c**). **a** compares the RMSE and NLL loss of the discovered formulas by Deflex and other methods in the experiments of particle motions, human and bird collective movement, water particles, and fluid dynamics. The abbreviations represent the Maxwell-Boltzmann distribution (MBD), energy conservation law (EC), momentum conservation (MC), Langevin dynamics (LD), continuity of quality(CQ), Newton viscosity law (NV), velocity distribution in fluid dynamics(VD), Navier-Stokes equation (NS), power law in human mobility (Power), Levy flight rule (Levy), vector navigation law (Vec), and crowd power law, respectively. Among them, the formulas in the form of distributions are marked with ∗. The absence of the method indicates that the corresponding metrics are not available for the method. **b** depicts the running time and usability of Deflex compared with other methods in different experiments. The horizontal axis represents the running time of the methods and the absence of the method indicates it is infeasible to discover the formula. Only Deflex can discover all the formulas in the experiments, and the time consumption of Deflex is significantly lower than that of most other methods for complex formulas. **c** shows the EMD values of known formulas and formulas discovered by Deflex and other methods across different coarse-graining scales for water particles' motion and fluid dynamics, where "SR-I" and "SR-D" represent the symbolic regression methods with invariant and distributional form settings, respectively. **d** shows the running time and relative mean square error for Deflex and other methods on the public benchmark dataset of Feynman Symbolic Regression Database.

Original Manuscript:

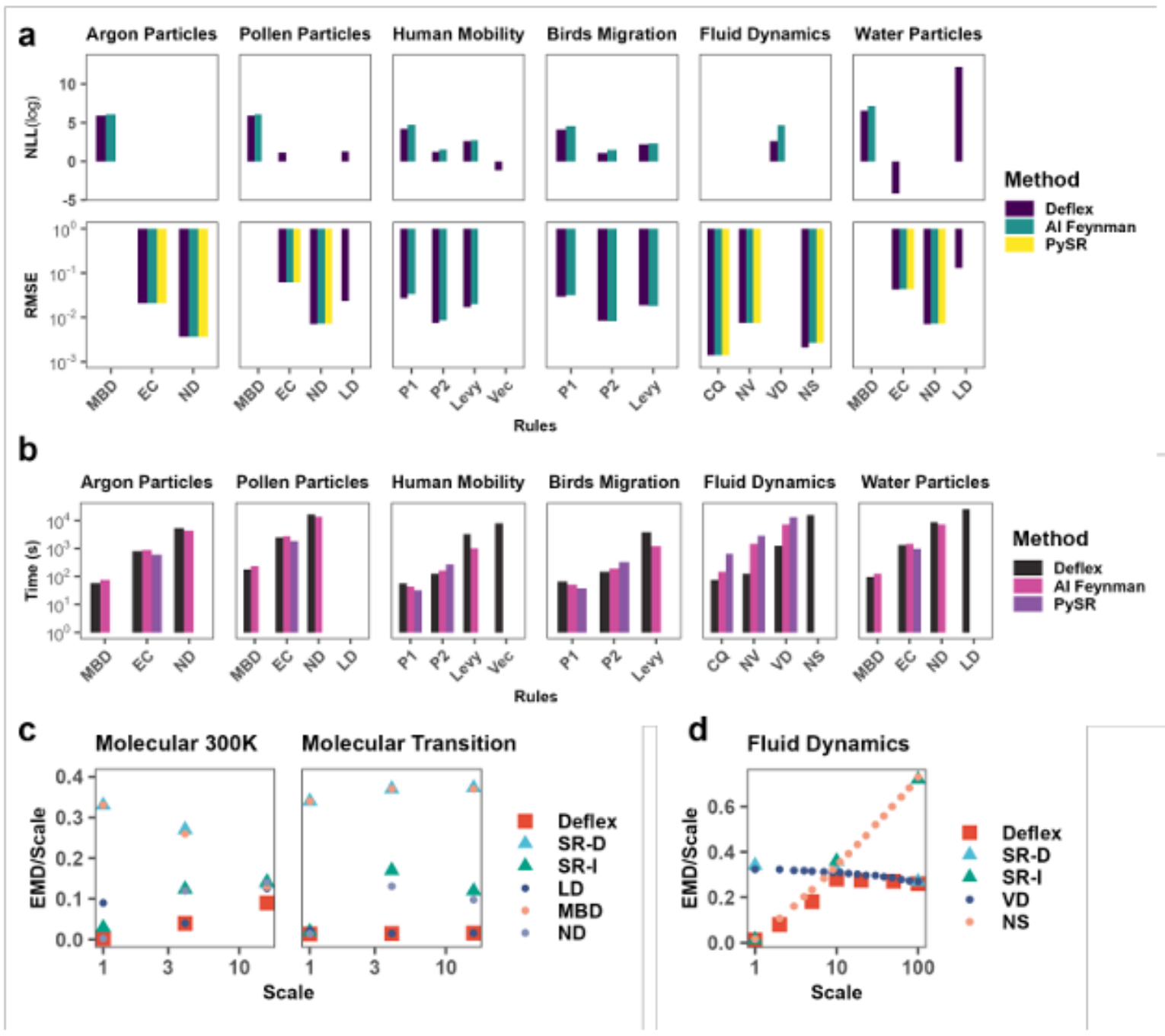

Fig. 4 Experimental results of Deflex in terms of accuracy (**a**), efficiency (**b**), and scalability (**c**). **a** compares the RMSE and NLL loss of the discovered formulas by Deflex and other methods in the experiments of particle motions, human and bird collective movement, fluid dynamics, and water particles. The abbreviations represent the Maxwell-Boltzmann distribution (MBD), energy conservation law (EC), Newtonian dynamics (ND), Langevin dynamics (LD), continuity of quality(CQ), Newton viscosity law(NV), velocity distribution in fluid dynamics(VD), Navier-Stokes equation (NS), first and second power law in human mobility(P1 and P2), Levy flight rule(Levy), and vector navigation law (Vec), respectively. The absence of the method indicates that the corresponding metrics are not available for the method. **b** depicts the running time and usability of Deflex compared with other methods in different experiments. The horizontal axis represents the running time of the methods and the absence of the method indicates it is infeasible to discover the formula. Only Deflex can discover all the formulas in the experiments, and the time consumption of Deflex is significantly lower than that of other methods for complex formulas. **c** and **d** shows the EMD values of known formulas and formulas discovered by Deflex and other methods across different coarse-graining scales for water particles motion (**c**) and fluid dynamics (**d**), where "SR-I" and "SR-D" represent the symbolic regression methods with invariant and distributional form settings, respectively.

3. We have added a new Table 2 to compare the capability of different methods for discovering the formulas in the six studied complex systems.

Revised Manuscript (Page 21):

Table 2 Comparison of methods on discovery capability

Rules	Deflex	Operon	SciMED	SINDy	gplearn	AI Feynman	PySR	DEAP
Energy conservation	✓	✓	✓	✓	✓	✓	✓	✓
Momentum conservation	✓	✓	✓	✓	✓	✓	✓	✓
MBD	✓	✓	✓	N.A.	✓	✓	✓	✓
MBD with potential	✓	N.A.	N.A.	N.A.	✗	N.A.	N.A.	✗
Continuity of quality	✓	✓	✓	✓	✓	✓	✓	✓
Newton viscosity law	✓	✓	✓	✓	✓	✓	✓	✓
Velocity distribution	✓	✗	✗	N.A.	✗	✓	✗	✗
Navier-Stokes equation	✓	✗	✗	✗	✗	✗	✗	✗
Langevin	✓	✗	✗	N.A.	✗	✗	✗	✗
Levy flight (Human)	✓	✗	✗	N.A.	✗	✓	✓	✓
Levy flight (Avian)	✓	✗	✗	N.A.	✗	✓	✓	✓
Power law	✓	✗	✗	N.A.	✗	✓	✗	✗
Crowd power law	✓	N.A.	N.A.	N.A.	✗	N.A.	N.A.	✗

✓ denotes a successful discovery, ✗ denotes a failure, and N.A. denotes an unsupported task (i.e., not applicable).

4. We have revised multiple places to analyze the new comparison results in Section 2 in the revised manuscript.

Revised Manuscript (Page 6-7):

As shown in Table 1, Deflex successfully identified a series of formulas in these particle motion systems, including the energy conservation, Boltzmann distribution, Maxwell-Boltzmann distribution (MBD), MBD with Lennard-Jones potential, and the Langevin equation. In particular, MBD with Lennard-Jones potential and the Langevin equation cannot be discovered by other compared methods in our experiments (shown in Table 2). And those tree-based symbolic regression method requires manual specification MBD with Lennard-Jones potential identified by Deflex in the dynamics of rare gas particles is a nonlinear equation that consists of up to 23 nodes, demonstrating Deflex's ability to discover complex nonlinear equations.

On the commonly identified Maxwell-Boltzmann distribution, Deflex managed to achieve the least NLL loss than the compared methods in both Argon and Pollen particles (shown in Fig. 4a). Meanwhile, the RMSEs of all compared methods are nearly the same on both energy conservation and momentum conservation in the two systems. This is because the structures of correctly identified formulas are almost identical in these methods, as mentioned before. As shown in Fig. 4b, Deflex incurs significantly less computational time than most of the comparatives. SINDy and Operon are quite close to or sometimes faster than Deflex, but only limited to some lower-order formulas like momentum conservation. This is due to their limitations in formula structures.

Original Manuscript:

As shown in Table 1, Deflex successfully identified the formulas of the energy conservation, Boltzmann distribution, and Maxwell-Boltzmann distribution in both particle motion systems. Langevin dynamic describes the interaction between matters at different scales and it does not hold for rare gas systems. And Deflex managed to manged to achieve higher accuracy than comparative methods, as shown in Fig. 4a. In contrast, PySR and AI Feynman could only discover momentum conservation due to the limited number of elements. Fig. 4b compares the computational effectiveness of Deflex with that of comparative methods in both systems. The computational time of Deflex was significantly lower than that of comparative methods for those formulas discovered in both systems.

Revised Manuscript (Page 7-8):

As presented in Table 1, Deflex identified multiple formulas, including power law equations [40], Lévy flight patterns [41], and vector navigation laws [42] in human crowds, and the Lévy flight patterns in bird migrations. Besides Deflex, AI Feynman, PySR, and DEAP can also discover the power law distribution and Lévy flight pattern, while only AI Feynman can discover the Power law distribution (shown in Table 2). But no other method can identify the vector navigation law. It is worth noting that Deflex also uncovered an undocumented power law distribution,

$$P\left(\Delta x_t^{(i)}\right) \propto r^{\alpha_0-\gamma\sum_{j\neq i}\mathbb{I}\left(\left\|x_t^{(j)}-x_t^{(i)}\right\|<2.46\right)}. \tag{2}$$

This rule may indicate a trend superimposed on the baseline power-law distribution and describe the collective behavior of human crowds: individuals exhibit a higher probability of making a long-distance move as their neighbors do.

For the commonly found rules of the power law distribution and Lévy flight pattern, we first compared the NLL loss and RMSE of Deflex with other methods in Fig. 4a. It shows that Deflex's discovered distributions have consistently high accuracy (both smaller NLL loss and RMSE value) than other capable methods. We also compared their time efficiency for discovering these distributions under the same accuracy (i.e., by setting an identical precision threshold for convergence). Fig. 4b shows that Deflex also leads to the least running time among the compared methods.

Original Manuscript:

The probabilistic forms of the formulas discovered by Deflex are also presented in Table 1. Deflex successfully identified multiple formulas, including power law equations [30], Lévy flight patterns [31], and vector navigation laws [32], in human crowds and the Lévy flight patterns in bird migrations. Furthermore, we compared the NLL loss based on the real data and the MSE loss based on the parameter estimation of the known formulas. The results show that PySR was unable to identify these formulas (Fig. 4b). Although AI Feynman could uncover the normal version of the Lévy flight pattern, it incurs greater errors in either the NLL loss based on real data and the estimated known formulas (Fig. 4a).

Revised Manuscript (Page 8):

For scale-independent characteristics, Deflex demonstrates similar performance in water particles as on Argon and pollen particles, as shown in Table 1, Figs. 4a and 4b. To validate the cross-scale properties, we conducted coarse-graining of water

As shown in Fig. 4c, Deflex managed to capture the cross-scale emergence of Langevin dynamics in water particles. In contrast, other methods were limited to observing molecular dynamics at smaller scales and the dynamics of ice crystals at larger scales, failing to bridge the transition across scales. It also shows that Deflex detected the scale invariance of the system at the phase transition point of water (i.e., at 273.15K).

Original Manuscript:

As shown in Fig. 4, Deflex managed to capture the cross-scale emergence of Langevin dynamics in water particles. In contrast, other methods were limited to observing molecular dynamics at smaller scales and the dynamics of ice crystals at larger scales, failing to bridge the transition between these regimes. It is also shown that the Deflex detects the scale invariance of at phase transitions for water at 273.15K.

Revised Manuscript (Page 8-9):

As listed in Table 1, Deflex detected the Navier-Stokes equation of fluid dynamics through small-scale observations, which cannot be discovered by other methods. In larger-scale random observations, Deflex extracted the velocity distribution of the fluid within the vortex flow. These formulas correspond to different manifestations of long-standing laws in physics. For complex distribution formulas like velocity distribution, most compared methods are infeasible (shown in Table 2), and only AI Feynman and Deflex manage to discover.

Fig.4a also shows that the equation-based formulas discovered by Deflex can achieve a similar RMSE as other methods. For the velocity distribution, Deflex's discovery has a much smaller NLL loss than AI Feynman's, showing its better accuracy. In Fig. 4b, most comparatives, except for SINDy and Operon, become much slower than Deflex with the increase of formula complexity. Among them, AI Feynman is around 10 times slower than Deflex.

In addition, we compared the EMD across different coarse-graining scales of Deflex with that of AI Feynman and the renormalization of known formulas. Fig. 4c shows that predictions from Deflex kept the lowest EMD across different scales, indicating the Deflex's ability to overcome the scale gap issue in complex systems. Meanwhile, other methods with a single setting could only capture the patterns at a single scale, and the EMD increased significantly when the scale changed.

Original Manuscript:

As shown in Table 1, the patterns we obtained correspond to different manifestations of long-standing laws applied in physics. Also shown in Fig.4a, we obtained the Navier-Stokes equation of fluid dynamics through small-scale observations. In larger-scale random observations, we acquired information such as the velocity distribution of the fluid within the vortex flow. Fig. 4b shows the time consumption of different methods. When the complexity of the formulas increases, the PySR and AI Feynman methods became much slower than Deflex (approximately 10× slower for velocity distribution). For very complex formulas including Navier-Stokes equation and Langevin dynamics for particle motion, the PySR and AI Feynman methods became infeasible.

In addition, we compared the EMD across different coarse-graining scales of Deflex with that of AI Feynman and the renormalization of known formulas. Fig. 4d shows that predictions from Deflex kept the lowest EMD across different scales, indicating the Deflex's ability to overcome the scale gap issue in complex systems. Meanwhile, other methods with a single setting could only capture the patterns at a single scale, and the EMD increased significantly when the scale changes.

5. We have further added a comparison experiment on a benchmark dataset as follows (Please refer to **Response to Comment 3.2** for more details).

Revised Manuscript (Page 9):

2.7 Comparison on Benchmark Dataset

We finally evaluated Deflex on the public benchmark dataset of Feynman Symbolic Regression Database [24]. This benchmark dataset is generated by sampling from 120 classical formulas spanning different fields of physics, with added noise to simulate real-world conditions. It captures essential characteristics of real-world mathematical formulas, such as symmetry, while also being a classic, well-established set of problems for symbolic regression. This dual characteristic facilitates a feasible performance comparison against other SR-based AI methods. In the experiment, Deflex was compared against Operon, SciMED, gplearn, AI Feynman 2.0, and PySR. We compared their performance by measuring the trade-off between the average RMSE of discovered formulas and the total runtime for the discovery. Note that SINDy was not included as it is sparse regression-based and not designed for symbolic regression tasks, while DEAP was omitted since it works as the backend of gplearn in the current task.

As shown in Fig. 4d, Deflex demonstrates a quite competitive performance among all compared methods on the benchmark dataset in terms of the accuracy-efficiency tradeoff, although it is not specialized for these traditional, closed-form equations. In particular, Deflex is only slightly outperformed by Operon, which relies on a fixed symbol set and is optimized for runtime. In our experiment, Deflex managed to discover the formula with an RMSE of 0.075 in just 146.2 seconds, which was quite close Operon with an RMSE of 0.0663 in 135.8 seconds. And Deflex and Operon showed significant advantages over other methods.

6. We have also added the implementation details of the additional comparatives in the revised supplementary material as follows.

Revised SI:

Our set of baseline frameworks for comparison comprises Operon, SciMED, SINDy, gplearn, AI Feynman 2.0, PySR, and DEAP. These frameworks have distinct capabilities and limitations:

- Only AI Feynman 2.0 has built-in support for distribution fitting.
- SINDy operates based on sparse regression rather than symbolic trees.
- The APIs for Operon and SciMED do not permit the addition of custom symbols.
- Most frameworks, with the exception of DEAP and parts of gplearn based on it, do not support abstract data types for symbolic nodes, which precludes the use of high-order functions like map.
- None of these frameworks include high-order operators natively.

To ensure maximum comparability across these diverse frameworks, we implemented a series of adaptations. For frameworks lacking native high-order operators (e.g., map, sum), we first attempted to inject these operators into their symbol sets. If a framework could not incorporate these operators, and the target expression used them at the top level, for instance, a formula of the form `result = sum(map(f, data))`, we adapted the task. The framework was made responsible for fitting only the underlying sub-expression (f), and the top-level operators were then applied manually by us within the loss function to evaluate the final error. Similarly, for tasks involving distribution fitting, we provided a custom Negative Log-Likelihood (NLL) loss function for frameworks lacking this capability.

Comment 1.6:
"Deflex can also reveal undocumented system relationships from the observation data." - not sure I understand from the results section how you proved this point.

Response to Comment 1.6:
We thank the reviewer for pointing out this, and we have included evidences on undocumented relationships in the revised manuscript.

We believe that novel discovery of undocumented system relationship is essential for AI-based methods for scientific exploration. However, current AI methods have always been validated on whether they can recover known equations from some benchmark datasets. Although this is a standard practice for the performance evaluation, we believe it is far from enough to evaluate the practical use for scientific analysis in real world. In our experiments, we found that several Deflex's discovered relationships were undocumented but could well fit on the observed data and imply some evident patterns. For instance, in the human mobility experiment, Deflex outputs a rule in the form of distribution, which is similar to power-law distribution and has very lower NLL or RMSE loss. It implies an evident pattern by empirically observing the original data: individuals will be more likely to have a long-distance move as their neighbors do. This is empirically reasonable and justifiable. Therefore, we believe it might be interesting to report the result and highlight the flexible discovery capability of our method. Of course, it would be still an important future work to explore their practical meaning with the related experts in that area.

To respond this comment, we have conducted the following modifications in the revised manuscript.

1. We have included a specific example of undocumented formulas discovered by Deflex in Table 1 and added justification of the formula in the revised manuscript.

Revised Manuscript (Page 7, 21):

Table 1 Discovered expressions by Deflex in comparison with the ground truths

Rules		Ground truths and discovered by Deflex
Energy conservation	True	$E = v^2 \cdot m/kT$
	Discovered	$E = 0.992v^2 \cdot m/kT$
Momentum conservation	True	$\sum m_i \mathbf{v}_i = C$
	Discovered	$\sum m_i \mathbf{v}_i = C$
Maxwell-Boltzmann distribution (MBD)	True	$E = \frac{mv^2}{2k_BT}$
	Discovered	$E = \frac{0.511mv^2}{k_BT}$
MBD with potential	True	$E = \frac{mv^2}{2k_BT} + \sum_{j \neq i} \left(\|\vec{x_j} - \vec{x_i}\|^{-12} - 2\|\vec{x_j} - \vec{x_i}\|^{-6} \right)$
	Discovered	$E = \frac{0.506mv^2}{k_BT} + \sum_{j \neq i} \left(0.97\|\vec{x_j} - \vec{x_i}\|^{-12} - 1.89\|\vec{x_j} - \vec{x_i}\|^{-6} \right)$
Continuity of quality	True	$E = \|\nabla \cdot v\| \times \infty$
	Discovered	$E = \|\nabla \cdot v\| \times 10^{26}$
Newton viscosity law	True	$\tau_{ij} = -\mu \left(\frac{\partial u_i}{\partial x_j} + \frac{\partial u_j}{\partial x_i} \right)$
	Discovered	$\tau_{ij} = -1.037\mu \left(\frac{\partial u_i}{\partial x_j} + \frac{\partial u_j}{\partial x_i} \right)$
Velocity distribution	True	$E = \frac{1}{2}mv^2 + (\delta v)^p$
	Discovered	$E = 0.501mv^2 + (\delta v)^{3.15}$
Navier-Stokes equation	True	$E = \|\rho\left(\frac{\partial \mathbf{u}}{\partial t} + \mathbf{u} \cdot \nabla \mathbf{u}\right) + \nabla p - \mu \nabla^2 \mathbf{u}\| \times \infty$
	Discovered	$E = \|\rho\left(\frac{\partial \mathbf{u}}{\partial t} + \mathbf{u} \cdot \nabla \mathbf{u}\right) + \nabla p - \mu \nabla^2 \mathbf{u}\| \times 1.62 \times 10^{21}$
Langevin	True	$E = \|4.468 \times 10^{16}\ \mathbf{m}\dot{v} + v\|^2$
	Discovered	$E = \|4.438 \times 10^{16} m\dot{v} + 1.003v\|^2$
Levy flight (Human)	True	$E = D \log u$
	Discovered	$E = 2.37 \log u$
Levy flight (Avian)	True	$E = D \log u$
	Discovered	$E = 1.627 \log u$
Power law	True	$E = \alpha \log l + \gamma l$
	Discovered	$E = 1.56 \log l + 0.00027l$
Crowd power law	True	Undocumented
	Discovered	$E = 1.63 \log r_i - 0.037 \log r_i \sum_{j \neq i} \mathbb{I}\left(\left\| x_t^{(j)} - x_t^{(i)} \right\| < 2.46 \right)$

As presented in Table 1, Deflex identified multiple formulas, including power law equations [40], Lévy flight patterns [41], and vector navigation laws [42] in human crowds, and the Lévy flight patterns in bird migrations. Besides Deflex, AI Feynman, PySR, and DEAP can also discover the power law distribution and Lévy flight pattern, while only AI Feynman can discover the Power law distribution (shown in Table 2). But no other method can identify the vector navigation law. It is worth noting that Deflex also uncovered an undocumented power law distribution,

$$P\left(\Delta x_t^{(i)}\right) \propto r^{\alpha_0 - \gamma \sum_{j \neq i} \mathbb{I}\left(\left\| x_t^{(j)} - x_t^{(i)} \right\| < 2.46 \right)}. \tag{2}$$

This rule may indicate a trend superimposed on the baseline power-law distribution and describe the collective behavior of human crowds: individuals exhibit a higher probability of making a long-distance move as their neighbors do.

Original Manuscript:

Table 1 Discoverred rules compared with ground truth

Rule name	Ground truth	exploration
Energy conservation	$E = v^2 \cdot m/kT$	$E = 0.992v^2 \cdot m/kT^1$
Momentum conservation	$\sum m_i \mathbf{v}_i = C$	$\sum m_i \mathbf{v}_i = C$
Maxwell-Boltzmann distribution	$E = \frac{U(\vec{x}) + \frac{1}{2}mv^2}{k_BT}$	$E = \frac{0.511mv^2}{k_BT}$
Continuity of quality [1]	$E = \|\nabla \cdot v\| \times \infty$	$E = \|\nabla \cdot v\| \times 10^26$
Newton viscosity law	$\tau_{ij} = -\mu \left(\frac{\partial u_i}{\partial x_j} + \frac{\partial u_j}{\partial x_i} \right)$	$\tau_{ij} = -1.037\mu \left(\frac{\partial u_i}{\partial x_j} + \frac{\partial u_j}{\partial x_i} \right)$
Velocity distribution	$E = \frac{1}{2}mv^2 + (\delta v)^p$	$E = 0.501mv^2 + (\delta v)^3.15$
Navier-Stokes equation	$E = \|\text{NSOperator}\mathbf{u}\| \times \infty$ [1]	$E = \|\text{NSOperator}\mathbf{u}\| \times 1.62 \times 10^21$
Langevin	$E = \|4.468 \times 10^{16}\ \mathbf{m}\dot{v} + \mathbf{v}\|^2$	$E = \|4.438 \times 10^{16} m\dot{v} + 1.003v\|^2$
Levy flight (Human)	$E = D \log u$	$E = 2.37 \log u$
Levy flight (Avian)	$E = D \log u$	$E = 1.627 \log u$
Power law	$E = \alpha \log l + \gamma l$	$E = 1.56 \log l + 0.00027l$

[1]Navier-Stokes equation

2. We have highlighted the necessities to explore the practical meaning with area experts in Section 3 “Discussion”.

Revised Manuscript (Page 10):

> In this work, we have presented Deflex, a framework for discovering governing equations from multiscale complex systems. Our empirical results demonstrate its ability to identify symbolic rules across different scales and, in some cases, reveal previously undocumented relationships from observational data. The proposed methodology for representing interactions among high-dimensional entities suggests potential applicability to domains beyond those tested in our experiments. Future work could explore the practical meaning of the undocumented formulas with area experts, and Deflex’s further use in areas such as atmospheric dynamics modeling [20] and materials science [44], particularly for systems where strong scale interdependencies pose challenges to conventional methods. We hope that this work serves as a step toward more capable automated scientific discovery in a range of disciplines.

Original Manuscript:

> In conclusion, Deflex offers a reliable and efficient AI method for the automatic discovery of multi-scale rules from complex systems. It enables deep formula discovery across massive elements and multiple scales and may uncover unknown rules in complex systems. Its methodology for handling the interactions among high-dimensional entities suggests potential generalization to areas beyond our experiments. Possible application domains include atmospheric dynamics modeling [34] and materials science [35], particularly for systems with strong scale interdependencies that challenge conventional analytical approaches. We hope that our work could be a useful tool for scientific discovery in many distinct disciplines.

Comment 1.7:

"Compared with state-of-the-art, Deflex achieves superior performance in both accuracy" - From Fig. 4 it seems Deflex does not outperform PySR or Ai Feynman in both NLL and RMSE.

Response to Comment 1.7:

We thank the reviewer for this comment. We agree that Deflex outperforms existing methods not in all cases, but in most cases.

First of all, we would like to clarify how we quantitively compare Deflex’s performance with other methods. Specifically, we consider two cases separately based on the different forms of formulas.

- For the formulas in the form of equations and invariants, we compare their fidelity by measuring the relative mean square error (RMSE) between the prediction and the true value based on the identified formulas. Note that, for these kinds of formulas, there is almost no structural difference (except for residual terms with negligible errors) in the commonly identified formulas by multiple methods, given the same convergence constraint. And the differences in RMSE are too small to have statistical significance.
- For the formulas in the form of distributions, there often can be differences in the commonly identified formulas, even under the same convergence constraint. Therefore, we used the NLL loss to measure the likelihood of the distribution fitting real data. Note that, we also reported RMSE to measure the fitting error of simple distributions for a reference.

Next, based on above clarification of the metrics on different forms of formulas, we would like to further clarify our experimental results (i.e., Fig. 4) in the original manuscript.

- Regarding the comparing results in terms of the NLL loss (on the commonly identified

distributions), Deflex outperforms the feasible method AI Feynman, shown in Fig. 4a in the original manuscript.

- Regarding the comparing results in terms of RMSE, we also analyze the results separately according the formula forms. For the equations and invariants, Deflex has almost the same or even smaller RMSE than AI Feynman and PySR, where the differences are mainly irreducible errors and not significant. For the simple-formed distributions (e.g., P1, P2, Levy in human mobility and birds migration), Deflex achieves smaller RMSE than AI Feynman in most cases. One exception is the Levy distribution in birds migration, which however was also due to the irreducible error according to our analysis.

Therefore, our method Deflex in general achieves better (in most cases) or at least comparable accuracy than PySR and AI Feynman, in the original manuscript.

Then, considering the reviewers' insightful comments and valuable suggestions for more comprehensive, we have conducted more extensive experiments to include additional comparisons and new datasets. In particular, we have added FIVE additional state-of-the-art methods and an international benchmark dataset. The new experimental results are presented in the updated Fig. 4 in the revised manuscript, which are also consistently with those in the original manuscript. Specifically,

- Regarding the comparing results in terms of the NLL loss (on the commonly identified distributions), Deflex outperforms all the feasible methods, shown in Fig. 4a in the revised manuscript.
- Regarding the comparing results in terms of RMSE, Deflex still has comparable or slightly smaller RMSE than other methods, given the same convergence threshold. This applies for equations/invariants and simple distributions.

Besides above results on accuracy, we have also compared the runtime efficiency of different methods for finding the formulas with the same accuracy. As shown in Fig. 4b, Deflex in general costs less runtime on the commonly identified formulas in most cases. In addition, on the newly added benchmark dataset, we compared the accuracy-efficiency tradeoff of different methods in Fig. 4d. As shown, Deflex significantly outperforms other 4 methods except Operon. The difference with Operon is also quite marginal. Therefore, we believe it still holds for the conclusion that, Deflex in general achieves better (in most cases) performance than the compared methods.

Finally, to respond this comment, based on above discussion, we have conducted more experiments, and extensive modifications in the revised manuscript to ensure the clarity of performance evaluation, which are summarized as follows.

1. We have revised the statement pointed by the reviewers and corresponding paragraphs to better summarize the performance of Deflex.

Revised Manuscript (Page 10):

> Experimental results demonstrate that Deflex can manage to overcome the scale gap issue by achieving the least differences across scales. Compared with state-of-the-art single-scale methods, it can also achieve better performance in both accuracy and computational efficiency in most cases, where the runtime reduction can achieve up to orders of magnitude. In addition to reconstructing established principles (e.g., Langevin equations, energy conservation), Deflex also shows the potential to reveal undocumented system relationships from the observed data.

Original Manuscript:

> Experimental results manifest that, besides being able to reconstruct the established principles (e.g., Langevin equations, energy conservation), Deflex can also reveal undocumented system relationships from the observation data. Compared with state-of-the-art, Deflex achieves superior performance in both accuracy and computational efficiency, which can reduce the learning time by orders of magnitude. Besides, it fills the gap of existing methods by realizing automatic multi-scale formula discovery.

2. We have revised the manuscript to clarify the experimental setup, including the compared methods and performance metrics.

Revised Manuscript (Page 4-5):

2.1 Compared Methods

We benchmarked our approach against several leading AI methods for formula discovery, including state-of-the-art SR method Operon [31], typical physics-inspired SR method SciMED [26], leading sparse regression method SINDy [22], and AI Feynman 2.0 [8], as well as classic SR methods gplearn [32], PySR [33] and DEAP [34] in terms of accuracy, efficiency, and adaptability to multiscale systems.

Different from Deflex with energy models and lambda calculus, existing AI methods (mainly based on symbolic regression) often support limited forms of problems and formulas. To maximize comparability, we made several accommodations for these compared methods. For the comparatives (PySR, AI Feynman, and DEAP) lacking native support for high-order operators (e.g., map or sum), we added these operators to their symbol set manually. For the rest ones (Operon, SciMED, SINDy, and gplean) that are incompatible with these operators, we provided an alternative. That is, if such an operator was at the top level of the target formula, we applied the method for the sub-expressions only and then handled the top-level operators within the loss function manually. Similarly, for the methods unable to approximate distributions, we provided custom energy-based loss functions. A method was regarded as failing in a task if it still could not discover the rule after these best-effort accommodations.

Original Manuscript:

> fluid dynamics, and the real-world data of human and bird collective movements. We compare the performance of Deflex with the state-of-the-art AI-based formula discovery algorithms, including AI Feynman 2.0 [8] and PySR [24], in terms of accuracy, efficiency, and adaptability to multi-scale systems.

Revised Manuscript (Page 5):

For established or empirical formulas, we first qualitatively assess whether the structure and parameters of the discovered formulas align with the ground truth. The formulas of invariants are considered successful recovery if they can be expressed as the correct formula with an additional stochastic residual term. Table 1 lists all the discovered formulas by Deflex in the studied six systems, compared to the ground truths. It should be noted that, for those undocumented discoveries, we provide justifications for their possible meanings in the real world. For a reference, we also summarize the capability of the compared methods for discovering these formulas in Table 2.

To quantitatively evaluate the accuracy of discovered formulas, we consider two cases based on the different forms of formulas separately.

- For the formulas in the form of equations and invariants, we employ the relative mean square error (RMSE) between the prediction and the true value. Specifically, all compared methods are given the same convergence threshold. Note that, in such a case, the RMSEs of different methods are hardly comparable since there is almost no structural difference (except for residual terms with negligible errors) in the commonly identified formulas in these forms.

- For distribution-based formulas, we use the NLL loss defined in Eq. (5) in the method section, which measures the likelihood of the distribution fitting real data. The feasible methods were also set with the same convergence constraint. Note that we also report the RMSE to measure the fitting error of simple distributions for reference.

Fig 4a compares the NLL loss of Deflex's distribution discovery in comparison with other methods, and reports the RMSE on the commonly identified equations and invariants.

Original Manuscript:

Since uncovering formulas within a complex system may represent an open-ended challenge, we incorporate qualitative assessments, basic quantitative analyses, and renormalization and coarse-graining techniques [25] in the performance evaluation.

For systems with established or empirical formulas, we qualitatively assess whether the structure and parameters of the discovered formulas align with the ground truth. The formulas of invariants are considered successful recovery if they can be expressed as the correct formula with an additional stochastic residual term. To assess the accuracy and distribution fidelity of invariant formulas, besides the NLL loss defined in Eq. (5) that measures the likelihood of the model fitting with real data, we employ the relative mean square error (MSE) measure the squared absolute error between the prediction and the true value,

$$RMSE(f) = \frac{\sqrt{\frac{1}{N}\sum_{i\in I}\left(f(x_i) - y_i\right)^2}}{\max_{i\in I} y_i}, \tag{1}$$

where I and N is the index set and number of data points, $f(x_i)$ is the predicted value, and y_i is the true value. Since the formulas are represented as distributions, we

3. We have also revised the experimental results.

Revised Manuscript (Page 6-7):

As shown in Table 1, Deflex successfully identified a series of formulas in these particle motion systems, including the energy conservation, Boltzmann distribution, Maxwell-Boltzmann distribution (MBD), MBD with Lennard-Jones potential, and the Langevin equation. In particular, MBD with Lennard-Jones potential and the Langevin equation cannot be discovered by other compared methods in our experiments (shown in Table 2). And those tree-based symbolic regression method requires manual specification MBD with Lennard-Jones potential identified by Deflex in the dynamics of rare gas particles is a nonlinear equation that consists of up to 23 nodes, demonstrating Deflex's ability to discover complex nonlinear equations.

On the commonly identified Maxwell-Boltzmann distribution, Deflex managed to achieve the least NLL loss than the compared methods in both Argon and Pollen particles (shown in Fig. 4a). Meanwhile, the RMSEs of all compared methods are nearly the same on both energy conservation and momentum conservation in the two systems. This is because the structures of correctly identified formulas are almost identical in these methods, as mentioned before. As shown in Fig. 4b, Deflex incurs significantly less computational time than most of the comparatives. SINDy and Operon are quite close to or sometimes faster than Deflex, but only limited to some lower-order formulas like momentum conservation. This is due to their limitations in formula structures.

Original Manuscript:

As shown in Table 1, Deflex successfully identified the formulas of the energy conservation, Boltzmann distribution, and Maxwell-Boltzmann distribution in both particle motion systems. Langevin dynamic describes the interaction between matters at different scales and it does not hold for rare gas systems. And Deflex managed to manged to achieve higher accuracy than comparative methods, as shown in Fig. 4a. In contrast, PySR and AI Feynman could only discover momentum conservation due to the limited number of elements. Fig. 4b compares the computational effectiveness of Deflex with that of comparative methods in both systems. The computational time of Deflex was significantly lower than that of comparative methods for those formulas discovered in both systems.

Revised Manuscript (Page 7-8):

As presented in Table 1, Deflex identified multiple formulas, including power law equations [40], Lévy flight patterns [41], and vector navigation laws [42] in human crowds, and the Lévy flight patterns in bird migrations. Besides Deflex, AI Feynman, PySR, and DEAP can also discover the power law distribution and Lévy flight pattern, while only AI Feynman can discover the Power law distribution (shown in Table 2). But no other method can identify the vector navigation law. It is worth noting that Deflex also uncovered an undocumented power law distribution,

$$P\left(\Delta x_t^{(i)}\right) \propto r^{\alpha_0-\gamma \sum_{j\neq i} \mathbb{I}\left(\left\|x_t^{(j)}-x_t^{(i)}\right\|<2.46\right)}. \qquad (2)$$

This rule may indicate a trend superimposed on the baseline power-law distribution and describe the collective behavior of human crowds: individuals exhibit a higher probability of making a long-distance move as their neighbors do.

For the commonly found rules of the power law distribution and Lévy flight pattern, we first compared the NLL loss and RMSE of Deflex with other methods in Fig. 4a. It shows that Deflex's discovered distributions have consistently high accuracy (both smaller NLL loss and RMSE value) than other capable methods. We also compared their time efficiency for discovering these distributions under the same accuracy

(i.e., by setting an identical precision threshold for convergence). Fig. 4b shows that Deflex also leads to the least running time among the compared methods.

Original Manuscript:

The probabilistic forms of the formulas discovered by Deflex are also presented in Table 1. Deflex successfully identified multiple formulas, including power law equations [30], Lévy flight patterns [31], and vector navigation laws [32], in human crowds and the Lévy flight patterns in bird migrations. Furthermore, we compared the NLL loss based on the real data and the MSE loss based on the parameter estimation of the known formulas. The results show that PySR was unable to identify these formulas (Fig. 4b). Although AI Feynman could uncover the normal version of the Lévy flight pattern, it incurs greater errors in either the NLL loss based on real data and the estimated known formulas (Fig. 4a).

Revised Manuscript (Page 8):

For scale-independent characteristics, Deflex demonstrates similar performance in water particles as on Argon and pollen particles, as shown in Table 1, Figs. 4a and 4b. To validate the cross-scale properties, we conducted coarse-graining of water

As shown in Fig. 4c, Deflex managed to capture the cross-scale emergence of Langevin dynamics in water particles. In contrast, other methods were limited to observing molecular dynamics at smaller scales and the dynamics of ice crystals at larger scales, failing to bridge the transition across scales. It also shows that Deflex detected the scale invariance of the system at the phase transition point of water (i.e., at 273.15K).

Original Manuscript:

As shown in Fig. 4, Deflex managed to capture the cross-scale emergence of Langevin dynamics in water particles. In contrast, other methods were limited to observing molecular dynamics at smaller scales and the dynamics of ice crystals at larger scales, failing to bridge the transition between these regimes. It is also shown that the Deflex detects the scale invariance of at phase transitions for water at 273.15K.

Revised Manuscript (Page 8-9):

As listed in Table 1, Deflex detected the Navier-Stokes equation of fluid dynamics through small-scale observations, which cannot be discovered by other methods. In larger-scale random observations, Deflex extracted the velocity distribution of the fluid within the vortex flow. These formulas correspond to different manifestations of long-standing laws in physics. For complex distribution formulas like velocity distribution, most compared methods are infeasible (shown in Table 2), and only AI Feynman and Deflex manage to discover.

Fig.4a also shows that the equation-based formulas discovered by Deflex can achieve a similar RMSE as other methods. For the velocity distribution, Deflex's discovery has a much smaller NLL loss than AI Feynman's, showing its better accuracy. In Fig. 4b, most comparatives, except for SINDy and Operon, become much slower than Deflex with the increase of formula complexity. Among them, AI Feynman is around 10 times slower than Deflex.

Original Manuscript:

As shown in Table 1, the patterns we obtained correspond to different manifestations of long-standing laws applied in physics. Also shown in Fig.4a, we obtained the Navier-Stokes equation of fluid dynamics through small-scale observations. In larger-scale random observations, we acquired information such as the velocity distribution of the fluid within the vortex flow. Fig. 4b shows the time consumption of different methods. When the complexity of the formulas increases, the PySR and AI Feynman methods became much slower than Deflex (approximately $10\times$ slower for velocity distribution). For very complex formulas including Navier-Stokes equation and Langevin dynamics for particle motion, the PySR and AI Feynman methods became infeasible.

In addition, we compared the EMD across different coarse-graining scales of Deflex with that of AI Feynman and the renormalization of known formulas. Fig. 4d shows that predictions from Deflex kept the lowest EMD across different scales, indicating the Deflex's ability to overcome the scale gap issue in complex systems. Meanwhile, other methods with a single setting could only capture the patterns at a single scale, and the EMD increased significantly when the scale changes.

Revised Manuscript (Page 9):

2.7 Comparison on Benchmark Dataset

We finally evaluated Deflex on the public benchmark dataset of Feynman Symbolic Regression Database [24]. This benchmark dataset is generated by sampling from 120 classical formulas spanning different fields of physics, with added noise to simulate real-world conditions. It captures essential characteristics of real-world mathematical formulas, such as symmetry, while also being a classic, well-established set of problems for symbolic regression. This dual characteristic facilitates a feasible performance comparison against other SR-based AI methods. In the experiment, Deflex was compared against Operon, SciMED, gplearn, AI Feynman 2.0, and PySR. We compared their performance by measuring the trade-off between the average RMSE of discovered formulas and the total runtime for the discovery. Note that SINDy was not included as it is sparse regression-based and not designed for symbolic regression tasks, while DEAP was omitted since it works as the backend of gplearn in the current task.

As shown in Fig. 4d, Deflex demonstrates a quite competitive performance among all compared methods on the benchmark dataset in terms of the accuracy-efficiency tradeoff, although it is not specialized for these traditional, closed-form equations. In particular, Deflex is only slightly outperformed by Operon, which relies on a fixed symbol set and is optimized for runtime. In our experiment, Deflex managed to discover the formula with an RMSE of 0.075 in just 146.2 seconds, which was quite close Operon with an RMSE of 0.0663 in 135.8 seconds. And Deflex and Operon showed significant advantages over other methods.

Comment 1.8:

The last paragraph in the Discussion section overpromises the empirical results presented in the study.

Response to Comment 1.8:

We thank the reviewer for this comment. We agree that some claims might have extended beyond the direct support of the presented empirical results, and we have revised the sentences with a more objective manner.

Revised Manuscript:

In this work, we have presented Deflex, a framework for discovering governing equations from multiscale complex systems. Our empirical results demonstrate its ability to identify symbolic rules across different scales and, in some cases, reveal previously undocumented relationships from observational data. The proposed methodology for representing interactions among high-dimensional entities suggests potential applicability to domains beyond those tested in our experiments. Future work could explore the practical meaning of the undocumented formulas with area experts, and Deflex's further use in areas such as atmospheric dynamics modeling [20] and materials science [44], particularly for systems where strong scale interdependencies pose challenges to conventional methods. We hope that this work serves as a step toward more capable automated scientific discovery in a range of disciplines.

Original Manuscript:

In conclusion, Deflex offers a reliable and efficient AI method for the automatic discovery of multi-scale rules from complex systems. It enables deep formula discovery across massive elements and multiple scales and may uncover unknown rules in complex systems. Its methodology for handling the interactions among high-dimensional entities suggests potential generalization to areas beyond our experiments. Possible application domains include atmospheric dynamics modeling [34] and materials science [35], particularly for systems with strong scale interdependencies that challenge conventional analytical approaches. We hope that our work could be a useful tool for scientific discovery in many distinct disciplines.

Comment 1.9:

For a physics-inspired symbolic regression paper, the related work part is highly limited given the huge number of papers from the last several years and the development of the field.

Response to Comment 1.9:

We thank the reviewer for this comment. We agree that a comprehensive literature review can help us highlight the novelties and contributions of our work. We have significantly expanded the related work with NINE additional references to provide a more complete overview, which still show that our work differs from the recent studies with significant novelties and advantages.

Physics-inspired SR is indeed one of the most rapidly advancing branches of symbolic regression [1]. In theory, any symbolic regression problem can be solved by exhaustive search, provided that the target formula is covered by the symbolic expression space. However, since the search space grows exponentially, symbolic regression is considered an NP-hard problem and even finding relatively simple formulas can become computationally infeasible. Physics-inspired SR addresses this challenge by leveraging physics-based priors to accelerate the searching process. Broadly, their studies can be categorized into the following directions.

1. Constraining the search space with physical knowledge. This includes introducing priors for properties such as monotonicity [1] and convexity [2]. Another prominent example is SINDy [3], which uses sparsity-promoting optimization to identify concise models, drastically reducing the search space of all possible functions. In tree-based SR, form constraints have long been used to improve performance [4]. AI Feynman employs physical symmetries as both soft and hard constraints to accelerate the search [5], while other work has leveraged soft constraints on the number of variables [6, 7]. Such methods can significantly speed up the search for physics-related problems, showing a distinct advantage over general-purpose SR on physical formulas. This highlights the effectiveness of the unique priors inherent in physical laws.

2. Using neural networks to enhance search efficiency. For instance, incorporating sparsity priors into neural networks has accelerated SR for astrophysical problems [6]. This technique is also utilized in AI Feynman and SciMED [5, 9]. Compared to traditional SR, neural networks often exhibit better fitting capabilities on massive noisy data, such as that sampled from real-world or simulated systems, making them particularly suitable for these scenarios. Other approaches have leveraged deep reinforcement learning [10] and even large language models (LLMs) recently to expedite the discovery of symbolic laws [11]. This class of work can effectively integrate existing human priors about the discovery of physical formulas.

These studies are highly relevant to ours and provide important inspirations. However, our work clearly presents novel ideas and shows significant advantages. On one hand, our work, in essence, was also inspired by the physical knowledge in term of the relevance across multiple scales in multi-scale complex system, which however, has never been considered in existing studies. On the other hand, we propose a neural-guided approach that can decompose the learned functions into elementary components for tractable discovery. In contrast, existing work lacks consideration of the elementary knowledge in the neural network, resulting low efficiency and even infeasible discovery for complex relationships. Our specific modifications are as follows.

Revised Manuscript:

Unfortunately, no existing AI methods can achieve automatic discovery of multiscale deep formulas in complex systems. While methods like sparse regression can effectively learn mathematical patterns from large datasets [9, 22], their reliance on pre-defined functional forms limits the exploration of unknown regularities. Symbolic regression (SR), based on symbolic trees and genetic programming [2, 8, 9, 23, 24], is a powerful tool for automatically discovering mathematical formulas with unknown forms from data. However, traditional SR methods suffer from low success rates and computational efficiency for identifying complex relationships. Recently, substantial progress has been made in symbolic regression, which has greatly enhanced the discovery of laws in real-world data. For instance, physics-inspired symbolic regression explored to incorporate physical knowledge (e.g., high-order symmetries) as priors [25, 26]. Also, neural network-integrated SR discovers novel patterns from representations learned from deep models (e.g., GNNs) [9]. Besides, many studies have enabled symbolic regression to find complex relationships on benchmark datasets by improving the searching strategies with evolutionary algorithms [8, 27], reinforcement learning [28], or Monte Carlo tree search [26]. Nonetheless, without high-order representations and tailored design, the automatic discovery of the above studies can hardly work for multiscale complex systems.

Original Manuscript:

> Unfortunately, no existing AI methods can achieve this. Fixed-form parametric models like sparse regression [9], provide us with remarkable successes in discovering state equations [10, 20], and governing equations [21] in various systems. However, they are inherently limited by their predefined form space, and therefore would fail in complex systems where prior knowledge of potential math patterns may not hold. Genetic programming [22], especially symbolic regression [1, 8, 9, 18] do not require prior knowledge, but fails to abstract concise math expressions from a large number of state variables in complex systems, due to their lack of compression and formula length reduction. Furthermore, their search space grows exponentially with the number of variables and expression depth [8, 18], making them computationally prohibitive to identify complex, nonlinear equations in such systems.

References of the response to this comment

[1] *Gupta, M., Cotter, A., Pfeifer, J., Voevodski, K., Canini, K., Mangylov, A., Moczydlowski, W., Van Esbroeck, A.: Monotonic calibrated interpolated look-up tables. J. Mach. Learn. Res.* ***17****(1), 3790–3836 (2016)*

[2] *Kronberger, G., Franca, F.O., Burlacu, B., Haider, C., Kommenda, M.: Shape-constrained symbolic regression—improving extrapolation with prior knowledge. Evolutionary Computation* ***30****(1), 75–98 (2022) https://doi.org/10.1162/evco_a_00294 https://direct.mit.edu/evco/articlepdf/30/1/75/1995582/evco_a_00294.pdf*

[3] *Makke, N., Chawla, S.: Interpretable scientific discovery with symbolic regression: a review. Artif. Intell. Rev.* ***57****(1) (2024) https://doi.org/10.1007/ s10462-023-10622-0*

[4] *Rudy, S.H., Brunton, S.L., Proctor, J.L., Kutz, J.N.: Data-driven discovery of partial differential equations. Science Advances* ***3****(4), 1602614 (2017) https://doi. org/10.1126/sciadv.1602614*

[5] *Udrescu, S.-M., Tegmark, M.: Ai feynman: A physics-inspired method for symbolic regression. Science Advances* ***6****(16), 2631 (2020) https://doi.org/10.1126/ sciadv.aay2631*

[6] *Jiang, N., Xue, Y.: Symbolic regression vianbsp;control variable genetic programming. In: Machine Learning and Knowledge Discovery in Databases: Research Track: European Conference, ECML PKDD 2023, Turin, Italy, September 18– 22, 2023, Proceedings, Part IV, pp. 178–195. Springer, Berlin, Heidelberg (2023). https://doi.org/10.1007/978-3-031-43421-1_11*

[7] *Jiang, N., Xue, Y.: Racing control variable genetic programming for symbolic regression. In: Proceedings of the 38th AAAI Conference on Artificial Intelligence (AAAI 2024). AAAI Press, Vancouver, Canada (2024). https://doi.org/10.1609/ aaai.v38i11.29187*

[8] *Udrescu, S.-M., Tan, A., Feng, J., Neto, O., Wu, T., Tegmark, M.: Ai feynman 2.0: Pareto-optimal symbolic regression exploiting graph modularity. Advances in Neural Information Processing Systems* ***33****, 4860–4871 (2020)*

[9] *Keren, L.S., Liberzon, A., Lazebnik, T.: A computational framework for physics-informed symbolic regression with straightforward integration of domain knowledge. Scientific Reports* ***13****(1), 1249 (2023)*

[10] *Mundhenk, T., Landajuela, M., Glatt, R., Santiago, C.P., faissol, D., Petersen, B.K.: Symbolic regression via deep reinforcement learning enhanced genetic programming seeding. In: Ranzato, M., Beygelzimer, A., Dauphin, Y., Liang, P.S., Vaughan, J.W. (eds.) Advances in Neural Information Processing Systems, vol. 34, pp. 24912–24923. Curran Associates, Inc., Second Virtual Conference (2021)*

[11] *Shojaee, P., Nguyen, N.-H., Meidani, K., Farimani, A.B., Doan, K.D., Reddy, C.K.: Llm-srbench: A new benchmark for scientific equation discovery with large language models. arXiv preprint arXiv:2504.10415 (2025)*

Comment 1.10

First statement - cite?

Response to Comment 1.10:

We thank the reviewer for the comment. We have added two references to support this statement in the revised manuscript.

Many past scientific studies, from Kepler's laws derived to from astronomical observations and the Boltzmann distribution mentioned in our manuscript, have demonstrated the paradigm of finding mathematical forms from the observations of the natural or social world. In 1961, Eugene Wigner discussed and summarized this class of research paradigm in "The Unreasonable Effectiveness of Mathematics in the Natural Sciences" [1], and the examples he cited—Galileo's law of falling bodies, the law of universal gravitation, matrix mechanics, and the quantum theory of the Lamb shift—all belong to this category. Further summaries of this paradigm include works such as [2]. In the field of automated symbolic discovery, important work like [3] has also explicitly proposed that this research constitutes the automation of this particular process of discovering mathematical forms from data.

Based on above discussions, we have added two references to the statement.

Revised Manuscript:

> Discovering rules in mathematical forms, such as energy conservation and Boltzmann distribution, in nature and society has always been an important endeavor in scientific research [1, 2]. In recent years, artificial intelligence (AI) has played an increasingly significant role in identifying mathematical formulas from different systems [2–5]. However, discovering genuine mathematical formulas in a fully automated fashion, without pre-specified equation structures, remains largely limited to single-scale and few-body systems of lower complexity [1, 6–11].

> Discovering rules in mathematical forms, such as energy conservation and Boltzmann distribution, in nature and society has always been an important endeavor in scientific research [1, 2]. In recent years, artificial intelligence (AI) has played an increasingly significant role in identifying mathematical formulas from different systems [2–5]. However, discovering genuine mathematical formulas in a fully automated fashion, without pre-specified equation structures, remains largely limited to single-scale and few-body systems of lower complexity [1, 6–11].

Original Manuscript:

> Discovering rules in mathematical forms, such as energy conservation and Boltzmann distribution, in nature and society has always been an important endeavor in scientific research. In recent years, artificial intelligence (AI) has played an increasingly significant role in identifying mathematical formulas from different systems [1–4]. However, it is primarily limited to single-element or few-element systems on a certain scale, such as identifying dominant equations of partial differential systems [5–11].

References of the response to this comment

[1] *Wigner, E.P.: The unreasonable effectiveness of mathematics in the natural sciences. In: Mathematics and Science, pp. 291–306. World Scientific, Singapore (1990)*

[2] *Tegmark, M.: The mathematical universe. Foundations of physics **38**(2), 101–150 (2008)*

[3] *Schmidt, M., Lipson, H.: Distilling free-form natural laws from experimental data. Science **324**(5923), 81–85 (2009) https://doi.org/10.1126/science.1165893*

Comment 1.11

The claim with cites 5-11, this is not true, the authors should check symbolic regression papers from 2023 forward, there are many counter examples.

Response to Comment 1.11:

We thank the reviewer for this comment. We have carefully reviewed the most recent work suggested by the reviewer and found that our work still differs from the most recent studies on symbolic regression and presents significant novelties and advantages. To respond this comment, we have revised the corresponding statement by including more recent studies as references and highlighting their limitations.

As the reviewer pointed out, recent advances, particularly in areas like sparse symbolic regression, have shown remarkable success in tackling complex, large-scale systems. Nonetheless, they could not achieve fully automatic discovery in complex systems yet, which are characterized by intricate and higher-order formulas. These methods either require pre-specifying the function set or suffer from low computational efficiency and success rates for identifying complex relationships, which limit their application only to single-scale and few-body systems with low complexity. For example, sparse regression can effectively learn mathematical patterns from large datasets, but can hardly explore unknown regularities due to the reliance on pre-defined functional forms. Symbolic regression can achieve automatic discovery based on symbolic trees and genetic programming. However, traditional symbolic regression methods have low success rates and computational efficiency. Therefore, we can conclude that current AI-based automatic discovery methods without pre-specified formula structures (instead of general AI-based discovery methods) are still limited to the system with low complexity. This still motivates our work of fully automatic formula discovery without being constrained by pre-specified functions.

Considering the above discussion, we have conducted following modifications in the revised manuscript.

1. We have included more recent work and modified the statement to highlight their limitations.

Revised Manuscript (Page 1):

> Discovering rules in mathematical forms, such as energy conservation and Boltzmann distribution, in nature and society has always been an important endeavor in scientific research [1, 2]. In recent years, artificial intelligence (AI) has played an increasingly significant role in identifying mathematical formulas from different systems [2–5]. However, discovering genuine mathematical formulas in a fully automated fashion, without pre-specified equation structures, remains largely limited to single-scale and few-body systems of lower complexity [1, 6–11].

Original Manuscript:

> Discovering rules in mathematical forms, such as energy conservation and Boltzmann distribution, in nature and society has always been an important endeavor in scientific research. In recent years, artificial intelligence (AI) has played an increasingly significant role in identifying mathematical formulas from different systems [1–4]. However, it is primarily limited to single-element or few-element systems on a certain scale, such as identifying dominant equations of partial differential systems [5–11].

2. We have revised the related work with more discussions to support the revised statement.

Revised Manuscript (Page 2):

Unfortunately, no existing AI methods can achieve automatic discovery of multiscale deep formulas in complex systems. While methods like sparse regression can effectively learn mathematical patterns from large datasets [9, 22], their reliance on pre-defined functional forms limits the exploration of unknown regularities. Symbolic regression (SR), based on symbolic trees and genetic programming [2, 8, 9, 23, 24], is a powerful tool for automatically discovering mathematical formulas with unknown forms from data. However, traditional SR methods suffer from low success rates and computational efficiency for identifying complex relationships. Recently, substantial progress has been made in symbolic regression, which has greatly enhanced the discovery of laws in real-world data. For instance, physics-inspired symbolic regression explored to incorporate physical knowledge (e.g., high-order symmetries) as priors [25, 26]. Also, neural network-integrated SR discovers novel patterns from representations learned from deep models (e.g., GNNs) [9]. Besides, many studies have enabled symbolic regression to find complex relationships on benchmark datasets by improving the searching strategies with evolutionary algorithms [8, 27], reinforcement learning [28], or Monte Carlo tree search [26]. Nonetheless, without high-order representations and tailored design, the automatic discovery of the above studies can hardly work for multiscale complex systems.

Original Manuscript:

Unfortunately, no existing AI methods can achieve this. Fixed-form parametric models like sparse regression [9], provide us with remarkable successes in discovering state equations [10, 20], and governing equations [21] in various systems. However, they are inherently limited by their predefined form space, and therefore would fail in complex systems where prior knowledge of potential math patterns may not hold. Genetic programming [22], especially symbolic regression [1, 8, 9, 18] do not require prior knowledge, but fails to abstract concise math expressions from a large number of state variables in complex systems, due to their lack of compression and formula length reduction. Furthermore, their search space grows exponentially with the number of variables and expression depth [8, 18], making them computationally prohibitive to identify complex, nonlinear equations in such systems.

Comment 1.12:

"Such chaos and order emergence also introduces different levels of regularity, requiring us to account for both conservation laws and stochastic behaviors in scientific analysis" - cite?

Response to Comment 1.12:

We thank the reviewer for this comment. We have added corresponding references and detailed discussions to support this claim in the revised manuscript.

Our original statement refers to how the governing principles of complex systems manifest as emergence and chaos at different scales. Both chaos and order emergence have been widely regarded as the core characteristics of complex systems [1]. For example, some researches on collective complex system focus on a specific dynamic [2, 3]: the transition where order emerges from disorder at one scale, only to revert to a chaotic state at larger spatiotemporal scales. The 2021 Nobel Prize in Physics [4] further established this concept as a central proposition in the field. According to [4], a fundamental question in complex systems science is the scale-dependent nature of physical laws, which is shaped by this interplay of emergence and chaos.

To respond this comment, we have expanded the statement with more detailed discussions and included the corresponding citations in the revised manuscript.

Revised Manuscript:

Meanwhile, increasing effort has been put into research on complex systems, such as fluid dynamics [4], crowd movement [12], climate evolution [13, 14], and social systems [15], which explain many complex phenomena and behaviors in the real world. Many distinct properties of complex systems come from the transition to chaos and the emergence of order from disorder across different spatial and temporal scales [16, 17]. Such chaos and order emergence also introduces different levels of regularity, requiring scientific analysis to account for distinct mathematical forms: deterministic descriptions in conservation laws and probability distributions for stochastic behaviors [18]. AI methods have demonstrated great potential for discovering the governing rules behind the interactions of massive elements at multiple scales in these complex systems. For instance, their capacity to efficiently approximate solutions for complex known equations in physics [19], their ability to discern intricate patterns from large-scale observational data in domains such as meteorology or climate science [20], and the emerging success of large models in learning or representing complex mathematical relationships from scientific data [21] all underscore this potential.

Original Manuscript:

Meanwhile, increasing effort has been put into research on complex systems, such as fluid dynamics [3], crowd movement [12], climate evolution [13, 14], and social systems [15], which would help us understand many complex phenomena and behaviors in the real world. The transition to chaos and the emergence of order from disorder across different spatial and temporal scales lead to many distinct properties of complex systems [16, 17]. Such chaos and order emergence also introduces different levels of regularity, requiring us to account for both conservation laws and stochastic behaviors in scientific analysis. AI methods show great potential for discovering the governing rules behind the interactions of massive elements at multiple scales in these complex systems.

References of the response to this comment

[1] *Kaneko, K., Tsuda, I.: Complex Systems: Chaos and Beyond: Chaos and Beyond: A Constructive Approach with Applications in Life Sciences. Springer, Berlin, Heidelberg: Springer (2001)*

[2] *Vicsek, T.: Collective Motion. Elsevier (2012)*

[3] *[3] Beekman, M., Sumpter, D.J.T., Ratnieks, F.L.W.: Phase transition between disordered and ordered foraging in pharaoh's ants. Proceedings of the National Academy of Sciences **98**(17), 9703–9706 (2001) https://doi.org/10.1073/pnas. 161285298 https://www.pnas.org/doi/pdf/10.1073/pnas.161285298*

[4] *Ravishankara, A.R., Randall, D.A., Hurrell, J.W.: Complex and yet predictable: The message of the 2021 nobel prize in physics. Proceedings of the National Academy of Sciences **119**(2), 2120669119 (2022) https://doi.org/10.1073/pnas. 2120669119 https://www.pnas.org/doi/pdf/10.1073/pnas.2120669119.*

Comment 1.13:

"AI methods show great potential for discovering the governing rules behind the interactions of massive elements at multiple scales in these complex systems" - right, but spesisfic examples?

Response to Comment 1.13:

The authors thank the reviewer for the comment. There are several prominent examples demonstrating the potential of AI-based rule discovery (not in the form of mathematic formulas) in complex systems. These include solving for relationships within complex systems by incorporating physics-informed priors [1]; identifying unique patterns and generating highly accurate predictions from large-scale observational data in meteorology [2]; and the emergent capability of large

language models to propose mathematical conjectures autonomously from scientific literatures and data [3, 4].

To respond this comment, we have added three evident examples with references in the revised manuscript.

Revised Manuscript:

> AI methods have demonstrated great potential for discovering the governing rules behind the interactions of massive elements at multiple scales in these complex systems. For instance, their capacity to efficiently approximate solutions for complex known equations in physics [19], their ability to discern intricate patterns from large-scale observational data in domains such as meteorology or climate science [20], and the emerging success of large models in learning or representing complex mathematical relationships from scientific data [21] all underscore this potential.

Original Manuscript:

> behaviors in scientific analysis. AI methods show great potential for discovering the governing rules behind the interactions of massive elements at multiple scales in these complex systems.

References of the response to this comment

[1] *Pun, G. P. P., Batra, R., Ramprasad, R. & Mishin, Y. Physically informed artificial neural networks for atomistic modeling of materials. Nat Commun* ***10****, 2339 (2019).*

[2] *Bi, K. et al. Accurate medium-range global weather forecasting with 3D neural networks. Nature* ***619****, 533–538 (2023).*

[3] *Romera-Paredes, B. et al. Mathematical discoveries from program search with large language models. Nature* ***625****, 468–475 (2024).*

[4] *Shojaee, P. et al. LLM-SRBench: A New Benchmark for Scientific Equation Discovery with Large Language Models. Preprint at https://doi.org/10.48550/arXiv.2504.10415 (2025).*

Comment 1.14

"Furthermore, their search space grows exponentially with the number of variables and expression depth [8, 18], making them computationally prohibitive to identify complex, nonlinear equations in such systems." - you can claim it to any reinforcement learning problem but still, it is working. This is because the search process is more important than the option space size.

Response to Comment 1.14:

We thank the reviewer for this comment, which helps us identify the key limitations of related work. We agree that the search process is a critical factor of the methodology, and have updated the discussions on the corresponding work.

Our intention was to highlight the importance of computational efficiency for designing an AI-based formula discovery method. Considering this issue, we are aware that many recent studies on symbolic regression have explored to improve the efficiency significantly by leveraging different search strategies, such as evolutionary algorithms [1, 2], reinforcement learning [3], and Monte Carlo tree search [4]. Therefore, we have followed the reviewer's suggestion to emphasize the corresponding related work on search process of symbolic regression. In addition, considering the reviewer's other comments on the related work, we have conducted a comprehensive literature review and rewritten the corresponding paragraph as follows in the revised manuscript.

Revised Manuscript:

> Unfortunately, no existing AI methods can achieve automatic discovery of multiscale deep formulas in complex systems. While methods like sparse regression can effectively learn mathematical patterns from large datasets [9, 22], their reliance on pre-defined functional forms limits the exploration of unknown regularities. Symbolic regression (SR), based on symbolic trees and genetic programming [2, 8, 9, 23, 24], is a powerful tool for automatically discovering mathematical formulas with unknown forms from data. However, traditional SR methods suffer from low success rates and computational efficiency for identifying complex relationships. Recently, substantial progress has been made in symbolic regression, which has greatly enhanced the discovery of laws in real-world data. For instance, physics-inspired symbolic regression explored to incorporate physical knowledge (e.g., high-order symmetries) as priors [25, 26]. Also, neural network-integrated SR discovers novel patterns from representations learned from deep models (e.g., GNNs) [9]. Besides, many studies have enabled symbolic regression to find complex relationships on benchmark datasets by improving the searching strategies with evolutionary algorithms [8, 27], reinforcement learning [28], or Monte Carlo tree search [26]. Nonetheless, without high-order representations and tailored design, the automatic discovery of the above studies can hardly work for multiscale complex systems.

Original Manuscript:

> Unfortunately, no existing AI methods can achieve this. Fixed-form parametric models like sparse regression [9], provide us with remarkable successes in discovering state equations [10, 20], and governing equations [21] in various systems. However, they are inherently limited by their predefined form space, and therefore would fail in complex systems where prior knowledge of potential math patterns may not hold. Genetic programming [22], especially symbolic regression [1, 8, 9, 18] do not require prior knowledge, but fails to abstract concise math expressions from a large number of state variables in complex systems, due to their lack of compression and formula length reduction. Furthermore, their search space grows exponentially with the number of variables and expression depth [8, 18], making them computationally prohibitive to identify complex, nonlinear equations in such systems.

References of the response to this comment

[1] *Udrescu, S.-M. et al. AI Feynman 2.0: Pareto-optimal symbolic regression exploiting graph modularity. in Advances in Neural Information Processing Systems (eds Larochelle, H., Ranzato, M., Hadsell, R., Balcan, M. F. & Lin, H.) vol.* ***33*** *4860–4871 (Curran Associates, Inc., 2020).*

[2] *Jiang, N. & Xue, Y. Racing control variable genetic programming for symbolic regression. in Proceedings of the Thirty-Eighth AAAI Conference on Artificial Intelligence and Thirty-Sixth Conference on Innovative Applications of Artificial Intelligence and Fourteenth Symposium on Educational Advances in Artificial Intelligence vol.* ***38*** *12901–12909 (AAAI Press, 2024).*

[3] *Mundhenk, T. et al. Symbolic Regression via Deep Reinforcement Learning Enhanced Genetic Programming Seeding. in Advances in Neural Information Processing Systems vol.* ***34*** *24912–24923 (Curran Associates, Inc., 2021).)*

[4] *Sun, F., Liu, Y., Wang, J.-X. & Sun, H. Symbolic Physics Learner: Discovering governing equations via Monte Carlo tree search. in (2022).*

Comment 1.15:

"However, traditional symbol-tree-based symbolic regressions may fail to learn higher-order mathematical relationships such as summation and mapping among a large volume of elements in complex systems." - why? Even GA-based SR can do it, just define such functions as basic functions...

Response to Comment 1.15:

We thank the reviewer for this comment. This is because these methods rely on pre-defining higher-

order relationships, and have significant limitations in both expression ability and discovery efficiency for higher-order mathematical relationships over a large volume of interacting elements in complex systems. To make this clear, we have revised the corresponding paragraph with more discussions in the revised manuscript.

We agree that, in principle, operators such as summation and mapping can be pre-defined as basic functions within a traditional genetic programming-based symbolic regression framework. Despite this, traditional methods have significant limitations in both expression ability and discovery efficiency for higher-order mathematical relationships over massive interacting elements. These limitations hinder the automatic formula discovery from complex systems, which is the focus of our work.

Different complex systems may have different and even unknown higher-order relationships. In the scenarios of scientific discovery, it may not be able to pre-define the corresponding higher-order relationships in prior. Besides, the potential higher-order relationships may have a large number of structures, scopes and arguments in complex systems, leading to a huge combinatorial search space for identifying the correct elements. Traditional symbolic regression methods may fail to identify correct relationships from such huge search spaces. Even from the implementation perspective, most of current symbolic regression libraries do not provide programming support for defining higher-order functions. Therefore, the requirement of pre-defining higher-order relationships greatly hinders the application of traditional symbolic regression-based methods for achieving automatic formula discovery in complex systems, which also motivates us to design new methods. Our work proposes a novel symbolic regression framework based on Lambda calculus, which has better ability to describe higher-order relationships via abstraction. In particular, the Lambda calculus system has been proved the completeness of the supported higher-order expressions [1].

Based on above discussion, we have revised the corresponding paragraph in the revised manuscript as follows.

Revised Manuscript:

> However, traditional tree-based symbolic regression may have significant limitations in both expression ability and discovery efficiency for higher-order mathematical relationships (such as summation or mapping) over a large volume of interacting elements in complex systems. The former limitation stems from the simple tree structures in representing variable bindings and operations over collections. The latter is due to the combinatorial search space of potentially massive arguments for complex systems. To address these limitations, we present Deflexpressor, a symbolic regression subsystem that leverages lambda calculus expressions. The inherent capabilities of Lambda calculus for function abstraction, variable binding, and higher-order functions provide a powerful and flexible representational framework. This allows Deflexpressor to express and discover free-form higher-order mathematical relationships without pre-specifying. In addition, Deflexpressor can efficiently navigate the search space for complex symbolic structures via two novel designs. One is to guide lambda expression construction by incorporating a rule-based generator and an auto-regressive neural network. The other optimizes the efficiency-diversity trade-offs of expression generation and mutation by extending genetic programming mutations with transformations specified for lambda calculus (e.g., inspired by beta-reduction or exploring variations in function arity and application).

Original Manuscript:

> However, traditional symbol-tree-based symbolic regressions may fail to learn higher-order mathematical relationships such as summation and mapping among a large volume of elements in complex systems. Therefore, we further present Deflexpressor, a symbolic regression subsystem based on Lambda calculus expressions, which has better abstraction ability and can learn free-form higher-order mathematical relationships effectively. By incorporating a rule-based generator and autoregressive neural network, as well as extending the genetic programming mutations with lambda calculus-specific transformations, Deflexpressor greatly optimizes the efficiency-diversity trade-offs during the expression generation and mutation in symbolic regression.

References of the response to this comment

[1] *Nederpelt, R. & Geuvers, H. Type Theory and Formal Proof: An Introduction. (Cambridge University Press, Cambridge, 2014).*

Comment 1.16:

"which effectively addresses the aforementioned challenges for AI-based formula discovery from complex systems." - This is a really bad practice to compliment yourself in your own work.

Response to Comment 1.16:

Many thanks for pointing out this writing issue. We have revised it to maintain a more objective tone, focusing on the intended purpose of our method design. Specific modifications are as follows.

Revised Manuscript:

> Thus far, we have outlined the design principles and general processes of the proposed method, which aim to address the aforementioned challenges for AI-based formula discovery from complex systems. Concerning the volume problem, Deflex uses

Original Manuscript:

> Thus far, we have outlined the design principle and general process of the proposed method, which effectively addresses the aforementioned challenges for AI-based formula discovery from complex systems. Concerning the volume problem, Deflex uses a

Comment 1.17:

RMSE is basic; you do not need to state it in Eq. (1).

Response to Comment 1.17:

Thanks for this suggestion. We fully agree that RMSE is very basic and do not need to present its definition equation. Therefore, we have removed the defining Equation of RMSE in the revised manuscript. Specific modifications are as follows.

Revised Manuscript:

> - For the formulas in the form of equations and invariants, we employ the relative mean square error (RMSE) between the prediction and the true value. Specifically, all compared methods are given the same convergence threshold. Note that, in such a case, the RMSEs of different methods are hardly comparable since there is almost no structural difference (except for residual terms with negligible errors) in the commonly identified formulas in these forms.

Original Manuscript:

in Eq. (5) that measures the likelihood of the model fitting with real data, we employ the relative mean square error (MSE) measure the squared absolute error between the prediction and the true value,

$$RMSE(f) = \frac{\sqrt{\frac{1}{N}\sum_{i\in I}(f(x_i) - y_i)^2}}{\max_{i\in I} y_i}, \quad (1)$$

where I and N is the index set and number of data points, $f(x_i)$ is the predicted value, and y_i is the true value. Since the formulas are represented as distributions, we

Comment 1.18:

I find Figure 4 hard to read... Add a grid on the y-axis, keep the same colors, do not use shorts (P1) as it is hard to follow.

Response to Comment 1.18:

Many thanks for this comment, which helps to improve the readability of the experimental figures. We have revised the figures according to the reviewer's suggestion. We believe the readability of Fig. 4 has been significantly improved. Specific modifications are as follows.

Revised Manuscript:

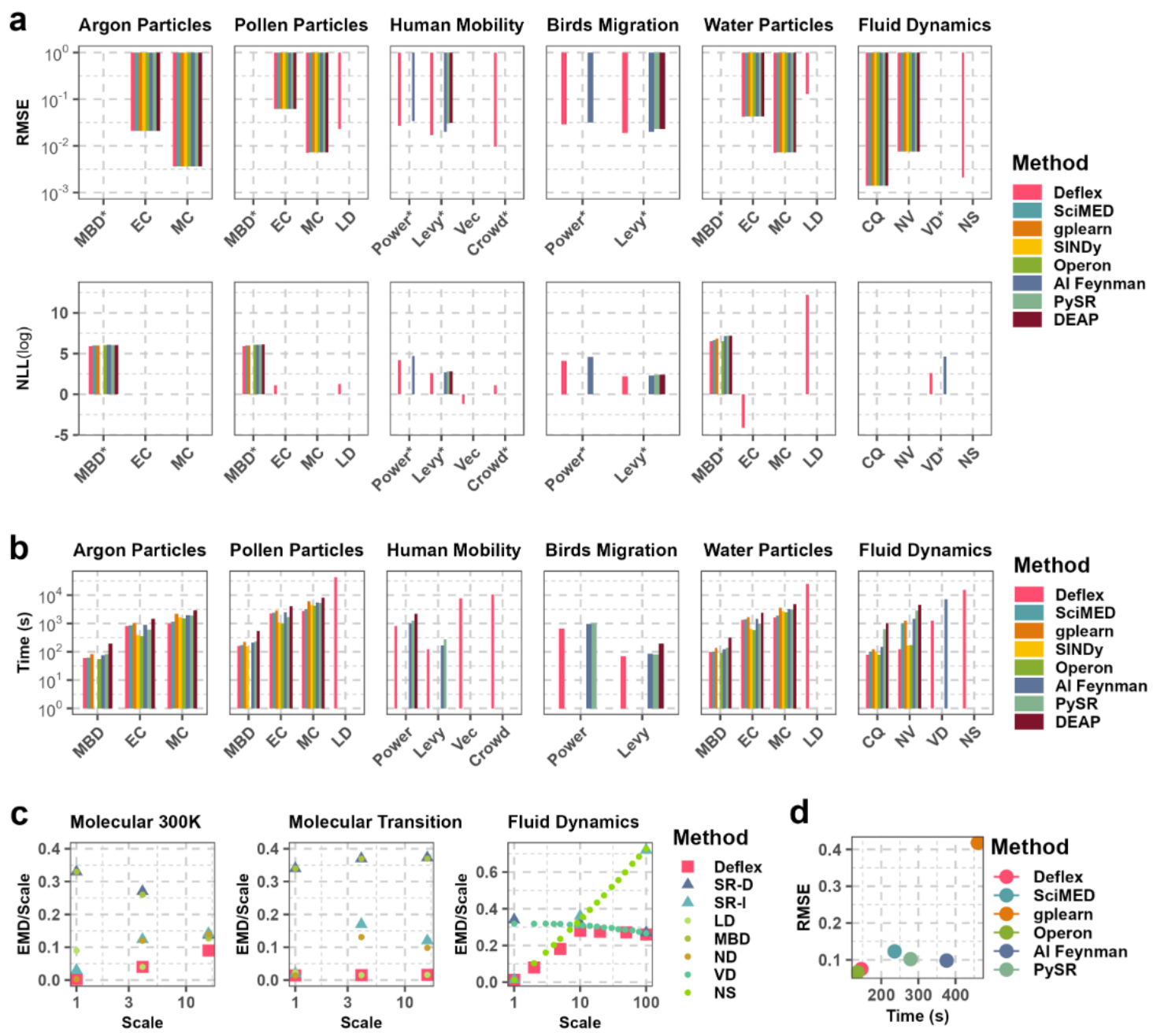

Fig. 4 Experimental results of Deflex in terms of accuracy (**a**), efficiency (**b**), and scalability (**c**). **a** compares the RMSE and NLL loss of the discovered formulas by Deflex and other methods in the experiments of particle motions, human and bird collective movement, water particles, and fluid dynamics. The abbreviations represent the Maxwell-Boltzmann distribution (MBD), energy conservation law (EC), momentum conservation (MC), Langevin dynamics (LD), continuity of quality(CQ), Newton viscosity law (NV), velocity distribution in fluid dynamics(VD), Navier-Stokes equation (NS), power law in human mobility (Power), Levy flight rule (Levy), vector navigation law (Vec), and crowd power law, respectively. Among them, the formulas in the form of distributions are marked with $*$. The absence of the method indicates that the corresponding metrics are not available for the method. **b** depicts the running time and usability of Deflex compared with other methods in different experiments. The horizontal axis represents the running time of the methods and the absence of the method indicates it is infeasible to discover the formula. Only Deflex can discover all the formulas in the experiments, and the time consumption of Deflex is significantly lower than that of most other methods for complex formulas. **c** shows the EMD values of known formulas and formulas discovered by Deflex and other methods across different coarse-graining scales for water particles' motion and fluid dynamics, where "SR-I" and "SR-D" represent the symbolic regression methods with invariant and distributional form settings, respectively. **d** shows the running time and relative mean square error for Deflex and other methods on the public benchmark dataset of Feynman Symbolic Regression Database.

Original Manuscript:

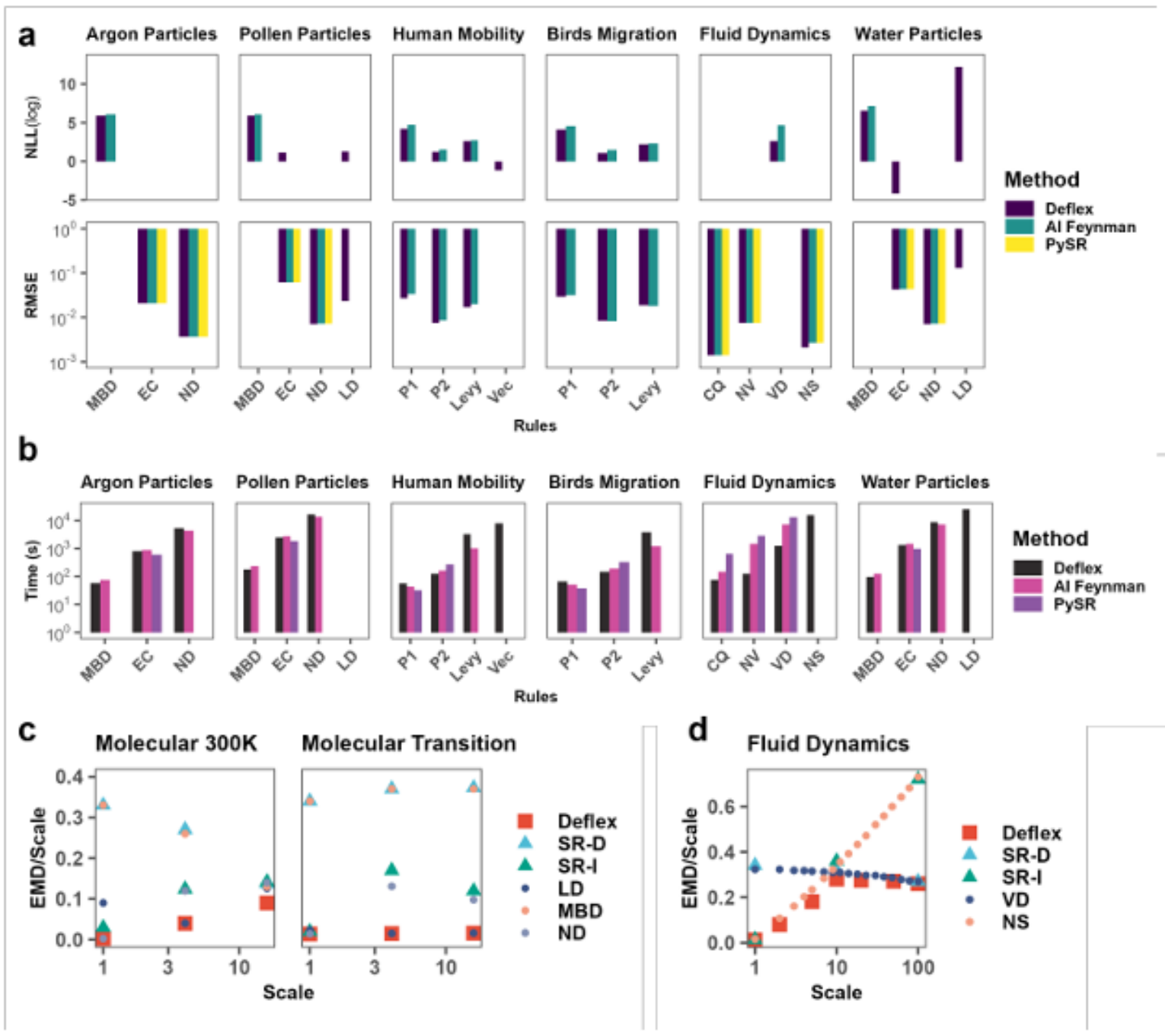

Fig. 4 Experimental results of Deflex in terms of accuracy (**a**), efficiency (**b**), and scalability (**c**). **a** compares the RMSE and NLL loss of the discovered formulas by Deflex and other methods in the experiments of particle motions, human and bird collective movement, fluid dynamics, and water particles. The abbreviations represent the Maxwell-Boltzmann distribution (MBD), energy conservation law (EC), Newtonian dynamics (ND), Langevin dynamics (LD), continuity of quality(CQ), Newton viscosity law(NV), velocity distribution in fluid dynamics(VD), Navier-Stokes equation (NS), first and second power law in human mobility(P1 and P2), Levy flight rule(Levy), and vector navigation law (Vec), respectively. The absence of the method indicates that the corresponding metrics are not available for the method. **b** depicts the running time and usability of Deflex compared with other methods in different experiments. The horizontal axis represents the running time of the methods and the absence of the method indicates it is infeasible to discover the formula. Only Deflex can discover all the formulas in the experiments, and the time consumption of Deflex is significantly lower than that of other methods for complex formulas. **c** and **d** shows the EMD values of known formulas and formulas discovered by Deflex and other methods across different coarse-graining scales for water particles motion (**c**) and fluid dynamics (**d**), where "SR-I" and "SR-D" represent the symbolic regression methods with invariant and distributional form settings, respectively.

Comment 1.19:

Figure 1 seems redundant to me, but the authors can leave it if they feel it is helpful.

Response to Comment 1.19:

We thank the reviewer for the comment. We believe it would be helpful to provide a concept figure for general audience to better understand the motivation and background of our work. Therefore, we choose to keep it in the manuscript.

Comment 1.20:

(Remarks on code availability):

I was not able to see the code, which further supports my criticism of this study: https://prnt.sc/gMutxu3XL1wG

Response to Comment 1.20:

We thank the reviewer for this comment. We have doublechecked our public source code and ensure it can be accessed at https://github.com/yhqjohn/deflex for artifact evaluation.

=======Reviewer #2=======

Comments to the Authors:

The paper tackles the problem of identifying scale-specific formulas in multi-scale complex systems, a current challenge for existing AI-based symbolic regression algorithms. The authors achieve this by developing a novel AI-based framework called Deflex.

By treating mathematical formulas as probability distributions within an energy-based model, in combination with lambda calculus, they are able to discover symbolic formulas in six different complex systems. They handle the usual issues related to the exponential explosion in the parameter space by breaking the complex system into smaller, manageable sub-problems. Their experiments show the efficiency of their algorithm in discovering formulas in multi-scale setups, performing better than other state-of-the-art algorithms. They are also able to discover new formulas in these systems, besides the established ones.

Moreover, they lay down clear directions of future research for improving their algorithm. I believe that this is quite an original idea, which can have many applications in various fields of research. The experiments are explained clearly, and the obtained results are well interpreted. I believe that this is a very nice paper and should be accepted for publication.

Response to Reviewer #2:
The authors thank the reviewer very much for this positive comment. We appreciate the reviewer for recognizing the importance of our studied problem, the contribution and novelty of the proposed method, as well as the potential impact.

=======Reviewer #3=======

Comments to the Authors:

The ideas presented by the authors are very interesting, this approach is a valuable contribution to explainable AI.

Response to Reviewer #3:

We thank the reviewer for this positive comment. We also appreciate the suggestions for strengthening the manuscript's analysis and documentation.

Comment 3.1:

Nevertheless, before this can be published, the authors definitively need to analyse the performance of the algorithm in further detail and document

- ***intermediate results (what do we get after each step of the algorithm).***
- *results on international benchmark data sets.*
- *comparisons with other state of the art algorithms used on the same data sets.*

Response to Comment 3.1:

We thank the reviewer for this comment, which helps us clarify the details of the algorithm design. Deflex contains two subsystems: Deflexpressor, a symbolic regression subsystem based on Lambda calculus, and Deflexformer, an energy-based DNN subsystem. The workflow of Deflex consists of four phases, as shown in Fig. 3a-d. The intermediate results at each phase are as follows.

- Phase 1 (Generation) outputs a synthetic dataset, where each data point is sampled from the randomly generated formulas by Deflexpressor.

- Phase 2 (Pretraining) obtains a pre-trained Deflexformer block (a Transformer-like neural network) on the synthetic dataset in Phase 1.

- Phase 3 (Learning) obtains an augmented dataset from the observation dataset of the targeted complex system. In this phase, we train the Deflexforer, initially built by cascading the pre-trained Deflexformer block multiple times, on the observation dataset. Then, we feed the observation dataset with some random data to the trained Deflexformer for inference, which generates a representation dataset including the data representations output by all the Deflexformer blocks.

- Phase 4 (Regression) finally outputs the desired symbolic expressions by Deflexpressor that can well fit on the representation dataset.

To respond this comment, we have conducted following modifications in the revised manuscript.

1. We have updated Fig. 3 to visualize the workflow as well as the intermediate results.

Revised Manuscript (Page 24):

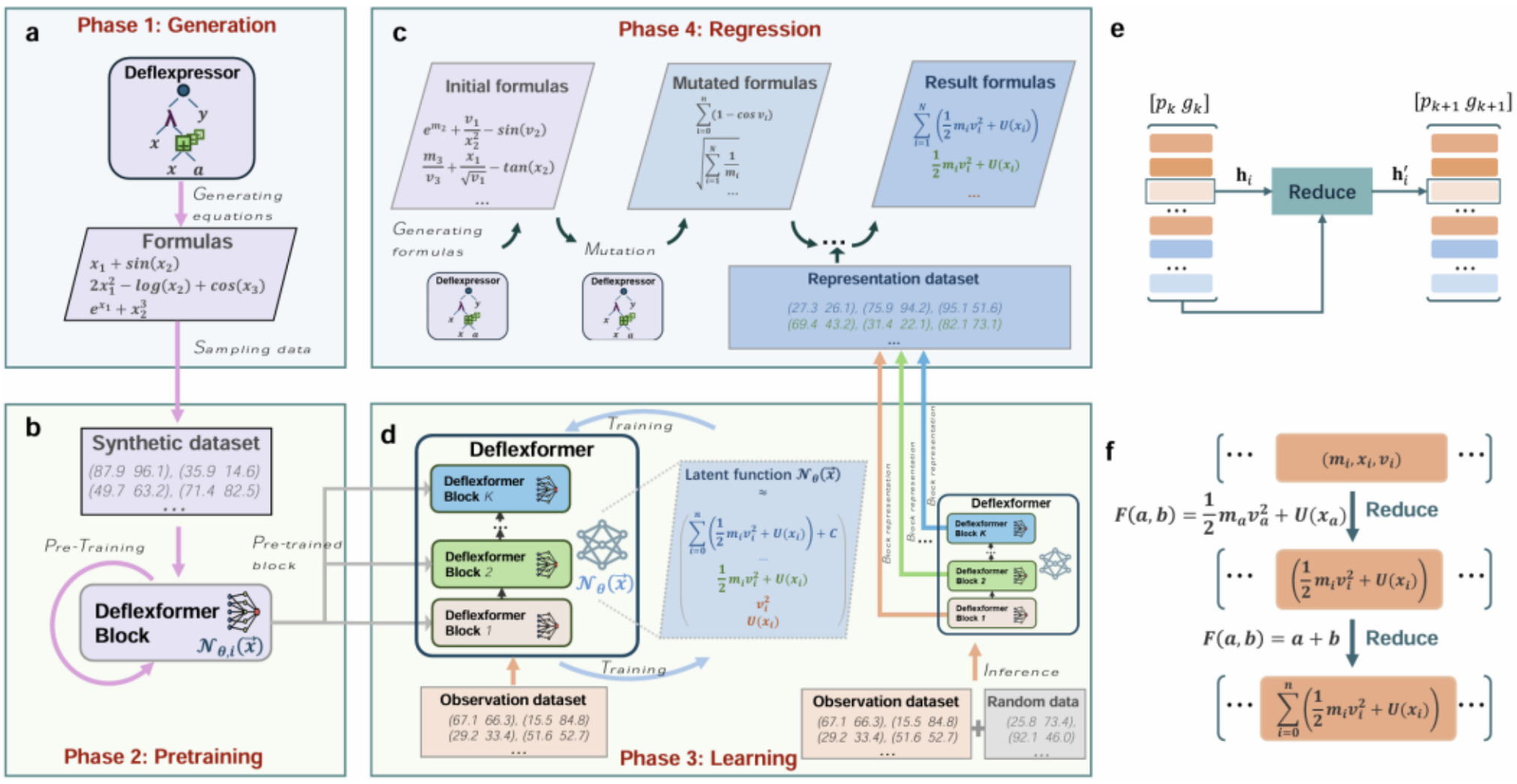

Fig. 3 Illustration of the workflow with 4 phases in Deflex. Deflex utilizes two subsystems: Deflexpressor and Deflexformer, where the former is a symbolic regression subsystem based on Lambda calculus and the latter is an energy-based DNN subsystem. In Phase 1 (**a**), Deflexpressor generates a large number of expressions, from which it samples a synthetic dataset (**a**). In Phase 2 (**b**), the synthetic dataset is used to pre-train a Deflexformer block, a Transformer-like neural network. In Phase 3 (**d**), the pre-trained block is repeatedly cascaded multiple times to build Deflexformer, which is then trained to learn latent formulas on the observation dataset in the target complex system. The trained Deflexformer is then fed with the observation dataset and some random data for inference, which generates a large number of data representations output by all blocks. In Phase 4 (**c**), the representation dataset is finally fit by Deflexpressor to obtain the desired formulas through the process of formula generation and multiple mutations. **e** shows the inference process of Deflexpressor with the Reduce process. **f** shows the evaluation process of Deflexpressor for the total energy of particles system.

Original Manuscript:

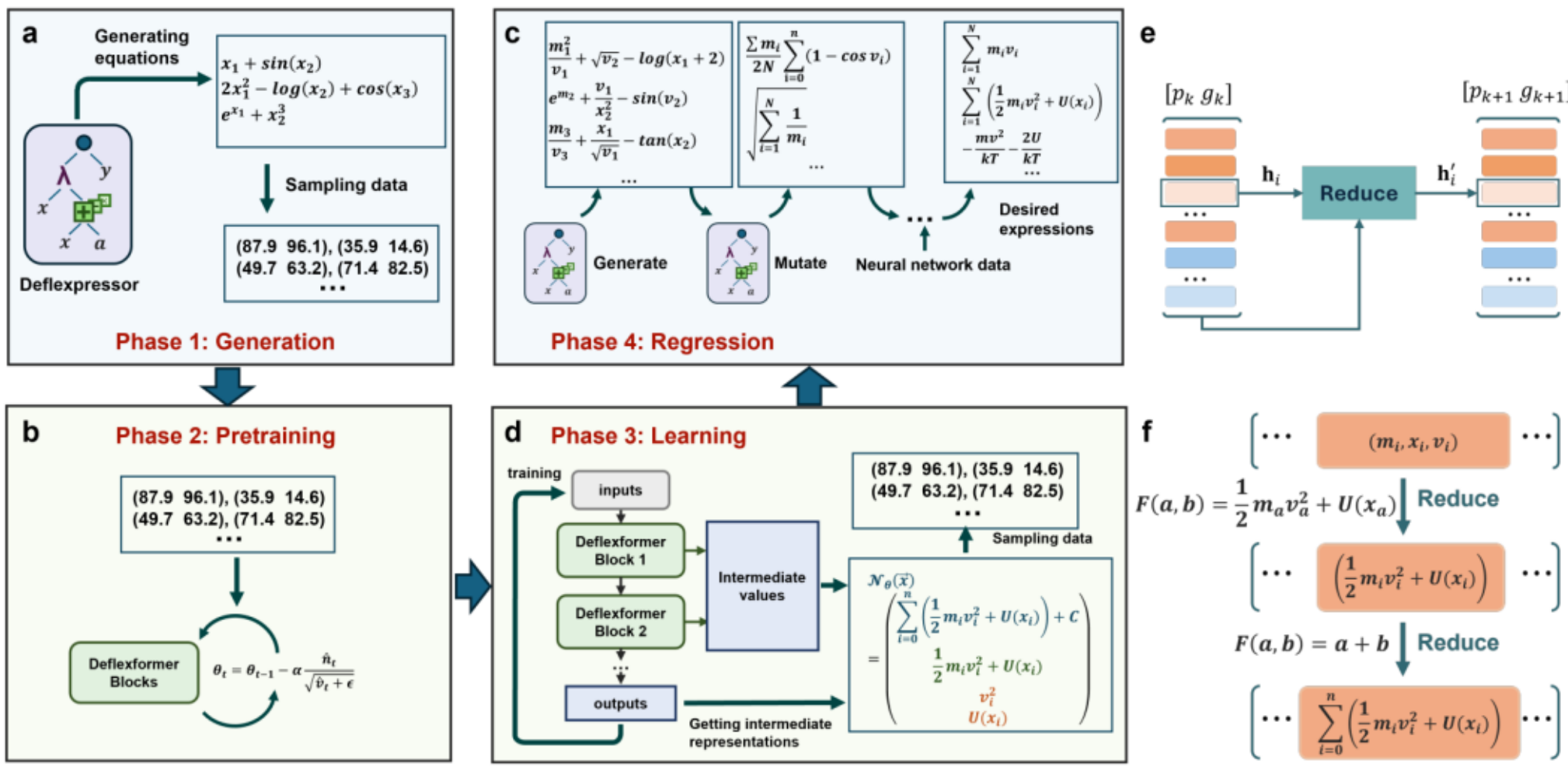

Fig. 3 Illustration of the workflow in Deflex. **a-d** illustrate the process of Deflex, where **a,d** are the symbolic subsystem, Deflexpressor and **b,c** are the DNN subsystem, Deflexformer. At phase 1, Deflexpressor generates a large number of expressions and corresponding data (**a**), which is then used to pre-train the Deflexformer model at phase 2 (**b**). After that, mathematic patterns are learned by Deflexformer at phase 3 (**d**). Finally, Deflexpressor learns mathematical formulas from the knowledge acquired by Deflexformer **c**. **e** shows the inference process of Deflexpressor with the Reduce function. **f** shows the Deflexpressor inference of total energy of particles.

2. We have revised the corresponding paragraphs in Section 1 of the revised manuscript to

highlight the overall workflow.

Revised Manuscript (Pages 3 and 4):

The workflow of our approach is depicted in Fig. 3. First, Deflexpressor generates a large number of formulas for sampling a synthetic dataset (Fig. 3a). The synthetic dataset is then used to pre-train a Deflexformer block, which is a Transformer-like

neural network (Fig. 3b). This pre-training serves as prior knowledge for the subsequent step, where we cascade the pre-trained block multiple times as the whole Deflexformer to learn patterns from the observation dataset of a certain complex system (Fig. 3d). During learning, Deflexformer employs element-wise and time-wise self-attention mechanisms to capture the interactions among different entities and time frames. Each trained block then essentially represents a distinct component of the latent formulas of the patterns. In this phase, the observation dataset, together with some random data, is then fed to the trained Deflexformer for inference, which generates a large volume of data representations output by all blocks. Finally, the representation dataset output by the elementary blocks in Deflexformer is fit with the Deflexpressor to obtain the desired formulas from each component, through formula generation and multiple mutations in the form of lambda expressions (Fig. 3c).

Original Manuscript:

The workflow of our approach is depicted in Fig. 3. First, Deflexpressor generates a large number of symbols for pre-training Deflexformer blocks (Fig. 3a). This pre-training serves as prior knowledge for the subsequent step, where we employ the trained Deflexformer to learn the patterns of complex systems (Fig. 3b). Different Deflexformer blocks represent a distinct component of expressions, and the element-wise and time-wise self-attention mechanisms are used to capture the interactions among different entities and time frames (Fig. 3d). Finally, the outputs of various blocks are sampled as data points, which are fit with Deflexpressor to obtain the desired formulas (Fig. 3c.)

3. We have revised both Subsections 4.3 and 4.5 of the revised manuscript to better clarify the details of our method.

Revised Manuscript (Pages 14 and 15):

4.5 Overall Workflow

Based on the above formulation and subsystem architectures, our approach is depicted in Fig. 3. First, the Deflexpressor generates a large number of formulas and randomly samples some data points to generate a synthetic dataset (Fig. 3a). The synthetic dataset is then used to pretrain a Deflexformer block (Fig. 3b), which is a Transformer-like neural network (shown in Fig. 2). Note that the element-wise and time-wise self-attention mechanisms in the Transformer architecture can effectively capture the interactions among a large volume of different entities and across multiple time frames. This pretrained block serves as prior knowledge for the subsequent step, where the block is chained multiple times to build a Deflexformer network. Deflexformer is then trained on the observation dataset to learn the latent formulas in the targeted complex system (Fig. 3d). Different blocks in the trained Deflexformer essentially represent distinct components of the latent formulas. By taking the observation data and some random data as input, the trained Deflexformer can generate a representation dataset, as shown in Fig. 3d). The representation dataset contains all the data representations output by different blocks. Suppose there are K blocks in Deflexformer, then we denote P_k and G_k the element-level and global-level data representations output by the k-th blocks (Fig. 2d). Then, the input-output representation pairs of one or multiple blocks (e.g., $[P_{k-l}G_{k-l}]$ as the input and $[P_kG_k]$ as the output) for the blocks indexed by $\{k-l,\ldots,k\}$ are taken as the dataset to fit using symbolic regression and discover the formulas learned by the corresponding blocks (Fig. 3c).

In the pre-training stage, Deflexpressor randomly generates a large number of Lambda calculus expressions(Fig. 3a). Note that these expressions should be mathematically concise. With random input data, these expressions can further generate corresponding output data. These input-output pairs are then collected to pretrain a

Deflexformer block, which can learn the basic mathematical transformations as prior knowledge (Fig. 3b).

Formally, each pretrained Deflexformer block takes element-level and global-level representations (the raw data or output of other blocks) P and G as input, and outputs a decomposed representation. Specifically, P is initialized as the observation data $P_0 = \mathbf{X}$, and G is randomly initialized as G_0 according to a Gaussian distribution. Then, the representation $[P_0 G_0]$ is fed into Deflexformer from the first block, where each element of them undergoes the point-wise transformation network, and the cross-attention fusion network [51]. The k-th Deflexformer block transforms the element-wise representation P_{k-1} and global representation P_{k-1} into P_k and G_k respectively, representing a basic mathematical transformation, such as sin, sum in a complex formula. After being transformed by a total of K consecutive blocks in Deflexformer, we obtain the final representations P_K and G_K. We can save all the intermediate representations $[P_0 G_0], [P_1, G_1], \ldots, [P_k G_k], \ldots, [P_K G_K]$ to facilitate arbitrary decomposition of problems in symbolic regression (Fig. 2a).

Specifically, after training Deflexformer on the observation data of the targeted complex system, the raw data and the intermediate representations of all blocks, i.e., $[P_0 G_0], [P_1, G_1], \ldots, [P_k G_k], \ldots, [P_K G_K]$, are fed into Deflexpressor for symbolic regression. In particular, the Deflexpressor subsystem takes the k-th intermediate representations $[P_k G_k]$ as the input features, and $[P_{k+l} G_{k+l}]$ as the label to perform symbolic regression, where l is the number of the Deflexformer blocks for Deflexpressor to regress. Commonly, l is set to be one, such that Deflexpressor can regress the formula of the input-output pair of one Deflexformer block. The Deflexpressor generates a set of Lambda calculus expressions and updates the population of expressions by the genetic algorithm [53]. The set of expressions that is Pareto-optimal in terms of the tradeoff between the expression complexity and the fitness of the data is then selected as the final formulas (Fig. 3d).

To find the Pareto-optimal set, we apply a Reduce process to each candidate expression f to evaluate its data fitness efficiently. Specifically, we apply f on two input data points as follows. First, we randomly take a data point $\mathbf{h}_i$ with the shape $(n \times t + c, d)$ from a dataset $\mathbf{H} = \{\mathbf{h}_0, \mathbf{h}_1, \ldots, \mathbf{h}_k, \ldots, \mathbf{h}_{k+l}\}$ where $\mathbf{h}_k = [p_k \; g_k]$ and $p_k \in P_k$ and $g_k \in G_k$, as the first input. Then, we take another one $\mathbf{h}_j \in \mathbf{H}$ $(j \neq i)$ as the second input and apply $f(\mathbf{h}_i, \mathbf{h}_j)$ to calculate the result denoted as $\mathbf{s}$, i.e., $\mathbf{s} \leftarrow f(s, \mathbf{h}_{j+1})$. The f is then applied to the third data point $\mathbf{h}_k \in \mathbf{H}$ $(k \neq i, k \neq j)$ to update $\mathbf{s} \leftarrow f(\mathbf{s}, \mathbf{h}_k)$. The process is repeated until all data points in $\mathbf{H}$ have been input to f. Note that f can be in various forms. For instance, it can be $f(a, b) = a + b$, and then the Reduce process behaves like a summing operation. f can also be independent of the second input, and then the Reduce process works like a mapping process (Fig. 3f provides an example). Finally, we compute the NLL loss between the final output $\mathbf{s}$ and the Deflexformer block's output $[p_{k+l} \; g_{k+l}]$, which reflects the data fitness of f and ideally should be as small as possible. This Reduce process enables modeling complex higher-order interactions among data and significantly accelerates the evaluation of expressions, thus allowing symbolic regression on the representation pair of one or several continuous Deflexformer blocks and producing the desired formulas.

Original Manuscript:

4.5 Overall Workflow

Based on the above formulation and subsystem architectures, our approach is depicted in Fig. 3. First, the Deflexpressor generates a large number of symbols for pre-training Deflexformer blocks (Fig. 3a). This pre-trained model serves as prior knowledge for the subsequent step, where it learns the patterns of complex systems (Fig. 3b). Different Deflexformer blocks represent distinct components of expressions, and the element-wise and time-wise self-attention mechanisms are used to capture the interactions among different entities and across time frames (Fig. 3d). Finally, the input-output pairs of various blocks (p_{k-1}, g_{k-1} and p_k, g_k as in Fig. 2a) are sampled as data points, and fit using symbolic regression for obtaining desired formulas (Fig. 3c).

In the pre-training stage, Deflexpressor randomly generates a large number of concise Lambda calculus expressions(Fig.3a), and collect the input-output pairs of these expressions with random input data. These pairs are then used to pre-train the Deflexformer blocks, to learn the basic mathematical transformations (Fig.3b).

Pretrained Deflexformer takes element-level and global-level representations P and G as input and outputs the decomposed representations. Specifically, P is initialized as the observation data $p_0 = \mathbf{x}$, and G are randomly initialized as g_0 from a Gaussian distribution. Then, both are fed into the Deflexformer network, where each element undergoes the pointwise transformation network, and the cross-attention fusion network [42]. Each Deflexformer block transforms the element-wise representation p_k and global representation g_k into p_{k+1} and g_{k+1}, representing a basic mathematical transformation, such as sin, sum in complex formula. After m steps through Deflexformer, we obtain the final result p_m and g_m. We save each intermediate parameter p_k and g_k to facilitate the decomposition of problems in symbolic regression (Fig. 2a).

After the training of Deflexformer, the raw data and the intermediate representations p_k and g_k are fed into the Deflexpressor for symbolic regression. Specifically, Deflexpressor subsystem takes the intermediate representations p_k and g_k as the input, and p_{k+l} and g_{k+l} as the label to perform symbolic regression, where l is the number of the Deflexformer blocks for Deflexpressor to regress. Commonly, l is set to be one, such that Deflexpressor can regress the formula of the input-output pair of one Deflexformer block. The Deflexpressor generates a set of Lambda calculus expressions and updates the population of expressions by genetic algorithm[44], the pareto optimal set of expressions are then selected as the final formulas (Fig. 3d).

The expressions are ultimately applied via a Reduce function to a set of data points in the same format as the input-output pairs of the Deflexformer blocks (as shown in Fig. 3e). The Reduce function operates by taking a data point $\mathbf{h}_i$ from $\mathbf{h} = [p_k\ g_k]$, an array of shape $(n \times t + c, d)$ as the initial input. Then it takes another point $data_j$ as a second input and runs $s \leftarrow F(\mathbf{h}_i, \mathbf{h}_j)$. The process continues to the next data point $\mathbf{h}_{j+1}$ calculating $s \leftarrow F(s, \mathbf{h}_{j+1})$, and repeats this until all data points, except $\mathbf{h}_i$, have been processed. The final output $\mathbf{h}'_i$ of the Reduce function corresponds to $\mathbf{h}_i$ is the last s, and the overall output $\mathbf{h}'$ should fit the Deflexformer block output $[p_{k+l}\ g_{k+l}]$. When $F(a, b) = a + b$, the Reduce operation behaves as a summing operation. On the contrary, when F is independent of the second input, the Reduce operation works like

a map (Fig. 3f provides an example). This enables the modeling of complex higher-order interactions between data, allowing symbolic regression on the input-output pair of one or several continuous Deflexformer blocks and producing the desired formula F.

By performing symbolic regression block-by-block instead of directly regressing symbolic relationships, we can reduce the exponential complexity to a linear one. After the block-wise symbolic regression, the block-wise Reduce expressions are combined into a single long expression, which is then further used to perform symbolic regression on the entire Deflexformer's input and output, yielding more precise and concise expressions.

Comment 3.2:

Nevertheless, before this can be published, the authors definitively need to analyse the performance of the algorithm in further detail and document

- *intermediate results (what do we get after each step of the algorithm).*
- ***results on international benchmark data sets.***
- *comparisons with other state of the art algorithms used on the same data sets.*

Our Response:

We thank the reviewer for this comment. We fully agree that it is important to evaluate our method on the international benchmark datasets for a comprehensive performance assessment. In our revised manuscript, we have included the additional international benchmark dataset named Feynman Symbolic Regression Database [1, 2, 3, 4] (publicly available at https://space.mit.edu/home/tegmark/aifeynman.html) by conducting extensive comparison experiments and analysis with FIVE state-of-the-art methods.

We would like to clarify that we did not have such results in our original submission because our work primarily focuses on multi-scale complex systems, which have no well-known benchmark datasets. Despite this, our work also works for single-scale systems as other existing AI methods. Therefore, considering that most of current AI-based discovery methods rely on symbolic regression, we have included the standard AI Feynman benchmark dataset in our revision. AI Feynman benchmark dataset is openly available from the widely recognized SRBench suite and has been commonly used in [1, 2, 3, 4]. It is generated by sampling from 120 classical formulas in physics, with added noise to simulate real-world conditions. It captures essential characteristics of real-world mathematical formulas, such as symmetry, while also being a classic, well-established set of problems for symbolic regression. This dual characteristic facilitates a feasible performance comparison against other SR-based AI methods.

Based on this benchmark dataset, we have not only conducted additional experiments to evaluate Deflex's performance, but also implemented other state-of-the-art methods for comparison (Please refer to **Response to Comment 3.3** for more details). Particular, the compared methods on this benchmark dataset include Operon, SciMED, gplearn, AI Feynman 2.0, and PySR. We compared their performance by measuring the trade-off between the RMSE of discovered formulas and the total runtime for discovery. The result on the public dataset demonstrates Deflex has quite competitive performance among the existing methods, which validates the effectiveness and efficiency of our proposed method.

To respond this comment, we have conducted following modifications in the revised manuscript.

1. We have added Subsection 2.7, with details of the added public dataset and the setup of the corresponding additional experiment.

Revised Manuscript (Page 9):

2.7 Comparison on Benchmark Dataset

We finally evaluated Deflex on the standard AI Feynman benchmark dataset [24]. This benchmark dataset is generated by sampling from 120 classical formulas spanning different fields of physics, with added noise to simulate real-world conditions. It captures essential characteristics of real-world mathematical formulas, such as symmetry, while also being a classic, well-established set of problems for symbolic regression. This dual characteristic facilitates a feasible performance comparison against other SR-based AI methods. In the experiment, Deflex was compared against Operon, SciMED, gplearn, AI Feynman 2.0, and PySR. We compared their performance by measuring the trade-off between the RMSE of discovered formulas and the total runtime for discovery. Note that SINDy was not included as it is sparse regression-based and not designed for symbolic regression tasks, while DEAP was omitted since it works as the backend of gplearn in the current task.

As shown in Fig. 4d, Deflex demonstrates a quite competitive performance among all compared methods on the benchmark dataset in terms of the accuracy-efficiency tradeoff, although it is not specialized for these traditional, closed-form equations. In particular, Deflex is only slightly outperformed by Operon, which relies on a fixed symbol set and is optimized for runtime. In our experiment, Deflex managed to discover the formula with an RMSE of 0.075 in just 146.2 seconds, which was quite close Operon with an RMSE of 0.0663 in 135.8 seconds. And Deflex and Operon showed significant advantages over other methods.

2. We have added the corresponding experimental results on this benchmark dataset in Fig. 4.

Revised Manuscript (Page 25):

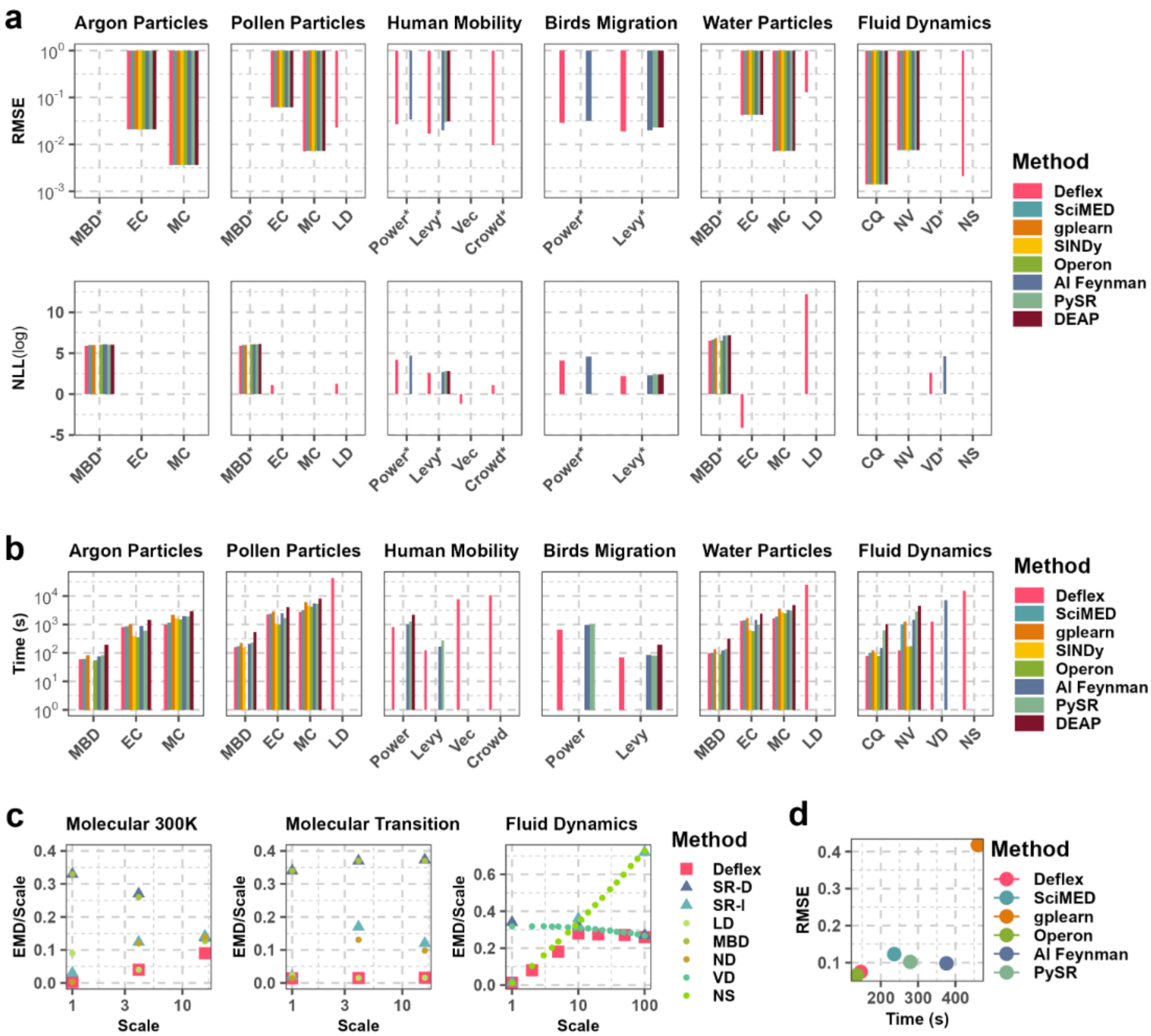

Fig. 4 Experimental results of Deflex in terms of accuracy (**a**), efficiency (**b**), and scalability (**c**). **a** compares the RMSE and NLL loss of the discovered formulas by Deflex and other methods in the experiments of particle motions, human and bird collective movement, water particles, and fluid dynamics. The abbreviations represent the Maxwell-Boltzmann distribution (MBD), energy conservation law (EC), momentum conservation (MC), Langevin dynamics (LD), continuity of quality(CQ), Newton viscosity law (NV), velocity distribution in fluid dynamics(VD), Navier-Stokes equation (NS), power law in human mobility (Power), Levy flight rule (Levy), vector navigation law (Vec), and crowd power law, respectively. Among them, the formulas in the form of distributions are marked with $*$. The absence of the method indicates that the corresponding metrics are not available for the method. **b** depicts the running time and usability of Deflex compared with other methods in different experiments. The horizontal axis represents the running time of the methods and the absence of the method indicates it is infeasible to discover the formula. Only Deflex can discover all the formulas in the experiments, and the time consumption of Deflex is significantly lower than that of most other methods for complex formulas. **c** shows the EMD values of known formulas and formulas discovered by Deflex and other methods across different coarse-graining scales for water particles' motion and fluid dynamics, where "SR-I" and "SR-D" represent the symbolic regression methods with invariant and distributional form settings, respectively. **d** shows the running time and relative mean square error for Deflex and other methods on the public benchmark dataset of Feynman Symbolic Regression Database.

Original Manuscript:

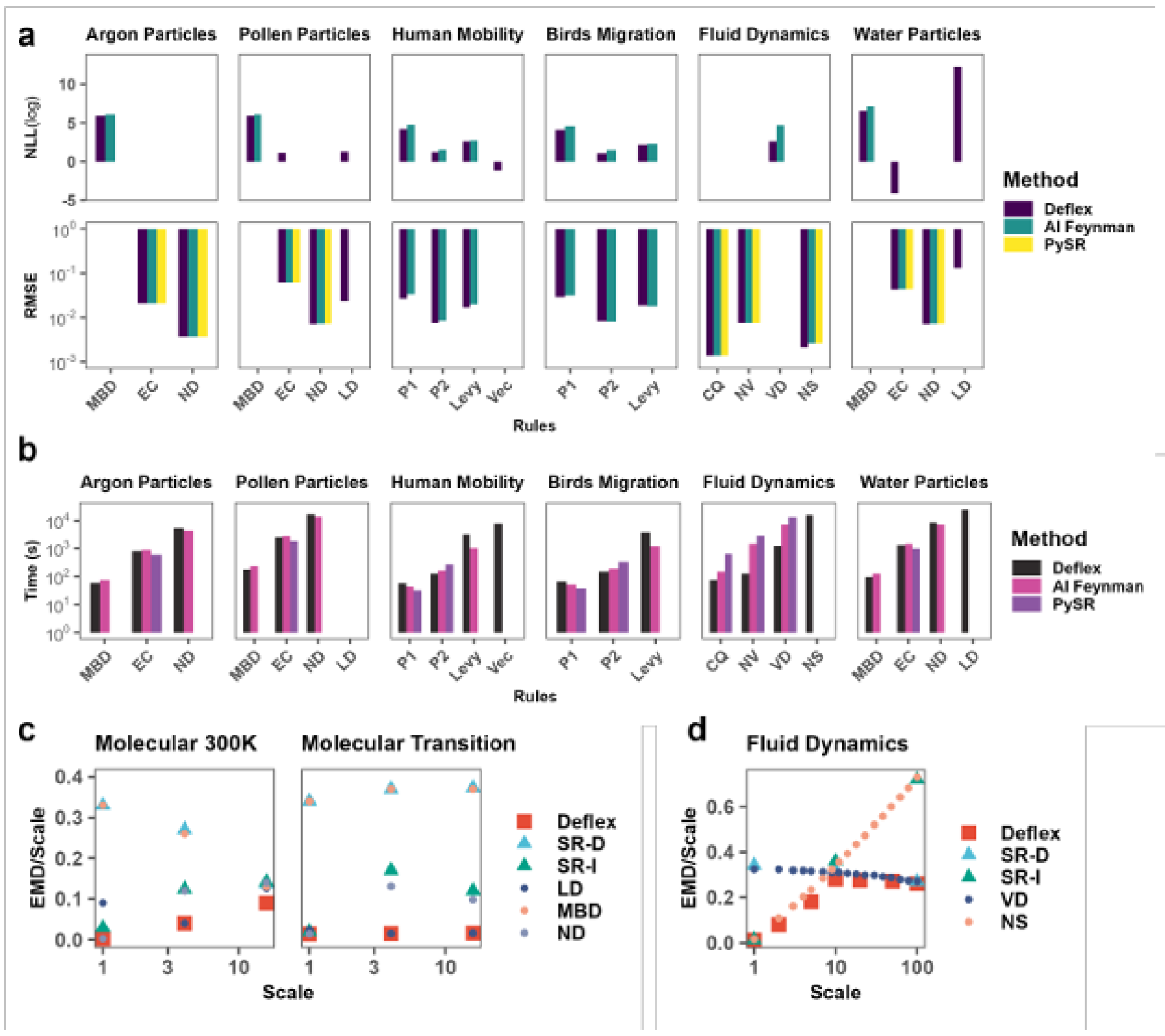

Fig. 4 Experimental results of Deflex in terms of accuracy (a), efficiency (b), and scalability (c). **a** compares the RMSE and NLL loss of the discovered formulas by Deflex and other methods in the experiments of particle motions, human and bird collective movement, fluid dynamics, and water particles. The abbreviations represent the Maxwell-Boltzmann distribution (MBD), energy conservation law (EC), Newtonian dynamics (ND), Langevin dynamics (LD), continuity of quality(CQ), Newton viscosity law(NV), velocity distribution in fluid dynamics(VD), Navier-Stokes equation (NS), first and second power law in human mobility(P1 and P2), Levy flight rule(Levy), and vector navigation law (Vec), respectively. The absence of the method indicates that the corresponding metrics are not available for the method. **b** depicts the running time and usability of Deflex compared with other methods in different experiments. The horizontal axis represents the running time of the methods and the absence of the method indicates it is infeasible to discover the formula. Only Deflex can discover all the formulas in the experiments, and the time consumption of Deflex is significantly lower than that of other methods for complex formulas. **c** and **d** shows the EMD values of known formulas and formulas discovered by Deflex and other methods across different coarse-graining scales for water particles motion (**c**) and fluid dynamics (**d**), where "SR-I" and "SR-D" represent the symbolic regression methods with invariant and distributional form settings, respectively.

References of the response to this comment

[1] *Udrescu, Silviu-Marian, Andrew Tan, Jiahai Feng, Orisvaldo Neto, Tailin Wu, and Max Tegmark. 2020. "AI Feynman 2.0: Pareto-Optimal Symbolic Regression Exploiting Graph Modularity." Pp. 4860–71 in Advances in Neural Information Processing Systems. Vol.* ***33****, edited by H. Larochelle, M. Ranzato, R. Hadsell, M. F. Balcan, and H. Lin. Curran Associates, Inc.*

[2] *Udrescu, S.-M., Tegmark, M.: Ai feynman: A physics-inspired method for symbolic regression. Science Advances* ***6****(16), 2631 (2020) https://doi.org/10.1126/ sciadv.aay2631.*

[3] *Kamienny, P., d'Ascoli, S., Lample, G. & Charton, F. End-to-end Symbolic Regression with Transformers. Advances in Neural Information Processing Systems* ***35****, 10269–10281 (2022).*

[4] *Grayeli, A., Sehgal, A., Reyes, O. C., Cranmer, M. & Chaudhuri, S. Symbolic Regression with a Learned Concept Library. in (2024).*

[5] *https://space.mit.edu/home/tegmark/aifeynman.html*

Comment 3.3:

Nevertheless, before this can be published, the authors definitively need to analyse the performance of the algorithm in further detail and document

- *intermediate results (what do we get after each step of the algorithm).*
- *results on international benchmark data sets.*
- ***comparisons with other state of the art algorithms used on the same data sets.***

Response to Comment 3.3:

The authors thank the reviewer for this comment. We fully agree that comparison to state-of-the-art algorithms can present a comprehensive evaluation of our proposed method. We have conducted extensively new experiments by comparing Deflex with FIVE additional state-of-the-art algorithms, including SINDy, SciMED, gplearn, DEAP, and Operon, in ALL SIX complex systems and one additional public dataset.

SINDy is a sparse regression-based, while SciMED, gplearn, and DEAP are all tree-based symbolic regression methods with GP. Among them, DEAP and GPLearn are classic SR frameworks based on evolution algorithm, while SciMED is a recent physics-inspired method based on Monte Carlo tree searching. Besides these suggested ones, we have also included Operon found by ourselves, which has been one of the SOTA symbolic regression method according to SRBench. These are all relevant work to us, so we have undertaken a significant expansion of experimental comparisons, including on the benchmark dataset suggested by the reviewer. Please note that, since these baselines were not designed for multi-scale systems, the comparison experiment were conducted on single-scale scenarios where the goal is to discovery the relationships between scale-specific variables.

Overall, we have added extensive comparison results on all datasets and systems. Specific modifications in the revised manuscript are as follows.

1. We have added detailed description of the compared methods in the revised manuscript.

Revised Manuscript (Page 4-5):

2.1 Compared Methods

We benchmarked our approach against several leading AI methods for formula discovery, including state-of-the-art SR method Operon [31], typical physics-inspired SR method SciMED [26], leading sparse regression method SINDy [22], and AI Feynman 2.0 [8], as well as classic SR methods gplearn [32], PySR [33] and DEAP [34] in terms of accuracy, efficiency, and adaptability to multiscale systems.

Different from Deflex with energy models and lambda calculus, existing AI methods (mainly based on symbolic regression) often support limited forms of problems and formulas. To maximize comparability, we made several accommodations for these compared methods. For the comparatives (PySR, AI Feynman, and DEAP) lacking native support for high-order operators (e.g., map or sum), we added these operators to their symbol set manually. For the rest ones (Operon, SciMED, SINDy, and gplean) that are incompatible with these operators, we provided an alternative. That is, if such an operator was at the top level of the target formula, we applied the method for the sub-expressions only and then handled the top-level operators within the loss function manually. Similarly, for the methods unable to approximate distributions, we provided custom energy-based loss functions. A method was regarded as failing in a task if it still could not discover the rule after these best-effort accommodations.

Original Manuscript:

2 Results

We evaluate the effectiveness and efficiency of Deflex in six representative complex systems with different behaviors. Particularly, these systems can be categorized into the simulation experiments, including particle motion, two-phase water particles, and fluid dynamics, and the real-world data of human and bird collective movements. We compare the performance of Deflex with the state-of-the-art AI-based formula discovery algorithms, including AI Feynman 2.0 [8] and PySR [24], in terms of accuracy, efficiency, and adaptability to multi-scale systems.

2. We have updated Fig. 4 in the revised manuscript to include the additional comparison results with SINDy, SciMED, gplearn, DEAP, and Operon.

Revised Manuscript (Page 25):

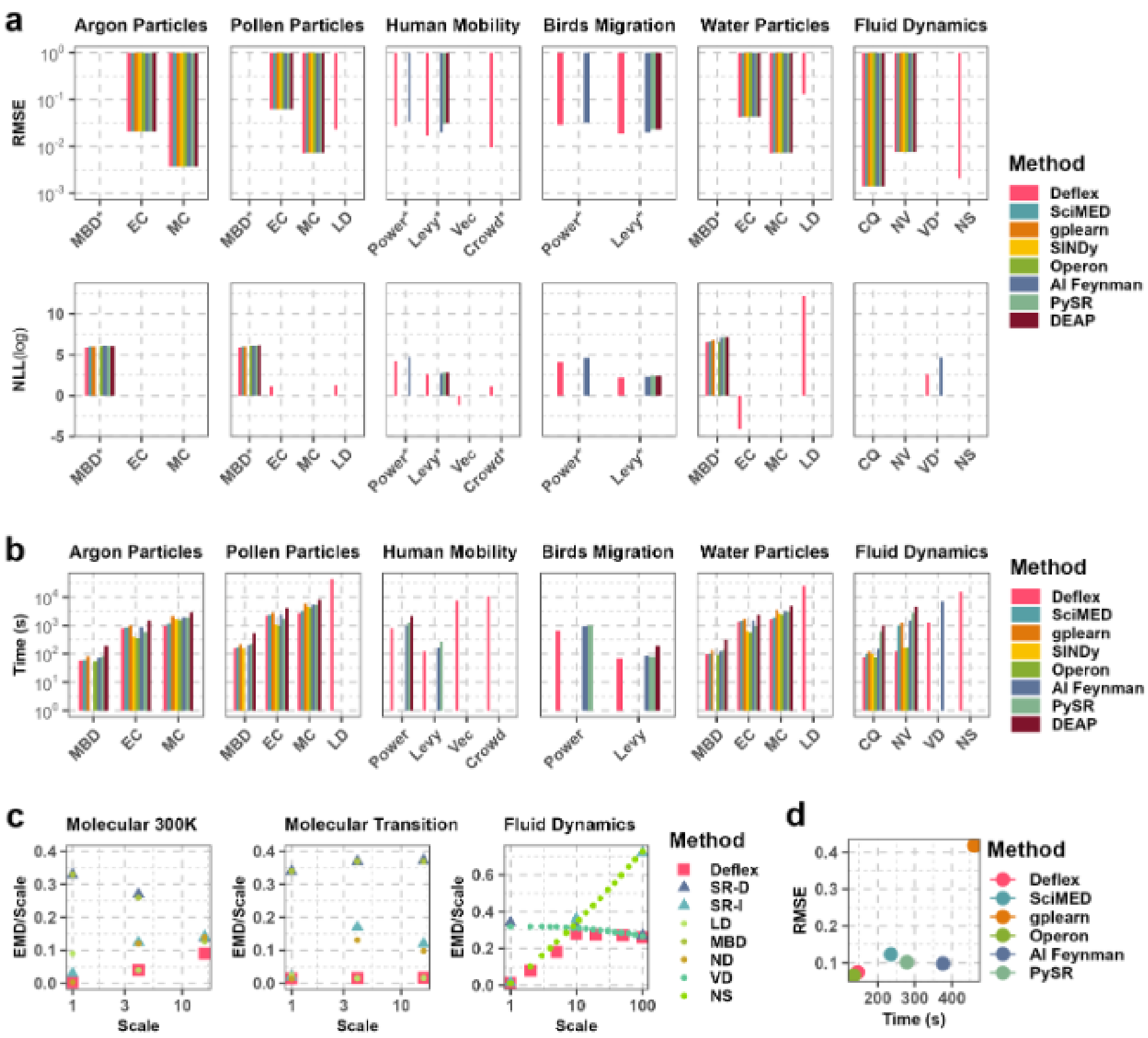

Fig. 4 Experimental results of Deflex in terms of accuracy (**a**), efficiency (**b**), and scalability (**c**). **a** compares the RMSE and NLL loss of the discovered formulas by Deflex and other methods in the experiments of particle motions, human and bird collective movement, water particles, and fluid dynamics. The abbreviations represent the Maxwell-Boltzmann distribution (MBD), energy conservation law (EC), momentum conservation (MC), Langevin dynamics (LD), continuity of quality(CQ), Newton viscosity law (NV), velocity distribution in fluid dynamics(VD), Navier-Stokes equation (NS), power law in human mobility (Power), Levy flight rule (Levy), vector navigation law (Vec), and crowd power law, respectively. Among them, the formulas in the form of distributions are marked with $*$. The absence of the method indicates that the corresponding metrics are not available for the method. **b** depicts the running time and usability of Deflex compared with other methods in different experiments. The horizontal axis represents the running time of the methods and the absence of the method indicates it is infeasible to discover the formula. Only Deflex can discover all the formulas in the experiments, and the time consumption of Deflex is significantly lower than that of most other methods for complex formulas. **c** shows the EMD values of known formulas and formulas discovered by Deflex and other methods across different coarse-graining scales for water particles' motion and fluid dynamics, where "SR-I" and "SR-D" represent the symbolic regression methods with invariant and distributional form settings, respectively. **d** shows the running time and relative mean square error for Deflex and other methods on the public benchmark dataset of Feynman Symbolic Regression Database.

Original Manuscript:

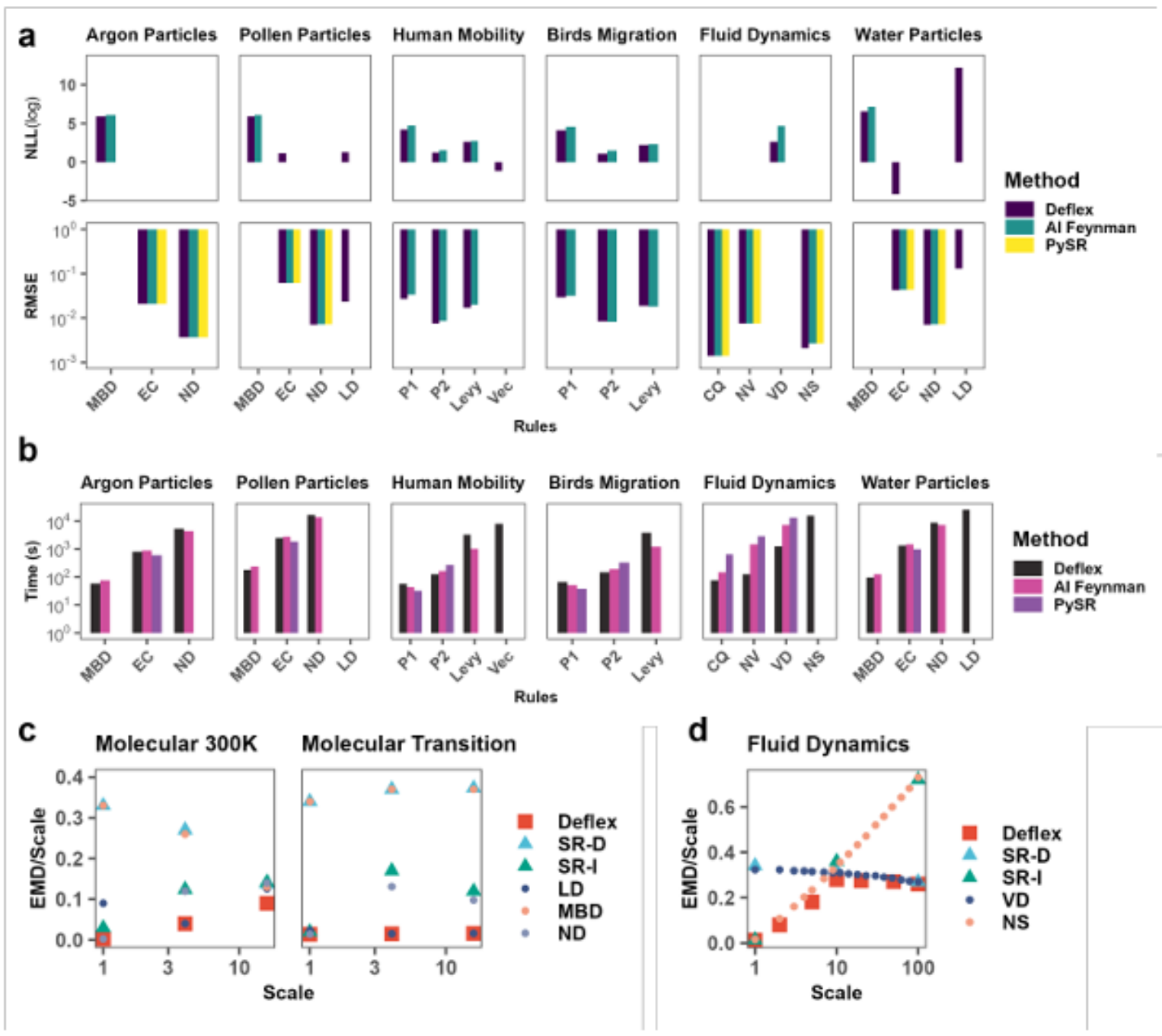

Fig. 4 Experimental results of Deflex in terms of accuracy (**a**), efficiency (**b**), and scalability (**c**). **a** compares the RMSE and NLL loss of the discovered formulas by Deflex and other methods in the experiments of particle motions, human and bird collective movement, fluid dynamics, and water particles. The abbreviations represent the Maxwell-Boltzmann distribution (MBD), energy conservation law (EC), Newtonian dynamics (ND), Langevin dynamics (LD), continuity of quality(CQ), Newton viscosity law(NV), velocity distribution in fluid dynamics(VD), Navier-Stokes equation (NS), first and second power law in human mobility(P1 and P2), Levy flight rule(Levy), and vector navigation law (Vec), respectively. The absence of the method indicates that the corresponding metrics are not available for the method. **b** depicts the running time and usability of Deflex compared with other methods in different experiments. The horizontal axis represents the running time of the methods and the absence of the method indicates it is infeasible to discover the formula. Only Deflex can discover all the formulas in the experiments, and the time consumption of Deflex is significantly lower than that of other methods for complex formulas. **c** and **d** shows the EMD values of known formulas and formulas discovered by Deflex and other methods across different coarse-graining scales for water particles motion (**c**) and fluid dynamics (**d**), where "SR-I" and "SR-D" represent the symbolic regression methods with invariant and distributional form settings, respectively.

3. We have added a table to compare the capability of different methods for discovering the formulas in the six complex systems studied.

Revised Manuscript (Page 21):

Table 2 Comparison of methods on discovery capability

Rules	Deflex	Operon	SciMED	SINDy	gplearn	AI Feynman	PySR	DEAP
Energy conservation	✓	✓	✓	✓	✓	✓	✓	✓
Momentum conservation	✓	✓	✓	✓	✓	✓	✓	✓
MBD	✓	✓	✓	N.A.	✓	✓	✓	✓
MBD with potential	✓	N.A.	N.A.	N.A.	✗	N.A.	N.A.	✗
Continuity of quality	✓	✓	✓	✓	✓	✓	✓	✓
Newton viscosity law	✓	✓	✓	✓	✓	✓	✓	✓
Velocity distribution	✓	✗	✗	N.A.	✗	✓	✗	✗
Navier-Stokes equation	✓	✗	✗	✗	✗	✗	✗	✗
Langevin	✓	✗	✗	N.A.	✗	✗	✗	✗
Levy flight (Human)	✓	✗	✗	N.A.	✗	✓	✓	✓
Levy flight (Avian)	✓	✗	✗	N.A.	✗	✓	✓	✓
Power law	✓	✗	✗	N.A.	✗	✓	✗	✗
Crowd power law	✓	N.A.	N.A.	N.A.	✗	N.A.	N.A.	✗

✓ denotes a successful discovery, ✗ denotes a failure, and N.A. denotes an unsupported task (i.e., not applicable).

4. We have revised multiple places to analyze the new comparison results in Sec. 2 "Results" of the revised manuscript.

Revised Manuscript (Page 6-7):

As shown in Table 1, Deflex successfully identified a series of formulas in these particle motion systems, including the energy conservation, Boltzmann distribution, Maxwell-Boltzmann distribution (MBD), MBD with Lennard-Jones potential, and the Langevin equation. In particular, MBD with Lennard-Jones potential and the Langevin equation cannot be discovered by other compared methods in our experiments (shown in Table 2). And those tree-based symbolic regression method requires manual specification MBD with Lennard-Jones potential identified by Deflex in the dynamics of rare gas particles is a nonlinear equation that consists of up to 23 nodes, demonstrating Deflex's ability to discover complex nonlinear equations.

On the commonly identified Maxwell-Boltzmann distribution, Deflex managed to achieve the least NLL loss than the compared methods in both Argon and Pollen particles (shown in Fig. 4a). Meanwhile, the RMSEs of all compared methods are nearly the same on both energy conservation and momentum conservation in the two systems. This is because the structures of correctly identified formulas are almost identical in these methods, as mentioned before. As shown in Fig. 4b, Deflex incurs significantly less computational time than most of the comparatives. SINDy and Operon are quite close to or sometimes faster than Deflex, but only limited to some lower-order formulas like momentum conservation. This is due to their limitations in formula structures.

Original Manuscript:

As shown in Table 1, Deflex successfully identified the formulas of the energy conservation, Boltzmann distribution, and Maxwell-Boltzmann distribution in both particle motion systems. Langevin dynamic describes the interaction between matters at different scales and it does not hold for rare gas systems. And Deflex managed to manged to achieve higher accuracy than comparative methods, as shown in Fig. 4a. In contrast, PySR and AI Feynman could only discover momentum conservation due to the limited number of elements. Fig. 4b compares the computational effectiveness of Deflex with that of comparative methods in both systems. The computational time of Deflex was significantly lower than that of comparative methods for those formulas discovered in both systems.

Revised Manuscript (Page 7-8):

As presented in Table 1, Deflex identified multiple formulas, including power law equations [40], Lévy flight patterns [41], and vector navigation laws [42] in human crowds, and the Lévy flight patterns in bird migrations. Besides Deflex, AI Feynman, PySR, and DEAP can also discover the power law distribution and Lévy flight pattern, while only AI Feynman can discover the Power law distribution (shown in Table 2). But no other method can identify the vector navigation law. It is worth noting that Deflex also uncovered an undocumented power law distribution,

$$P\left(\Delta x_t^{(i)}\right) \propto r^{\alpha_0-\gamma\sum_{j\neq i}\mathbb{I}\left(\left\|x_t^{(j)}-x_t^{(i)}\right\|<2.46\right)}. \tag{2}$$

This rule may indicate a trend superimposed on the baseline power-law distribution and describe the collective behavior of human crowds: individuals exhibit a higher probability of making a long-distance move as their neighbors do.

For the commonly found rules of the power law distribution and Lévy flight pattern, we first compared the NLL loss and RMSE of Deflex with other methods in Fig. 4a. It shows that Deflex's discovered distributions have consistently high accuracy (both smaller NLL loss and RMSE value) than other capable methods. We also compared their time efficiency for discovering these distributions under the same accuracy (i.e., by setting an identical precision threshold for convergence). Fig. 4b shows that Deflex also leads to the least running time among the compared methods.

Original Manuscript:

The probabilistic forms of the formulas discovered by Deflex are also presented in Table 1. Deflex successfully identified multiple formulas, including power law equations [30], Lévy flight patterns [31], and vector navigation laws [32], in human crowds and the Lévy flight patterns in bird migrations. Furthermore, we compared the NLL loss based on the real data and the MSE loss based on the parameter estimation of the known formulas. The results show that PySR was unable to identify these formulas (Fig. 4b). Although AI Feynman could uncover the normal version of the Lévy flight pattern, it incurs greater errors in either the NLL loss based on real data and the estimated known formulas (Fig. 4a).

Revised Manuscript (Page 8):

For scale-independent characteristics, Deflex demonstrates similar performance in water particles as on Argon and pollen particles, as shown in Table 1, Figs. 4a and 4b. To validate the cross-scale properties, we conducted coarse-graining of water

As shown in Fig. 4c, Deflex managed to capture the cross-scale emergence of Langevin dynamics in water particles. In contrast, other methods were limited to observing molecular dynamics at smaller scales and the dynamics of ice crystals at larger scales, failing to bridge the transition across scales. It also shows that Deflex detected the scale invariance of the system at the phase transition point of water (i.e., at 273.15K).

Original Manuscript:

As shown in Fig. 4, Deflex managed to capture the cross-scale emergence of Langevin dynamics in water particles. In contrast, other methods were limited to observing molecular dynamics at smaller scales and the dynamics of ice crystals at larger scales, failing to bridge the transition between these regimes. It is also shown that the Deflex detects the scale invariance of at phase transitions for water at 273.15K.

Revised Manuscript (Page 8-9):

As listed in Table 1, Deflex detected the Navier-Stokes equation of fluid dynamics through small-scale observations, which cannot be discovered by other methods. In larger-scale random observations, Deflex extracted the velocity distribution of the fluid within the vortex flow. These formulas correspond to different manifestations of long-standing laws in physics. For complex distribution formulas like velocity distribution, most compared methods are infeasible (shown in Table 2), and only AI Feynman and Deflex manage to discover.

Fig.4a also shows that the equation-based formulas discovered by Deflex can achieve a similar RMSE as other methods. For the velocity distribution, Deflex's discovery has a much smaller NLL loss than AI Feynman's, showing its better accuracy. In Fig. 4b, most comparatives, except for SINDy and Operon, become much slower than Deflex with the increase of formula complexity. Among them, AI Feynman is around 10 times slower than Deflex.

In addition, we compared the EMD across different coarse-graining scales of Deflex with that of AI Feynman and the renormalization of known formulas. Fig. 4c shows that predictions from Deflex kept the lowest EMD across different scales, indicating the Deflex's ability to overcome the scale gap issue in complex systems. Meanwhile, other methods with a single setting could only capture the patterns at a single scale, and the EMD increased significantly when the scale changed.

Original Manuscript:

As shown in Table 1, the patterns we obtained correspond to different manifestations of long-standing laws applied in physics. Also shown in Fig.4a, we obtained the Navier-Stokes equation of fluid dynamics through small-scale observations. In larger-scale random observations, we acquired information such as the velocity distribution of the fluid within the vortex flow. Fig. 4b shows the time consumption of different methods. When the complexity of the formulas increases, the PySR and AI Feynman methods became much slower than Deflex (approximately 10× slower for velocity distribution). For very complex formulas including Navier-Stokes equation and Langevin dynamics for particle motion, the PySR and AI Feynman methods became infeasible.

In addition, we compared the EMD across different coarse-graining scales of Deflex with that of AI Feynman and the renormalization of known formulas. Fig. 4d shows that predictions from Deflex kept the lowest EMD across different scales, indicating the Deflex's ability to overcome the scale gap issue in complex systems. Meanwhile, other methods with a single setting could only capture the patterns at a single scale, and the EMD increased significantly when the scale changes.

5. We have further added a comparison experiment on a benchmark dataset as follows (Please refer to **Response to Comment 3.2** for more details).

Revised Manuscript (Page 9):

2.7 Comparison on Benchmark Dataset

We finally evaluated Deflex on the standard AI Feynman benchmark dataset [24]. This benchmark dataset is generated by sampling from 120 classical formulas spanning different fields of physics, with added noise to simulate real-world conditions. It captures essential characteristics of real-world mathematical formulas, such as symmetry, while also being a classic, well-established set of problems for symbolic regression. This dual characteristic facilitates a feasible performance comparison against other SR-based AI methods. In the experiment, Deflex was compared against Operon, SciMED, gplearn, AI Feynman 2.0, and PySR. We compared their performance by measuring the trade-off between the RMSE of discovered formulas and the total runtime for discovery. Note that SINDy was not included as it is sparse regression-based and not designed for symbolic regression tasks, while DEAP was omitted since it works as the backend of gplearn in the current task.

As shown in Fig. 4d, Deflex demonstrates a quite competitive performance among all compared methods on the benchmark dataset in terms of the accuracy-efficiency tradeoff, although it is not specialized for these traditional, closed-form equations. In particular, Deflex is only slightly outperformed by Operon, which relies on a fixed symbol set and is optimized for runtime. In our experiment, Deflex managed to discover the formula with an RMSE of 0.075 in just 146.2 seconds, which was quite close Operon with an RMSE of 0.0663 in 135.8 seconds. And Deflex and Operon showed significant advantages over other methods.

Response Letter

Manuscript NCOMMS-25-26170A

Dear reviewers,

We thank the editor and reviewers for the helpful and insightful comments, which significantly improve the quality of the article. Compared with the original manuscript, considerable modifications have been made in this revision (marked as blue in the revised manuscript and supplementary information), which are summarized as follows:

1. **Detailed Intermediate Results** (Comments 1.3, 1.4, 3.1, 3.2, 4.3, 4.11, 4.15): We have added much more important detailed intermediate results in the experiments, especially the necessary ablation studies to demonstrate the impact of different components. Besides, we have added detailed algorithmic pseudocodes of these components in SI to elaborate the intermediate parameters.
2. **Revised Methodology Description** (Comments 3.1, 4.4, 4.6, 4.7, 4.8, 4.9, 4.10, 4.11, 4.12, 4.13, 4.14, 4.19): We have significantly revised the description of the methods, including more rigorous notations and formulations in the manuscript, clearer charts of the main procedures, and the detailed algorithmic pseudocodes of different components in SI.
3. **Statistical Experimental Analysis** (Comments 3.2, 4.11): We have reported all the statistical values of our experimental results, including the newly added ablation studies, to ensure a sound statistical analysis. We have recreated all related figures to illustrate these analyses.
4. **Clarified Comparison Configurations** (Comments 1.1, 1.2, 1.3, 1.4, 4.2): We have added the detailed configurations of all the experiments, including the scale of provided features of datasets, and the configurations of all compared methods.
5. **Added Experiment Benchmark** (Comments 4.1, 4.5): We have performed the evaluations on an additional benchmark based on reviewers' suggestions, which can better demonstrate the effectiveness and efficiency of our method.
6. **Improved Presentation** (Comments 4.16, 3.17, 4.18, 4.20, 4.21, 4.22): We have carefully proofread both the manuscript and SI to avoid typos and grammar errors, and the readability and presentation have been significantly improved.

Detailed point-by-point responses are as follows.

=======Reviewer #1=======

Comments to the Authors:

I would like to start by complimenting the authors on the detailed revision, which addressed most of my concerns.

Response to Reviewer #1:

We thank the reviewer for the positive comments regarding the topic importance and recognizing the presentations and readability of the manuscript. We also thank the reviewer for later constructive comments and revision suggestions, which help us significantly improve the quality of this article.

Comment 1.1:

*The authors put V on all the equations, while in some this is just not true (for example, D != constant). The last equation is also not V, as there is no baseline, and the latter is not compared numerically. Moreover, I tested the Levy flight (both versions) and SciMED and gplearn was able to do it - which disagrees with the table. SciMED was able to find the Velocity distribution as well. In addition, not sure why it states that SINDy cannot run this equation? D * log(u)? This is as easy as it gets.*

Response to Comment 1.1:

The authors thank the reviewer for this comment. We would like to respond point by point.

1) Regarding the point "... *while in some this is just not true (for example, D != constant)*", we used symbols like *D* to represent an unprovided constant for a given system but vary for different settings. In particular, we have claimed in Subsection 2.2 of the revised manuscript that "*the formulas of invariants are considered successful recovery if they can be expressed as the correct formula with an additional stochastic residual term*", which is commonly adopted by other studies [1]. According to this principle, we believe our result is a successful discovery. <u>To make the presentation more rigor, we have revised Table 1 to highlight all the unprovided constants in the formulas, and added detailed explanations to the provided features in SI. Also, we have provided the complete, unabbreviated forms of the discovered equations in the Supplementary Information.</u>

2) Regarding the point "*The last equation is also not V...* ", we agree that our presentation was not sufficiently precise in the absence of a direct baseline for comparison. <u>To respond this point, we only keep this formula in Table 1 as Deflex's discovery and have removed the corresponding comparison in Table 2.</u>

3) We respectfully do not agree with the comment regarding the distribution discovery capabilities of the existing methods SciMED, gplean, and SINDy. In particular, our problem is defined as directly discovering the probabilistic distributions from the observation system data (e.g., particals' trajectories) with only the features (x1, x2,...,xd), rather than from the sampling data points (x1, x2, ...,xd, y) of the equations y=f(x1,x2,...xd). Specifically, it is easy to perform symbolic regressions to fit the equation E=D*log(u) from the data points

with the format of (u, E) pairs. However, symbolic regression-based methods (e.g., SciMED, gplean, and SINDy) can hardly discover this equation from the massive birds' flying trajectories, which do not explicitly contain u and E. In contrast, our Deflex present a novel framework to identify all distribution-based laws by learning the energy function from the observation data. To respond this point, we have revised the SI to present more details of the experiment settings, especially the provided features of all observation datasets. Also, we have added the configuration details of all compared methods in the SI.

References of response to this comment:

[1] Udrescu, S.-M., Tan, A., Feng, J., Neto, O., Wu, T., Tegmark, M.: Ai feynman 2.0: Pareto-optimal symbolic regression exploiting graph modularity. Advances in Neural Information Processing Systems 33, 4860–4871 (2020)

Revised SI for Comment 1.1:

1.4.2 Configurations of Compared Methods

Beyond the above general setups for comparison, we adhered to the algorithmic configurations and hyperparameter settings recommended in the respective official documentations, which normally reflect standard usage scenarios. The specific implementation details for each method are delineated below.

Operon: We deployed the Operon framework, a high-performance C++ library for symbolic regression. The algorithm was configured to utilize its analytical gradient-based optimization capabilities to fine-tune scalar coefficients within the expression trees. We set the population size to $1,000$ individuals and allowed the evolutionary process to run for up to $1,000$ generations, subject to the global time limit. Selection was performed using a tournament size of 5, and we employed the standard suite of genetic operators including subtree crossover and mutation with the library's default probability distributions.

SciMED: The SciMED (Scientist-Machine Equation Discovery) method was implemented utilizing its physics-informed Monte Carlo Tree Search (MCTS) architecture. We maintained the default exploration-exploitation balance governed by the Upper Confidence Bound (UCB) exploration constant $c_p = 1.0$. The search process was constrained to expression trees of maximum depth comparable to other tree-based methods to ensure a fair exploration of the functional space. The method's unique capability to leverage physical unit constraints was enabled where variable dimensions were applicable.

SINDy: Sparse Identification of Nonlinear Dynamics (SINDy) was executed using the STLSQ (Sequentially Thresholded Least Squares) optimizer. To balance sparsity and accuracy, we set the threshold parameter to 0.1 and allowed for a maximum of 20 iterations for the sparse regression coefficients to converge. The library of candidate functions was constructed to include polynomial terms up to degree 3 as well as trigonometric functions (sine and cosine), providing a comprehensive basis for discovering governing dynamical equations.

gplearn: We utilized the `SymbolicRegressor` module from the `gplearn` library, which implements a canonical genetic programming algorithm. The evolution was initialized with a population of $1,000$ programs and evolved over 20 generations. To mitigate the phenomenon of code bloat, a parsimony coefficient of 0.001 was applied to the fitness function, penalizing overly complex expressions. The genetic operations were configured with standard

probabilities: crossover (0.9), subtree mutation (0.01), hoist mutation (0.05), and point mutation (0.01).

AI Feynman 2.0: This physics-inspired framework was deployed with its full recursive strategy enabled. The method integrates neural network fitting to approximate the underlying function, followed by a battery of structural tests including dimensional analysis, symmetry detection, and separability checks. We utilized the default settings for the internal neural network training (epochs and architecture) to act as a reliable proxy for the data. The brute-force search component, invoked for identifying low-dimensional sub-problems, was restricted by the global timeout to prevent excessive computational overhead on high-dimensional tasks.

PySR: We employed the `PySR` package, which leverages a high-performance backend for regularized evolution. The search was distributed across multiple populations with a size of 33 individuals each, running for 100 iterations per restart cycle. The complexity-accuracy trade-off was managed via the default parsimony settings. We utilized the standard set of unary and binary operators (addition, subtraction, multiplication, division, sine, cosine, and exponential) to define the search space, allowing the algorithm to optimize both the structure and constants of the candidate equations.

DEAP: A standard Genetic Programming (GP) pipeline was constructed using the `DEAP` evolutionary computation framework. This baseline implementation followed the classic Koza-style GP paradigm. The evolutionary parameters were set to a population size of 500 evolving over 50 generations. Tree initialization was performed using the ramped half-and-half method with a depth limit of 17 to prevent memory overflow. Genetic operators included one-point crossover and uniform mutation, configured with probabilities of 0.5 and 0.1 respectively, to drive the search through the program space.

1.5 Successfully Discovered Formulas of All Methods

Supplementary Table 8 presents the full mathematical expressions of the laws discovered by Deflex and the baseline methods. These formulas represent the original outputs obtained from the symbolic regression process, corresponding to the experimental results discussed in the main text. Most Deflex formulas are expressed in the energy form (E), while the baseline methods' formulas are either equations (for deterministic laws, e.g., momentum conservation, continuity,

Newton viscosity law) or probability distributions (P, e.g., Maxwell-Boltzmann distribution, Lévy flight).

Supplementary Table 8: Full forms of discovered formulas by all compared methods

Law Name	Methods	Formula Form
Energy conservation	Deflex	`E = 0.992 * mass * (vel_x^2 + vel_y^2 + vel_z^2) / (k_B * temperature)`
	Operon	`E = mass * (vel_x^2 + vel_y^2 + vel_z^2) / (k_B * temperature)`
	SciMED	`E = mass * (vel_x^2 + vel_y^2 + vel_z^2) / (k_B * temperature)`
	SINDy	`E = mass * (vel_x^2 + vel_y^2 + vel_z^2) / (k_B * temperature)`
	gplearn	`E = mass * (vel_x^2 + vel_y^2 + vel_z^2) / (k_B * temperature)`
	AI Feynman	`E = mass * (vel_x^2 + vel_y^2 + vel_z^2) / (k_B * temperature)`
	PySR	`E = mass * (vel_x^2 + vel_y^2 + vel_z^2) / (k_B * temperature)`
	DEAP	`E = mass * (vel_x^2 + vel_y^2 + vel_z^2) / (k_B * temperature)`
Momentum conservation	Deflex	`E = (sum(mass * vel_x) - C_x)^2 + (sum(mass * vel_y) - C_y)^2 + (sum(mass * vel_z) - C_z)^2`
	Operon	`sum(mass(i) * vel_x(i)) = C_x,`<br>`sum(mass(i) * vel_y(i)) = C_y,`<br>`sum(mass(i) * vel_z(i)) = C_z`
	SciMED	`sum(mass(i) * vel_x(i)) = C_x,`<br>`sum(mass(i) * vel_y(i)) = C_y,`<br>`sum(mass(i) * vel_z(i)) = C_z`
	SINDy	`sum(mass(i) * vel_x(i)) = C_x,`<br>`sum(mass(i) * vel_y(i)) = C_y,`<br>`sum(mass(i) * vel_z(i)) = C_z`
	gplearn	`sum(mass(i) * vel_x(i)) = C_x,`<br>`sum(mass(i) * vel_y(i)) = C_y,`<br>`sum(mass(i) * vel_z(i)) = C_z`
	AI Feynman	`sum(mass(i) * vel_x(i)) = C_x,`<br>`sum(mass(i) * vel_y(i)) = C_y,`<br>`sum(mass(i) * vel_z(i)) = C_z`
	PySR	`sum(mass(i) * vel_x(i)) = C_x,`<br>`sum(mass(i) * vel_y(i)) = C_y,`<br>`sum(mass(i) * vel_z(i)) = C_z`
	DEAP	`sum(mass(i) * vel_x(i)) = C_x,`<br>`sum(mass(i) * vel_y(i)) = C_y,`<br>`sum(mass(i) * vel_z(i)) = C_z`
Maxwell-Boltzmann (MBD)	Deflex	`E = 0.511 * mass * (vel_x^2 + vel_y^2 + vel_z^2) / (k_B * temperature)`
	Operon	`P ~ exp( -mass * (vel_x^2 + vel_y^2 + vel_z^2) / (2 * k_B * temperature) )`
	SciMED	`P ~ exp( -mass * (vel_x^2 + vel_y^2 + vel_z^2) / (2 * k_B * temperature) )`
	gplearn	`P ~ exp( -mass * (vel_x^2 + vel_y^2 + vel_z^2) / (2 * k_B * temperature) )`
	AI Feynman	`P ~ exp( -mass * (vel_x^2 + vel_y^2 + vel_z^2) / (2 * k_B * temperature) )`
	PySR	`P ~ exp( -mass * (vel_x^2 + vel_y^2 + vel_z^2) / (2 * k_B * temperature) )`
	DEAP	`P ~ exp( -mass * (vel_x^2 + vel_y^2 + vel_z^2) / (2 * k_B * temperature) )`
MBD with potential	Deflex	`E = 0.506 * mass * (vel_x^2 + vel_y^2 + vel_z^2) / (k_B * temperature)`<br>`+ sum(0.97 * ((pos_x(i)-pos_x(j))^2+(pos_y(i)-pos_`<br>`- 1.89 * ((pos_x(i)-pos_x(j))^2+(pos_y(i)-pos_y(j)`
Continuity of quality	Deflex	`E = (vel_x_dx + vel_y_dy)^2 * 10^26`
	Operon	`vel_x_dx + vel_y_dy = 0`
	SciMED	`vel_x_dx + vel_y_dy = 0`
	SINDy	`vel_x_dx + vel_y_dy = 0`
	gplearn	`vel_x_dx + vel_y_dy = 0`
	AI Feynman	`vel_x_dx + vel_y_dy = 0`
	PySR	`vel_x_dx + vel_y_dy = 0`
	DEAP	`vel_x_dx + vel_y_dy = 0`

Law Name	Methods	Formula Form
Newton viscosity law	Deflex	τ_xy = -1.037 * μ * (vel_x_dy + vel_y_dx)
	Operon	τ_xy = −μ * (vel_x_dy + vel_y_dx)
	SciMED	τ_xy = −μ * (vel_x_dy + vel_y_dx)
	SINDy	τ_xy = −μ * (vel_x_dy + vel_y_dx)
	gplearn	τ_xy = −μ * (vel_x_dy + vel_y_dx)
	AI Feynman	τ_xy = −μ * (vel_x_dy + vel_y_dx)
	PySR	τ_xy = −μ * (vel_x_dy + vel_y_dx)
	DEAP	τ_xy = −μ * (vel_x_dy + vel_y_dx)
Velocity distribution	Deflex	E = 0.501 * mass * (vel_x^2 + vel_y^2) + (δv)^3.15
	AI Feynman	P ~ exp(-(0.5 * mass * (vel_x^2 + vel_y^2) + (δv)^p))
Navier-Stokes equation (2D Cylinder)	Deflex	E = (ρ * (vel_x_dt + vel_x * vel_x_dx + vel_y * vel_x_dy) + pressure_dx − μ * (vel_x_dx_dx + vel_x_dy_dy))^2 * 1.62e21 + (ρ * (vel_y_dt + vel_x * vel_y_dx + vel_y * vel_y_dy) + pressure_dy − μ * (vel_y_dx_dx + vel_y_dy_dy))^2 * 1.62e21
	Operon	ρ * (vel_x_dt + vel_x * vel_x_dx + vel_y * vel_x_dy) + pressure_dx − μ * (vel_x_dx_dx + vel_x_dy_dy) = 0, ρ * (vel_y_dt + vel_x * vel_y_dx + vel_y * vel_y_dy) + pressure_dy − μ * (vel_y_dx_dx + vel_y_dy_dy) = 0

Law Name	Methods	Formula Form
Navier-Stokes equation (3D JHTDB)	Deflex	E = (ρ * (vel_x_dt + vel_x * vel_x_dx + vel_y * vel_x_dy + vel_z * vel_x_dz) + pressure_dx − μ * (vel_x_dx_dx + vel_x_dy_dy + vel_x_dz_dz))^2 * 3.27e21 + (ρ * (vel_y_dt + vel_x * vel_y_dx + vel_y * vel_y_dy + vel_z * vel_y_dz) + pressure_dy − μ * (vel_y_dx_dx + vel_y_dy_dy + vel_y_dz_dz))^2 * 3.27e21 + (ρ * (vel_z_dt + vel_x * vel_z_dx + vel_y * vel_z_dy + vel_z * vel_z_dz) + pressure_dz − μ * (vel_z_dx_dx + vel_z_dy_dy + vel_z_dz_dz))^2 * 3.27e21
Langevin	Deflex	E = (4.438e16 * mass * vel_x_dt + 1.003 * vel_x)^2 + (4.438e16 * mass * vel_y_dt + 1.003 * vel_y)^2 + (4.438e16 * mass * vel_z_dt + 1.003 * vel_z)^2
	Operon	4.468e16 * mass * vel_x_dt + vel_x = 0, 4.468e16 * mass * vel_y_dt + vel_y = 0, 4.468e16 * mass * vel_z_dt + vel_z = 0
Levy flight (Human)	Deflex	E = 2.37 * log(sqrt((lat(time) - lat(time-1))^2 + (lon(time) - lon(time-1))^2))
	AI Feynman	P ~ u^-2.37
	PySR	P ~ u^-2.37
	DEAP	P ~ u^-2.37
Levy flight (Avian)	Deflex	E = 1.627 * log(sqrt((lat(time) - lat(time-1))^2 + (lon(time) - lon(time-1))^2))
	AI Feynman	P ~ u^-1.627
	PySR	P ~ u^-1.627
	DEAP	P ~ u^-1.627
Power law	Deflex	E = 1.56 * log(l) + 0.00027 * l
	AI Feynman	P ~ l^-1.56 * exp(-0.00027 * l)

Law Name	Methods	Formula Form
Navier-Stokes equation (3D JHTDB)	Deflex	E = (ρ * (vel_x_dt + vel_x * vel_x_dx + vel_y * vel_x_dy + vel_z * vel_x_dz) + pressure_dx − μ * (vel_x_dx_dx + vel_x_dy_dy + vel_x_dz_dz))^2 * 3.27e21 + (ρ * (vel_y_dt + vel_x * vel_y_dx + vel_y * vel_y_dy + vel_z * vel_y_dz) + pressure_dy − μ * (vel_y_dx_dx + vel_y_dy_dy + vel_y_dz_dz))^2 * 3.27e21 + (ρ * (vel_z_dt + vel_x * vel_z_dx + vel_y * vel_z_dy + vel_z * vel_z_dz) + pressure_dz − μ * (vel_z_dx_dx + vel_z_dy_dy + vel_z_dz_dz))^2 * 3.27e21
Langevin	Deflex	E = (4.438e16 * mass * vel_x_dt + 1.003 * vel_x)^2 + (4.438e16 * mass * vel_y_dt + 1.003 * vel_y)^2 + (4.438e16 * mass * vel_z_dt + 1.003 * vel_z)^2
	Operon	4.468e16 * mass * vel_x_dt + vel_x = 0, 4.468e16 * mass * vel_y_dt + vel_y = 0, 4.468e16 * mass * vel_z_dt + vel_z = 0
Levy flight (Human)	Deflex	E = 2.37 * log(sqrt((lat(time) - lat(time-1))^2 + (lon(time) - lon(time-1))^2))
	AI Feynman	P ~ u^-2.37
	PySR	P ~ u^-2.37
	DEAP	P ~ u^-2.37
Levy flight (Avian)	Deflex	E = 1.627 * log(sqrt((lat(time) - lat(time-1))^2 + (lon(time) - lon(time-1))^2))
	AI Feynman	P ~ u^-1.627
	PySR	P ~ u^-1.627
	DEAP	P ~ u^-1.627
Power law	Deflex	E = 1.56 * log(l) + 0.00027 * l
	AI Feynman	P ~ l^-1.56 * exp(-0.00027 * l)

Law Name	Methods	Formula Form
Crowd power law	Deflex	E = 1.63 * log(r(i)) - 0.037 * log(r(i)) * sum(I(sqrt((lat(j)-lat(i))^2 + (lon(j)-lon(i))^2)<2.46))

Supplementary Table 8 lists the full, raw mathematical expressions discovered by Deflex and baseline methods. These expressions use the specific variable names defined in the feature tables (Supplementary Tables 1–5) and correspond to the standard mathematical notations presented in Table 1 of the main text. Note that most Deflex formulas are expressed in the energy form (E) to unify different types of physical laws, while baseline methods output either equations (for deterministic laws) or probability distributions (P). For the fluid dynamics experiments, we report results from both the 2D cylinder wake flow and the 3D JHTDB turbulence dataset, demonstrating Deflex's ability to discover the Navier-Stokes equation across different spatial dimensions and flow regimes. For clarity, the mapping between the symbols in the main text and the variable names in the Supplementary Information is as follows: The velocity vectors v (or u in fluid dynamics) correspond to the component features vel_x, vel_y, vel_z. The position vectors $\vec{x}$ correspond to pos_x, pos_y, pos_z (or lat, lon in mobility tasks). Particle or individual indices i, j are represented using parentheses, e.g., pos_x(i), mass(i), lat(j). Time indices are similarly denoted by lat(time) and lat(time-1) for current and previous time steps. The displacement scalar Δx and interaction distances $\|\vec{x}_j - \vec{x}_i\|$ in the main text are explicitly expanded here as Euclidean distances, e.g., sqrt((lat(time) - lat(time-1))^2 + (lon(time) - lon(time-1))^2) or sqrt((pos_x(i)-pos_x(j))^2 + ...). Differential operators such as ∇ and $\partial/\partial t$ are represented by suffix notation, where _dx and _dt denote partial derivatives with respect to x and t, respectively (e.g., $\frac{\partial u}{\partial x}$ becomes vel_x_dx). Global parameters like mass m, temperature T, and pressure p directly map to

`mass`, `temperature`, and `pressure`. Greek letters such as μ (viscosity), ρ (density), τ (stress), and δ (variation) are retained in mathematical notation.

Comment 1.2:

Very strange for me - for the "Newton viscosity law", if du/dx is not implicitly provided, all the methods should be N.A. and not V as they do not support it. If du/dx is provided explicitly, this is not a fair comparison. To this end, Velocity distribution for Deflex replaces "p" with "3.15" - sorry, this cannot considered a "V". The SR misses a term means it does not recreate the physics as it should do. Same for "Power law" and "Levy flight".

Response to Comment 1.2:

We thank the reviewer for this comment and would like to clarify that this is a fair comparison, where the gradients of field *du/dt* were provided to all compared methods as input features in the observation data. Regarding the point "*... Velocity distribution for Deflex replaces "p" with "3.15"...*", the symbol p represents an unprovided constant for a given system but vary for different settings, rather than a feature-related parameter. Please also note, we have claimed in Subsection 2.2 of both original and revised manuscripts that *"the formulas of invariants are considered successful recovery if they can be expressed as the correct formula with an additional stochastic residual term"*. According to this principle, we believe velocity distribution is also a successful discovery.

To make the presentation more rigor, we have made the following modifications in this revision. We have revised Table 1 to highlight all the unprovided constants in the formulas. We have also revised SI to provide the complete, unabbreviated forms of the discovered equations, and presented more details of the experimental setups, especially the provided features of all observation datasets.

Revised Manuscript for Comment 1.2:

Table 1 Discovered expressions by Deflex in comparison with the ground truths

Rules		Ground truths and discovered by Deflex
Energy conservation	True	$E = v^2 \cdot m/k_BT$
	Discovered	$E = 0.992v^2 \cdot m/k_BT$
Momentum conservation	True	$\sum m_i\mathbf{v}_i = C$
	Discovered	$\sum m_i\mathbf{v}_i = C$
Maxwell-Boltzmann distribution (MBD)	True	$E = \frac{mv^2}{2k_BT}$
	Discovered	$E = \frac{0.511mv^2}{k_BT}$
MBD with potential	True	$E = \frac{mv^2}{2k_BT} + \sum_{j\neq i}\left(\lVert\vec{x_j} - \vec{x_i}\rVert^{-12} - 2\lVert\vec{x_j} - \vec{x_i}\rVert^{-6}\right)$
	Discovered	$E = \frac{0.506mv^2}{k_BT} + \sum_{j\neq i}\left(0.97\lVert\vec{x_j} - \vec{x_i}\rVert^{-12} - 1.89\lVert\vec{x_j} - \vec{x_i}\rVert^{-6}\right)$
Continuity of quality	True	$E = \lVert\nabla \cdot v\rVert \times \infty$
	Discovered	$E = \lVert\nabla \cdot v\rVert \times 10^{26}$
Newton viscosity law	True	$\tau_{ij} = -\mu\left(\frac{\partial u_i}{\partial x_j} + \frac{\partial u_j}{\partial x_i}\right)$
	Discovered	$\tau_{ij} = -1.037\mu\left(\frac{\partial u_i}{\partial x_j} + \frac{\partial u_j}{\partial x_i}\right)$
Velocity distribution	True	$E = \frac{1}{2}mv^2 + (\delta v)^P$
	Discovered	$E = 0.501mv^2 + (\delta v)^{3.15}$
Navier-Stokes equation	True	$E = \lVert\rho\left(\frac{\partial\mathbf{u}}{\partial t} + \mathbf{u}\cdot\nabla\mathbf{u}\right) + \nabla p - \mu\nabla^2\mathbf{u}\rVert \times \infty$
	Discovered	$E = \lVert\rho\left(\frac{\partial\mathbf{u}}{\partial t} + \mathbf{u}\cdot\nabla\mathbf{u}\right) + \nabla p - \mu\nabla^2\mathbf{u}\rVert \times 1.62 \times 10^{21}$
Langevin	True	$E = \lVert 4.468 \times 10^{16}\ \mathrm{m}\dot{v} + v\rVert^2$
	Discovered	$E = \lVert 4.438 \times 10^{16} m\dot{v} + 1.003v\rVert^2$
Levy flight (Human)	True	$E = D_1 \log \Delta x$
	Discovered	$E = 2.37 \log \Delta x$
Levy flight (Avian)	True	$E = D_2 \log \Delta x$
	Discovered	$E = 1.627 \log \Delta x$
Power law	True	$E = \alpha \log l + \gamma l$
	Discovered	$E = 1.56 \log l + 0.00027l$
Crowd power law	True	Undocumented
	Discovered	$E = 1.63 \log r_i - 0.037 \log r_i \sum_{j\neq i} \mathbb{I}\left(\left\lVert x_t^{(j)} - x_t^{(i)}\right\rVert < 2.46\right)$

k_B, δ, ρ, P, D_1, D_2, α, and γ are unprovided constants in the experiments. All other symbols denote the provided features of observation data in the experiments. Detailed explanations of these features are described in SI.

Revised SI for Comment 1.2:

Supplementary Table 2: Features for Symbolic Regression in Argon Particle Experiment

Type	Feature	Description
Element-wise	pos_x, pos_y, pos_z<br>vel_x, vel_y, vel_z<br>time	Coordinates of the particle in the 3D field.<br>Velocity components of the particle.<br>Simulation timestamp.
Global	mass<br>temperature	Mass of the particle (constant for all particles).<br>System temperature (varies across three states: 149.72K, 150.72K, 151.72K).
Scale	N, T_{obs}	100 particles $\times$ 100,000 frames across three temperature points.

Supplementary Table 3: Features of Observation Data in Pollen Particle Experiment

Type	Feature	Description
Element-wise	pos_x, pos_y, pos_z<br>vel_x, vel_y, vel_z<br>radius<br>density<br>time	3D coordinates of the pollen particle in the tube.<br>Velocity components.<br>Radius of the pollen particle (30–50 μm).<br>Particle density (1.5$\times$ water).<br>Simulation timestamp.
Global	temperature	System temperature (300K).
Scale	N, T_{obs}	400 particles $\times$ 10,000 frames.

Supplementary Table 4: Features for Symbolic Regression in Human Mobility Experiment

Type	Feature	Description
Element-wise	lat, lon<br>time	Latitude and longitude from GPS data.<br>Timestamp of the record.
Scale	N, T_{obs}	1,000 users $\times$ Daily trajectories (Carnival).

Supplementary Table 5: Features of Observation Data in Bird Migration Experiment

Type	Feature	Description
Element-wise	lat, lon<br>time	Global latitude and longitude.<br>Temporal information of the observation.
Scale	N, T_{obs}	100,000 birds $\times$ 1,000 days.

Comment 1.3:

Moreover, the authors must indicate how many samples there are for each equation, how they sampled, and what features were provided to each model - otherwise, it is impossible to understand the experiments and the results.

Response to Comment 1.3:

The authors thank the reviewer very much for this suggestion. We agree that it is essential to provide the complete details of the provided features for each equation, their exact discovered forms, and the specifics of data sampling.

To respond this comment, in this revision, we have added the details of the datasets, including the sampling methods, provided features, their descriptions, and sizes for each experiment in Supplementary Tables 2-5 in the revised SI. Besides, we have also provided the detailed configurations for all compared methods in the revised SI. Also, we have updated the experiment setup for each experiment in Sec. 2 of the revised manuscript.

Revised Manuscript for Comment 1.3:

2.3 Particle Motion

The study of the motion of particles, like pollen, molecules, and fundamental particles, has contributed to some breakthrough discoveries, such as the law of energy conservation, the Maxwell distribution, the Boltzmann distribution, and the Langevin equation. Many of these have helped reveal fundamental understandings of how patterns emerge in systems across scales [2, 37]. For instance, the Langevin equation manifests that small-scale dynamics can be approximated as noise at larger scales.

We first applied Deflex to the particle motion systems of rare gases and pollen in water [37]. In the rare gas particle motion experiment, we simulated the motion of argon particles at three temperature points near the critical point (T_c, $T_c \pm 1$K, where $T_c = 150.72$K) within a cube, which allows us to capture the system's behavior across different thermodynamic states. When the particles approached the cube edge, they experienced a repulsive force analogous to intermolecular forces. See SI 2.1 for a detailed experimental setup. In the pollen experiment in water, we simulated water and pollen within a microtube. This represents a typical two-scale system, involving microscopic water molecules and mesoscopic pollen. We sampled 16 to 2048 particles for 50 time frames as a data item and trained our network with a dataset of 65,536 sampled data items. For each particle at a time frame, the mass, position, and velocity are embedded into the embedding layer of 64 dimensions.

As shown in Table 1, Deflex successfully identifies a series of formulas, including the energy conservation, Boltzmann distribution, Maxwell-Boltzmann distribution (MBD), MBD with Lennard-Jones potential, and the Langevin equation, with only one pass training of Deflexformer in each of these particle motion systems. Notably, since temperature is provided as an input variable in the argon system, the discovered formulas naturally capture the temperature-dependent behaviors across different thermodynamic states, with temperature T appearing explicitly in the energy expressions. In particular, MBD with Lennard-Jones potential and the Langevin equation cannot be discovered by other compared methods in our experiments (shown in Table 2). And those tree-based symbolic regression method requires manual specification MBD with Lennard-Jones potential identified by Deflex in the dynamics of rare gas particles is a nonlinear equation that consists of up to 23 nodes, demonstrating Deflex's ability to discover complex nonlinear equations.

On the commonly identified Maxwell-Boltzmann distribution, Deflex manages to achieve the least NLL loss than the compared methods in both Argon and Pollen particles (shown in Fig. 3a). Meanwhile, the RMSEs of all compared methods are nearly the same on both energy conservation and momentum conservation in the two systems. This is because the structures of correctly identified formulas are almost identical in these methods, as mentioned before. As shown in Fig. 3b, Deflex incurs significantly less computational time than most of the comparatives. SINDy and Operon are quite close to or sometimes faster than Deflex, but only limited to some lower-order formulas like momentum conservation. This is due to their limitations in formula structures. Furthermore, ablation results indicate that our Langevin sampling strategy is essential for efficiently identifying these complex equilibrium distributions (Fig. 5g).

2.6 Fluid Dynamics

We conducted experiments on fluids (Fig. 3a, b), one of the most typical complex systems in physics [43]. Specifically, we focused on the wake behind a cylinder in a two-dimensional flow. The specific experimental setup is detailed in SI Document S2.2. We built a 1024×1024 mesh for simulation, and each one of 1M grids was regarded as an element, containing the position, pressure, and velocity of the grid as the data fields, and 2048 time frames were collected in total. We collected 9 to 256 adjacent or random grids and 128 time frames as a data snippet for training.

To test the generalizability of our approach across different flow regimes and spatial dimensions, we conducted a second fluid dynamics experiment using the Johns Hopkins Turbulence Database (JHTDB) [44]. Unlike the confined cylinder wake flow, the JHTDB provides Direct Numerical Simulation of three-dimensional forced isotropic turbulence, representing a canonical case of fully developed turbulence without boundary effects. The dataset features a 1024^3 computational grid with a Taylor microscale Reynolds number $Re_\lambda \approx 433$. We extracted volumetric subsamples from the database, with each grid point containing the three-dimensional velocity field (u, v, w), pressure p, and their spatial derivatives. Following the same training protocol as the cylinder experiment, we sampled 9 to 256 spatial locations and 128 temporal snapshots as input data. Remarkably, Deflex successfully identified the three-dimensional Navier-Stokes equation from this turbulent dataset (Table 1, Fig. 3), capturing the full three-dimensional convective and diffusive terms. The complementary nature of these two experiments—2D laminar wake versus 3D turbulent flow—demonstrates Deflex's capability to discover governing equations across different flow regimes, spatial dimensions, and Reynolds numbers.

As listed in Table 1, Deflex detects the Navier-Stokes equation of fluid dynamics through small-scale observations, which cannot be discovered by other methods. In larger-scale random observations, Deflex extracts the velocity distribution of the fluid within the vortex flow. These formulas correspond to different manifestations of long-standing laws in physics. For complex distribution formulas like velocity distribution, most compared methods are infeasible (shown in Table 2), and only AI Feynman and Deflex manage to discover.

Revised SI for Comment 1.3:

Supplementary Table 2: Features for Symbolic Regression in Argon Particle Experiment

Type	Feature	Description
Element-wise	pos_x, pos_y, pos_z<br>vel_x, vel_y, vel_z<br>time	Coordinates of the particle in the 3D field.<br>Velocity components of the particle.<br>Simulation timestamp.
Global	mass<br>temperature	Mass of the particle (constant for all particles).<br>System temperature (varies across three states: 149.72K, 150.72K, 151.72K).
Scale	N, T_{obs}	100 particles $\times$ 100,000 frames across three temperature points.

Supplementary Table 3: Features of Observation Data in Pollen Particle Experiment

Type	Feature	Description
Element-wise	pos_x, pos_y, pos_z<br>vel_x, vel_y, vel_z<br>radius<br>density<br>time	3D coordinates of the pollen particle in the tube.<br>Velocity components.<br>Radius of the pollen particle (30–50 μm).<br>Particle density (1.5$\times$ water).<br>Simulation timestamp.
Global	temperature	System temperature (300K).
Scale	N, T_{obs}	400 particles $\times$ 10,000 frames.

Supplementary Table 4: Features for Symbolic Regression in Human Mobility Experiment

Type	Feature	Description
Element-wise	lat, lon<br>time	Latitude and longitude from GPS data.<br>Timestamp of the record.
Scale	N, T_{obs}	1,000 users $\times$ Daily trajectories (Carnival).

Supplementary Table 5: Features of Observation Data in Bird Migration Experiment

Type	Feature	Description
Element-wise	lat, lon<br>time	Global latitude and longitude.<br>Temporal information of the observation.
Scale	N, T_{obs}	100,000 birds $\times$ 1,000 days.

1.4.2 Configurations of Compared Methods

Beyond the above general setups for comparison, we adhered to the algorithmic configurations and hyperparameter settings recommended in the respective official documentations, which normally reflect standard usage scenarios. The specific implementation details for each method are delineated below.

Operon: We deployed the Operon framework, a high-performance C++ library for symbolic regression. The algorithm was configured to utilize its analytical gradient-based optimization capabilities to fine-tune scalar coefficients within the expression trees. We set the population size to 1,000 individuals and allowed the evolutionary process to run for up to 1,000 generations, subject to the global time limit. Selection was performed using a tournament size of 5, and

we employed the standard suite of genetic operators including subtree crossover and mutation with the library's default probability distributions.

SciMED: The SciMED (Scientist-Machine Equation Discovery) method was implemented utilizing its physics-informed Monte Carlo Tree Search (MCTS) architecture. We maintained the default exploration-exploitation balance governed by the Upper Confidence Bound (UCB) exploration constant $c_p = 1.0$. The search process was constrained to expression trees of maximum depth comparable to other tree-based methods to ensure a fair exploration of the functional space. The method's unique capability to leverage physical unit constraints was enabled where variable dimensions were applicable.

SINDy: Sparse Identification of Nonlinear Dynamics (SINDy) was executed using the STLSQ (Sequentially Thresholded Least Squares) optimizer. To balance sparsity and accuracy, we set the threshold parameter to 0.1 and allowed for a maximum of 20 iterations for the sparse regression coefficients to converge. The library of candidate functions was constructed to include polynomial terms up to degree 3 as well as trigonometric functions (sine and cosine), providing a comprehensive basis for discovering governing dynamical equations.

gplearn: We utilized the `SymbolicRegressor` module from the `gplearn` library, which implements a canonical genetic programming algorithm. The evolution was initialized with a population of $1,000$ programs and evolved over 20 generations. To mitigate the phenomenon of code bloat, a parsimony coefficient of 0.001 was applied to the fitness function, penalizing overly complex expressions. The genetic operations were configured with standard probabilities: crossover (0.9), subtree mutation (0.01), hoist mutation (0.05), and point mutation (0.01).

AI Feynman 2.0: This physics-inspired framework was deployed with its full recursive strategy enabled. The method integrates neural network fitting to approximate the underlying function, followed by a battery of structural tests including dimensional analysis, symmetry de-

tection, and separability checks. We utilized the default settings for the internal neural network training (epochs and architecture) to act as a reliable proxy for the data. The brute-force search component, invoked for identifying low-dimensional sub-problems, was restricted by the global timeout to prevent excessive computational overhead on high-dimensional tasks.

PySR: We employed the `PySR` package, which leverages a high-performance backend for regularized evolution. The search was distributed across multiple populations with a size of 33 individuals each, running for 100 iterations per restart cycle. The complexity-accuracy trade-off was managed via the default parsimony settings. We utilized the standard set of unary and binary operators (addition, subtraction, multiplication, division, sine, cosine, and exponential) to define the search space, allowing the algorithm to optimize both the structure and constants of the candidate equations.

DEAP: A standard Genetic Programming (GP) pipeline was constructed using the `DEAP` evolutionary computation framework. This baseline implementation followed the classic Koza-style GP paradigm. The evolutionary parameters were set to a population size of 500 evolving over 50 generations. Tree initialization was performed using the ramped half-and-half method with a depth limit of 17 to prevent memory overflow. Genetic operators included one-point crossover and uniform mutation, configured with probabilities of 0.5 and 0.1 respectively, to drive the search through the program space.

1.4.3 Coarse-graining and Renormalization Protocols

To rigorously evaluate the capability of Deflex in discovering multiscale laws, we introduce a renormalization analysis framework. This involves applying specific coarse-graining operators $\mathcal{R}_\lambda$ with a scale factor λ to the system, and examining whether the method can independently identify the renormalized physical laws at each scale. We adopt two distinct coarse-graining protocols tailored to the nature of the simulated systems: re-simulation for particle systems and

Comment 1.4:

By the way, why not provide the equation for all models? It is an important comparison that the readers should be aware of...

Response to Comment 1.4:

We thank the reviewer for this comment. We have followed the suggestion to provide the successfully discovered formulas of all compared methods in the revised SI. Please note that, a successful discovery is defined in Subsection 2.2 of both original and revised manuscripts as that the discovered formula has the correct formula structure of the ground truth while allowing an additional random residual term, which is commonly adopted by other existing studies [1].

References to this comment:

[1] Udrescu, S.-M., Tan, A., Feng, J., Neto, O., Wu, T., Tegmark, M.: Ai feynman 2.0: Pareto-optimal symbolic regression exploiting graph modularity. Advances in Neural Information Processing Systems 33, 4860–4871 (2020)

Revised SI for Comment 1.4:

1.5 Successfully Discovered Formulas of All Methods

Supplementary Table 8 presents the full mathematical expressions of the laws discovered by Deflex and the baseline methods. These formulas represent the original outputs obtained from the symbolic regression process, corresponding to the experimental results discussed in the main text. Most Deflex formulas are expressed in the energy form (E), while the baseline methods' formulas are either equations (for deterministic laws, e.g., momentum conservation, continuity, Newton viscosity law) or probability distributions (P, e.g., Maxwell-Boltzmann distribution, Lévy flight).

Supplementary Table 8: Full forms of discovered formulas by all compared methods

Law Name	Methods	Formula Form
Energy conservation	Deflex	`E = 0.992 * mass * (vel_x^2 + vel_y^2 + vel_z^2) / (k_B * temperature)`
	Operon	`E = mass * (vel_x^2 + vel_y^2 + vel_z^2) / (k_B * temperature)`
	SciMED	`E = mass * (vel_x^2 + vel_y^2 + vel_z^2) / (k_B * temperature)`
	SINDy	`E = mass * (vel_x^2 + vel_y^2 + vel_z^2) / (k_B * temperature)`
	gplearn	`E = mass * (vel_x^2 + vel_y^2 + vel_z^2) / (k_B * temperature)`
	AI Feynman	`E = mass * (vel_x^2 + vel_y^2 + vel_z^2) / (k_B * temperature)`
	PySR	`E = mass * (vel_x^2 + vel_y^2 + vel_z^2) / (k_B * temperature)`
	DEAP	`E = mass * (vel_x^2 + vel_y^2 + vel_z^2) / (k_B * temperature)`
Momentum conservation	Deflex	`E = (sum(mass * vel_x) - C_x)^2 + (sum(mass * vel_y) - C_y)^2 + (sum(mass * vel_z) - C_z)^2`
	Operon	`sum(mass(i) * vel_x(i)) = C_x, sum(mass(i) * vel_y(i)) = C_y, sum(mass(i) * vel_z(i)) = C_z`
	SciMED	`sum(mass(i) * vel_x(i)) = C_x, sum(mass(i) * vel_y(i)) = C_y, sum(mass(i) * vel_z(i)) = C_z`
	SINDy	`sum(mass(i) * vel_x(i)) = C_x, sum(mass(i) * vel_y(i)) = C_y, sum(mass(i) * vel_z(i)) = C_z`
	gplearn	`sum(mass(i) * vel_x(i)) = C_x, sum(mass(i) * vel_y(i)) = C_y, sum(mass(i) * vel_z(i)) = C_z`

Law Name	Methods	Formula Form
	AI Feynman	sum(mass(i) * vel_x(i)) = C_x,<br>sum(mass(i) * vel_y(i)) = C_y,<br>sum(mass(i) * vel_z(i)) = C_z
	PySR	sum(mass(i) * vel_x(i)) = C_x,<br>sum(mass(i) * vel_y(i)) = C_y,<br>sum(mass(i) * vel_z(i)) = C_z
	DEAP	sum(mass(i) * vel_x(i)) = C_x,<br>sum(mass(i) * vel_y(i)) = C_y,<br>sum(mass(i) * vel_z(i)) = C_z
Maxwell-Boltzmann (MBD)	Deflex	E = 0.511 * mass * (vel_x^2 + vel_y^2 + vel_z^2) / (k_B * temperature)
	Operon	P ~ exp(-mass * (vel_x^2 + vel_y^2 + vel_z^2) / (2 * k_B * temperature))
	SciMED	P ~ exp(-mass * (vel_x^2 + vel_y^2 + vel_z^2) / (2 * k_B * temperature))
	gplearn	P ~ exp(-mass * (vel_x^2 + vel_y^2 + vel_z^2) / (2 * k_B * temperature))
	AI Feynman	P ~ exp(-mass * (vel_x^2 + vel_y^2 + vel_z^2) / (2 * k_B * temperature))
	PySR	P ~ exp(-mass * (vel_x^2 + vel_y^2 + vel_z^2) / (2 * k_B * temperature))
	DEAP	P ~ exp(-mass * (vel_x^2 + vel_y^2 + vel_z^2) / (2 * k_B * temperature))
MBD with potential	Deflex	E = 0.506 * mass * (vel_x^2 + vel_y^2 + vel_z^2) / (k_B * temperature)<br>+ sum(0.97 * ((pos_x(i)-pos_x(j))^2+(pos_y(i)-pos<br>- 1.89 * ((pos_x(i)-pos_x(j))^2+(pos_y(i)-pos_y(j)
Continuity of quality	Deflex	E = (vel_x_dx + vel_y_dy)^2 * 10^26
	Operon	vel_x_dx + vel_y_dy = 0
	SciMED	vel_x_dx + vel_y_dy = 0
	SINDy	vel_x_dx + vel_y_dy = 0
	gplearn	vel_x_dx + vel_y_dy = 0
	AI Feynman	vel_x_dx + vel_y_dy = 0
	PySR	vel_x_dx + vel_y_dy = 0
	DEAP	vel_x_dx + vel_y_dy = 0

Law Name	Methods	Formula Form
Newton viscosity law	Deflex	τ_xy = -1.037 * μ * (vel_x_dy + vel_y_dx)
	Operon	τ_xy = -μ * (vel_x_dy + vel_y_dx)
	SciMED	τ_xy = -μ * (vel_x_dy + vel_y_dx)
	SINDy	τ_xy = -μ * (vel_x_dy + vel_y_dx)
	gplearn	τ_xy = -μ * (vel_x_dy + vel_y_dx)
	AI Feynman	τ_xy = -μ * (vel_x_dy + vel_y_dx)
	PySR	τ_xy = -μ * (vel_x_dy + vel_y_dx)
	DEAP	τ_xy = -μ * (vel_x_dy + vel_y_dx)
Velocity distribution	Deflex	E = 0.501 * mass * (vel_x^2 + vel_y^2) + (δv)^3.15
	AI Feynman	P ~ exp(-(0.5 * mass * (vel_x^2 + vel_y^2) + (δv)^p))
Navier-Stokes equation (2D Cylinder)	Deflex	E = (ρ * (vel_x_dt + vel_x * vel_x_dx + vel_y * vel_x_dy) + pressure_dx - μ * (vel_x_dx_dx + vel_x_dy_dy))^2 * 1.62e21<br>+ (ρ * (vel_y_dt + vel_x * vel_y_dx + vel_y * vel_y_dy) + pressure_dy - μ * (vel_y_dx_dx + vel_y_dy_dy))^2 * 1.62e21
	Operon	ρ * (vel_x_dt + vel_x * vel_x_dx + vel_y * vel_x_dy) + pressure_dx - μ * (vel_x_dx_dx + vel_x_dy_dy) = 0,<br>ρ * (vel_y_dt + vel_x * vel_y_dx + vel_y * vel_y_dy) + pressure_dy - μ * (vel_y_dx_dx + vel_y_dy_dy) = 0

Law Name	Methods	Formula Form
Navier-Stokes equation (3D JHTDB)	Deflex	E = (ρ * (vel_x_dt + vel_x * vel_x_dx + vel_y * vel_x_dy + vel_z * vel_x_dz) + pressure_dx − μ * (vel_x_dx_dx + vel_x_dy_dy + vel_x_dz_dz))^2 * 3.27e21 + (ρ * (vel_y_dt + vel_x * vel_y_dx + vel_y * vel_y_dy + vel_z * vel_y_dz) + pressure_dy − μ * (vel_y_dx_dx + vel_y_dy_dy + vel_y_dz_dz))^2 * 3.27e21 + (ρ * (vel_z_dt + vel_x * vel_z_dx + vel_y * vel_z_dy + vel_z * vel_z_dz) + pressure_dz − μ * (vel_z_dx_dx + vel_z_dy_dy + vel_z_dz_dz))^2 * 3.27e21
Langevin	Deflex	E = (4.438e16 * mass * vel_x_dt + 1.003 * vel_x)^2 + (4.438e16 * mass * vel_y_dt + 1.003 * vel_y)^2 + (4.438e16 * mass * vel_z_dt + 1.003 * vel_z)^2
	Operon	4.468e16 * mass * vel_x_dt + vel_x = 0, 4.468e16 * mass * vel_y_dt + vel_y = 0, 4.468e16 * mass * vel_z_dt + vel_z = 0
Levy flight (Human)	Deflex	E = 2.37 * log(sqrt((lat(time) − lat(time−1))^2 + (lon(time) − lon(time−1))^2))
	AI Feynman	P ~ u^−2.37
	PySR	P ~ u^−2.37
	DEAP	P ~ u^−2.37
Levy flight (Avian)	Deflex	E = 1.627 * log(sqrt((lat(time) − lat(time−1))^2 + (lon(time) − lon(time−1))^2))
	AI Feynman	P ~ u^−1.627
	PySR	P ~ u^−1.627
	DEAP	P ~ u^−1.627
Power law	Deflex	E = 1.56 * log(l) + 0.00027 * l
	AI Feynman	P ~ l^−1.56 * exp(−0.00027 * l)

Law Name	Methods	Formula Form
Crowd power law	Deflex	E = 1.63 * log(r(i)) − 0.037 * log(r(i)) * sum(I(sqrt((lat(j)−lat(i))^2 + (lon(j)−lon(i))^2)<2.46))

Supplementary Table 8 lists the full, raw mathematical expressions discovered by Deflex and baseline methods. These expressions use the specific variable names defined in the feature tables (Supplementary Tables 1–5) and correspond to the standard mathematical notations presented in Table 1 of the main text. Note that most Deflex formulas are expressed in the energy form (E) to unify different types of physical laws, while baseline methods output either equations (for deterministic laws) or probability distributions (P). For the fluid dynamics experiments, we report results from both the 2D cylinder wake flow and the 3D JHTDB turbulence dataset, demonstrating Deflex's ability to discover the Navier-Stokes equation across different spatial dimensions and flow regimes. For clarity, the mapping between the symbols in the main text and the variable names in the Supplementary Information is as follows: The velocity vectors $\mathbf{v}$ (or $\mathbf{u}$ in fluid dynamics) correspond to the component features `vel_x`, `vel_y`, `vel_z`. The position vectors $\vec{x}$ correspond to `pos_x`, `pos_y`, `pos_z` (or `lat`, `lon` in mobility tasks). Particle or individual indices i, j are represented using parentheses, e.g., `pos_x(i)`, `mass(i)`, `lat(j)`. Time indices are similarly denoted by `lat(time)` and `lat(time-1)` for current and previous time steps. The displacement scalar Δx and interaction distances $\|\vec{x}_j - \vec{x}_i\|$ in the main text are explicitly expanded here as Euclidean distances, e.g., `sqrt((lat(time) - lat(time-1))^2 + (lon(time) - lon(time-1))^2)` or `sqrt((pos_x(i)-pos_x(j))^2 + ...)`. Differential operators such as ∇ and $\partial/\partial t$ are represented by suffix notation, where `_dx` and `_dt` denote partial derivatives with respect to x and t, respectively (e.g., $\frac{\partial u}{\partial x}$ becomes `vel_x_dx`). Global parameters like mass m, temperature T, and pressure p directly map to `mass`, `temperature`, and `pressure`. Greek letters such as μ (viscosity), ρ (density), τ (stress), and δ (variation) are retained in mathematical notation.

2.2 Evaluation Metrics

Since uncovering formulas within a complex system may represent an open-ended challenge, we incorporate qualitative assessments, basic quantitative analyses, renormalization and coarse-graining techniques [35] in the performance evaluation.

For established or empirical formulas, we first qualitatively assess whether the structure and parameters of the discovered formulas align with the ground truth. The formulas of invariants are considered successful recovery if they can be expressed as the correct formula with an additional stochastic residual term. Table 1 lists all the discovered formulas by Deflex in the studied six systems, compared to the ground truths. It should be noted that, for those undocumented discoveries, we provide justifications for their possible meanings in the real world. For a reference, we also summarize the capability of the compared methods for discovering these formulas in Table 2.

To quantitatively evaluate the accuracy of discovered formulas, we consider two cases based on the different forms of formulas separately.

- For the formulas in the form of equations and invariants, we employ the relative mean square error (RMSE) between the prediction and the true value. Specifically, all compared methods are given the same convergence threshold. Note that, in such a case, the RMSEs of different methods are hardly comparable since there is almost no structural difference (except for residual terms with negligible errors) in the commonly identified formulas in these forms.
- For distribution-based formulas, we use the negative log-likelihood (NLL) loss defined in Eq. (8) in the method section, which measures the likelihood of the distribution fitting real data. The feasible methods were also set with the same convergence constraint. Note that we also report the RMSE to measure the fitting error of simple distributions for reference.

Fig 4a compares the NLL loss of Deflex's distribution discovery in comparison with other methods, and reports the RMSE on the commonly identified equations and invariants.

To evaluate the efficiency of Deflex, we run Deflex and other comparative algorithms on a workstation with 32 CPU cores working in parallel, and two Tesla V100 GPUs for GPU-accelerated computing (details shown in SI Sec. 3). We compare the time efficiency of Deflex with that of other methods by recording the run-time of finding the same or equivalent formulas from the same dataset, within the same tolerable error in NLL or RMSE. A discovery is considered successful when its error remains within a small margin of the residual error, defined as the minimum achievable discrepancy when fitting the ground truth formula to observations [6]. This baseline accounts for irreducible noise from measurements and stochastic fluctuations in the system (details in SI). Fig 4b shows the comparison results.

In our experiments, we employ different observation scales by varying the spatiotemporal scope of data sampling. Small-scale observations use localized sampling (e.g., selecting spatiotemporally neighboring data points) to capture fine-grained interactions and facilitate the discovery of governing equations, while large-scale observations employ uniform random sampling across the entire domain to reveal macroscopic statistical patterns such as distributions (see SI for detailed sampling protocols).

=======Reviewer #3=======

Comment 3.1:

The authors did a very good job in improving the paper. Nevertheless, I can still not advise publishing it. On the one hand, intermediate results are still not documented and discussed, only described superficially. This does not explain how the method works to the reader.

Response to Comment 3.1:

We thank the reviewer for recognizing our improvements and sincerely appreciate the rigorous requirements for the details. To respond this comment, we have made substantial revisions, especially providing extensively new experimental results and the details of the method. By providing extensively more method details and experimental results, we believe the intermediate results are clearly presented in the current version and hope our current revision could fully address your concerns.

Our specific modifications in this revision are as follows:

1. We have provided all the discovered formulas in their raw format in our experiments of the revised SI for documenting the intermediate results.
2. To explain the methodology concretely, we have thoroughly rewritten the Methods section in the revised manuscript with rigorous notations and formulations.
3. We have significantly expanded the SI to add the detailed algorithms, network design and training methods.
4. We have revised the figures to better illustrate both the overall workflow and the novel ideas in core components.
5. To validate the effectiveness and efficiency of our novel designs in intermediate modules, we have also conducted extensively new experiments for complete ablation studies. In particular, the novelties and contributions of Deflex lies in the Lambda calculus-based symbolic regression, the energy-based neural network with decomposable pre-trained blocks. Therefore, our extensive ablation experiments include the effect of Lambda-aware mutation on SR efficiency, the impact of pre-training, the neural network hyperparameters like the block number, the data sampling strategies. The ablation studies demonstrate the effectiveness and efficiency of our main designs in our work.

Revised SI for Comment 3.1:

1.5 Successfully Discovered Formulas of All Methods

Supplementary Table 8 presents the full mathematical expressions of the laws discovered by Deflex and the baseline methods. These formulas represent the original outputs obtained from the symbolic regression process, corresponding to the experimental results discussed in the main text. Most Deflex formulas are expressed in the energy form (E), while the baseline methods' formulas are either equations (for deterministic laws, e.g., momentum conservation, continuity,

32

Newton viscosity law) or probability distributions (P, e.g., Maxwell-Boltzmann distribution, Lévy flight).

Supplementary Table 8: Full forms of discovered formulas by all compared methods

Law Name	Methods	Formula Form
Energy conservation	Deflex	E = 0.992 * mass * (vel_x^2 + vel_y^2 + vel_z^2) / (k_B * temperature)
	Operon	E = mass * (vel_x^2 + vel_y^2 + vel_z^2) / (k_B * temperature)
	SciMED	E = mass * (vel_x^2 + vel_y^2 + vel_z^2) / (k_B * temperature)
	SINDy	E = mass * (vel_x^2 + vel_y^2 + vel_z^2) / (k_B * temperature)
	gplearn	E = mass * (vel_x^2 + vel_y^2 + vel_z^2) / (k_B * temperature)
	AI Feynman	E = mass * (vel_x^2 + vel_y^2 + vel_z^2) / (k_B * temperature)
	PySR	E = mass * (vel_x^2 + vel_y^2 + vel_z^2) / (k_B * temperature)
	DEAP	E = mass * (vel_x^2 + vel_y^2 + vel_z^2) / (k_B * temperature)
Momentum conservation	Deflex	E = (sum(mass * vel_x) - C_x)^2 + (sum(mass * vel_y) - C_y)^2 + (sum(mass * vel_z) - C_z)^2
	Operon	sum(mass(i) * vel_x(i)) = C_x,<br>sum(mass(i) * vel_y(i)) = C_y,<br>sum(mass(i) * vel_z(i)) = C_z
	SciMED	sum(mass(i) * vel_x(i)) = C_x,<br>sum(mass(i) * vel_y(i)) = C_y,<br>sum(mass(i) * vel_z(i)) = C_z
	SINDy	sum(mass(i) * vel_x(i)) = C_x,<br>sum(mass(i) * vel_y(i)) = C_y,<br>sum(mass(i) * vel_z(i)) = C_z
	gplearn	sum(mass(i) * vel_x(i)) = C_x,<br>sum(mass(i) * vel_y(i)) = C_y,<br>sum(mass(i) * vel_z(i)) = C_z
	AI Feynman	sum(mass(i) * vel_x(i)) = C_x,<br>sum(mass(i) * vel_y(i)) = C_y,<br>sum(mass(i) * vel_z(i)) = C_z
	PySR	sum(mass(i) * vel_x(i)) = C_x,<br>sum(mass(i) * vel_y(i)) = C_y,<br>sum(mass(i) * vel_z(i)) = C_z
	DEAP	sum(mass(i) * vel_x(i)) = C_x,<br>sum(mass(i) * vel_y(i)) = C_y,<br>sum(mass(i) * vel_z(i)) = C_z
Maxwell-Boltzmann (MBD)	Deflex	E = 0.511 * mass * (vel_x^2 + vel_y^2 + vel_z^2) / (k_B * temperature)
	Operon	P ~ exp(-mass * (vel_x^2 + vel_y^2 + vel_z^2) / (2 * k_B * temperature))
	SciMED	P ~ exp(-mass * (vel_x^2 + vel_y^2 + vel_z^2) / (2 * k_B * temperature))
	gplearn	P ~ exp(-mass * (vel_x^2 + vel_y^2 + vel_z^2) / (2 * k_B * temperature))
	AI Feynman	P ~ exp(-mass * (vel_x^2 + vel_y^2 + vel_z^2) / (2 * k_B * temperature))
	PySR	P ~ exp(-mass * (vel_x^2 + vel_y^2 + vel_z^2) / (2 * k_B * temperature))
	DEAP	P ~ exp(-mass * (vel_x^2 + vel_y^2 + vel_z^2) / (2 * k_B * temperature))
MBD with potential	Deflex	E = 0.506 * mass * (vel_x^2 + vel_y^2 + vel_z^2) / (k_B * temperature)<br>+ sum(0.97 * ((pos_x(i)-pos_x(j))^2+(pos_y(i)-pos_<br>- 1.89 * ((pos_x(i)-pos_x(j))^2+(pos_y(i)-pos_y(j)
Continuity of quality	Deflex	E = (vel_x_dx + vel_y_dy)^2 * 10^26
	Operon	vel_x_dx + vel_y_dy = 0
	SciMED	vel_x_dx + vel_y_dy = 0
	SINDy	vel_x_dx + vel_y_dy = 0
	gplearn	vel_x_dx + vel_y_dy = 0
	AI Feynman	vel_x_dx + vel_y_dy = 0
	PySR	vel_x_dx + vel_y_dy = 0
	DEAP	vel_x_dx + vel_y_dy = 0

Law Name	Methods	Formula Form
Newton viscosity law	Deflex	τ_xy = −1.037 * μ * (vel_x_dy + vel_y_dx)
	Operon	τ_xy = −μ * (vel_x_dy + vel_y_dx)
	SciMED	τ_xy = −μ * (vel_x_dy + vel_y_dx)
	SINDy	τ_xy = −μ * (vel_x_dy + vel_y_dx)
	gplearn	τ_xy = −μ * (vel_x_dy + vel_y_dx)
	AI Feynman	τ_xy = −μ * (vel_x_dy + vel_y_dx)
	PySR	τ_xy = −μ * (vel_x_dy + vel_y_dx)
	DEAP	τ_xy = −μ * (vel_x_dy + vel_y_dx)
Velocity distribution	Deflex	E = 0.501 * mass * (vel_x^2 + vel_y^2) + (δv)^3.15
	AI Feynman	P ~ exp(−(0.5 * mass * (vel_x^2 + vel_y^2) + (δv)^p))
Navier-Stokes equation (2D Cylinder)	Deflex	E = (ρ * (vel_x_dt + vel_x * vel_x_dx + vel_y * vel_x_dy) + pressure_dx − μ * (vel_x_dx_dx + vel_x_dy_dy))^2 * 1.62e21 + (ρ * (vel_y_dt + vel_x * vel_y_dx + vel_y * vel_y_dy) + pressure_dy − μ * (vel_y_dx_dx + vel_y_dy_dy))^2 * 1.62e21
	Operon	ρ * (vel_x_dt + vel_x * vel_x_dx + vel_y * vel_x_dy) + pressure_dx − μ * (vel_x_dx_dx + vel_x_dy_dy) = 0, ρ * (vel_y_dt + vel_x * vel_y_dx + vel_y * vel_y_dy) + pressure_dy − μ * (vel_y_dx_dx + vel_y_dy_dy) = 0

Law Name	Methods	Formula Form
Navier-Stokes equation (3D JHTDB)	Deflex	E = (ρ * (vel_x_dt + vel_x * vel_x_dx + vel_y * vel_x_dy + vel_z * vel_x_dz) + pressure_dx − μ * (vel_x_dx_dx + vel_x_dy_dy + vel_x_dz_dz))^2 * 3.27e21 + (ρ * (vel_y_dt + vel_x * vel_y_dx + vel_y * vel_y_dy + vel_z * vel_y_dz) + pressure_dy − μ * (vel_y_dx_dx + vel_y_dy_dy + vel_y_dz_dz))^2 * 3.27e21 + (ρ * (vel_z_dt + vel_x * vel_z_dx + vel_y * vel_z_dy + vel_z * vel_z_dz) + pressure_dz − μ * (vel_z_dx_dx + vel_z_dy_dy + vel_z_dz_dz))^2 * 3.27e21
Langevin	Deflex	E = (4.438e16 * mass * vel_x_dt + 1.003 * vel_x)^2 + (4.438e16 * mass * vel_y_dt + 1.003 * vel_y)^2 + (4.438e16 * mass * vel_z_dt + 1.003 * vel_z)^2
	Operon	4.468e16 * mass * vel_x_dt + vel_x = 0, 4.468e16 * mass * vel_y_dt + vel_y = 0, 4.468e16 * mass * vel_z_dt + vel_z = 0
Levy flight (Human)	Deflex	E = 2.37 * log(sqrt((lat(time) − lat(time−1))^2 + (lon(time) − lon(time−1))^2))
	AI Feynman	P ~ u^−2.37
	PySR	P ~ u^−2.37
	DEAP	P ~ u^−2.37
Levy flight (Avian)	Deflex	E = 1.627 * log(sqrt((lat(time) − lat(time−1))^2 + (lon(time) − lon(time−1))^2))
	AI Feynman	P ~ u^−1.627
	PySR	P ~ u^−1.627
	DEAP	P ~ u^−1.627
Power law	Deflex	E = 1.56 * log(l) + 0.00027 * l
	AI Feynman	P ~ l^−1.56 * exp(−0.00027 * l)

Law Name	Methods	Formula Form
Crowd power law	Deflex	`E = 1.63 * log(r(i)) - 0.037 * log(r(i)) * sum(I(sqrt((lat(j)-lat(i))^2 + (lon(j)-lon(i))^2)<2.46))`

Supplementary Table 8 lists the full, raw mathematical expressions discovered by Deflex and baseline methods. These expressions use the specific variable names defined in the feature tables (Supplementary Tables 1–5) and correspond to the standard mathematical notations presented in Table 1 of the main text. Note that most Deflex formulas are expressed in the energy form (E) to unify different types of physical laws, while baseline methods output either equations (for deterministic laws) or probability distributions (P). For the fluid dynamics experiments, we report results from both the 2D cylinder wake flow and the 3D JHTDB turbulence dataset, demonstrating Deflex's ability to discover the Navier-Stokes equation across different spatial dimensions and flow regimes. For clarity, the mapping between the symbols in the main text and the variable names in the Supplementary Information is as follows: The velocity vectors $\mathbf{v}$ (or $\mathbf{u}$ in fluid dynamics) correspond to the component features `vel_x`, `vel_y`, `vel_z`. The position vectors $\vec{x}$ correspond to `pos_x`, `pos_y`, `pos_z` (or `lat`, `lon` in mobility tasks). Particle or individual indices i, j are represented using parentheses, e.g., `pos_x(i)`, `mass(i)`, `lat(j)`. Time indices are similarly denoted by `lat(time)` and `lat(time-1)` for current and previous time steps. The displacement scalar Δx and interaction distances $\|\vec{x}_j - \vec{x}_i\|$ in the main text are explicitly expanded here as Euclidean distances, e.g., `sqrt((lat(time) - lat(time-1))^2 + (lon(time) - lon(time-1))^2)` or `sqrt((pos_x(i)-pos_x(j))^2 + ...)`. Differential operators such as ∇ and $\partial/\partial t$ are represented by suffix notation, where `_dx` and `_dt` denote partial derivatives with respect to x and t, respectively (e.g., $\frac{\partial u}{\partial x}$ becomes `vel_x_dx`). Global parameters like mass m, temperature T, and pressure p directly map to `mass`, `temperature`, and `pressure`. Greek letters such as μ (viscosity), ρ (density), τ (stress), and δ (variation) are retained in mathematical notation.

Revised Manuscript for Comment 3.1:

4 Methods

We present the details of the Deflex framework for discovering mathematical laws in complex systems, which can automatically learn concise mathematical formulas from the observed data of the systems.

Notation. Throughout this section, we use bold symbols to denote vectors and matrices (e.g., $\mathbf{X}$ for data, $\mathbf{x}$ for individual samples, $\boldsymbol{\theta}$ for model parameters). Specifically, $\mathbf{X} = \{\mathbf{x}_1, \ldots, \mathbf{x}_N\}$ represents the observation dataset with N samples, each $\mathbf{x}_i$ characterizing the dynamics of element i over time. We denote energy functions as $E(\cdot)$ or $E_{\boldsymbol{\theta}}(\cdot)$ when parameterized by $\boldsymbol{\theta}$, and use $\mathcal{E}_{\boldsymbol{\theta}}(\cdot)$ to specifically refer to the Deflexformer neural network approximation. The partition function is denoted as $Z_{\boldsymbol{\theta}}$, and probability distributions as $p(\cdot)$ or $Q_{\boldsymbol{\theta}}(\cdot)$ for model distributions.

4.1 Problem formulation

We consider describing a complex system by a set of measurable features $\mathbf{X} = (x_1, \ldots, x_D)$ over time t, where $\mathbf{X}$ denotes the feature vector. They are normally state variables describe the local, dynamic condition of individual components (or elements) within the system at a specific time (e.g., a particle's position and velocity), and optionally some global features characterize the system from a macroscopic level (e.g., a system-wide temperature). Suppose we can obtain an observation dataset with the features $\mathbf{X}(t)$ of N elements within a time period of T from the system. Specifically, we consider the dimensionality of $\mathbf{X}(t)$ to be D. This dataset can be denoted as $\mathbf{X} \in \mathbb{R}^{NTD}$. The observed data of the state variables can be represented as $\mathbf{X} = \{\mathbf{x}_1, \ldots, \mathbf{x}_N\}$, where $\mathbf{x}_i \in \mathbb{R}^{TD}$ characterizes element i's dynamics from time $t = 1$ to $t = T$. Note that, except for the category of global or elementary features, we have no prior knowledge about the relationships among the features $(x_1, \ldots, x_D)$.

Let $\mathcal{F}$ denote the set of all possible mathematical formulas, including equations, invariants, and distributions. The behaviors in the system can be described by a set of formulas $\mathbf{f} \subseteq \mathcal{F}$ on $\mathbf{X}$. Normally, $\mathbf{f} = \{f_1, \cdots, f_m\}$ (where $\forall f_i \in \mathbf{f}, f_i \in \mathcal{F}$) with m formulas is expected to satisfy two properties: 1) $\mathbf{f}$ should fit the observed data well, i.e., have a large likelihood. Specifically, we can define a certain loss function $\mathcal{L}(f_i, \mathbf{X})$ to measure how well $f_i \in \mathcal{F}$ fits the datasets $\mathbf{X}$, where lower loss indicates better fit. 2) $\mathbf{f}$ should be concise, that is, have low complexity in mathematical formulas, denoted as $R(f)$. Then, the goal of Deflex is to find the mathematical formulas $\mathbf{f}^* \subseteq \mathcal{F}$ that balance the trade-off between the complexity of the expression of the function and the fitness of the data (in terms of the expected loss over the observed data $\mathbf{X}$). This can be formulated as the Pareto optimal set of patterns $\mathbf{f}^* \subseteq \mathcal{F}$ that satisfies:

Revised Manuscript for Comment 3.1:

$$\forall \mathbf{g} \subseteq \mathcal{F},\ \sum_{f\in\mathbf{f}^*} \mathcal{L}(f,\mathbf{X}) \leq \sum_{g\in\mathbf{g}} \mathcal{L}(g,\mathbf{X}) \Rightarrow \sum_{f\in\mathbf{f}} R(f) \leq \sum_{g\in\mathbf{g}^*} R(g). \quad (3)$$

Existing symbolic regression (SR) methods explores to solve the above problem by evaluating large-scale candidate expressions. However, facing complex systems with multiscale rules, existing studies would suffer from three major challenges:

- Limited expressibility for higher-order relationships: Traditional expression trees struggle to represent mappings and higher-order functions autonomously, often resorting to rigid, pre-specified operators or suffering from explosive tree growth when modeling collective behaviors.
- Disjoint representations across scales: Existing approaches lack a unified framework to characterize and learn both deterministic laws (invariants) and stochastic patterns (distributions) that emerge at different scales.
- Inefficiency in complex search spaces: The search space for complex nonlinear formulas grows exponentially with the number of variables, making standard search algorithms computationally prohibitive for high-dimensional systems.

4.2 Deflexpressor: SR subsystem based on Lambda calculus

Traditional tree-based SR relies on first-order expression trees and manually specified higher-order primitives, which makes it difficult to learn relationships such as summation or mapping over massive interacting elements in complex systems. To this end, we first present a Lambda calculus-based SR system, Deflexpressor.

4.2.1 Expressing relationships by Lambda calculus

In particular, by using a Lambda-calculus-based expression space that natively supports function abstraction and recursion, Deflexpressor can express operations like summation and mapping directly rather than as fixed templates. Specifically, operations over collections can be written as recursively defined functions or higher-order maps, enabling the system to learn how to aggregate interactions from data without requiring pre-specified structures. For instance, we consider a common formula $E = \sum_{i\neq j} V(r_{ij})$ that describes the aggregation of a variable number of particle interactions. Traditional SR either requires hard-coding symbols or extremely deep trees, leading to an intractable search space that grows exponentially with the number of variables. In contrast, Deflexpressor can synthesize a recursive generator using the fixpoint operator:

$$\lambda\mathbf{x}.\,(\text{Fix }(\lambda f\lambda \text{xs}\lambda a.\text{ if (null xs) then } a \text{ else } f \text{ (tail xs) } (a + V(\text{head xs}))))\ 0\ \mathbf{x}. \quad (4)$$

Here, Fix introduces recursion, enabling the expression to iterate over the state list $\mathbf{x}$, updating an accumulator a with a physical potential $V(\cdot)$ until the list is empty. This demonstrates that the system constructs the algorithm of summation from scratch, covering cases where predefined summation templates are inapplicable or insufficient. Besides, we directly use the expression length as the complexity metric $R(f)$ in Pareto selection, enabling an explicit trade-off between data fitness and interpretability.

4.2.2 Extended Lambda calculus with type and array

Directly applying the Lambda calculus to symbolic regression (SR) is inefficient due to the combinatorial explosion of invalid structures. Random generation often produces syntactically legal but semantically meaningless expressions. For example, in a typed context $\mathbb{T} = \{\mathbb{R}, \mathbb{V}, \mathbb{R} \to \mathbb{R}, \dots\}$, an unconstrained system might generate an application (MN) where $M : \mathbb{R}$ is a scalar being applied as a function, or attempts to add a vector to a boolean. Such ill-typed terms (e.g., $\lambda x.x + 1$ where x : Vec but $+ : \mathbb{R} \to \mathbb{R} \to \mathbb{R}$) cannot be normalized to a valid value. To rigorously constrain the search space to the manifold of semantically valid expressions, we implement a type inference engine based on Algorithm W (see SI for details). This engine verifies each candidate term M through two steps. First, it checks internal consistency by solving type constraints. Specifically, terms with conflicting uses (e.g., $1 + (\lambda x.x)$, where a scalar is added to a function) are rejected as ill-typed. Second, it ensures the inferred type unifies with the target signature required by the physical task (e.g., $\mathbb{R}^n \to \mathbb{R}$). In particular, valid terms like $\lambda x.(x + 1)$ is retained, whereas those like 5 (scalar) are rejected if a function is expected. By enforcing lightweight type checks during expression generation and mutation, we ensure only type-consistent candidates are evaluated, substantially increasing search efficiency. Another challenge is representing multi-dimensional data, such as vectors. In pure Lambda calculus, a vector $\mathbf{v} \in \mathbb{R}^n$ must be encoded as a nested form over scalar variables like $\lambda f.fv_1(\lambda f.fv_2 \dots)$, making operations like traversing v_i require $O(n)$ recursive unpacking steps. This would lead SR to fall in traversing basic data structures, other than learning any physical law. To adapt the framework for scientific discovery, we predefine array Array$[\tau]$ as a built-in primitive data type, and provide $O(1)$-time access and basic operators (e.g., indexing and size). This allows formulas like $E = \sum v_i^2$ to be expressed as $\text{Fix}(\lambda f\mathbf{v}s.\text{if Empty}(\mathbf{v}) \text{ then } s \text{ else } f(\text{Tail}(\mathbf{v}))(s + \text{Head}(\mathbf{v})^2))\mathbf{v}0$ concisely without

Revised Manuscript for Comment 3.1:

the overhead of functional encoding, letting the search focus on the mathematical relationship rather than data representation details (formal definitions in SI).

4.2.3 Hybrid expression generation with rules and NNs

Exploring the vast space of Lambda expressions presents distinct challenges in terms of efficiency and coverage. Firstly, rule-based mutations (e.g., random subtree replacement) explore the space diffusively and often generate low-quality expressions. To boost search efficiency, we incorporate a causal autoregressive neural network based on the Transformer Decoder architecture (see SI for architecture details). This network is trained offline via self-supervised learning on a manually-prepared corpus of valid expression trees and their rule-based mutation pairs. By learning the probabilistic priors of valid structural transformations, the network can generate syntactically correct and type-consistent Lambda expressions either from scratch or by mutating existing ones. Note that, the produced expressions will be parsed and type-checked to ensure validity. Secondly, relying solely on a neural network can lead to mode collapse, where the search gets stuck in familiar patterns and misses novel solutions. To expand search coverage, we implement a hybrid sampling strategy with simulated annealing. The process transitions from predominantly rule-based generation (for broad exploration) to neural-guided generation (for exploitation). Third, expressions after multiple mutations often become bloated with redundancy. To maintain compact and interpretable forms, we enforce Lambda-specific mutations like β-reduction and common sub-expression elimination in the rule-based component (see SI for mutation details).

4.2.4 Initialization with global aggregation priors

While Deflexpressor can generate expressions of any computable functions via the Lambda calculus, standard random initialization tends to produce expression trees that operate on a fixed number of arguments. In such a case, it would be combinatorially expensive to evolve a structure that iteratively computes over a variable-length input list $\mathbf{H} = [\mathbf{h}_1, \ldots, \mathbf{h}_N]$ with N components from scratch, such as calculating the total energy by summing over the energy of N particles. This is because it requires the spontaneous emergence of recursive abstractions.

To accelerate this process without imposing hard constraints, we employ a heuristic initialization strategy that generates the seed expressions in a "Reduce-like" structure. Specifically, these seed expressions are written in the form of a Lambda abstraction $\lambda\mathbf{H}.\text{Reduce}(\text{kernel}, \mathbf{H})$. It means to apply a learnable binary operator, $\text{kernel}(\mathbf{s}, \mathbf{h})$, to sequentially update an accumulated state $\mathbf{s}$ by recursively taking each element $\mathbf{h}_i$ of $\mathbf{H}$ as the input. The recursive expression can be written as

$$\text{reduced}_{\ i} = \begin{cases} h_i, i = 1 \\ \text{kernel}\ (\text{ reduced}_{\ i-1}, h_i)\ ,\ \text{else} \end{cases} \tag{5}$$

In particular, the kernel is a randomly generated expression tree that defines the interaction logic between the accumulated state $\mathbf{s}$ and the current data element $\mathbf{h}_i$. This

initialization serves as a soft inductive bias. By providing a pre-formed loop mechanism, we allow the symbolic regression to focus immediately on discovering the local interaction laws (encoded in the kernel) that fit the data. Meanwhile, since these structures are standard Lambda terms, they are fully mutable. Subsequent evolutionary steps (e.g., subtree mutation, β-reduction) can alter the kernel, break the recursion, or discard the "Reduce-like" structure entirely if a simpler non-iterative law fits the data well. This provides an initial computational skeleton capable of traversing the entire dataset, which enables the evolutionary algorithm to optimize, modify, or dismantle as needed.

4.3 Distribution-based formula representation

In the following, we first use probability distributions to unify the formulas and use energy-based models (EBMs) [46–49] to learn the distribution-based formulas from the observed data $\mathbf{X}$ (optionally including the global context $\mathbf{c}$). Then the search space $\mathcal{F}$ of Deflex is the set of all lambda expressions with basic operators and predefined functions which map the input state data $\mathbf{X}$ to some scalar values $f_1(\mathbf{X}), \ldots, f_m(\mathbf{X})$ representing the energy of the state.

Revised Manuscript for Comment 3.1:

4.3.2 Charachterizing distribution with energy function.

The unified representation can tackle the scale gap issue in multiscale systems. However, without the ability to normalize probability density functions, SR cannot directly

Revised Manuscript for Comment 3.1:

discover the distribution-based formulas. Therefore, we further characterize distributions as energy functions under the energy-based models (EBM) framework, which are less restrictive in functional forms (i.e., without requiring a normalized probability).

EBM are probabilistic models inspired by the Boltzmann distribution in statistical physics. Let $p(\mathbf{X})$ denote a distribution over features $\mathbf{X}$. Then, $p(\mathbf{X})$ can be characterized as an energy function $E(\mathbf{X})$, which indicates different energy values over the features $\mathbf{X}$. A probability distribution function (PDF) $p(\mathbf{X})$ given by an EBM is:

$$p(\mathbf{X}) = \frac{1}{Z}\exp(-E(\mathbf{X})) \tag{6}$$

where $Z = \int \exp(-E(\mathbf{X}))d\mathbf{X}$ is the normalization factor (i.e., partition function) indicating the system's total energy and ensures the integral of the distribution equals 1. It can avoid complex normalization calculations and volume transformations for complex and multi-dimensional variables. Therefore, finding the optimal distribution $p^*(\mathbf{X})$ is essentially equivalent to finding the optimal energy function $E^*(\mathbf{X})$, which can be achieved by performing SR over observation data.

4.4 Deflexformer: energy-based NNs with decomposable blocks

Expressions of energy function can be inferred via SRs over the observation dataset. However, this can be computationally expensive and even infeasible for complex expressions. Generally, any complex $E(\cdot)$ can be expressed as the composition of a series of (e.g., k) base functions $E^i(\cdot)$, e.g., $E(\cdot) = E^1 \circ E^2 \circ \ldots \circ E^k(\cdot)$. So, we propose to approximate $E(\cdot)$ by training a deep neural network Deflexformer $\mathcal{E}_{\boldsymbol{\theta}}(\cdot)$ (parameterized by $\boldsymbol{\theta}$) with k decomposable blocks $\mathcal{E}^i(\cdot)$, each approximating a base function $E^i(\cdot)$ (where $i = 1, \ldots, k$). The decomposable structure enables penetrating each block $\mathcal{E}^i(\cdot)$ to derive explicit expressions of $E^i(\cdot)$, making SR feasible to discover complex expressions of $E(\cdot)$.

4.4.1 Architecture of Deflexformer

The Deflexformer network predicts the energy value for a given dataset using the aligned state sequences of elements and global context as inputs. The architecture comprises an input embedding layer, k stacked Deflexformer blocks, and an output projection module.

The input layer embeds raw observations X into a high-dimensional feature space via Fourier multiscale embedding (see SI), producing element-level representations $P_0 \in \mathbb{R}^{n\times t\times D_{model}}$ and global representations $G_0 \in \mathbb{R}^{c\times D_{model}}$, where n is the number of elements, t is the number of time frames, c is the number of global tokens, and $D_{model} = 64$ is the model dimension.

Each Deflexformer block $\mathcal{E}^i(\cdot)$ (Fig. 1a) transforms representations (P_{i-1}, G_{i-1}) into (P_i, G_i), approximating a base mathematical transformation $E^i(\cdot)$ in the energy decomposition $E(\cdot) = E^1 \circ E^2 \circ \ldots \circ E^k(\cdot)$. Each block comprises two sub-modules operating on the concatenated input of dimension $(c + nt) \times D_{model}$: (1) A point-wise transformation network implemented as a four-layer fully connected network with architecture $(D_{model}, 2D_{model}, 2D_{model}, D_{model})$ and ReLU activations, modeling element-wise evolution (e.g., the dynamics of an individual particle). (2) A multi-head cross-attention network with $h = 8$ heads for capturing the interactions among particles. The attention operates in two sequential stages (Fig. 1b): spatial mixing performs attention over the element dimension to capture inter-element interactions, while temporal mixing applies causal attention over the time dimension to model temporal dependencies. By factorizing attention into spatial and temporal axes, computational complexity is reduced from $O(nt)$ to $O(n + t)$. Residual connections and layer normalization are applied after each sub-module (see SI for detailed specifications).

The output module projects the final global representation G_k back to a scalar energy via inverse Fourier embedding $e = \mathcal{E}^{-1}(G_k)$ (see SI). For example, when modeling particle energy $E = \frac{1}{2}m_i\mathbf{v}_i^2 + U(\mathbf{x}_i)$, early blocks may capture kinetic energy contributions while later blocks model potential interactions, with their composition yielding the total energy.

4.4.2 Pre-training the Deflexformer block

Directly training Deflexformer $\mathcal{E}(\cdot)$ from scratch requires a vast amount of observation data. Yet, its blocks $\mathcal{E}^i(\cdot)$ are normally simple functions, which have a much smaller search space. To this end, we adopt a common two-stage training framework of pre-training and post-training in other transformer-based deep networks. In particular, a single Deflexformer block is first pre-trained with a large amount of synthetic data based on some specified relationships. Each Deflexformer block $\mathcal{E}^i(\cdot)$ represents a basic mathematical transformation, similar to some base functions (e.g., $\sin(\cdot)$) with a simple expression form. Therefore, we can pre-train the Deflexformer block on a synthetic dataset sampled from some known base functions. Specifically, we first call Deflexpressor to generate a pool of expressions for functions $f(\cdot)$. Then, based on these functions, we can sample numerous data pairs (x, y) to synthesize a pre-training dataset $\mathcal{D}$, where x is drawn from a Gaussian distribution and $y = f(x)$. Here, pre-training aims to fit a block model $\mathcal{E}'(\cdot)$ via supervised learning on the synthetic dataset. So the loss function is defined simply as the MSE between the predictions with labels, given the features of data pairs in the synthetic dataset. That is, $L_{\mathcal{E}'} = \sum_{i=1}^{|\mathcal{D}|} \mathrm{MSE}(y, \mathcal{E}'(x))$ (see SI for training details).

Revised Manuscript for Comment 3.1:

4.4.3 Post-training the full Deflexformer

Then, the full Deflexformer, composed of multiple pre-trained blocks and an output module, is post-trained on the observation data. Note that Deflexformer $\mathcal{E}_{\boldsymbol{\theta}}(\cdot)$ is an energy-based model for approximating the optimal energy function $E(\cdot)$. So, its PDF $p_{\boldsymbol{\theta}}(\cdot)$ should fit $p_{\text{data}}(\cdot)$ with a maximized likelihood $\mathcal{L}(\mathcal{E}_{\boldsymbol{\theta}}, \mathbf{X})$. Here, our likelihood function adopts the common expected log-likelihood over the data distribution in the EBM framework as

$$\mathcal{L}(\mathcal{E}_{\boldsymbol{\theta}}, \mathbf{X}) = \mathbb{E}_{\mathbf{x}\sim p_{\text{data}}}[\log p_{\boldsymbol{\theta}}(\mathbf{x})] = \mathbb{E}[\log \frac{1}{Z_{\boldsymbol{\theta}}} \exp(-\mathcal{E}_{\boldsymbol{\theta}}(\mathbf{x}))], \quad (7)$$

where $\boldsymbol{\theta}$ denotes the network parameters, and $Z_{\boldsymbol{\theta}}$ is the partition function dependent on $\boldsymbol{\theta}$. Then, we can train $\mathcal{E}_{\boldsymbol{\theta}}$ to maximize (7). A common training methodology is to

define a loss function as the negative log-likelihood (NLL):

$$L_{\boldsymbol{\theta}} = -\mathcal{L}(\mathcal{E}_{\boldsymbol{\theta}}, \mathbf{X}) = \frac{1}{N} \sum\nolimits_{i=1}^{N} (\mathcal{E}_{\boldsymbol{\theta}}(\mathbf{x}_i) + \log(Z_{\boldsymbol{\theta}})), \quad (8)$$

and adopt the Malkov Chain Monte Carlo (MCMC) method with Langevin sampling [50, 51] (see SI for detailed methodology and parameter setups). Crucially, this approach discovers probability distributions directly from raw data points via likelihood maximization, rather than fitting predefined probability density functions (PDFs) to pre-computed statistics.

4.4.4 Extracting intermediate representations by inference

The post-trained Deflexformer $\mathcal{E}_{\boldsymbol{\theta}}$ approximates an energy function $E(\cdot)$ while the post-trained block $\mathcal{E}^i$ corresponds to the base functions $E^i(\cdot)$ within $E(\cdot)$. As P_i and G_i denote the element-level and global-level representations output by the $\mathcal{E}^i$ (P_0 and G_0 correspond to the representations of raw data $\mathbf{X}$), there should be $P_i||G_i = \mathcal{E}^i(P_{i-1}||G_{i-1})$ where $P_i||G_i$ means the concatenation of representations. More generally, there is $P_i||G_i = \mathcal{E}^{i-l} \circ \cdots \circ \mathcal{E}^i(P_{i-l-1}||G_{i-l-1})$. Therefore, to identify the relationships $E^{i-l} \circ \cdots \circ E^i$, we can perform SR on a large number of input-output representation pairs $(P_{i-l-1}||G_{i-l-1}, P_i||G_i)$ by inferring Deflexformer $\mathcal{E}_{\boldsymbol{\theta}}$. Here, we can feed each sample in the observation data into Deflexformer to conduct forward computation over a block array $\mathcal{E}^{i-l} \circ \cdots \circ \mathcal{E}^i$ of multiple sequential blocks and produce the corresponding intermediate representations. After multiple inferences, we can collect a dataset to perform SR, thus identifying any relationship represented by an array with an arbitrary number of sequential blocks. However, the number of collected samples may be constrained by that of the observation dataset, thereby limiting SR accuracy. Fortunately, as an energy model, Deflexformer inherently supports generating more data samples with a similar distribution to the original observation data. Specifically, it can work by using Langevin sampling from random noise (see SI for sampling details).

4.4.5 Hierarchical SR from intermediate representations

SR for a smaller block array with fewer Deflexformer blocks can reduce the computation complexity, but may converge to fragmented expressions with no meaning or oversimplified with larger decomposition error. Then their composition would lead to significantly biased energy functions. Nevertheless, these incomplete/inaccurate expressions can serve as base expressions to accelerate the SR for a larger block array, which has smaller decomposition errors. To this end, we propose a hierarchical SR strategy for recovering the whole energy function. Here, denote $\hat{E}^{i-l} \circ \ldots \circ \hat{E}^k \leftarrow SR(P_{i-l-1}||G_{i-l-1}, P_k||G_k)$ as SR for the l consecutive blocks indexed by $k-l, k-l+1, \ldots, k$. let $k = 1, 2, \ldots, K$ denote the block index. We perform the SRs by building a binary tree $\mathcal{T}$ with the depth of $\lceil \log K \rceil$. For each node v in $\mathcal{T}$, we denote $\mathcal{T}(v)$ as the sub-tree with node v as its root. In $\mathcal{T}$, each node v represents an SR process over the consecutive blocks indexed by the leaf nodes of the subtree $\mathcal{T}(v)$. The data samples (input-output represent pairs) for SR_v are constructed by

extracting the pair of the input representation of $\mathcal{T}(v)$'s leftmost leaf node and the corresponding output representation of $\mathcal{T}(v)$'s rightmost leaf node. In particular, for the leaf layer, the base expression set for SR is user-specified. For other layers in the tree, the base expression set for any node v is constructed by taking the union of the SR-resulted expression set of its two children. Up to the root, we can gradually refine the SR results and finally obtain an expression set $\{\hat{E}_1, \ldots, \hat{E}_m\}$ for insp. Although this incurs $O(K \log K)$ SRs, it can greatly reduce the searching space of SR for complex relationships (see SI for detailed algorithm).

Comment 3.2:

On the other hand, the evaluation section has been extended significantly, but is still very superficial. Statistically sound analysis including cross validation, mean / std / p-values, etc is still

missing.

Response to Comment 3.2:

We thank the reviewer for this comment and agree statistically sound analysis is essential for solidating our work. We have made substantial revision for much more detailed and convincing results, which are as follows.

First, to improve the soundness of our evaluation, we now employ test-set cross-validation for all datasets. To this end, we have updated the experimental results in Figure 4 of original manuscript (Figure 3 of revised manuscript) to include the error bars, which indicate both the mean value and standard variance.

Second, as detailed in our Response to Comment 3.2, we have conducted a series of ablation studies to validate the effectiveness and efficiency of different components of our method. These additional results with statistical analysis, presented and analyzed in both revised manuscript and SI, validate the soundness of our hyperparameter choices and our overall methodological design.

Regarding the p-value analysis, it is inapplicable since our symbolic regression prior does not involve an explicit null hypothesis. However, we have incorporated other measures to validate our findings. In Subsections 2.7 and 2.8 of the revised manuscript, we have now provided a more thorough discussion on the reliability of our experimental outcomes, with a particular focus on the robustness of our method compared to other methods.

Revised Manuscript for Comment 3.1:

2.7 Comparison on Benchmark Dataset

We finally evaluated Deflex on the public benchmark dataset of Feynman Symbolic Regression Database [7]. This benchmark dataset is generated by sampling from 120 classical formulas spanning different fields of physics, with added noise to simulate real-world conditions. It captures essential characteristics of real-world mathematical formulas, such as symmetry, while also being a classic, well-established set of problems for symbolic regression. This dual characteristic facilitates a feasible performance comparison against other SR-based AI methods. In the experiment, Deflex was compared against Operon, SciMED, gplearn, AI Feynman 2.0, and PySR. We compared their performance by measuring the trade-off between the average RMSE of discovered formulas and the total runtime for the discovery. Note that SINDy was not included as it is sparse regression-based and not designed for symbolic regression tasks, while DEAP was omitted since it works as the backend of gplearn in the current task.

As shown in Fig. 3d, Deflex demonstrates a quite competitive performance among all compared methods on the benchmark dataset in terms of the accuracy-efficiency tradeoff, although it is not specialized for these traditional, closed-form equations. In particular, Deflex is only slightly outperformed by Operon, which relies on a fixed symbol set and is optimized for runtime. In our experiment, Deflex managed to discover the formula with an RMSE of 0.075 in just 146.2 seconds, which was quite close to Operon with an RMSE of 0.0663 in 135.8 seconds. And Deflex and Operon show significant advantages over other methods. This high efficiency is largely attributed to the pre-training mechanism, which significantly accelerates formula discovery by incorporating mathematical priors (Fig. 5a, d).

2.8 Ablation Analysis

To validate the contribution of each component in Deflex, we conducted systematic ablation studies across multiple dimensions (Fig. 5). We first examined the impact of pre-training on discovery efficiency by varying the synthetic dataset size from 0 to 10^6 formulas. Results demonstrate that larger pre-training volumes lead to substantially faster convergence and higher formula recovery rates (Fig. 5a, d). Pre-training with 10^6 examples achieved over 80% recovery rate within 5,000 steps, while training from

scratch required more than 20,000 steps to reach similar performance, confirming that incorporating mathematical priors through pre-training is crucial for efficient exploration of the formula space.

Architectural ablations reveal that both network depth and embedding dimensions critically affect the model's capacity to capture complex system dynamics. Increasing the number of Deflexformer blocks from 1 to 8 progressively improves performance, with NLL loss decreasing from approximately 1.8 to 0.6 (Fig. 5b, e). Similarly, expanding embedding dimensions from 32 to 128 enhances the model's representational power (Fig. 5c, f). However, performance saturates beyond 6 blocks and 128 dimensions, indicating that these configurations provide sufficient capacity for the tested systems. Moreover, the Deflexformer effectively internalizes physical constraints, autonomously learning energy and momentum conservation manifolds (Fig. 5h). Finally, comparing data sampling strategies demonstrates that Langevin sampling is essential for accurately identifying complex equilibrium distributions (Fig. 5g).

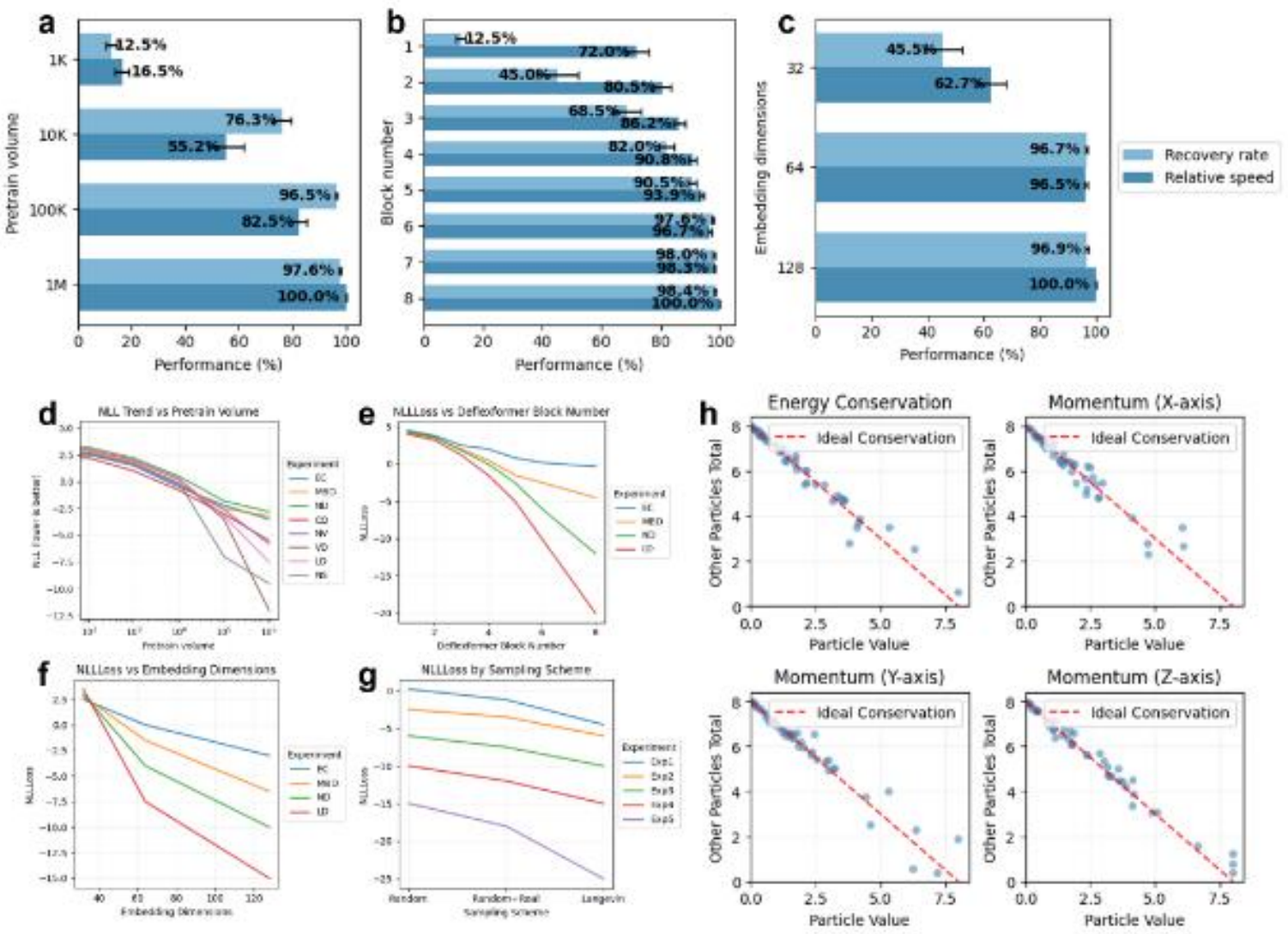

Fig. 4 Ablation studies and component analysis of Deflex. a, d, Impact of pre-training volume on formula discovery performance (recovery rate and relative speed) and NLL trend. Larger pre-training volumes significantly accelerate discovery and improve accuracy. **b, e,** Performance metrics and NLL loss with varying Deflexformer block numbers. Increasing depth improves representation power. **c, f,** Effect of embedding dimensions on performance. **g,** NLL loss comparison across different sampling schemes (Random, Random+Real, Langevin). Langevin sampling yields the best fitness. **h,** Verification of conservation laws (Energy and Momentum) learned by the Deflexformer, showing close alignment between sampled states (blue dots) and ideal laws (red lines).

=======Reviewer #4=======

Comments to the Authors:

This work presents a method for discovering equations from data through a combination of deep learning and symbolic regression. The authors refer to this approach as Deflex. This method consists of training deflexformer blocks (modified transformers) to find function approximations for energy models, and then using deflexpressor with symbolic regression to fit the deflexformer latent functions. An advantage of this approach is that it allows for the symbolic regression to solve a series of subproblems for each of the deflexformer blocks, as opposed to solving the entire symbolic regression straight from data. The method is validated on many example problems, including: Argon particles, pollen particles, human mobility, bird migration, water particles, flow around a cylinder, and on a benchmark database.

Overall, this paper presents a new method for equation discovery that yields impressive results. The authors do an excellent job of validating the approach across a variety of problems and of comparing the approach to a variety of other methods. The main shortcomings are in the presentation of Deflex and in the justifications for why it works. In the following section, I list the major and minor comments I had while reading the paper.

Response to Reviewer #4:

We thank the reviewer for their insightful feedback and positive assessment of our work. We fully agree that the manuscript would be greatly improved by a clearer presentation of the method and a more thorough justification for its effectiveness.

Comment 4.1:

I have a few concerns and questions about the fluid dynamics problem:

Citation [10] shows that SINDy finds the Navier-Stokes Eq. (NSE) with and without noise. Why are you unable to find the correct equation using SINDy in this paper?

Response to Comment 4.1:

The authors thank the reviewer for this comment. After a careful review of our experiments, we found the reason was that our experimental setup and datasets were different from [10] (reference [14] in the revised manuscript). The study [10] evaluated a much shorter form of the NSE expression based on a larger dataset. Instead, our considered NSE expression was longer and we used a smaller dataset. Specifically, the NSE expression in [10] has much smaller depth of the symobolic tree than ours. It has only five terms with the depth of 16 but ours has at least 10 terms with the depth of 28. Although the expression in [10] in more concise, it may not be the pareto-optimal between the accuracy and conciseness. Please also note that, we enforced a same time budget of 20, 000 seconds to all comparative frameworks in all tasks.

To respond this comment, we have re-conducted the corresponding experiments under an aligned setup with [10]. The experimental results in the same setup indeed validated that SINDy can find NS equation. Nevertheless, in this new setting, Deflex still demonstrates to be much more efficient than SINDy. Both new experimental setups and results have been added in both manuscript and SI.

Revised Manuscript for Comment 4.1:

Table 1 Discovered expressions by Deflex in comparison with the ground truths

Rules		Ground truths and discovered by Deflex
Energy conservation	True	$E = v^2 \cdot m/k_BT$
	Discovered	$E = 0.992v^2 \cdot m/k_BT$
Momentum conservation	True	$\sum m_i\mathbf{v}_i = C$
	Discovered	$\sum m_i\mathbf{v}_i = C$
Maxwell-Boltzmann distribution (MBD)	True	$E = \frac{mv^2}{2k_BT}$
	Discovered	$E = \frac{0.511mv^2}{k_BT}$
MBD with potential	True	$E = \frac{mv^2}{2k_BT} + \sum_{j\neq i}\left(\lVert\vec{x_j} - \vec{x_i}\rVert^{-12} - 2\lVert\vec{x_j} - \vec{x_i}\rVert^{-6}\right)$
	Discovered	$E = \frac{0.506mv^2}{k_BT} + \sum_{j\neq i}\left(0.97\lVert\vec{x_j} - \vec{x_i}\rVert^{-12} - 1.89\lVert\vec{x_j} - \vec{x_i}\rVert^{-6}\right)$
Continuity of quality	True	$E = \lVert\nabla \cdot v\rVert \times \infty$
	Discovered	$E = \lVert\nabla \cdot v\rVert \times 10^{26}$
Newton viscosity law	True	$\tau_{ij} = -\mu\left(\frac{\partial u_i}{\partial x_j} + \frac{\partial u_j}{\partial x_i}\right)$
	Discovered	$\tau_{ij} = -1.037\mu\left(\frac{\partial u_i}{\partial x_j} + \frac{\partial u_j}{\partial x_i}\right)$
Velocity distribution	True	$E = \frac{1}{2}mv^2 + (\delta v)^P$
	Discovered	$E = 0.501mv^2 + (\delta v)^{3.15}$
Navier-Stokes equation	True	$E = \lVert\rho\left(\frac{\partial\mathbf{u}}{\partial t} + \mathbf{u}\cdot\nabla\mathbf{u}\right) + \nabla p - \mu\nabla^2\mathbf{u}\rVert \times \infty$
	Discovered	$E = \lVert\rho\left(\frac{\partial\mathbf{u}}{\partial t} + \mathbf{u}\cdot\nabla\mathbf{u}\right) + \nabla p - \mu\nabla^2\mathbf{u}\rVert \times 1.62 \times 10^{21}$
Langevin	True	$E = \lVert 4.468 \times 10^{16}\ \mathrm{m}\dot{v} + v\rVert^2$
	Discovered	$E = \lVert 4.438 \times 10^{16}m\dot{v} + 1.003v\rVert^2$
Levy flight (Human)	True	$E = D_1 \log \Delta x$
	Discovered	$E = 2.37 \log \Delta x$
Levy flight (Avian)	True	$E = D_2 \log \Delta x$
	Discovered	$E = 1.627 \log \Delta x$
Power law	True	$E = \alpha \log l + \gamma l$
	Discovered	$E = 1.56 \log l + 0.00027l$
Crowd power law	True	Undocumented
	Discovered	$E = 1.63 \log r_i - 0.037 \log r_i \sum_{j\neq i}\mathbb{I}\left(\left\lVert x_t^{(j)} - x_t^{(i)}\right\rVert < 2.46\right)$

k_B, δ, ρ, P, D_1, D_2, α, and γ are unprovided constants in the experiments. All other symbols denote the provided features of observation data in the experiments. Detailed explanations of these features are described in SI.

Comment 4.2:

SINDy is typically designed as a regression problem for the output du/dt. How are you setting up the problem such that SINDy can be used to find the continuity equation and the Newtonian constitutive equation?

Response to Comment 4.2:

We thank the reviewer for this comment. We have made adaptations to all the compared methods to ensure broader and more competitive baselines, which have already been described in both the manuscript (Subsection. 2.1 Compared Methods) and SI of our previous submissions.

It is correct that SINDy was originally designed for the regression task of identifying dynamical equations. However, existing study [1] has demonstrated that it is flexible and can be adapted to unsupervised tasks by incorporating a reward for non-trivial solutions. Specifically, an equation of the form F(x)=0 can be framed as a regression problem where the target output is always zero. By adding a non-triviality term to the scoring function, as proposed in [1], SINDy can be effectively utilized for this class of problems. This is precisely the approach we adopted in our adaption.

To respond this comment, we have revised the manuscript to highlight the adaptations to the compared methods. Besides, we have also clarified the details of the adaptations in the revised SI.

References of response to this comment:

[1] Rudy, S.H., Brunton, S.L., Proctor, J.L., Kutz, J.N.: Data-driven discovery of partial

differential equations. Science Advances 3(4), 1602614 (2017) https://doi.org/10.1126/sciadv.1602614

Revised Manuscript for Comment 4.2:

2.1 Compared Methods

We benchmark our approach against several leading AI methods for formula discovery, including state-of-the-art SR method Operon [31], typical physics-inspired SR method SciMED [27], leading sparse regression method SINDy [24], and AI Feynman 2.0 [6], as well as classic SR methods gplearn [32], PySR [33] and DEAP [34] in terms of accuracy, efficiency, and adaptability to multiscale systems.

Different from Deflex with energy models and lambda calculus, existing AI methods (mainly based on symbolic regression) often support limited forms of problems and formulas. To maximize comparability, we made several accommodations for these compared methods. For the comparatives (PySR, AI Feynman, and DEAP) lacking native support for high-order operators (e.g., map or sum), we added these operators to their symbol set manually. For the rest ones (Operon, SciMED, SINDy, and gplean) that are incompatible with these operators, we provided an alternative. That is, if such an operator was at the top level of the target formula, we applied the method for the sub-expressions only and then handled the top-level operators within the loss function manually. Similarly, for the methods unable to approximate distributions, we provided custom energy-based loss functions (see SI for comparison details). A method was considered to have failed a task if it still could not discover the rule after these best-effort accommodations.

2.2 Evaluation Metrics

Since uncovering formulas within a complex system may represent an open-ended challenge, we incorporate qualitative assessments, basic quantitative analyses, renormalization and coarse-graining techniques [35] in the performance evaluation.

For established or empirical formulas, we first qualitatively assess whether the structure and parameters of the discovered formulas align with the ground truth. The formulas of invariants are considered successful recovery if they can be expressed as the correct formula with an additional stochastic residual term. Table 1 lists all the discovered formulas by Deflex in the studied six systems, compared to the ground truths. It should be noted that, for those undocumented discoveries, we provide justifications for their possible meanings in the real world. For a reference, we also summarize the capability of the compared methods for discovering these formulas in Table 2.

To quantitatively evaluate the accuracy of discovered formulas, we consider two cases based on the different forms of formulas separately.

- For the formulas in the form of equations and invariants, we employ the relative mean square error (RMSE) between the prediction and the true value. Specifically, all compared methods are given the same convergence threshold. Note that, in such a case, the RMSEs of different methods are hardly comparable since there is almost no structural difference (except for residual terms with negligible errors) in the commonly identified formulas in these forms.
- For distribution-based formulas, we use the negative log-likelihood (NLL) loss defined in Eq. (8) in the method section, which measures the likelihood of the distribution fitting real data. The feasible methods were also set with the same convergence constraint. Note that we also report the RMSE to measure the fitting error of simple distributions for reference.

Fig 4a compares the NLL loss of Deflex's distribution discovery in comparison with other methods, and reports the RMSE on the commonly identified equations and invariants.

To evaluate the efficiency of Deflex, we run Deflex and other comparative algorithms on a workstation with 32 CPU cores working in parallel, and two Tesla V100 GPUs for GPU-accelerated computing (details shown in SI Sec. 3). We compare the time efficiency of Deflex with that of other methods by recording the run-time of finding the same or equivalent formulas from the same dataset, within the same tolerable error in NLL or RMSE. A discovery is considered successful when its error remains within a small margin of the residual error, defined as the minimum achievable discrepancy when fitting the ground truth formula to observations [6]. This baseline accounts for irreducible noise from measurements and stochastic fluctuations in the system (details in SI). Fig 4b shows the comparison results.

In our experiments, we employ different observation scales by varying the spatiotemporal scope of data sampling. Small-scale observations use localized sampling (e.g., selecting spatiotemporally neighboring data points) to capture fine-grained interactions and facilitate the discovery of governing equations, while large-scale observations employ uniform random sampling across the entire domain to reveal macroscopic statistical patterns such as distributions (see SI for detailed sampling protocols).

Comment 4.3:

How is the Newtonian constitutive equation found using these methods? With the norm of continuity and NSE, the value should go to 0, which is what is shown. But how can the shear stress be found like this? Does deflex just find different groupings of terms in the NSE? For example,

why wouldn't the equation for the total derivative also come out of this analysis (Du/Dt = ∂u/∂t + u·∇u)

Response to Comment 4.3:

We thank the reviewer for this comment. Newtonian constitutive equation was found in the form of energy function while we have recovered and presented their equation forms in the manuscript. For example, the energy function corresponds to the Momentum constitutive law is as follows.

Momentum conservation	Deflex	E = (sum(mass * vel_x) - 0.0045)^2 + (sum(mass * vel_y) - 0.0051)^2 + (sum(mass * vel_z) - 0.0022)^2

You are correct that these equations are described in the form that the norm of error should go to 0. Their corresponding energy function that we had discovered has been shown in SI.

You are also correct that we identified the shear stress from the corresponding term in the NSE. We highlighted them in our discovery formulas in Table 1 as they have a distinct physical meaning about the Newton's law of viscosity. Accordingly, the derivative *(Du/Dt = ∂u/∂t + u·∇u)* could also be identified in the say way. However, this derivative term does not have a clear physical meaning to our knowledge, so we did not include it in our discovered formulas in Table 1.

<u>To respond this comment, we have provided the raw equations found in our experiments and added corresponding explanations in the revised SI.</u>

Revised SI for Comment 4.3:

1.5 Successfully Discovered Formulas of All Methods

Supplementary Table 8 presents the full mathematical expressions of the laws discovered by Deflex and the baseline methods. These formulas represent the original outputs obtained from the symbolic regression process, corresponding to the experimental results discussed in the main text. Most Deflex formulas are expressed in the energy form (E), while the baseline methods' formulas are either equations (for deterministic laws, e.g., momentum conservation, continuity, Newton viscosity law) or probability distributions (P, e.g., Maxwell-Boltzmann distribution, Lévy flight).

Supplementary Table 8: Full forms of discovered formulas by all compared methods

Law Name	Methods	Formula Form
Energy conservation	Deflex	E = 0.992 * mass * (vel_x^2 + vel_y^2 + vel_z^2) / (k_B * temperature)
	Operon	E = mass * (vel_x^2 + vel_y^2 + vel_z^2) / (k_B * temperature)
	SciMED	E = mass * (vel_x^2 + vel_y^2 + vel_z^2) / (k_B * temperature)
	SINDy	E = mass * (vel_x^2 + vel_y^2 + vel_z^2) / (k_B * temperature)
	gplearn	E = mass * (vel_x^2 + vel_y^2 + vel_z^2) / (k_B * temperature)
	AI Feynman	E = mass * (vel_x^2 + vel_y^2 + vel_z^2) / (k_B * temperature)
	PySR	E = mass * (vel_x^2 + vel_y^2 + vel_z^2) / (k_B * temperature)
	DEAP	E = mass * (vel_x^2 + vel_y^2 + vel_z^2) / (k_B * temperature)
Momentum conservation	Deflex	E = (sum(mass * vel_x) - C_x)^2 + (sum(mass * vel_y) - C_y)^2 + (sum(mass * vel_z) - C_z)^2
	Operon	sum(mass(i) * vel_x(i)) = C_x, sum(mass(i) * vel_y(i)) = C_y, sum(mass(i) * vel_z(i)) = C_z
	SciMED	sum(mass(i) * vel_x(i)) = C_x, sum(mass(i) * vel_y(i)) = C_y, sum(mass(i) * vel_z(i)) = C_z
	SINDy	sum(mass(i) * vel_x(i)) = C_x, sum(mass(i) * vel_y(i)) = C_y, sum(mass(i) * vel_z(i)) = C_z
	gplearn	sum(mass(i) * vel_x(i)) = C_x, sum(mass(i) * vel_y(i)) = C_y, sum(mass(i) * vel_z(i)) = C_z

Revised SI for Comment 4.3:

Law Name	Methods	Formula Form
	AI Feynman	sum(mass(i) * vel_x(i)) = C_x, sum(mass(i) * vel_y(i)) = C_y, sum(mass(i) * vel_z(i)) = C_z
	PySR	sum(mass(i) * vel_x(i)) = C_x, sum(mass(i) * vel_y(i)) = C_y, sum(mass(i) * vel_z(i)) = C_z
	DEAP	sum(mass(i) * vel_x(i)) = C_x, sum(mass(i) * vel_y(i)) = C_y, sum(mass(i) * vel_z(i)) = C_z
Maxwell-Boltzmann (MBD)	Deflex	E = 0.511 * mass * (vel_x^2 + vel_y^2 + vel_z^2) / (k_B * temperature)
	Operon	P ~ exp(-mass * (vel_x^2 + vel_y^2 + vel_z^2) / (2 * k_B * temperature))
	SciMED	P ~ exp(-mass * (vel_x^2 + vel_y^2 + vel_z^2) / (2 * k_B * temperature))
	gplearn	P ~ exp(-mass * (vel_x^2 + vel_y^2 + vel_z^2) / (2 * k_B * temperature))
	AI Feynman	P ~ exp(-mass * (vel_x^2 + vel_y^2 + vel_z^2) / (2 * k_B * temperature))
	PySR	P ~ exp(-mass * (vel_x^2 + vel_y^2 + vel_z^2) / (2 * k_B * temperature))
	DEAP	P ~ exp(-mass * (vel_x^2 + vel_y^2 + vel_z^2) / (2 * k_B * temperature))
MBD with potential	Deflex	E = 0.506 * mass * (vel_x^2 + vel_y^2 + vel_z^2) / (k_B * temperature) + sum(0.97 * ((pos_x(i)-pos_x(j))^2+(pos_y(i)-pos_ - 1.89 * ((pos_x(i)-pos_x(j))^2+(pos_y(i)-pos_y(j)
Continuity of quality	Deflex	E = (vel_x_dx + vel_y_dy)^2 * 10^26
	Operon	vel_x_dx + vel_y_dy = 0
	SciMED	vel_x_dx + vel_y_dy = 0
	SINDy	vel_x_dx + vel_y_dy = 0
	gplearn	vel_x_dx + vel_y_dy = 0
	AI Feynman	vel_x_dx + vel_y_dy = 0
	PySR	vel_x_dx + vel_y_dy = 0
	DEAP	vel_x_dx + vel_y_dy = 0

Law Name	Methods	Formula Form
Newton viscosity law	Deflex	τ_xy = -1.037 * μ * (vel_x_dy + vel_y_dx)
	Operon	τ_xy = -μ * (vel_x_dy + vel_y_dx)
	SciMED	τ_xy = -μ * (vel_x_dy + vel_y_dx)
	SINDy	τ_xy = -μ * (vel_x_dy + vel_y_dx)
	gplearn	τ_xy = -μ * (vel_x_dy + vel_y_dx)
	AI Feynman	τ_xy = -μ * (vel_x_dy + vel_y_dx)
	PySR	τ_xy = -μ * (vel_x_dy + vel_y_dx)
	DEAP	τ_xy = -μ * (vel_x_dy + vel_y_dx)
Velocity distribution	Deflex	E = 0.501 * mass * (vel_x^2 + vel_y^2) + (δv)^3.15
	AI Feynman	P ~ exp(-(0.5 * mass * (vel_x^2 + vel_y^2) + (δv)^p))
Navier-Stokes equation (2D Cylinder)	Deflex	E = (ρ * (vel_x_dt + vel_x * vel_x_dx + vel_y * vel_x_dy) + pressure_dx - μ * (vel_x_dx_dx + vel_x_dy_dy))^2 * 1.62e21 + (ρ * (vel_y_dt + vel_x * vel_y_dx + vel_y * vel_y_dy) + pressure_dy - μ * (vel_y_dx_dx + vel_y_dy_dy))^2 * 1.62e21
	Operon	ρ * (vel_x_dt + vel_x * vel_x_dx + vel_y * vel_x_dy) + pressure_dx - μ * (vel_x_dx_dx + vel_x_dy_dy) = 0, ρ * (vel_y_dt + vel_x * vel_y_dx + vel_y * vel_y_dy) + pressure_dy - μ * (vel_y_dx_dx + vel_y_dy_dy) = 0

Revised SI for Comment 4.3:

Law Name	Methods	Formula Form
Navier-Stokes equation (3D JHTDB)	Deflex	E = (ρ * (vel_x_dt + vel_x * vel_x_dx + vel_y * vel_x_dy + vel_z * vel_x_dz) + pressure_dx − μ * (vel_x_dx_dx + vel_x_dy_dy + vel_x_dz_dz))^2 * 3.27e21 + (ρ * (vel_y_dt + vel_x * vel_y_dx + vel_y * vel_y_dy + vel_z * vel_y_dz) + pressure_dy − μ * (vel_y_dx_dx + vel_y_dy_dy + vel_y_dz_dz))^2 * 3.27e21 + (ρ * (vel_z_dt + vel_x * vel_z_dx + vel_y * vel_z_dy + vel_z * vel_z_dz) + pressure_dz − μ * (vel_z_dx_dx + vel_z_dy_dy + vel_z_dz_dz))^2 * 3.27e21
Langevin	Deflex	E = (4.438e16 * mass * vel_x_dt + 1.003 * vel_x)^2 + (4.438e16 * mass * vel_y_dt + 1.003 * vel_y)^2 + (4.438e16 * mass * vel_z_dt + 1.003 * vel_z)^2
	Operon	4.468e16 * mass * vel_x_dt + vel_x = 0, 4.468e16 * mass * vel_y_dt + vel_y = 0, 4.468e16 * mass * vel_z_dt + vel_z = 0
Levy flight (Human)	Deflex	E = 2.37 * log(sqrt((lat(time) − lat(time−1))^2 + (lon(time) − lon(time−1))^2))
	AI Feynman	P ~ u^−2.37
	PySR	P ~ u^−2.37
	DEAP	P ~ u^−2.37
Levy flight (Avian)	Deflex	E = 1.627 * log(sqrt((lat(time) − lat(time−1))^2 + (lon(time) − lon(time−1))^2))
	AI Feynman	P ~ u^−1.627
	PySR	P ~ u^−1.627
	DEAP	P ~ u^−1.627
Power law	Deflex	E = 1.56 * log(l) + 0.00027 * l
	AI Feynman	P ~ l^−1.56 * exp(−0.00027 * l)

Law Name	Methods	Formula Form
Crowd power law	Deflex	E = 1.63 * log(r(i)) − 0.037 * log(r(i)) * sum(I(sqrt((lat(j)−lat(i))^2 + (lon(j)−lon(i))^2)<2.46))

Supplementary Table 8 lists the full, raw mathematical expressions discovered by Deflex and baseline methods. These expressions use the specific variable names defined in the feature tables (Supplementary Tables 1–5) and correspond to the standard mathematical notations presented in Table 1 of the main text. Note that most Deflex formulas are expressed in the energy form (E) to unify different types of physical laws, while baseline methods output either equations (for deterministic laws) or probability distributions (P). For the fluid dynamics experiments, we report results from both the 2D cylinder wake flow and the 3D JHTDB turbulence dataset, demonstrating Deflex's ability to discover the Navier-Stokes equation across different spatial dimensions and flow regimes. For clarity, the mapping between the symbols in the main text and the variable names in the Supplementary Information is as follows: The velocity vectors v (or u in fluid dynamics) correspond to the component features vel_x, vel_y, vel_z. The position vectors $\vec{x}$ correspond to pos_x, pos_y, pos_z (or lat, lon in mobility tasks). Particle or individual indices i, j are represented using parentheses, e.g., pos_x(i), mass(i), lat(j). Time indices are similarly denoted by lat(time) and lat(time−1) for current and previous time steps. The displacement scalar Δx and interaction distances $\|\vec{x}_j - \vec{x}_i\|$ in the main text are explicitly expanded here as Euclidean distances, e.g., sqrt((lat(time) − lat(time−1))^2 + (lon(time) − lon(time−1))^2) or sqrt((pos_x(i)−pos_x(j))^2 + ...). Differential operators such as ∇ and $\partial/\partial t$ are represented by suffix notation, where _dx and _dt denote partial derivatives with respect to x and t, respectively (e.g., $\frac{\partial u}{\partial x}$ becomes vel_x_dx). Global parameters like mass m, temperature T, and pressure p directly map to mass, temperature, and pressure. Greek letters such as μ (viscosity), ρ (density), τ (stress), and δ (variation) are retained in mathematical notation.

Comment 4.4:

How do the large-scale observations work for this problem? I do not understand what is meant by: "In larger-scale random observations, Deflex extracts the velocity distribution of the fluid within the vortex flow." Also, what is the δ in the velocity distribution equation?

Response to Comment 4.4:

Thank you for this question. The scale of observation in our work refers to spatiotemporal scale of data, which depends on how we obtained the observation data via different sampling strategies. A smaller-scale observation means that the observation dataset was collected from a smaller scope in some clusters, which can reflect some clustered patterns. To achieve this, we adopted a two-level sampling approach: we first randomly sample an initial set of points across time (frames) and space (elements, which are grids in the context of the fluid dynamics problem), and then we randomly sampled additional points from the spatiotemporal vicinity of this initial set. In contrast, a large-scale observation means that the observation data was collected from a broader scope, which reflects the macro-scopic behaviors. In this case, we directly sampled a set of points randomly over time (frames) and space. This approach discourages the learning framework from focusing on local interactions, enabling it to instead capture more coarse-grained, global relationships. <u>To respond this point, we have revised the manuscript to clarify the objective and process.</u>

Regarding the term δ in the velocity distribution, it is an unprovided constant for the given experimental setup. We have claimed in Subsection 2.2 (Evaluation Metrics) of the revised manuscript that "*the formulas of invariants are considered successful recovery if they can be expressed as the correct formula with an additional stochastic residual term*", which is commonly adopted by other studies [1]. This is common for other symbolic regression methods while they often chose to report the raw value. <u>To respond this point, we have provided the raw equations and explanations in the revised SI.</u>

References to this comment:

[1] Udrescu, S.-M., Tan, A., Feng, J., Neto, O., Wu, T., Tegmark, M.: Ai feynman 2.0: Pareto-optimal symbolic regression exploiting graph modularity. Advances in Neural Information Processing Systems 33, 4860–4871 (2020)

Revised Manuscript for Comment 4.4:

1.4.4 Observation Scale and Sampling Strategy

The scale of observation refers to the spatiotemporal scope from which observational data $\mathbf{X}$ are collected. Unlike the coarse-graining scale factor λ that characterizes the physical resolution of the system itself, the observation scale characterizes the data sampling strategy, defining whether the collected data points reflect local clustered interactions or global macroscopic behaviors.

For small-scale observations, we adopt a two-level sampling approach to capture localized spatiotemporal patterns. First, we randomly sample an initial set of seed points across time (frames) and space (elements or spatial positions). Then, we draw additional sample points from the spatiotemporal vicinity of these seeds. For particle systems, we sample neighboring particles within a certain spatial radius around the seed particle. For continuous fields, we extract a compact spatial region (e.g., a 4×4 grid block) centered at the seed position. This localized sampling facilitates the discovery of governing equations such as differential equations.

For large-scale observations, we employ uniform random sampling over the entire spatiotemporal domain. Sample points are drawn independently and uniformly across all available frames and spatial elements, without preferential clustering. This promotes the discovery of macroscopic statistical patterns such as velocity distributions.

In practice, we alternate or combine both sampling strategies depending on the target discovery. For example, in fluid systems, small-scale observations via localized spatial regions facilitate the recovery of differential equations, while large-scale random sampling reveals emergent statistical patterns.

Revised SI for Comment 4.4:

Newton viscosity law) or probability distributions (P, e.g., Maxwell-Boltzmann distribution, Lévy flight).

Supplementary Table 8: Full forms of discovered formulas by all compared methods

Law Name	Methods	Formula Form
Energy conservation	Deflex	`E = 0.992 * mass * (vel_x^2 + vel_y^2 + vel_z^2) / (k_B * temperature)`
	Operon	`E = mass * (vel_x^2 + vel_y^2 + vel_z^2) / (k_B * temperature)`
	SciMED	`E = mass * (vel_x^2 + vel_y^2 + vel_z^2) / (k_B * temperature)`
	SINDy	`E = mass * (vel_x^2 + vel_y^2 + vel_z^2) / (k_B * temperature)`
	gplearn	`E = mass * (vel_x^2 + vel_y^2 + vel_z^2) / (k_B * temperature)`
	AI Feynman	`E = mass * (vel_x^2 + vel_y^2 + vel_z^2) / (k_B * temperature)`
	PySR	`E = mass * (vel_x^2 + vel_y^2 + vel_z^2) / (k_B * temperature)`
	DEAP	`E = mass * (vel_x^2 + vel_y^2 + vel_z^2) / (k_B * temperature)`
Momentum conservation	Deflex	`E = (sum(mass * vel_x) - C_x)^2 + (sum(mass * vel_y) - C_y)^2 + (sum(mass * vel_z) - C_z)^2`
	Operon	`sum(mass(i) * vel_x(i)) = C_x, sum(mass(i) * vel_y(i)) = C_y, sum(mass(i) * vel_z(i)) = C_z`
	SciMED	`sum(mass(i) * vel_x(i)) = C_x, sum(mass(i) * vel_y(i)) = C_y, sum(mass(i) * vel_z(i)) = C_z`
	SINDy	`sum(mass(i) * vel_x(i)) = C_x, sum(mass(i) * vel_y(i)) = C_y, sum(mass(i) * vel_z(i)) = C_z`
	gplearn	`sum(mass(i) * vel_x(i)) = C_x, sum(mass(i) * vel_y(i)) = C_y, sum(mass(i) * vel_z(i)) = C_z`

Law Name	Methods	Formula Form
	AI Feynman	sum(mass(i) * vel_x(i)) = C_x,<br>sum(mass(i) * vel_y(i)) = C_y,<br>sum(mass(i) * vel_z(i)) = C_z
	PySR	sum(mass(i) * vel_x(i)) = C_x,<br>sum(mass(i) * vel_y(i)) = C_y,<br>sum(mass(i) * vel_z(i)) = C_z
	DEAP	sum(mass(i) * vel_x(i)) = C_x,<br>sum(mass(i) * vel_y(i)) = C_y,<br>sum(mass(i) * vel_z(i)) = C_z
Maxwell-Boltzmann (MBD)	Deflex	E = 0.511 * mass * (vel_x^2 + vel_y^2<br>+ vel_z^2) / (k_B * temperature)
	Operon	P ~ exp(-mass * (vel_x^2 + vel_y^2 +<br>vel_z^2) / (2 * k_B * temperature))
	SciMED	P ~ exp(-mass * (vel_x^2 + vel_y^2 +<br>vel_z^2) / (2 * k_B * temperature))
	gplearn	P ~ exp(-mass * (vel_x^2 + vel_y^2 +<br>vel_z^2) / (2 * k_B * temperature))
	AI Feynman	P ~ exp(-mass * (vel_x^2 + vel_y^2 +<br>vel_z^2) / (2 * k_B * temperature))
	PySR	P ~ exp(-mass * (vel_x^2 + vel_y^2 +<br>vel_z^2) / (2 * k_B * temperature))
	DEAP	P ~ exp(-mass * (vel_x^2 + vel_y^2 +<br>vel_z^2) / (2 * k_B * temperature))
MBD with potential	Deflex	E = 0.506 * mass * (vel_x^2 + vel_y^2<br>+ vel_z^2) / (k_B * temperature)<br>+ sum(0.97 * ((pos_x(i)-pos_x(j))^2+(pos_y(i)-pos_<br>- 1.89 * ((pos_x(i)-pos_x(j))^2+(pos_y(i)-pos_y(j)
Continuity of quality	Deflex	E = (vel_x_dx + vel_y_dy)^2 * 10^26
	Operon	vel_x_dx + vel_y_dy = 0
	SciMED	vel_x_dx + vel_y_dy = 0
	SINDy	vel_x_dx + vel_y_dy = 0
	gplearn	vel_x_dx + vel_y_dy = 0
	AI Feynman	vel_x_dx + vel_y_dy = 0
	PySR	vel_x_dx + vel_y_dy = 0
	DEAP	vel_x_dx + vel_y_dy = 0

Law Name	Methods	Formula Form
Newton viscosity law	Deflex	τ_xy = -1.037 * μ * (vel_x_dy +<br>vel_y_dx)
	Operon	τ_xy = -μ * (vel_x_dy + vel_y_dx)
	SciMED	τ_xy = -μ * (vel_x_dy + vel_y_dx)
	SINDy	τ_xy = -μ * (vel_x_dy + vel_y_dx)
	gplearn	τ_xy = -μ * (vel_x_dy + vel_y_dx)
	AI Feynman	τ_xy = -μ * (vel_x_dy + vel_y_dx)
	PySR	τ_xy = -μ * (vel_x_dy + vel_y_dx)
	DEAP	τ_xy = -μ * (vel_x_dy + vel_y_dx)
Velocity distribution	Deflex	E = 0.501 * mass * (vel_x^2 + vel_y^2)<br>+ (δv)^3.15
	AI Feynman	P ~ exp(-(0.5 * mass * (vel_x^2 +<br>vel_y^2) + (δv)^p))
Navier-Stokes equation (2D Cylinder)	Deflex	E = (ρ * (vel_x_dt + vel_x * vel_x_dx +<br>vel_y * vel_x_dy) + pressure_dx<br>- μ * (vel_x_dx_dx + vel_x_dy_dy))^2 *<br>1.62e21<br>+ (ρ * (vel_y_dt + vel_x * vel_y_dx +<br>vel_y * vel_y_dy) + pressure_dy<br>- μ * (vel_y_dx_dx + vel_y_dy_dy))^2 *<br>1.62e21
	Operon	ρ * (vel_x_dt + vel_x * vel_x_dx +<br>vel_y * vel_x_dy) + pressure_dx - μ<br>* (vel_x_dx_dx + vel_x_dy_dy) = 0,<br>ρ * (vel_y_dt + vel_x * vel_y_dx +<br>vel_y * vel_y_dy) + pressure_dy - μ<br>* (vel_y_dx_dx + vel_y_dy_dy) = 0

Law Name	Methods	Formula Form
Navier-Stokes equation (3D JHTDB)	Deflex	E = (ρ * (vel_x_dt + vel_x * vel_x_dx + vel_y * vel_x_dy + vel_z * vel_x_dz) + pressure_dx − μ * (vel_x_dx_dx + vel_x_dy_dy + vel_x_dz_dz))^2 * 3.27e21 + (ρ * (vel_y_dt + vel_x * vel_y_dx + vel_y * vel_y_dy + vel_z * vel_y_dz) + pressure_dy − μ * (vel_y_dx_dx + vel_y_dy_dy + vel_y_dz_dz))^2 * 3.27e21 + (ρ * (vel_z_dt + vel_x * vel_z_dx + vel_y * vel_z_dy + vel_z * vel_z_dz) + pressure_dz − μ * (vel_z_dx_dx + vel_z_dy_dy + vel_z_dz_dz))^2 * 3.27e21
Langevin	Deflex	E = (4.438e16 * mass * vel_x_dt + 1.003 * vel_x)^2 + (4.438e16 * mass * vel_y_dt + 1.003 * vel_y)^2 + (4.438e16 * mass * vel_z_dt + 1.003 * vel_z)^2
	Operon	4.468e16 * mass * vel_x_dt + vel_x = 0, 4.468e16 * mass * vel_y_dt + vel_y = 0, 4.468e16 * mass * vel_z_dt + vel_z = 0
Levy flight (Human)	Deflex	E = 2.37 * log(sqrt((lat(time) − lat(time−1))^2 + (lon(time) − lon(time−1))^2))
	AI Feynman	P ~ u^−2.37
	PySR	P ~ u^−2.37
	DEAP	P ~ u^−2.37
Levy flight (Avian)	Deflex	E = 1.627 * log(sqrt((lat(time) − lat(time−1))^2 + (lon(time) − lon(time−1))^2))
	AI Feynman	P ~ u^−1.627
	PySR	P ~ u^−1.627
	DEAP	P ~ u^−1.627
Power law	Deflex	E = 1.56 * log(l) + 0.00027 * l
	AI Feynman	P ~ l^−1.56 * exp(−0.00027 * l)

Law Name	Methods	Formula Form
Crowd power law	Deflex	E = 1.63 * log(r(i)) − 0.037 * log(r(i)) * sum(I(sqrt((lat(j)−lat(i))^2 + (lon(j)−lon(i))^2)<2.46))

Supplementary Table 8 lists the full, raw mathematical expressions discovered by Deflex and baseline methods. These expressions use the specific variable names defined in the feature tables (Supplementary Tables 1–5) and correspond to the standard mathematical notations presented in Table 1 of the main text. Note that most Deflex formulas are expressed in the energy form (E) to unify different types of physical laws, while baseline methods output either equations (for deterministic laws) or probability distributions (P). For the fluid dynamics experiments, we report results from both the 2D cylinder wake flow and the 3D JHTDB turbulence dataset, demonstrating Deflex's ability to discover the Navier-Stokes equation across different spatial dimensions and flow regimes. For clarity, the mapping between the symbols in the main text and the variable names in the Supplementary Information is as follows: The velocity vectors $\mathbf{v}$ (or $\mathbf{u}$ in fluid dynamics) correspond to the component features `vel_x`, `vel_y`, `vel_z`. The position vectors $\vec{x}$ correspond to `pos_x`, `pos_y`, `pos_z` (or `lat`, `lon` in mobility tasks). Particle or individual indices i, j are represented using parentheses, e.g., `pos_x(i)`, `mass(i)`, `lat(j)`. Time indices are similarly denoted by `lat(time)` and `lat(time-1)` for current and previous time steps. The displacement scalar Δx and interaction distances $\|\vec{x}_j - \vec{x}_i\|$ in the main text are explicitly expanded here as Euclidean distances, e.g., `sqrt((lat(time) - lat(time-1))^2 + (lon(time) - lon(time-1))^2)` or `sqrt((pos_x(i)-pos_x(j))^2 + ...)`. Differential operators such as ∇ and $\partial/\partial t$ are represented by suffix notation, where `_dx` and `_dt` denote partial derivatives with respect to x and t, respectively (e.g., $\frac{\partial u}{\partial x}$ becomes `vel_x_dx`). Global parameters like mass m, temperature T, and pressure p directly map to `mass`, `temperature`, and `pressure`. Greek letters such as μ (viscosity), ρ (density), τ (stress), and δ (variation) are retained in mathematical notation.

Comment 4.5:

The details of what is happening in the simulation are not very important here. Only the fact that it is a numerical solution to the NSE. However, the simulation appears not to be very high-quality. The boundary conditions have a major influence on the flow due to the small domain. This means the results will differ from typical cylinder flow results. It would be useful to report the boundary conditions and the initial condition. Is this a startup flow? Lastly, I do find it odd to use simple periodic shedding when the example in Fig. 1b is turbulence, especially when trying to highlight the importance of multiscale flows (which is only the case for a turbulent flow). A much more interesting case study would have been to consider data from the Johns Hopkins turbulence database.

Response to Comment 4.5:

Many thanks for this insightful comment. We agree that the boundary and initial conditions are important for reproducing the simulations and validating the results. And yes, this is a startup flow. To respond, we have added corresponding descriptions in the revised SI to report the boundaries.

We also agree that the periodic shedding in Fig.1b is too simple to reflect multiscale systems. To respond, we have revised Fig.1 with large scale turbulence diagram. However, considering that conceptual Fig.1 carries little information and new results have been added, we have placed the revised Fig.1 in the revised SI instead of the main manuscript.

Last, we appreciate the suggestion to include the Johns Hopkins turbulence database, which is a high-quality benchmark. Following this suggestion, we have incorporated the 1024x1024x1024 3D isotropic turbulence data from the Johns Hopkins Turbulence Database (JHTDB) into our fluid dynamics experiments. The experimental results from this new, higher-quality dataset have been added in Subsection 2.6 of our revised manuscript, and they are consistent with our original findings. Please note that, as the JHTDB is a 3D fluid dynamic dataset, we have kept the original simulation dataset for comparison in 2D fluid dynamics.

Revised Manuscript for Comment 4.5:

2.6 Fluid Dynamics

We conducted experiments on fluids (Fig. 3a, b), one of the most typical complex systems in physics [43]. Specifically, we focused on the wake behind a cylinder in a two-dimensional flow. The specific experimental setup is detailed in SI Document S2.2. We built a 1024×1024 mesh for simulation, and each one of 1M grids was regarded as an element, containing the position, pressure, and velocity of the grid as the data fields, and 2048 time frames were collected in total. We collected 9 to 256 adjacent or random grids and 128 time frames as a data snippet for training.

To test the generalizability of our approach across different flow regimes and spatial dimensions, we conducted a second fluid dynamics experiment using the Johns Hopkins Turbulence Database (JHTDB) [44]. Unlike the confined cylinder wake flow, the JHTDB provides Direct Numerical Simulation of three-dimensional forced isotropic turbulence, representing a canonical case of fully developed turbulence without boundary effects. The dataset features a 1024^3 computational grid with a Taylor microscale Reynolds number $Re_\lambda \approx 433$. We extracted volumetric subsamples from the database, with each grid point containing the three-dimensional velocity field (u, v, w), pressure p, and their spatial derivatives. Following the same training protocol as the cylinder experiment, we sampled 9 to 256 spatial locations and 128 temporal snapshots as input data. Remarkably, Deflex successfully identified the three-dimensional Navier-Stokes equation from this turbulent dataset (Table 1, Fig. 3), capturing the full three-dimensional convective and diffusive terms. The complementary nature of these two experiments—2D laminar wake versus 3D turbulent flow—demonstrates Deflex's capability to discover governing equations across different flow regimes, spatial dimensions, and Reynolds numbers.

Comment 4.6:

I have many questions about the details of the architecture and training of Deflex: It is unclear what makes up the element and embedding dimensions of P . For example, in particle motion, the time frame, the mass, position, and velocity are embedded into 64 dimensions. How is this done? And, are the elements here supposed to represent the dimension of the batch size, or is there a separate batch dimension not included?

Response to Comment 4.6:

We thank the reviewer for this comment. The element dimensions mainly include the provided element-level features, e.g., the mass, position, and velocity. These features are mapped into a 64-dimensional vector P via Fourier multi-scale encoding, which provides a robust representation across heterogeneous features with different numerical range. Specifically, each feature is mapped into 4 embedding dimensions. Except for the embeddings of the provided features, the remaining dimensions are padded with 0. The batch size, on the other hand, is purely a training hyperparameter and is independent of the system's physical properties. For reasons of computational performance, we set the batch size to 4 in our experiments.

To make these architectural details clearer, we have revised the description of our neural network in the Methods section of the revised manuscript and added the details in the revised SI.

Revised Manuscript for Comment 4.6 :

4.4.1 Architecture of Deflexformer

The Deflexformer network predicts the energy value for a given dataset using the aligned state sequences of elements and global context as inputs. The architecture comprises an input embedding layer, k stacked Deflexformer blocks, and an output projection module.

The input layer embeds raw observations X into a high-dimensional feature space via Fourier multiscale embedding (see SI), producing element-level representations $P_0 \in \mathbb{R}^{n \times t \times D_{model}}$ and global representations $G_0 \in \mathbb{R}^{c \times D_{model}}$, where n is the number of elements, t is the number of time frames, c is the number of global tokens, and $D_{model} = 64$ is the model dimension.

Each Deflexformer block $\mathcal{E}^i(\cdot)$ (Fig. 1a) transforms representations (P_{i-1}, G_{i-1}) into (P_i, G_i), approximating a base mathematical transformation $E^i(\cdot)$ in the energy decomposition $E(\cdot) = E^1 \circ E^2 \circ \ldots \circ E^k(\cdot)$. Each block comprises two sub-modules operating on the concatenated input of dimension $(c + nt) \times D_{model}$: (1) A point-wise transformation network implemented as a four-layer fully connected network with architecture $(D_{model}, 2D_{model}, 2D_{model}, D_{model})$ and ReLU activations, modeling element-wise evolution (e.g., the dynamics of an individual particle). (2) A multi-head cross-attention network with $h = 8$ heads for capturing the interactions among particles. The attention operates in two sequential stages (Fig. 1b): spatial mixing performs attention over the element dimension to capture inter-element interactions, while temporal mixing applies causal attention over the time dimension to model temporal dependencies. By factorizing attention into spatial and temporal axes, computational complexity is reduced from $O(nt)$ to $O(n + t)$. Residual connections and layer normalization are applied after each sub-module (see SI for detailed specifications).

The output module projects the final global representation G_k back to a scalar energy via inverse Fourier embedding $e = \mathcal{E}^{-1}(G_k)$ (see SI). For example, when modeling particle energy $E = \frac{1}{2} m_i \mathbf{v}_i^2 + U(\mathbf{x}_i)$, early blocks may capture kinetic energy contributions while later blocks model potential interactions, with their composition yielding the total energy.

Revised SI for Comment 4.6:

1.8 Detailed Algorithms

The algorithmic implementation of Deflex is detailed below, encompassing data representation, neural network architecture, energy-based learning, and the overall workflow. This section provides the complete technical specifications, including network architectures, hyperparameter configurations, and pseudocode for all major computational modules.

1.8.1 Data Representation and Deflexformer Architecture

We denote the observation data as $\mathbf{X} = \{x_i\}_{i=1}^{N}$, where each x_i comprises n elements observed over t time frames. The Deflexformer network approximates an energy function $\mathcal{E}_\Theta : \mathbb{R}^{n\times t\times d_{in}} \to \mathbb{R}$ parameterized by Θ. Each Deflexformer block $\mathcal{E}^i(\cdot)$ transforms element-level representations $P_i \in \mathbb{R}^{n\times t\times D_{model}}$ and global representations $G_i \in \mathbb{R}^{c\times D_{model}}$ into updated states P_{i+1} and G_{i+1}. The embedding dimension is denoted d, the model hidden dimension $D_{model} = d_\ell$, the number of global tokens c, the number of Fourier scales S, and the data range R.

The Fourier-based multiscale embedding (Algorithm 1) maps input scalars to a high-dimensic vector space using a range of frequencies, capturing both fine-grained invariants and coarse-grained distributions. The inverse mapping (Algorithm 2) recovers scalar energy values. The Deflexformer architecture processes these representations via decomposable blocks (Algorithm 3) and a forward inference pass (Algorithm 4).

Algorithm 1 Fourier Multiscale Embedding

Input: Embedding dimension d, scale count S, range R, input $\mathbf{u} \in \mathbb{R}^{d_{\text{in}}}$ ($d_{\text{in}} \le d/(2S)$)
Output: Multiscale embedding $\mathbf{v} \in \mathbb{R}^d$

```
                                              ▷ Implements EMBED(u, d, S, R)
d_in ← LEN(u)
d_used ← 2S d_in
ASSERT(d_used ≤ d)                 ▷ Sine/cosine pairs must fit into the embedding
ω_0 ← π/R                          ▷ Base angular frequency covering [−R, R]
v ← ZEROVEC(d)                     ▷ Zero padding for unused channels
m ← 1
for s = 0 → S − 1 do
    ω ← 2^s ω_0
    for j = 1 → d_in do
        v_m ← sin(ω u_j)
        v_{m+1} ← cos(ω u_j)
        m ← m + 2
    end for
end for
while m ≤ d do
    v_m ← 0
    m ← m + 1
end while
return v
```

Algorithm 1 implements the Fourier multiscale embedding. The angular frequency $\omega = 2^s\omega_0$ generates sine-cosine pairs across scales s, establishing a harmonic basis that captures features at different resolutions. The base frequency $\omega_0 = \pi/R$ is calibrated to the data range R, ensuring the finest scale resolves variations across the full domain. Each input dimension u_j produces $2S$ embedding channels (sine and cosine at S scales), yielding a total of $d_{used} = 2Sd_{in}$

Revised SI for Comment 4.6:

used channels. Unused channels are zero-padded to maintain a fixed layout d for downstream blocks, facilitating parameter sharing across different input dimensionalities. This spectral representation is analogous to the positional encoding in Transformers but adapted for continuous-valued physical quantities, enabling the network to distinguish between fine-grained invariants (high-frequency components) and coarse-grained distributions (low-frequency components).

Algorithm 2 Inverse Fourier Multiscale Embedding

Input: Embedding dimension d, scale count S, range R, input width d_{in}, embedding $\mathbf{v} \in \mathbb{R}^d$ (layout per Alg. 1)

Output: Recovered signal $\hat{\mathbf{u}} \in \mathbb{R}^{d_{\text{in}}}$

```
                                        ▷ Implements EMBEDINV(v, d, S, R, d_in)
ASSERT(2·S·d_in ≤ d)
ω_0 ← π/R
û ← ZEROVEC(d_in)
                                        ▷ Coarse reconstruction from lowest frequency
for j = 1 → d_in do
    i ← 2(j − 1) + 1
    θ ← atan2(v_i, v_{i+1})
    û_j ← θ/ω_0
end for
                                        ▷ Gradient refinement with higher harmonics
for s = 1 → S − 1 do
    ω ← 2^s ω_0
    η_s ← 2^{−s}                        ▷ Diminishing step size for stability
    for j = 1 → d_in do
        i ← 2(s·d_in + j − 1) + 1
        θ̂ ← ω û_j
        e_sin ← sin(θ̂) − v_i
        e_cos ← cos(θ̂) − v_{i+1}
        g ← ω(e_sin cos(θ̂) − e_cos sin(θ̂))
        û_j ← û_j − η_s g
    end for
end for
return û
```

Algorithm 2 performs the inverse transformation from global channels to scalar energies. The reconstruction proceeds in two stages. First, principal angles are retrieved via $\text{atan2}(\sin(\omega_0 u), \cos(\omega_0 u))$

Comment 4.7:

Are all of the equations for a single system found in one pass of training the deflexformer through multiple symbolic regression passes? Or, is everything retrained for each equation?

Response to Comment 4.7:

We thank the reviewer for this comment. All equations for a single system are discovered in one pass of training the Deflexformer while performing multiple passes of symbolic regressions.

One pass of Deflexformer training is because the Deflexformer network approximates an energy function that unifies all equations via a mixed probabilistic distribution. In particular, in our work, all equations are modeled as probabilistic distributions, which are equivalent to a mixed distribution while a probabilistic distribution can be characterized by an energy function.

Multiple passes of symbolic regression correspond to different symbolic regressions for identifying the relationships (or sub-functions) approximated by each Deflexformer block or their cascaded modules. As you understand correctly, these symbolic regressions solve a series of subproblems for each of the Deflexformer blocks, instead of solving the entire symbolic regression straight from the observation data.

Also please note that, each symbolic regression will produce a set of equation expressions. And the final result of these symbolic regressions is also an expression set that includes all equations

that can describe the approximated energy function by one-pass trained Deflexformer.

To respond this comment, we have highlighted this in the revised manuscript.

Revised Manuscript for Comment 4.7:

> The workflow of our approach is depicted in Fig. 1. First, Deflexpressor generates a large number of formulas for sampling a synthetic dataset (Fig. 1a). The synthetic dataset is then used to pre-train a Deflexformer block, which is a Transformer-like neural network (Fig. 1b). This pre-training serves as prior knowledge for the subsequent step, where we cascade the multiple copies of the pre-trained block as the whole Deflexformer to learn patterns from the observation dataset of a certain complex system (Fig. 1c). During learning, Deflexformer employs element-wise and time-wise self-attention mechanisms to capture the interactions among different entities and time frames. Each trained block then essentially represents a distinct component of the latent formulas of the patterns. In this phase, the observation dataset, together with some random data, is then fed to the trained Deflexformer for inference, which generates a large volume of data representations output by all blocks (Fig. 1d). Finally, the representation dataset output by the elementary blocks in Deflexformer is fit with the Deflexpressor to obtain the desired formulas from each component, through iterative formula generation and mutation in the form of lambda expressions (Fig. 1e).

Comment 4.8:

It is challenging to understand the choices in the deflexpressor. Was there extensive manual tuning on how to select the types of mutations? Is this how you chose to add the new styles of mutation? Also, how does the autoregressive neural network work in this framework? How is this NN train?

Response to Comment 4.8:

Thanks for this comment. Almost no manual tuning in the selection of mutations. In particular, all mutations are generated automatically, some are randomly sampled from a pool of legal candidates while others are generated by the autoregressive neural network followed by a type-checking process to ensure validity.

The reviewer is correct that we add the new styles of mutations to support type-checking, which can ensure the validity of mutations.

The auto-regressive neural network is essentially a generative transformer model for generating new Lambda expressions. It can achieve automatic generation and mutations of diversed expressions much more efficiently than sampling from the known expression candidates. It takes an existing expression (represented as a sequence of tokens) as input and produce a new expression that is structurally similar. Repeat this process until a new expression can pass the type checking.

It was trained on a corpus of Lambda expressions. It is trained offline via a standard language modeling objective (next-token prediction)on a dataset constructed by the rule-based engine.The training corpus comprises 128,000 syntactically valid expressions for \textit{ab initio} generation and 54,000 expression pairs for mutation learning. In these pairs, the target was derived from the source through 2 to 5 steps of random rule-based mutations, capturing non-trivial structural transitions. We used the AdamW optimizer with a learning rate of 5×10^{-4} and a batch size of 32, training for 200 epochs. Since the length of lambda expressions is shorter, it is relatively easier to train.

To respond this comment and clarify the details, we have revised the description in the manuscript to add more details about the Lambda-calculas subsystem.

Revised Manuscript for Comment 4.8:

4.2 Deflexpressor: SR subsystem based on Lambda calculus

Traditional tree-based SR relies on first-order expression trees and manually specified higher-order primitives, which makes it difficult to learn relationships such as summation or mapping over massive interacting elements in complex systems. To this end, we first present a Lambda calculus-based SR system, Deflexpressor.

4.2.1 Expressing relationships by Lambda calculus

In particular, by using a Lambda-calculus-based expression space that natively supports function abstraction and recursion, Deflexpressor can express operations like summation and mapping directly rather than as fixed templates. Specifically, operations over collections can be written as recursively defined functions or higher-order maps, enabling the system to learn how to aggregate interactions from data without requiring pre-specified structures. For instance, we consider a common formula $E = \sum_{i \neq j} V(r_{ij})$ that describes the aggregation of a variable number of particle interactions. Traditional SR either requires hard-coding symbols or extremely deep trees, leading to an intractable search space that grows exponentially with the number of variables. In contrast, Deflexpressor can synthesize a recursive generator using the fixpoint operator:

$$\lambda \mathbf{x}.\,(\text{Fix } (\lambda f \lambda \text{xs} \lambda a.\ \text{if (null xs) then } a \text{ else } f \text{ (tail xs) } (a + V(\text{head xs})))) \ 0 \ \mathbf{x}. \quad (4)$$

Here, Fix introduces recursion, enabling the expression to iterate over the state list $\mathbf{x}$, updating an accumulator a with a physical potential $V(\cdot)$ until the list is empty. This demonstrates that the system constructs the algorithm of summation from scratch, covering cases where predefined summation templates are inapplicable or insufficient. Besides, we directly use the expression length as the complexity metric $R(f)$ in Pareto selection, enabling an explicit trade-off between data fitness and interpretability.

4.2.2 Extended Lambda calculus with type and array

Directly applying the Lambda calculus to symbolic regression (SR) is inefficient due to the combinatorial explosion of invalid structures. Random generation often produces syntactically legal but semantically meaningless expressions. For example, in a typed context $\mathbb{T} = \{\mathbb{R}, \mathbb{V}, \mathbb{R} \to \mathbb{R}, \dots\}$, an unconstrained system might generate an application (MN) where $M : \mathbb{R}$ is a scalar being applied as a function, or attempts to add a vector to a boolean. Such ill-typed terms (e.g., $\lambda x.x + 1$ where x : Vec but $+ : \mathbb{R} \to \mathbb{R} \to \mathbb{R}$) cannot be normalized to a valid value. To rigorously constrain the search space to the manifold of semantically valid expressions, we implement a type inference engine based on Algorithm W (see SI for details). This engine verifies each candidate term M through two steps. First, it checks internal consistency by solving type constraints. Specifically, terms with conflicting uses (e.g., $1 + (\lambda x.x)$, where a scalar is added to a function) are rejected as ill-typed. Second, it ensures the inferred type unifies with the target signature required by the physical task (e.g., $\mathbb{R}^n \to \mathbb{R}$). In particular, valid terms like $\lambda x.(x + 1)$ is retained, whereas those like 5 (scalar) are rejected if a function is expected. By enforcing lightweight type checks during expression generation and mutation, we ensure only type-consistent candidates are evaluated, substantially increasing search efficiency. Another challenge is representing multi-dimensional data, such as vectors. In pure Lambda calculus, a vector $\mathbf{v} \in \mathbb{R}^n$ must be encoded as a nested form over scalar variables like $\lambda f.fv_1(\lambda f.fv_2 \dots)$, making operations like traversing v_i require $O(n)$ recursive unpacking steps. This would lead SR to fall in traversing basic data structures, other than learning any physical law. To adapt the framework for scientific discovery, we predefine array Array$[\tau]$ as a built-in primitive data type, and provide $O(1)$-time access and basic operators (e.g., indexing and size). This allows formulas like $E = \sum v_i^2$ to be expressed as $\text{Fix}(\lambda f \mathbf{v} s.\text{if Empty}(\mathbf{v}) \text{ then } s \text{ else } f(\text{Tail}(\mathbf{v}))(s + \text{Head}(\mathbf{v})^2))\mathbf{v}0$ concisely without

the overhead of functional encoding, letting the search focus on the mathematical relationship rather than data representation details (formal definitions in SI).

4.2.3 Hybrid expression generation with rules and NNs

Exploring the vast space of Lambda expressions presents distinct challenges in terms of efficiency and coverage. Firstly, rule-based mutations (e.g., random subtree replacement) explore the space diffusively and often generate low-quality expressions. To boost search efficiency, we incorporate a causal autoregressive neural network based on the Transformer Decoder architecture (see SI for architecture details). This network is trained offline via self-supervised learning on a manually-prepared corpus of valid expression trees and their rule-based mutation pairs. By learning the probabilistic priors of valid structural transformations, the network can generate syntactically correct and type-consistent Lambda expressions either from scratch or by mutating existing ones. Note that, the produced expressions will be parsed and type-checked to ensure validity. Secondly, relying solely on a neural network can lead to mode collapse, where the search gets stuck in familiar patterns and misses novel solutions. To expand search coverage, we implement a hybrid sampling strategy with simulated annealing. The process transitions from predominantly rule-based generation (for broad exploration) to neural-guided generation (for exploitation). Third, expressions after multiple mutations often become bloated with redundancy. To maintain compact and interpretable forms, we enforce Lambda-specific mutations like β-reduction and common sub-expression elimination in the rule-based component (see SI for mutation details).

4.2.4 Initialization with global aggregation priors

While Deflexpressor can generate expressions of any computable functions via the Lambda calculus, standard random initialization tends to produce expression trees that operate on a fixed number of arguments. In such a case, it would be combinatorially expensive to evolve a structure that iteratively computes over a variable-length input list $\mathbf{H} = [\mathbf{h}_1, \ldots, \mathbf{h}_N]$ with N components from scratch, such as calculating the total energy by summing over the energy of N particles. This is because it requires the spontaneous emergence of recursive abstractions.

To accelerate this process without imposing hard constraints, we employ a heuristic initialization strategy that generates the seed expressions in a "Reduce-like" structure. Specifically, these seed expressions are written in the form of a Lambda abstraction $\lambda\mathbf{H}.\text{Reduce}(\text{kernel}, \mathbf{H})$. It means to apply a learnable binary operator, $\text{kernel}(\mathbf{s}, \mathbf{h})$, to sequentially update an accumulated state $\mathbf{s}$ by recursively taking each element $\mathbf{h}_i$ of $\mathbf{H}$ as the input. The recursive expression can be written as

$$\text{reduced}_{\ i} = \begin{cases} h_i, i = 1 \\ \text{kernel}\ (\text{reduced}_{\ i-1}, h_i),\ \text{else} \end{cases} \tag{5}$$

In particular, the kernel is a randomly generated expression tree that defines the interaction logic between the accumulated state $\mathbf{s}$ and the current data element $\mathbf{h}_i$. This initialization serves as a soft inductive bias. By providing a pre-formed loop mechanism, we allow the symbolic regression to focus immediately on discovering the local interaction laws (encoded in the kernel) that fit the data. Meanwhile, since these structures are standard Lambda terms, they are fully mutable. Subsequent evolutionary steps (e.g., subtree mutation, β-reduction) can alter the kernel, break the recursion, or discard the "Reduce-like" structure entirely if a simpler non-iterative law fits the data well. This provides an initial computational skeleton capable of traversing the entire dataset, which enables the evolutionary algorithm to optimize, modify, or dismantle as needed.

Revised SI for Comment 4.8:

Neural-guided Expression Generation. As a complement to the explicit rule-based mutations, Deflexpressor integrates a causal autoregressive neural network to generate more diverse expressions efficiently. The Deflexpressor network is essentially a Transformer decoder, configured with 6 attention layers, a hidden dimension of 64, and 8 attention heads. It operates on serialized token sequences of lambda expressions. It is trained offline via a standard language modeling objective (next-token prediction) on a dataset constructed by the rule-based engine. The training corpus comprises 128,000 syntactically valid expressions for *ab initio* generation and 54,000 expression pairs for mutation learning. In these pairs, the target was derived from the source through 2 to 5 steps of random rule-based mutations, capturing non-trivial structural transitions. We used the AdamW optimizer with a learning rate of 5×10^{-4} and a batch size of 32, training for 200 epochs. In particular, no special task tokens are required. The network implicitly distinguishes modes based on the input context. In the generation mode, it is iteratively called to produce an expression from an empty context until the expression conforms to the type signatures in type check. In the mutation mode, it uses an existing expression as a prompt to generate a structural variant that can pass the type check.

Comment 4.9:

The training process for the deflexformer is unclear. Is it also using the NLL loss? If so, how do you get f out of the deflexformer? Figure 3d makes it seem like each deflexformer block has a scalar output. However, I thought that there would be some layer after the final deflexformer block that would generate f . Or is there another loss used directly with PK , GK for training the deflexformer?

Response to Comment 4.9:

The authors thank the reviewer for this comment. Yes, we use NLL loss to train Deflexformer, which is an energy-based model (EBM) that approximates an energy function f. Specifically, the EBM Deflexformer approximates f is parameterized by \theta. Therefore, it can be trained by gradient descent of the NLL loss, where the gradient is calculated instead of f itself. You are correct that there is an output layer after the final Deflexformer block that outputs the energy value, which is then used to calculate the gradient. In particular, there is no other loss used.

The original Figure 3d only highlighted the repeated Deflexformer blocks and neglects both the input and output layer. <u>To make this clear, we have recreated the corresponding figure present the full network architecture of the network and revised the description in the manuscript.</u>

Revised Manuscript for Comment 4.9:

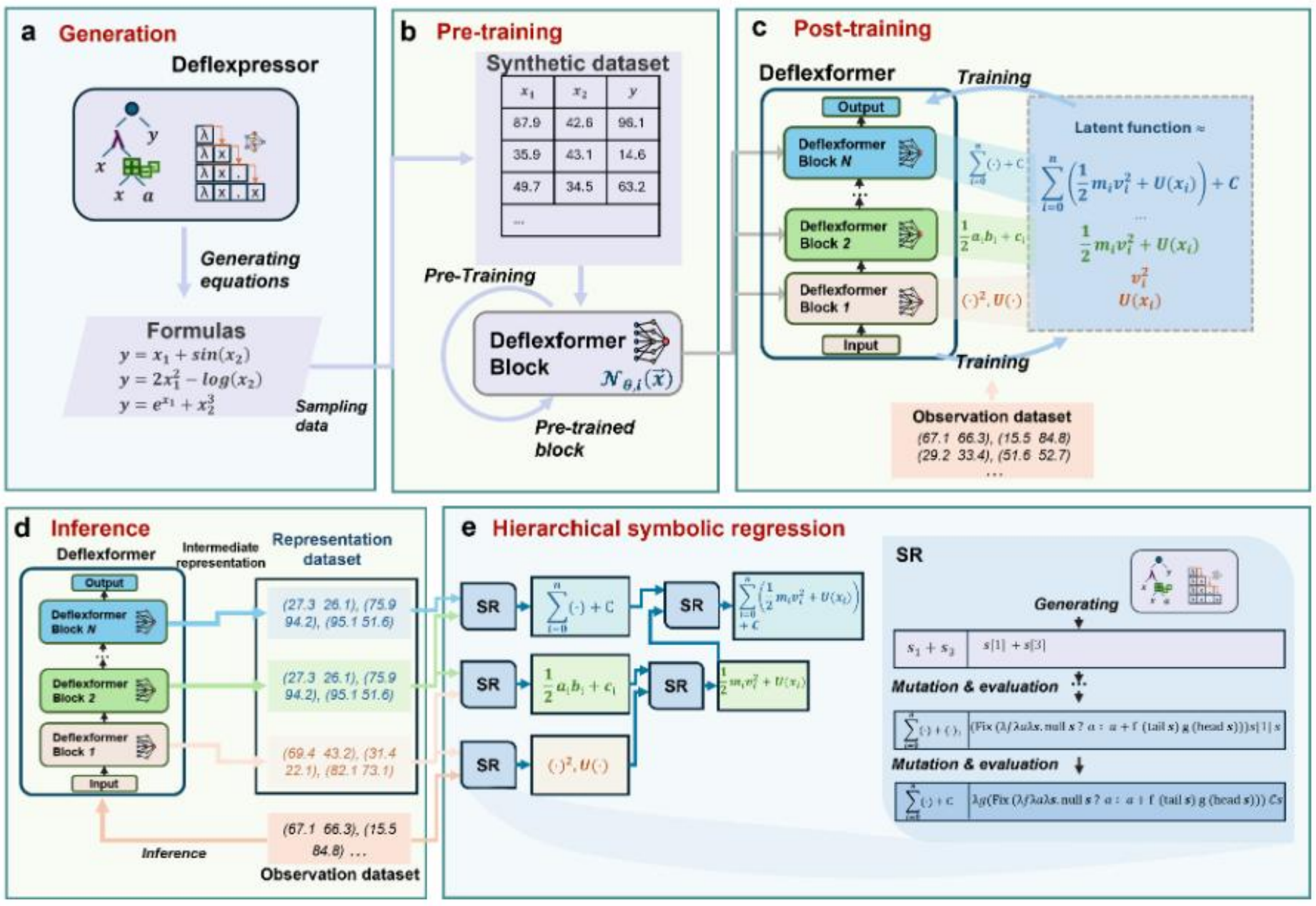

Fig. 1 Illustration of the workflow in Deflex. Deflex utilizes two subsystems: Deflexpressor and Deflexformer, where the former is a symbolic regression subsystem based on Lambda calculus and the latter is an energy-based DNN subsystem. The overall process flows sequentially from **a** through **b**, **c**, **d**, and finally to **e**. **a**, Deflexpressor generates a large number of expressions, from which it samples a synthetic dataset. **b**, The synthetic dataset is used to pre-train a Deflexformer block, a Transformer-like neural network. **c**, The multiple copies of the pre-trained block are cascaded to build Deflexformer, which is then trained to learn latent formulas on the observation dataset in the target complex system. **d**, The trained Deflexformer is fed with the observation dataset and some random data for inference, which generates a large number of data representations output by all blocks. **e**, The representation dataset is finally fit by Deflexpressor to obtain the desired formulas through iterative generation and mutation, producing both mathematical expressions and executable lambda calculus code.

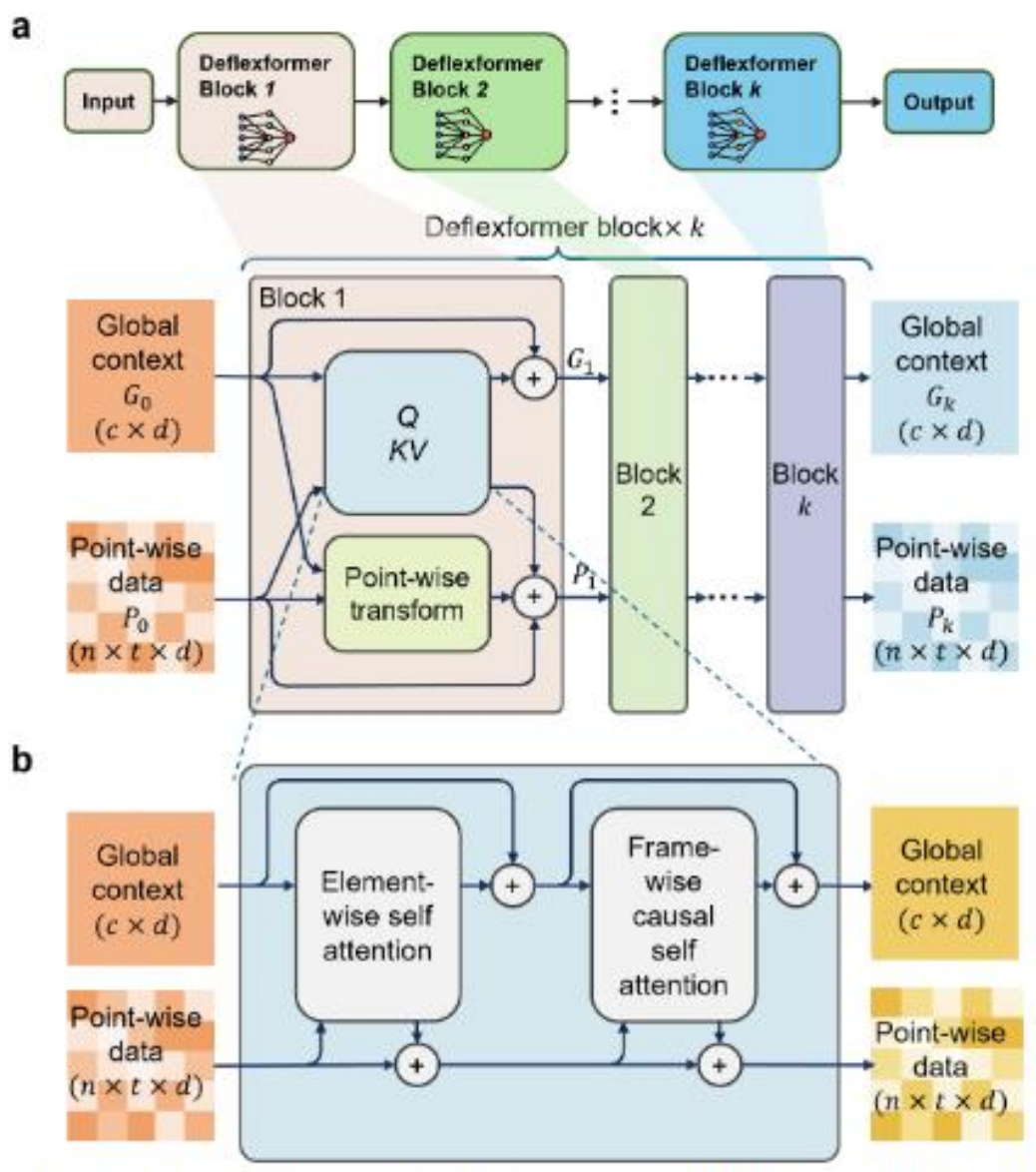

Fig. 2 Illustration of the neural network architecture of Deflex. **a** illustrates the overall model architecture of Deflexformer. Arrows show the information flow among the various components. The intermediate expressions are separated into element-wise and global variables and are processed by different neural networks. **b** illustrates the self-attention network for a Deflexformer block. The point-wise data consists of t time frames, each containing n elements with d dimensions. The point-wise data is first processed by the element-wise residual self-attention with the fusion of global variables and then processed by the frame-wise residual causal self-attention with the fusion of global variables.

Comment 4.10:

Do you pretrain a single deflexformer block or many different ones?

Response to Comment 4.10:

A single Deflexformer block is pre-trained. This single pre-trained block is copied multiple times and cascaded together with an output layer at the end to form the full Deflexformer network. The Deflexformer is then post-trained and the blocks will be fine-tuned differently. To respond this comment, we have clarified this in Sec. 4.4.2 Pre-training the Deflexformer block in the revised manuscript.

Revised Manuscript for Comment 4.10:

4.4.2 Pre-training the Deflexformer block

Directly training Deflexformer $\mathcal{E}(\cdot)$ from scratch requires a vast amount of observation data. Yet, its blocks $\mathcal{E}^i(\cdot)$ are normally simple functions, which have a much smaller search space. To this end, we adopt a common two-stage training framework of pre-training and post-training in other transformer-based deep networks. In particular, a single Deflexformer block is first pre-trained with a large amount of synthetic data based on some specified relationships. Each Deflexformer block $\mathcal{E}^i(\cdot)$ represents a basic mathematical transformation, similar to some base functions (e.g., $\sin(\cdot)$) with a simple expression form. Therefore, we can pre-train the Deflexformer block on a synthetic dataset sampled from some known base functions. Specifically, we first call Deflexpressor to generate a pool of expressions for functions $f(\cdot)$. Then, based on these functions, we can sample numerous data pairs (x, y) to synthesize a pre-training dataset $\mathcal{D}$, where x is drawn from a Gaussian distribution and $y = f(x)$. Here, pre-training aims to fit a block model $\mathcal{E}'(\cdot)$ via supervised learning on the synthetic dataset. So the loss function is defined simply as the MSE between the predictions with labels, given the features of data pairs in the synthetic dataset. That is, $L_{\mathcal{E}'} = \sum_{i=1}^{|\mathcal{D}|} \mathrm{MSE}(y, \mathcal{E}'(x))$ (see SI for training details).

4.4.3 Post-training the full Deflexformer

Then, the full Deflexformer, composed of multiple copies of the pre-trained block and an output module, is post-trained on the observation data. Note that Deflexformer $\mathcal{E}_{\boldsymbol{\theta}}(\cdot)$ is an energy-based model for approximating the optimal energy function $E(\cdot)$. So, its PDF $Q_{\boldsymbol{\theta}}(\cdot)$ should fit $p_{\mathrm{data}}(\cdot)$ with a maximized likelihood $\mathcal{L}(\mathcal{E}_{\boldsymbol{\theta}}, \mathbf{X})$. Here, our likelihood function adopts the common expected log-likelihood over the data distribution

Comment 4.11:

How close are the deflexformer blocks to the original equations they are pretrained on? This is an important question because it deter- mines if the deflexformer blocks are actually beneficial. It seems like they are unnecessary if they are not learning new functions.

Response to Comment 4.11:

We thank the reviewer for this comment. The pre-trained Deflexformer block with extensive synthetic equations is beneficial in learning the prior and common knowledge of equations, thus reducing the required amount of samples for post-training the whole Deflexformer. The cascaded pre-trained blocks can learn new functions in the post-training over the observation data. According to our experiments, they are necessary for the formula discovery. Detailed explanations are as follows.

First, there would be a discrepancy between the pre-trained block and any specific one of the original equations because these equations are randomly synthesized with different structures and parameters. However, with a vast amount of synthesized equation samples, the pre-trained block will learn and extract some higher-level structural common knowledge across the diverse equations.

Second, the pre-trained block carries the prior knowledge of many diverse equations. This prior knowledge has at least two benefits. One is to significantly reduce the sample complexity of post-training on the observation data, which is often scarce in reality. The other is the prior knowledge introduces some inductive bias to lead the post-training towards some semantically common and concise function forms.

In our experiments, we have validated that, with the increasing number of synthesized equations, pre-training can effectively reduce the discovery time in symbolic regression and enhance the accuracy of discovered formulas. In particular, without of pre-training or pre-training with less than certain number of synthesized equations, almost no formula can be discovered and the Deflexformer can hardly fit with the observation data.

Revised Manuscript for Comment 4.11:

2.8 Ablation Analysis

To validate the contribution of each component in Deflex, we conducted systematic ablation studies across multiple dimensions (Fig. 5). We first examined the impact of pre-training on discovery efficiency by varying the synthetic dataset size from 0 to 10^6 formulas. Results demonstrate that larger pre-training volumes lead to substantially faster convergence and higher formula recovery rates (Fig. 5a, d). Pre-training with 10^6 examples achieved over 80% recovery rate within 5,000 steps, while training from

scratch required more than 20,000 steps to reach similar performance, confirming that incorporating mathematical priors through pre-training is crucial for efficient exploration of the formula space.

Architectural ablations reveal that both network depth and embedding dimensions critically affect the model's capacity to capture complex system dynamics. Increasing the number of Deflexformer blocks from 1 to 8 progressively improves performance, with NLL loss decreasing from approximately 1.8 to 0.6 (Fig. 5b, e). Similarly, expanding embedding dimensions from 32 to 128 enhances the model's representational power (Fig. 5c, f). However, performance saturates beyond 6 blocks and 128 dimensions, indicating that these configurations provide sufficient capacity for the tested systems. Moreover, the Deflexformer effectively internalizes physical constraints, autonomously learning energy and momentum conservation manifolds (Fig. 5h). Finally, comparing data sampling strategies demonstrates that Langevin sampling is essential for accurately identifying complex equilibrium distributions (Fig. 5g).

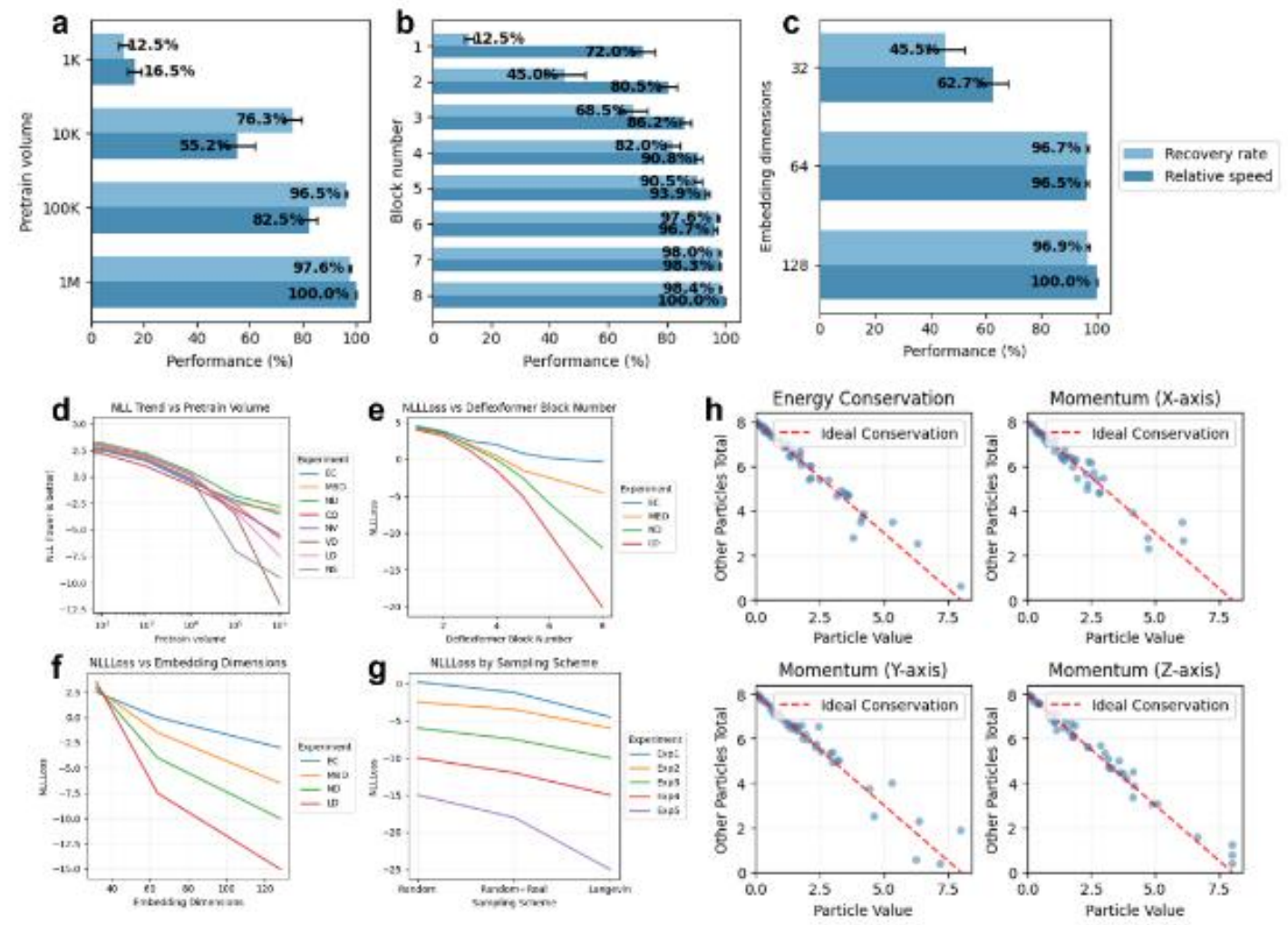

Fig. 4 Ablation studies and component analysis of Deflex. a, d, Impact of pre-training volume on formula discovery performance (recovery rate and relative speed) and NLL trend. Larger pre-training volumes significantly accelerate discovery and improve accuracy. **b, e,** Performance metrics and NLL loss with varying Deflexformer block numbers. Increasing depth improves representation power. **c, f,** Effect of embedding dimensions on performance. **g,** NLL loss comparison across different sampling schemes (Random, Random+Real, Langevin). Langevin sampling yields the best fitness. **h,** Verification of conservation laws (Energy and Momentum) learned by the Deflexformer, showing close alignment between sampled states (blue dots) and ideal laws (red lines).

Comment 4.12:

In Fig. 2, it is not clear where the self-attention network (2b) lies in (2a). Furthermore, it is hard to decipher what is happening in the grey boxes (e.g., element-wise self attention) without showing more details of the equations.

Response to Comment 4.12:

We thank the reviewer for the comment. The self-attention network lies in the QKV module in Fig. 2a of the original manuscript. To make this connection more explicit, we have revised the illustration in Figure 2 for better clarity. Furthermore, we have now provided a more rigorous mathematical formulation of our neural network architecture in the revised Methods section, which includes a formal description of the self-attention mechanism. To respond this comment, we have revised both the manuscript and SI to include more details of the network architecture.

Revised Manuscript for Comment 4.12:

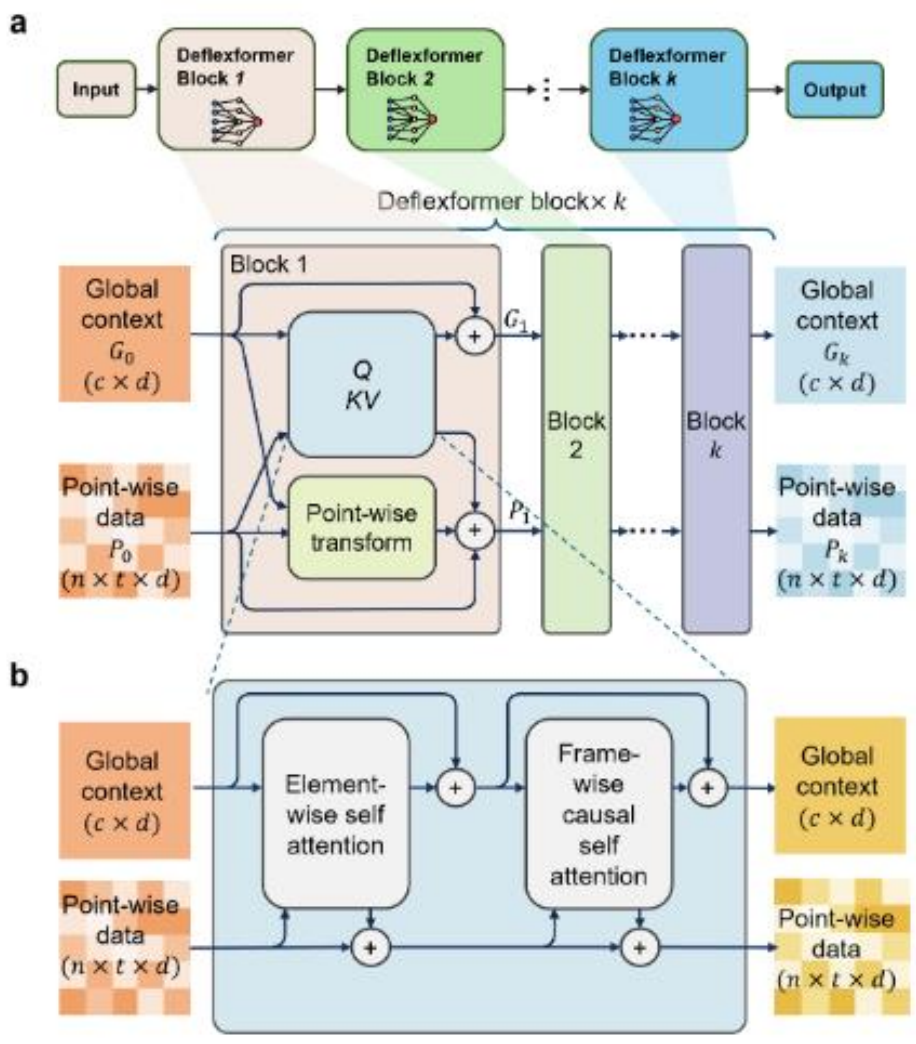

Revised SI for Comment 4.12:

at the base frequency ω_0, providing a coarse estimate of the underlying scalar. Second, higher-frequency harmonics refine this estimate through iterative gradient corrections. For each scale $s > 0$, the reconstruction error in the sine and cosine channels is computed, and a gradient-based update is applied with step size $\eta_s = 2^{-s}$. The diminishing step sizes ensure stability as higher harmonics contribute finer details. This hierarchical refinement mirrors the multiscale nature of the forward embedding, linking the learned latent representation back to the scalar energy values required by the symbolic regression module. The inversion is approximate but sufficient for guiding the evolutionary search, as the primary role is to provide relative orderings rather than exact reconstructions.

Algorithm 3 Deflexformer Block Map

Input: Element state $P \in \mathbb{R}^{n \times t \times d_\ell}$, Global state $G \in \mathbb{R}^{c \times d_\ell}$
Output: Updated states $P \in \mathbb{R}^{n \times t \times d_\ell}, G \in \mathbb{R}^{c \times d_\ell}$

▷ 1. Spatial Mixing (Dimension: n)
1: $H \leftarrow G_{\text{expand}} || P \in \mathbb{R}^{t \times (c+n) \times d_\ell}$ ▷ Concatenation along n
2: $H \leftarrow \text{LayerNorm}(H + \text{MHA}(H))$ ▷ Self-attention over $c + n$
3: $G', P \leftarrow \text{Split}(H)$ ▷ Separate global/element states
4: $G \leftarrow \text{Mean}(G', \text{dim} = t)$ ▷ Aggregate temporal updates
▷ 2. Temporal Mixing (Dimension: t)
5: $H \leftarrow G_{\text{expand}} || P \in \mathbb{R}^{n \times (c+t) \times d_\ell}$ ▷ Concatenation along t
6: $H \leftarrow \text{LayerNorm}(H + \text{MHA}(H, \text{mask}))$ ▷ Mask: G visible to all; P causal
7: $G', P \leftarrow \text{Split}(H)$
8: $G \leftarrow \text{Mean}(G', \text{dim} = n)$ ▷ Fuse spatial updates
9: **return** P, G

Algorithm 3 formally defines the Deflexformer block transformation, mapping input tensors $P \in \mathbb{R}^{n \times t \times d_\ell}$ and $G \in \mathbb{R}^{c \times d_\ell}$ to updated states. The process consists of two decoupled attention mechanisms. First, spatial mixing concatenates G (broadcast along t) and P along the element dimension n, yielding $H^{(s)} = G_{\text{expand}} || P \in \mathbb{R}^{t \times (c+n) \times d_\ell}$. Multi-head attention with $h = 8$ heads is performed over the $(c+n)$ axis, enabling dense cross-element interactions, and the global partition is aggregated via mean pooling over t. Second, temporal mixing concatenates the updated

G (broadcast along n) and P along time dimension t, forming $H^{(t)} = G_{\text{expand}} || P \in \mathbb{R}^{n \times (c+t) \times d_\ell}$. Multi-head attention is applied with a causal mask $M^{(t)}$ where $M_{ij}^{(t)} = 0$ if $j \le c$ or $c < j \le i$, otherwise $-\infty$, ensuring global tokens attend to the entire sequence while element tokens attend only to their causal history. Each block also includes a point-wise transformation network implemented as a four-layer fully connected network with architecture $(d_\ell, 2d_\ell, 2d_\ell, d_\ell)$, ReLU activations, and layer normalization.

The multi-head attention mechanism is computed as:

$$\text{MHA}(Q, K, V, M) = \text{Concat}(\text{head}_1, \ldots, \text{head}_h)W^O, \tag{13}$$

where each head applies scaled dot-product attention:

$$\text{head}_i = \text{softmax}\left(\frac{Q_i K_i^T}{\sqrt{d_k}} + M\right) V_i, \tag{14}$$

with $d_k = d_\ell / h$. For spatial mixing, $H^{(s)} \leftarrow \text{LayerNorm}(H^{(s)} + \text{MHA}(H^{(s)}, H^{(s)}, H^{(s)}, M^{(s)}))$ where $M^{(s)} = 0$. For temporal mixing, $H^{(t)} \leftarrow \text{LayerNorm}(H^{(t)} + \text{MHA}(H^{(t)}, H^{(t)}, H^{(t)}, M^{(t)}))$ where:

$$M_{ij}^{(t)} = \begin{cases} 0 & \text{if } j \le c \text{ or } (j > c \text{ and } j \le i), \\ -\infty & \text{otherwise.} \end{cases} \tag{15}$$

The default configuration uses $d_\ell = D_{model} = 64$, $c = 8$, $h = 8$, $S = 4$, and $k = 4$ to 8 blocks. For particle energy $E = \frac{1}{2} m_i \mathbf{v}_i^2 + U(\mathbf{x}_i)$, each block captures elementary transformations via the decomposable architecture.

Comment 4.13:

Why is the global context needed, and what is the purpose of adding random noise for this context?

Response to Comment 4.13:

Thanks for this question. The global context is a necessary representation in the Deflexformer but is not necessary to be included in the observation data. Adding random noise here referes to its random initialization in the input layer. Our detailed explanations are as follows.

The global context is a specific tensor in our Deflexformer network, and works for representing the global features or variable (e.g., system-wide temperature) from a macroscopic level. Note that, point-wise data directly represents the state information of all the system components/elements from a microscopic level. In our network architecture, it is essential for learning global or system-wide relationships, which often reflect some aggregation information of system elements, e.g., the energy function of the probabilistic distribution that characterizing the elements' states (e.g., the relative positions and velocities of particles) in a complex system.

In particular, global context is not necessary to be explicitly included in the observation data. So, it is initialized as random noise for the input of the first Deflexformer block, if no such global information can be naturally distinguished (by observation) from the system element information in the observation data. However, when such global information can be precisely distinguished (e.g., system temperature value) in the observation data, embedding it and inputting into the global context tensor can make the computation of attention networks much more efficient and accurately. This is because that the element data must perform complex element-wise attentions while such

global information is mainly associated with more efficient aggregation operations. Therefore, taking the global information together with the element data as the input, would lead to expanded network dimensions for the hidden layers and greater computation costs.

To respond this comment, we have rewritten both the problem formulation and the network architecture of Deflex in the revised manuscript.

Revised Manuscript for Comment 4.13:

4.1 Problem formulation

We consider describing a complex system by a set of measurable features $\mathbf{X} = (x_1, \ldots, x_D)$ over time t, where $\mathbf{X}$ denotes the feature vector. They are normally state variables describe the local, dynamic condition of individual components (or elements) within the system at a specific time (e.g., a particle's position and velocity), and optionally some global features characterize the system from a macroscopic level (e.g., a system-wide temperature). Suppose we can obtain an observation dataset

with the features $\mathbf{X}(t)$ of N elements within a time period of T from the system. Specifically, we consider the dimensionality of $\mathbf{X}(t)$ to be D. This dataset can be denoted as $\mathbf{X} \in \mathbb{R}^{NTD}$. The observed data of the state variables can be represented as $\mathbf{X} = \{\mathbf{x}_1, \ldots, \mathbf{x}_N\}$, where $\mathbf{x}_i \in \mathbb{R}^{TD}$ characterizes element i's dynamics from time $t = 1$ to $t = T$. Note that, except for the category of global or elementary features, we have no prior knowledge about the relationships among the features $(x_1, \ldots, x_D)$.

4.4.1 Architecture of Deflexformer

The Deflexformer network predicts the energy value for a given dataset using the aligned state sequences of elements and global context as inputs. The architecture comprises an input embedding layer, k stacked Deflexformer blocks, and an output projection module.

The input layer embeds raw observations X into a high-dimensional feature space via Fourier multiscale embedding (see SI), producing element-level representations $P_0 \in \mathbb{R}^{n \times t \times D_{model}}$ and global representations $G_0 \in \mathbb{R}^{c \times D_{model}}$, where n is the number of elements, t is the number of time frames, c is the number of global tokens, and $D_{model} = 64$ is the model dimension.

Each Deflexformer block $\mathcal{E}^i(\cdot)$ (Fig. 2a) transforms representations (P_{i-1}, G_{i-1}) into (P_i, G_i), approximating a base mathematical transformation $E^i(\cdot)$ in the energy decomposition $E(\cdot) = E^1 \circ E^2 \circ \ldots \circ E^k(\cdot)$. Each block comprises two sub-modules operating on the concatenated input of dimension $(c + nt) \times D_{model}$: (1) A point-wise transformation network implemented as a four-layer fully connected network with architecture $(D_{model}, 2D_{model}, 2D_{model}, D_{model})$ and ReLU activations, modeling element-wise evolution (e.g., the dynamics of an individual particle). (2) A multi-head cross-attention network with $h = 8$ heads for capturing the interactions among particles. The attention operates in two sequential stages (Fig. 2b): spatial mixing performs attention over the element dimension to capture inter-element interactions, while temporal mixing applies causal attention over the time dimension to model temporal dependencies. By factorizing attention into spatial and temporal axes, computational complexity is reduced from $O(nt)$ to $O(n + t)$. Residual connections and layer normalization are applied after each sub-module (see SI for detailed specifications).

The output module projects the final global representation G_k back to a scalar energy via inverse Fourier embedding $e = \mathcal{E}^{-1}(G_k)$ (see SI). For example, when modeling particle energy $E = \frac{1}{2} m_i \mathbf{v}_i^2 + U(\mathbf{x}_i)$, early blocks may capture kinetic energy contributions while later blocks model potential interactions, with their composition yielding the total energy.

Comment 4.14:

In 4.2, f is shown as taking one argument x, which makes sense to me. How in 4.5 is f able to take two arguments h_i and h_j ? The paragraph discussing how to find the Pareto-optimal set with the Reduce process is difficult to follow. It would be helpful to add more or rephrase this section.

Response to Comment 4.14:

We thank the reviewer for the comment. The reviewer is correct: the symbol f was used to refer to the same underlying relationship but in two different contexts in Sections 4.2 and 4.5.

In Sec. 4.2 of the original manuscript, we described the overall relationship as a function that operates on the entire data vector, which we denoted x. In Sec. 4.5 of the original manuscript, when detailing the learning process through our recursive lambda calculus framework, we described it as a recursive function that acts on individual elements h_i and h_j.

We agree that using the same symbol for both the high-level function and its recursive, element-wise definition was confusing. In the revised manuscript, we have now assigned distinct symbols to these two formulations to ensure clarity and improve readability. Please note that, to make our methodology clearer, we have rewritten the methods part in the revised manuscript.

To respond this comment, we have rewritten the corresponding paragraphs and clarified this process with clearer notations and formulations in the revised manuscript.

Revised Manuscript for Comment 4.14:

4.2.4 Initialization with global aggregation priors

While Deflexpressor can generate expressions of any computable functions via the Lambda calculus, standard random initialization tends to produce expression trees that operate on a fixed number of arguments. In such a case, it would be combinatorially expensive to evolve a structure that iteratively computes over a variable-length input list $\mathbf{H} = [\mathbf{h}_1, \ldots, \mathbf{h}_N]$ with N components from scratch, such as calculating the total energy by summing over the energy of N particles. This is because it requires the spontaneous emergence of recursive abstractions.

To accelerate this process without imposing hard constraints, we employ a heuristic initialization strategy that generates the seed expressions in a "Reduce-like" structure. Specifically, these seed expressions are written in the form of a Lambda abstraction $\lambda\mathbf{H}.\text{Reduce}(\text{kernel}, \mathbf{H})$. It means to apply a learnable binary operator, $\text{kernel}(\mathbf{s}, \mathbf{h})$, to sequentially update an accumulated state $\mathbf{s}$ by recursively taking each element $\mathbf{h}_i$ of $\mathbf{H}$ as the input. The recursive expression can be written as

$$\text{reduced}_{\ i} = \begin{cases} h_1, i = 1 \\ \text{kernel} \ (\text{reduced}_{\ i-1}, h_i), i > 1 \end{cases} \tag{5}$$

In particular, the kernel is a randomly generated expression tree that defines the interaction logic between the accumulated state $\mathbf{s}$ and the current data element $\mathbf{h}_i$. This initialization serves as a soft inductive bias. By providing a pre-formed loop mechanism, we allow the symbolic regression to focus immediately on discovering the local interaction laws (encoded in the kernel) that fit the data. Meanwhile, since these structures are standard Lambda terms, they are fully mutable. Subsequent evolutionary steps (e.g., subtree mutation, β-reduction) can alter the kernel, break the recursion, or discard the "Reduce-like" structure entirely if a simpler non-iterative law fits the data well. This provides an initial computational skeleton capable of traversing the entire dataset, which enables the evolutionary algorithm to optimize, modify, or dismantle as needed.

Comment 4.15:

Are the equations presented in Table 1 the only equations discovered from Deflex? Or were additional equations discovered that were not presented

Response to Comment 4.15:

Many thanks for this comment. Not only these equations. The formulas presented in Table 1 were just a highlighted selection of the full, which have clear physical meaning and comparable to the baseline methods. Omitting other discovered equations was due to the page limit of main manuscript.

To improve the rigor and transparency of our work, we have provided the complete list of all unique, non-duplicate formulas discovered by Deflex in the revised SI for reference.

Revised SI for Comment 4.15:

Supplementary Table 8: Full forms of discovered formulas by all compared methods

Law Name	Methods	Formula Form
Energy conservation	Deflex	E = 0.992 * mass * (vel_x^2 + vel_y^2 + vel_z^2) / (k_B * temperature)
	Operon	E = mass * (vel_x^2 + vel_y^2 + vel_z^2) / (k_B * temperature)
	SciMED	E = mass * (vel_x^2 + vel_y^2 + vel_z^2) / (k_B * temperature)
	SINDy	E = mass * (vel_x^2 + vel_y^2 + vel_z^2) / (k_B * temperature)
	gplearn	E = mass * (vel_x^2 + vel_y^2 + vel_z^2) / (k_B * temperature)
	AI Feynman	E = mass * (vel_x^2 + vel_y^2 + vel_z^2) / (k_B * temperature)
	PySR	E = mass * (vel_x^2 + vel_y^2 + vel_z^2) / (k_B * temperature)
	DEAP	E = mass * (vel_x^2 + vel_y^2 + vel_z^2) / (k_B * temperature)
Momentum conservation	Deflex	E = (sum(mass * vel_x) − C_x)^2 + (sum(mass * vel_y) − C_y)^2 + (sum(mass * vel_z) − C_z)^2
	Operon	sum(mass(i) * vel_x(i)) = C_x, sum(mass(i) * vel_y(i)) = C_y, sum(mass(i) * vel_z(i)) = C_z
	SciMED	sum(mass(i) * vel_x(i)) = C_x, sum(mass(i) * vel_y(i)) = C_y, sum(mass(i) * vel_z(i)) = C_z
	SINDy	sum(mass(i) * vel_x(i)) = C_x, sum(mass(i) * vel_y(i)) = C_y, sum(mass(i) * vel_z(i)) = C_z
	gplearn	sum(mass(i) * vel_x(i)) = C_x, sum(mass(i) * vel_y(i)) = C_y, sum(mass(i) * vel_z(i)) = C_z
	AI Feynman	sum(mass(i) * vel_x(i)) = C_x, sum(mass(i) * vel_y(i)) = C_y, sum(mass(i) * vel_z(i)) = C_z
	PySR	sum(mass(i) * vel_x(i)) = C_x, sum(mass(i) * vel_y(i)) = C_y, sum(mass(i) * vel_z(i)) = C_z
	DEAP	sum(mass(i) * vel_x(i)) = C_x, sum(mass(i) * vel_y(i)) = C_y, sum(mass(i) * vel_z(i)) = C_z
Maxwell-Boltzmann (MBD)	Deflex	E = 0.511 * mass * (vel_x^2 + vel_y^2 + vel_z^2) / (k_B * temperature)
	Operon	P ~ exp(−mass * (vel_x^2 + vel_y^2 + vel_z^2) / (2 * k_B * temperature))
	SciMED	P ~ exp(−mass * (vel_x^2 + vel_y^2 + vel_z^2) / (2 * k_B * temperature))
	gplearn	P ~ exp(−mass * (vel_x^2 + vel_y^2 + vel_z^2) / (2 * k_B * temperature))
	AI Feynman	P ~ exp(−mass * (vel_x^2 + vel_y^2 + vel_z^2) / (2 * k_B * temperature))
	PySR	P ~ exp(−mass * (vel_x^2 + vel_y^2 + vel_z^2) / (2 * k_B * temperature))
	DEAP	P ~ exp(−mass * (vel_x^2 + vel_y^2 + vel_z^2) / (2 * k_B * temperature))
MBD with potential	Deflex	E = 0.506 * mass * (vel_x^2 + vel_y^2 + vel_z^2) / (k_B * temperature) + sum(0.97 * ((pos_x(i)−pos_x(j))^2+(pos_y(i)−pos_ − 1.89 * ((pos_x(i)−pos_x(j))^2+(pos_y(i)−pos_y(j)
Continuity of quality	Deflex	E = (vel_x_dx + vel_y_dy)^2 * 10^26
	Operon	vel_x_dx + vel_y_dy = 0
	SciMED	vel_x_dx + vel_y_dy = 0
	SINDy	vel_x_dx + vel_y_dy = 0
	gplearn	vel_x_dx + vel_y_dy = 0
	AI Feynman	vel_x_dx + vel_y_dy = 0
	PySR	vel_x_dx + vel_y_dy = 0
	DEAP	vel_x_dx + vel_y_dy = 0

Law Name	Methods	Formula Form
Newton viscosity law	Deflex	τ_xy = −1.037 * μ * (vel_x_dy + vel_y_dx)
	Operon	τ_xy = −μ * (vel_x_dy + vel_y_dx)
	SciMED	τ_xy = −μ * (vel_x_dy + vel_y_dx)
	SINDy	τ_xy = −μ * (vel_x_dy + vel_y_dx)
	gplearn	τ_xy = −μ * (vel_x_dy + vel_y_dx)
	AI Feynman	τ_xy = −μ * (vel_x_dy + vel_y_dx)
	PySR	τ_xy = −μ * (vel_x_dy + vel_y_dx)
	DEAP	τ_xy = −μ * (vel_x_dy + vel_y_dx)
Velocity distribution	Deflex	E = 0.501 * mass * (vel_x^2 + vel_y^2) + (δv)^3.15
	AI Feynman	P ~ exp(−(0.5 * mass * (vel_x^2 + vel_y^2) + (δv)^p))
Navier-Stokes equation (2D Cylinder)	Deflex	E = (ρ * (vel_x_dt + vel_x * vel_x_dx + vel_y * vel_x_dy) + pressure_dx − μ * (vel_x_dx_dx + vel_x_dy_dy))^2 * 1.62e21 + (ρ * (vel_y_dt + vel_x * vel_y_dx + vel_y * vel_y_dy) + pressure_dy − μ * (vel_y_dx_dx + vel_y_dy_dy))^2 * 1.62e21
	Operon	ρ * (vel_x_dt + vel_x * vel_x_dx + vel_y * vel_x_dy) + pressure_dx − μ * (vel_x_dx_dx + vel_x_dy_dy) = 0, ρ * (vel_y_dt + vel_x * vel_y_dx + vel_y * vel_y_dy) + pressure_dy − μ * (vel_y_dx_dx + vel_y_dy_dy) = 0

Law Name	Methods	Formula Form
Navier-Stokes equation (3D JHTDB)	Deflex	E = (ρ * (vel_x_dt + vel_x * vel_x_dx + vel_y * vel_x_dy + vel_z * vel_x_dz) + pressure_dx − μ * (vel_x_dx_dx + vel_x_dy_dy + vel_x_dz_dz))^2 * 3.27e21 + (ρ * (vel_y_dt + vel_x * vel_y_dx + vel_y * vel_y_dy + vel_z * vel_y_dz) + pressure_dy − μ * (vel_y_dx_dx + vel_y_dy_dy + vel_y_dz_dz))^2 * 3.27e21 + (ρ * (vel_z_dt + vel_x * vel_z_dx + vel_y * vel_z_dy + vel_z * vel_z_dz) + pressure_dz − μ * (vel_z_dx_dx + vel_z_dy_dy + vel_z_dz_dz))^2 * 3.27e21
Langevin	Deflex	E = (4.438e16 * mass * vel_x_dt + 1.003 * vel_x)^2 + (4.438e16 * mass * vel_y_dt + 1.003 * vel_y)^2 + (4.438e16 * mass * vel_z_dt + 1.003 * vel_z)^2
	Operon	4.468e16 * mass * vel_x_dt + vel_x = 0, 4.468e16 * mass * vel_y_dt + vel_y = 0, 4.468e16 * mass * vel_z_dt + vel_z = 0
Levy flight (Human)	Deflex	E = 2.37 * log(sqrt((lat(time) − lat(time−1))^2 + (lon(time) − lon(time−1))^2))
	AI Feynman	P ~ u^−2.37
	PySR	P ~ u^−2.37
	DEAP	P ~ u^−2.37
Levy flight (Avian)	Deflex	E = 1.627 * log(sqrt((lat(time) − lat(time−1))^2 + (lon(time) − lon(time−1))^2))
	AI Feynman	P ~ u^−1.627
	PySR	P ~ u^−1.627
	DEAP	P ~ u^−1.627
Power law	Deflex	E = 1.56 * log(l) + 0.00027 * l
	AI Feynman	P ~ l^−1.56 * exp(−0.00027 * l)

Law Name	Methods	Formula Form
Crowd power law	Deflex	E = 1.63 * log(r(i)) − 0.037 * log(r(i)) * sum(I(sqrt((lat(j)−lat(i))^2 + (lon(j)−lon(i))^2)<2.46))

Comment 4.16:

On pg 3, DNN is used before it is defined

Response to Comment 4.16:

Thanks. We have added its full term. We have proofread the manuscript and clarified the full terms of all abbreviations at their first appearance.

Revised Manuscript for Comment 4.16:

> With these unified representations and formulations, we next develop Deflexformer, a deep neural network (DNN) to learn the energy functions. Different from existing neural-integrated methods, Deflexformer can further decompose the learned functions into elementary components that are tractable for symbolic regression. It is

Comment 4.17:

Figure 1 and the comments like "...mimicking the process scientists use to break down intricate systems to make the approach efficient for highly complex formulas" seem very out of place. These connections are loose at best.

Response to Comment 4.17:

Many thanks for this comment. We agree that Figure 1 and the comments are too conceptual when presented before the main results and detailed methods. Therefore, we have moved to Figure 1 in the original manuscript to SI. The comments have also been removed in the Introduction section.

Comment 4.18:

On pg 6, there is a typo "Here,Since".

Response to Comment 4.18:

Thanks. This has been corrected in the revised manuscript.

Comment 4.19:

It would be useful to show examples of some of the probabilities used to compute the EMD in Fig. 4c. Adding further clarification here would also be helpful. I do not understand how you are getting the different scales here, especially for cases like the NS.

Response to Comment 4.19:

We thank the reviewer for this comment. First, we would like to clarify how we define and analyze different scales: our approach is based on a coarse-graining procedure. We first generate data at multiple scales. For the particle system, this is done by aggregating spatially adjacent particles into larger effective entities. For the fluid system, we similarly combine neighboring grid cells into larger blocks. The macroscopic properties of these new entities are then computed.

Then let's clarify our use of the EMD with an example from our particle dynamics experiment. The Langevin equation predicts a theoretical distribution of particle trajectories. The observed data from the simulation forms an empirical distribution. The EMD we measured is the average minimum transport distance required to transform the empirical distribution into the theoretical one. Critically, the EMD has the same units as the underlying space—in this case, units of length.

To create a scale-invariant metric for comparison, we report the ratio of the EMD to a characteristic length scale of the system. This normalization is essential for our multi-scale analysis. For instance, in the fluid experiment, if we coarse-grain the system by a factor of 3, the characteristic length scale triples. If the absolute EMD (in meters, for example) remains the same, our reported dimensionless EMD value becomes one-third of its original value. This approach effectively decouples our error metric from absolute physical units, allowing for a meaningful and fair comparison of a formula's accuracy across different scales.

Subsequently, we perform symbolic regression on this coarse-grained data at each scale. To create a valid benchmark for comparison, we take the known ground-truth equation and re-calculate its parameters for each level of coarse-graining—a process known as renormalization. The series of EMD errors we report is then obtained by comparing the predictions of our discovered formulas against the coarse-grained data at each respective scale.

We used EMD (earth moving distance) here since it inherently has the same units as the distance metric of the space in which the distribution is defined. In particular, the EMD between a spatial distribution and a single data point at location x is simply the average distance from the distribution to that point x. Therefore, when we compare the EMD to a characteristic length scale of the system, the resulting ratio is independent of the dimensionality.

Revised SI for Comment 4.19:

1.4.3 Coarse-graining and Renormalization Protocols

To rigorously evaluate the capability of Deflex in discovering multiscale laws, we introduce a renormalization analysis framework. This involves applying specific coarse-graining operators $\mathcal{R}_\lambda$ with a scale factor λ to the system, and examining whether the method can independently identify the renormalized physical laws at each scale. We adopt two distinct coarse-graining protocols tailored to the nature of the simulated systems: re-simulation for particle systems and data aggregation for continuous fields.

For discrete particle systems (specifically the water particle experiment), we performed coarse-graining at the initialization stage and conduct re-simulations. We define a set of scale factors $\lambda \in \{1, 3, 10\}$, representing the ratio of the characteristic length of the coarse-grained particles to the original molecules. At the initial frame, we employ a distance-based clustering approach to group water molecules into macro-particles. Assuming the characteristic distance between molecules is d_0, the clustering threshold is set to λd_0, which scales linearly with the coarse-graining factor. Molecules within this threshold distance are grouped into a single macro-particle. The physical properties of these macro-particles are calculated to serve as the initial conditions for a new run of simulations. Specifically, for a macro-particle I consisting of a set of molecules S_I, its mass M_I, position $\mathbf{R}_I$, and velocity $\mathbf{V}_I$ are initialized as:

$$M_I = \sum_{i \in S_I} m_i, \quad \mathbf{R}_I = \frac{1}{M_I} \sum_{i \in S_I} m_i \mathbf{r}_i, \quad \mathbf{V}_I = \frac{1}{M_I} \sum_{i \in S_I} m_i \mathbf{v}_i. \tag{11}$$

where m_i, $\mathbf{r}_i$, and $\mathbf{v}_i$ denote the mass, position, and velocity of individual molecule i, respectively. The system is then evolved using the same interaction potentials but scaled parameters, generating the "ground truth" data for each scale λ.

For continuous fluid dynamics, we applied a standard data coarse-graining approach to the high-resolution simulation outputs. The scale factor λ varies continuously from 1 to 100. We partitioned the computational grid into blocks of size $\lambda \times \lambda$. The coarse-grained fields (e.g.,

velocity $\mathbf{u}'$) are obtained by block averaging the microscopic fields ($\mathbf{u}$):

$$\mathbf{u}'(x,y) = \frac{1}{\lambda^2} \sum_{i,j \in \text{Block}(x,y)} \mathbf{u}(i,j). \tag{12}$$

where $\mathbf{u}'(x,y)$ denotes the coarse-grained velocity field, and $\text{Block}(x,y)$ represents the $\lambda \times \lambda$ spatial block centered at (x,y). This process generates coarse-grained datasets representing the flow at macroscopic scales.

Renormalization Verification Renormalization refers to a common feature or process in many multiscale systems, where a formula describing a system at a fine-grained level can be transformed into one with the same form at a coarse-grained level by just changing the scale-related parameter. Taking the Maxwell-Boltzmann distribution as a concrete example: at the microscopic scale ($\lambda = 1$), the speed distribution of particles with mass m follows $f(v) \propto v^2 \exp(-\frac{mv^2}{2k_BT})$ where v denotes the particle speed, m is the particle mass, k_B is the Boltzmann constant, and T is the temperature. When coarse-grained to scale λ, if the macro-particles (now with mass $M \approx \lambda^3 m$) reach thermal equilibrium, the law formula renormalizes to the same functional form but with a scaled parameter: $f(V) \propto V^2 \exp(-\frac{MV^2}{2k_BT})$.

To validate whether Deflex can recover the renormalization group flow, we compared the distribution predicted by Deflex against two baselines: 1) the distribution of the actual coarse-grained data (Ground Truth), and 2) the theoretical renormalized formulas.

Comment 4.20:

In Eq. 3 are g and f flipped in the inequality for L?

Response to Comment 4.20:

Thank you for this comment. Yes, this was a typo and they should be flipped in the inequality. We have corrected this typo and proofread carefully to minimize other typos in the revised manuscript.

Revised Manuscript for Comment 4.20:

$$\forall \mathbf{g} \subseteq \mathcal{F},\ \sum_{f \in \mathbf{f}^*} \mathcal{L}(f, \mathbf{X}) \leq \sum_{g \in \mathbf{g}} \mathcal{L}(g, \mathbf{X}) \Rightarrow \sum_{f \in \mathbf{f}} R(f) \leq \sum_{g \in \mathbf{g}^*} R(g). \tag{3}$$

Comment 4.21:

Pg. 15 has a typo where you write "global representation P_{k-1}"

Response to Comment 4.21:

Thank you for this comment. It should be "global representation G_{k-1}" and P_{k-1} corresponds to the element-wise representation. This has been corrected and the subsection has been rewritten in the revised manuscript. We have also proofread the manuscript carefully to minimize the typos.

Comment 4.22:

The abbreviation "NLL" is used before it is defined.

Response to Comment 4.22:

Many thanks. We have added its full term. We have proofread the manuscript and clarified the full terms of all abbreviations at their first appearance.

Revised Manuscript for Comment 4.22:

- For distribution-based formulas, we use the negative log-likelihood (NLL) loss defined in Eq. (8) in the method section, which measures the likelihood of the distribution fitting real data. The feasible methods were also set with the same convergence constraint. Note that we also report the RMSE to measure the fitting error of simple distributions for reference.

Response Letter

Manuscript NCOMMS-25-26170B

Dear reviewers,

We thank the editor and reviewers for the helpful and insightful comments, which significantly improve the quality of the article. Considerable modifications have been made in this version (marked as blue in the revised manuscript and supplementary information), which are summarized as follows:

1. **End-to-end Experiments** (Comments 1.1 and 4.2): we have conducted new experiments to validate the end-to-end formula discovery from raw observation data, without requiring derivative features.
2. **Discussions of Extensions** (Comment 1.3): we have discussed how our method and experiments can be extended to different physical systems in the real world.
3. **Discussions of Limitations** (Comments 1.4): we have added discussions on the limitations of our method in formula discovery, and provided possible directions for further exploration.
4. **Description Clarification and Corrections** (Comments 4.4 and 4.8): we have revised all unclear descriptions and confusing writings pointed out by the reviewers.
5. **Details of Experimental Results** (Comments 4.1, 4.3, 4.5, and 4.6): we have re-conducted some experiments to present more details of raw formulas discovered by all methods.
6. **Improved Presentation and Readability** (Comments 4.9 and 4.10): we have made several modifications, such as re-organizing the chapters to improve the presentation and readability of the manuscript.
7. **Other Modifications** (Comments 4.20): we have fixed all typos and other issues pointed out by the reviewers and found by ourselves.

Detailed point-by-point responses are as follows:

=======Reviewer #1=======

Comments to the Authors:

I would start this review by saying I suggested rejecting the paper the entire process - the experiments were poorly presented, the results seemed like cheating, and the model was described like black magic with "trust us" feeling. However, after several hard review rounds, I changed my mind. This time, I have several final comments which I think are important for the fairness of the claims for potential readers following them, I will recommend acceptance.

Response to Reviewer #1:

We thank the reviewer for this positive comment and for all your previous constructive comments and suggestions, which have helped us significantly in improving the quality of our manuscript.

Comment 1.1:

Proving "du/dt" is a fair methodological approach but missing the point of discovering "du/dt" from the raw "u" signal - the same argument you had for comment 1.1. You can argue this is a scientist in a loop here or some feature engineering pre the SR part. Both options are legitimate, but you must make this decision.

Response to Comment 1.1:

We thank the reviewer for this insightful comment. We agree that it is reasonable that there could be scientists in the loop of automatic formula discovery, and it is quite common to include feature engineering for AI-based methods. We also admit that providing the derivative features in the datasets may undermine Deflex's capability of end-to-end formula discovery. The main reason why the derivative features were provided in our experiments is because that they are already included in the publicly available simulation datasets, which are necessary for some traditional SR-based methods like Operon. By incorporating them, we can thus ensure a fair comparison with the state-of-the-art methods.

To validate Deflex's capability for end-to-end formula discovery, we have conducted new experiments by removing the derivative features from the original datasets. New experimental results have shown that Deflex is capable to discover almost all the formulas again without these derivative features, although there will be slightly higher accuracy loss and prolonged run-time due to the implicit learning of these derivative terms.

To respond to this comment, we have made the following modifications in the revised manuscript and SI.

1) We have added the performance comparison results between the datasets with and without derivative features, and provided corresponding analysis in Subsection 7.5 in the revised SI.

Revised SI (Subsection 7.5):

7.5 Ablation study on derivative (gradient) features

In the simulated spatiotemporal systems (e.g., the fluid and particle systems), the governing laws are naturally expressed with temporal derivatives (e.g., $\partial \mathbf{u}/\partial t$) and spatial gradients (e.g.,

$\nabla \mathbf{u}$ and ∇p). In our default feature construction for such datasets (e.g., fluid dynamics; see Supplementary Table 6), these derivative features are treated as part of the observation feature set: they are either directly provided by the simulator/dataset (e.g., JHTDB) or computed deterministically from the raw fields using standard finite-difference schemes (e.g., the cylinder wake setup).

To evaluate Deflex's end-to-end discovery capability from the raw data, we further conducted experiments over the fluid and particle systems, where all the spatial-derivative and temporal-derivative features were removed. In particular, Deflex was set up with the same training protocol, stopping criteria, and time budget as the default setting.

In these experiments, except for the 3D Navier–Stokes equation in the JHTDB setting, all discovered formulas in the previous experiments were recovered. Specifically, the 3D Navier–St equation could not be recovered in a reasonable time without the derivative features. This failure would be due to its substantially higher structural complexity. Once derivative features were removed from the observation dataset, they must be represented implicitly through difference-based expressions, which significantly increases the required expression complexity. Although such representations remain expressible within our operator set, they typically require much deeper expression trees and significantly higher computational cost, which would go beyond the search configuration (including the initial population size and runtime budget) in our experiments.

We further compare their performance in both cases. Supplementary Fig. 8 compares both the accuracy and efficiency of Deflex on the spatial-temporal systems with and without the derivative features. As shown, removing the derivative features caused an accuracy loss in both NLL and RMSE. The reason is that learning these derivatives from the raw data would introduce additional errors, increasing the overall loss. On the other hand, the runtime was prolonged due to an additional search for these derivative features.

Note that we do not compare the performance of the baselines in this ablation study. The reason is that most compared symbolic regression frameworks operate on fixed-dimensional feature vectors and do not natively support expressing operators that traverse spatiotemporal neighborhoods or perform high-order reductions over variable-sized adjacency. In particular, without these derivative features, without additional adaptations (e.g., injecting finite-difference stencil operators and explicit aggregation/traversal routines into their symbol sets, or constraining the expression space to enforce such structures), these baselines could barely learn the formulas with derivative terms.

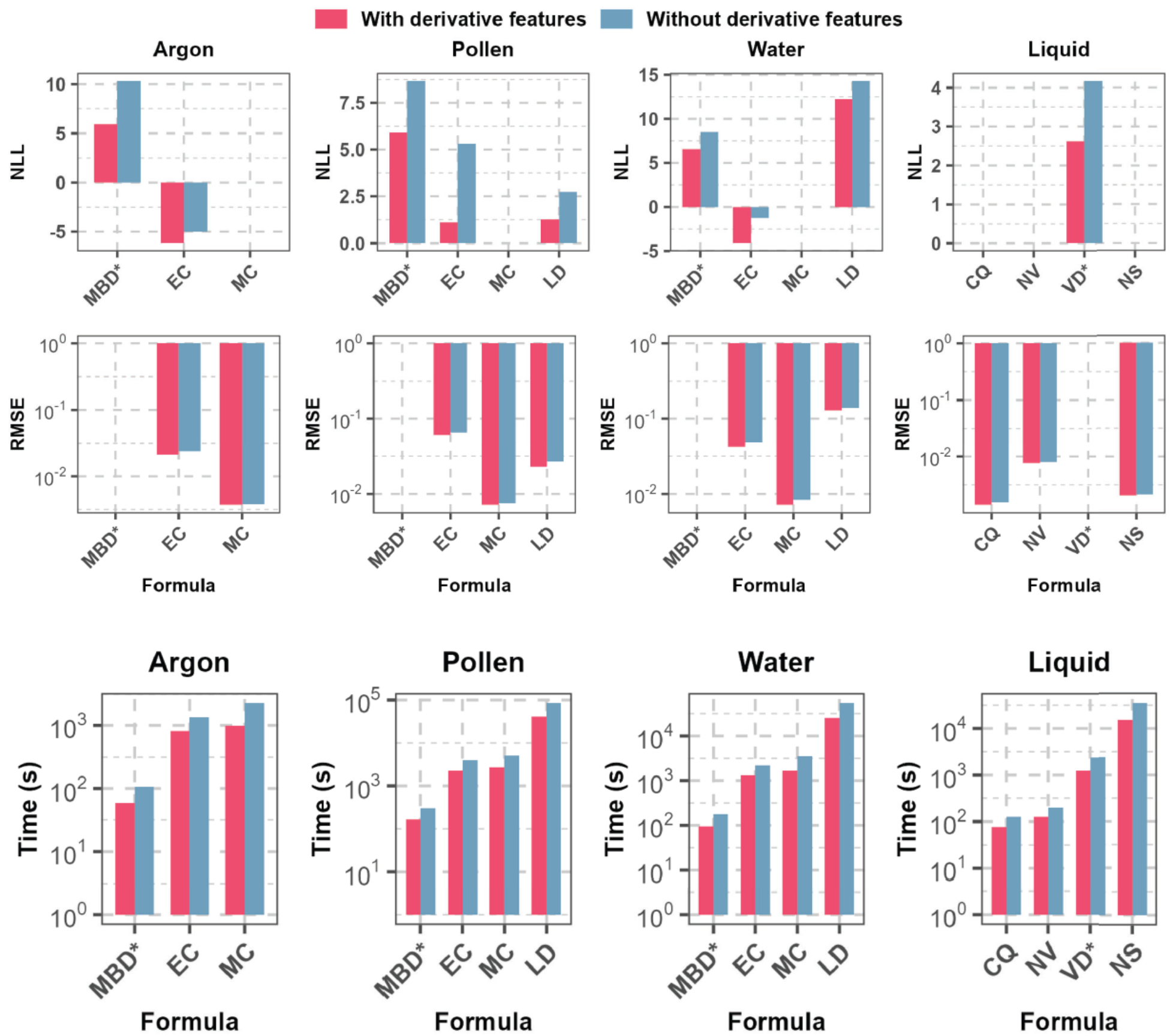

Supplementary Figure 8: Ablation study on derivative (gradient) features. We compare the discovery performance of Deflex on the datasets with and without derivative features. Top row reports NLL for distributional targets, middle row reports RMSE for equation/invariant targets, and bottom row reports runtime. Removing derivative features generally preserves accuracy but reduces efficiency; All formulas were recovered again except for the 3D Navier–Stokes equation (JHTDB), which did not reliably converge within the same time budget and stopping criteria after removing derivative features.

2) We have also summarized the comparison results in Fig. 4 in the revised manuscript.

Revised Manuscript (Subsection 2.8):

cating that these configurations provide sufficient capacity for the tested systems. We also conducted ablation studies on the derivative (gradient) features from the datasets (Fig. 4d), finding that accuracy is mostly preserved but discovery efficiency degrades, and the complicated 3D Navier–Stokes case (JHTDB) does not reliably converge under the same time budget. Moreover, the Deflexformer effectively internalizes physical con-

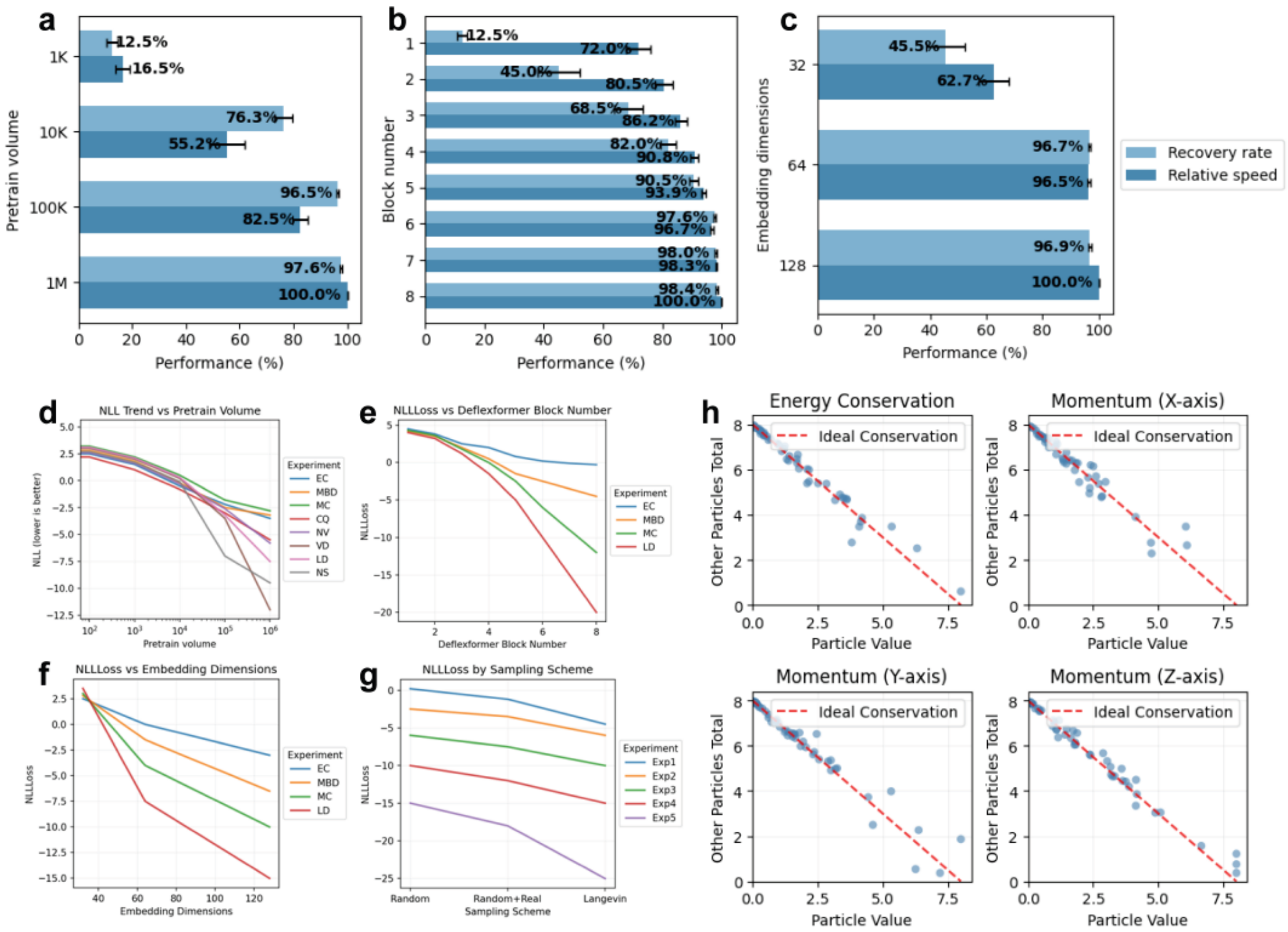

Fig. 4 Ablation studies and component analysis of Deflex. a, **e**, Impact of pre-training volume on formula discovery performance (recovery rate and relative speed) and NLL trend. Larger pre-training volumes significantly accelerate discovery and improve accuracy. **b**, **f**, Performance metrics and NLL loss with varying Deflexformer block numbers. Increasing depth improves representation power. **c**, **g**, Effect of embedding dimensions on performance. **d**, Ablation on derivative (gradient) features, comparing the default feature configuration against a variant where temporal-derivative and spatial-gradient channels are removed from the input. **h**, NLL loss comparison across different sampling schemes (Random, Random+Real, Langevin). Langevin sampling yields the best fitness. **i**, Verification of conservation laws (Energy and Momentum) learned by the Deflexformer, showing close alignment between sampled states (black dots) and ideal laws (red lines).

Comment 1.2:

For SciMED, one of its main capabilities (which is also shared with AI Feynman) is that the user provides feature sets or constraints on the expression tree. I understand this cannot be fairly provided without shifting the results. However, it is worth stating that SciMED is used in its most "raw" version and one can gain better results with more "Scientist-in-the-loop".

Response to Comment 1.2:

We thank the reviewer for this comment. We agree that most of the comparative methods have variants that would gain better performance with the scientists-in-the-loop approach. For instance, manually providing feature sets or constraints on the expression tree for SciMED and AI Feynman, would improve their performance by incorporating prior scientific knowledge such as symmetry or structural constraints.

In particular, we agree that our comparison is based on the raw version of existing methods despite some adaptations for alignment. Therefore, to reflect a fair comparison and objective description, we have added remarks to highlight this in the revised manuscript, which are shown as follows.

Revised Manuscript (Subsection 2.1):

> Note that some of these frameworks have variants that would gain better performance with scientists in the loop (e.g., manually providing feature sets or constraints on the expression tree for SciMED and AI Feynman), while we mainly used the raw version for simplicity despite the above adaptations. A method was considered to have failed a task if it still could not discover the rule after these best-effort accommodations.

Besides, to provide references for interested readers, we have also performed additional experiments under such settings and reported the results in Supplemental Fig.2 in the revised SI. These experiments show that introducing scientist-in-the-loop configurations does not increase the number of formulas recovered by these methods, although it contributes to a noticeable improvement in discovery speed, in the particle system experiments.

Revised SI (Subsection 7.8):

7.8 Impact of scientist-in-the-loop approach on baseline methods

The scientist-in-the-loop approach is proposed [11] in AI-driven formula discovery, which improves the performance of existing methods. However, in the main text, we present the performance of existing methods in their raw form to ensure an aligned experimental setup. To provide complete references for readers, we briefly discuss this approach in this subsection and demonstrate additional evaluation results of the compared methods under this approach.

For SciMED [11], the scientist-in-the-loop approach injects prior knowledge over the space of the candidate formulas. In particular, implausible operators can be removed for a given task. For example, trigonometric operators like $\sin$ can be removed from the systems considered in this work. For variables with physical dimensions, dimensional information can be provided whenever feasible. The complexity can also be preset according to the expected target formula: the maximum tree depth can be set to 5 for relatively simple formulas, whereas it can be increase to 15 for more complex ones. When necessary, some minimal template-level constraints for structures such as summation can be retained for the expected formulas.

For AI Feynman [14], the scientist-in-the-loop approach works by providing dimensional constraints and restricted subsets of candidate operators. In addition, the polynomial-degree cutoff can be adjusted according to the expected complexity of the target formula, e.g., using a lower cutoff for simpler formulas and a higher cutoff for more complex ones. For more challenging formulas, a larger brute-force search budget can be also allowed.

To show a more complete performance of the compared methods, we implemented the above scientist-in-the-loop approach on SciMED and AI Feynman and conducted additional experiments on the particle and fluid tasks. Please note that, here, we mainly considered compact forms of prior knowledge that can be specified in a reasonably consistent manner across tasks. To ensure a rigorous and fair performance assessment, we kept the same overall computational budget and success criterion as in the main comparative setup. In particular, we used the same

time budget of 20, 000 seconds and the same residual-error-based standard for successful recovery.

Supplementary Fig. 10 compares the discovery time of SciMED and AI Feynman with and without the scientist-in-the-loop approach. Please note that, since the experimental setups and convergence conditions are the same for both cases, the discovered formulas and their accuracy also remained unchanged and thus not reported. As shown, there is a noticeable reduction in the discovery time in both SciMED or AI Feynman, by incorporating a limited scientist-in-the-loop setting with relatively generic prior knowledge.

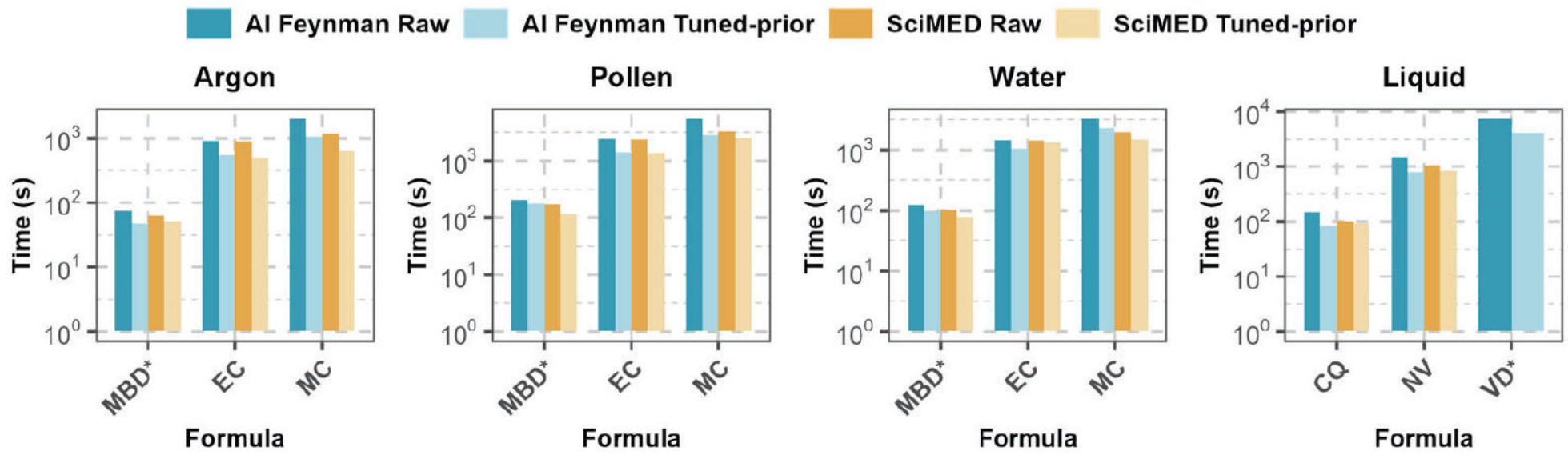

Supplementary Figure 10: Impact of scientist-in-the-loop configurations on discovery time for SciMED/AI Feynman. We compare the wall-clock runtime (log scale) under the default "raw" settings and tuned settings with manually provided feature/structure constraints in the Argon, Pollen, Water, and Fluid experiments.

Comment 1.3:

In the discussion, as one of the main novelties of this study is the time-series capabilities, it would be nice to have a discussion on how such a capability can be used in different physical settings and implementation for experiment design, so practitioners will learn how to generate data for such models.

Response to Comment 1.3:

We thank the reviewer for this constructive suggestion. We agree that, since time-series-like fine-grained state data is the main input of Deflex, it is helpful to clarify how to generate data and design experiments so that practitioners can apply Deflex to different physical systems in practice.

The most typical physical setting of our work is based on time-series and interacting-entities: each training sample is formed by an optional global context, and entity-level trajectories for a subset of n elements observed over T consecutive time steps. Concretely, practitioners can collect a continuous segment of frames, track each element at each time step, and extract per-entity features (at minimum positions; often also velocities when available). Practitioners can then repeatedly construct a set of training samples by sampling spatiotemporal sub trajectories (subsets of entities and adjacent time windows) from the raw dataset, and apply the model to learn an energy-form expression that aggregates the variable-size interactions.

Regarding experiment design, successful symbolic recovery benefits from diverse and sufficiently

large coverage of the system's state space, i.e., multiple trajectories under varied initial conditions. In terms of scale, the number of distinct spatiotemporal samples should be largely proportional to the target expression complexity. According to our experience, this typically means at least on the order of 10^3 samples and often much more (up to10^5–10^6) when the target law is more complex or the noise level is higher. If the available data for collection is limited, the same sampling mechanism over trajectories (multiple windows/subsets per run) can be used to augment effective training samples without changing the underlying physics.

In addition, Deflex is not restricted to time series. Specifically, it can operate in a global context-only mode (no explicit entity set and no time axis), where it reduces to a conventional symbolic-regression setting on tabular inputs while still leveraging the neural decomposition for complex formulas.

Finally, like other scientific discovery, the scientist-in-the-loop approach is very important for effective formula discovery. Especially, for practitioners working in new systems with unfamiliar behaviors, it is necessary to consult or collaborate with the experts in the area.

To respond to this comment, we have added a paragraph in Section 3 "Discussion" of the revised manuscript, which briefly discusses on how to apply Deflex in practical systems in terms of both data generation and experimental implementation, which are shown as follows.

Revised Manuscript (Section 3):

> From a practical application perspective, Deflex requires that the observation of complex systems can be organized into learning samples. In a typical physical setting, each sample combines global context with evolving states of a set of n interacting entities over consecutive time steps. In practice, one can obtain a long continuous record and then construct training samples by extracting spatiotemporal sub-trajectories, allowing the model to learn an energy-form expression that aggregates variable-size interactions. Successful symbolic recovery further demands sufficiently diverse and broad coverage of the system's state space. This can be done by collecting multiple trajectories under varied initial conditions. If data collection is limited, sampling over multiple windows and entity subsets may help to increase the number of training samples without altering the underlying physics. In real applications, the scientist-in-the-loop approach can also be incorporated into data collection and feature engineering when domain knowledge is needed.

Comment 1.4:

Most important - the current table with the main results is showing the method with "V" all the time. Find one example where the model does not work and explain why in the discussion. This is how you drive the field forward, where others have an open question to deal with

Response to Comment 1.4:

We thank the reviewer for this important suggestion. Deflex is showing "V" all the time in Table 2 mainly because we focused on summarizing the formulas that can be discovered by Deflex with clear physical meanings. However, we agree with the reviewer that it is important to find one example where Deflex is not good at and raise an open question for further exploration.

During our experiments, we observed that, compared with most of the baselines, Deflex has a lower discovery rate on the formula of quantum transition probabilities from the AI-Feynman dataset, which describes the likelihood of a system moving between states due to perturbations and has the form of

$$p = \frac{p_d E_f t}{\hbar} \frac{\sin^2\left(\left(\omega - \omega_0\right)t/2\right)}{\left(\left(\omega - \omega_0\right)t/2\right)^2}$$

where $\hbar = h/(2\pi)$, p is the transition probability, p_d, E_f, ω, ω_0 denote the transition dipole moment, the field amplitude, the driving angular frequency, and the transition angular frequency, respectively.

The potential reason for low discovery would be due to the adverse effect of Deflex's multi-scale discovery. That is, Deflex searches for multiple formulas at different scales simultaneously. While formulas with very different scales coexist, strong patterns could dominate and suppress other correct formulas, leading to a lowered discovery rate. Specifically, the formulas of quantum transition probabilities possess a distinct characteristic that their original form is a high-frequency function on certain scales with complex forms but can be approximated by low-frequency functions on other scales with simple forms. In such cases, the low-frequency approximations would hinder the discovery of the original patterns. Differently, other comparatives were all single-scale methods and less impacted by such a characteristic.

We believe this is an interesting open problem for multi-scale formula discovery, which is worth further investigation. One possible direction is to explicitly model the system and discover formulas in a multi-scale fashion.

To respond to this comment, we have made the following modifications in the revised manuscript and SI.

-1) We have added a brief discussion on the above findings and a possible solution in Section 3 of the revised manuscript, which is shown as follows.

Revised Manuscript (Section 3):

ity. Nonetheless, on the benchmark AI Feynman dataset, we observe that Deflex has a lower recovery rate on formulas of quantum transition probability derived from time-dependent perturbation theory [45] (see SI for detailed introduction and the recovery comparison). We guess that this phenomenon exposes a potential limitation of Deflex's multiscale discovery. That is, when candidate structures of markedly different magnitudes coexist, low-frequency approximations may overwhelm the original high-frequency but complex structures. This suggests an open problem in balancing competing scales so that small yet physically meaningful terms are not suppressed.

ing neural guidance with type-theoretic constraints. Third, regarding interferences of

the approximated patterns among multiple scales, a promising direction is to separate scales explicitly in the discovery procedures, including problem formulation, rather than extracting implicitly within a single joint run. Also, it is important to establish

-2) We have also added the detailed analysis and validation results about this finding in Subsection 4.3 of the revised SI, which are shown as follows.

Revised SI (Section 7.7):

7.7 Quantum transition probability laws in the AI-Feynman benchmark

Quantum transition probabilities describe the likelihood of a system moving between states due to perturbations. Its probability mass function can be written as

$$p(t) = \frac{p_d E_f t}{\hbar} \cdot \frac{\sin^2((\omega - \omega_0)t/2)}{((\omega - \omega_0)t/2)^2}, \tag{19}$$

where $\hbar = h/(2\pi)$, p_d, E_f, ω, and ω_0 denote the transition dipole moment, field amplitude, driving angular frequencies, and transition angular frequencies, respectively. This function can be found from the AI-Feynman dataset [14]. The discovered formulas of quantum transition probabilities often demonstrate a distinct characteristic. That is, their original form is rather complicated and corresponds to a high-frequency function on certain scales, but can be approximated by low-frequency functions on other scales with simple forms.

Supplementary Table 17 compares the recovery rates of different methods on quantum transition probability formulas from the AI-Feynman benchmark. Specifically, we performed the formula discovery process on the AI-Feynman dataset 20 times and calculated the average number of times that the quantum transition probability formulas were successfully identified. As shown, Deflex demonstrates a lower recovery rate on this type of formulas than the compared baseline methods. The potential reason for low recovery would be due to the adverse effect of Deflex's multi-scale discovery. That is, Deflex searches for multiple formulas at different scales simultaneously. While formulas with very different scales coexist, strong patterns could dominate and suppress other correct formulas, leading to a lowered recovery rate. As we mentioned above, the original formula could be easily approximated by low-frequency functions on different scales with simple forms. In such cases, the low-frequency approximations would hinder the recovery of the original patterns of quantum transition probabilities. Differently, other comparatives were all single-scale methods and less impacted.

Supplementary Table 17: Comparison of average recovery rate on quantum transition probability formulas from the total 20 runs on the AI-Feynman benchmark.

Method	Recovery frequency	Recovery rate
Deflex	10/20	50%
AI Feynman	16/20	80%
PySR	12/20	60%
Operon	13/20	65%
SciMED	14/20	70%
gplearn	13/20	65%

=======Reviewer #4=======

Comments to the Authors:

First, I would like to thank the authors for their extensive response to my questions. I think the many clarifications and inclusion of the Johns Hopkins Database have substantially strengthened the manuscript. I still have some remaining questions and confusion.

Response to Reviewer #4:

We thank the reviewer for this positive comment and the previous constructive comments and suggestions.

Comment 4.1:

Table 2 shows that Operon and SINDy both fail to capture the Navier-Stokes equation (NSE), however the supplemental material shows Operon succeeds at this for the 2D cylinder, and your response to comment 4.1 suggests that SINDy was able to find the Navier-Stokes after changing the settings. Table 2 makes it seem like these methods fail at finding the NSE, which is misleading if they work in some scenarios.

Response to Comment 4.1:

We thank the reviewer for this series of insightful comments. In our last revision, the successful discovery of the Navier-Stokes equation (NSE) refers to the case that a single method can discover all the variants of NSE to ensure a reliable report of discovery capability. SINDy and Operon were marked as unable to discover NSE because they could not find the 3D Navier–Stokes equations (in 3D flow fields). Please note that, we found that, SINDy and Operon could only recover the Navier–Stokes equations in the 2D scenario. Even so, the discovery accuracy was low as the pressure term was often missing. Instead, Deflex can discover the complete form of Navier–Stokes equations in both cases.

Nonetheless, we agree with the reviewer that this strict principle might cause confusions for readers following these methods. To avoid misunderstanding, we have changed the corresponding entries as being able to discover NSE but clarified the differences in the manuscript. Specifically, we have made the following modifications in the revised manuscript, which are shown as follows.

-1) We have revised Table 2 in the manuscript to accurately summarize the discovery capability of compared methods.

Revised Manuscript (Table 2):

Table 2 Comparison of methods on discovery capability

Rules	Deflex	Operon	SciMED	SINDy	gplearn	AI Feynman	PySR	DEAP
Energy conservation	✓	✓	✓	✓	✓	✓	✓	✓
Momentum conservation	✓	✓	✓	✓	✓	✓	✓	✓
MBD	✓	✓	✓	N.A.	✓	✓	✓	✓
MBD with potential	✓	N.A.	N.A.	N.A.	✗	N.A.	N.A.	✗
Continuity of quantity	✓	✓	✓	✓	✓	✓	✓	✓
Newton viscosity law	✓	✓	✓	✓	✓	✓	✓	✓
Velocity distribution	✓	✗	✗	N.A.	✗	✓	✗	✗
Navier-Stokes equation	✓	✓[1]	✗	✓[1]	✗	✗	✗	✗
Langevin	✓	✗	✗	N.A.	✗	✗	✗	✗
Lévy flight (Human)	✓	✗	✗	N.A.	✗	✓	✓	✓
Lévy flight (Avian)	✓	✗	✗	N.A.	✗	✓	✓	✓
Power law	✓	✗	✗	N.A.	✗	✓	✓	✓

Operon and SINDy can recover a 2D Navier–Stokes form (cylinder wake) but fail in the 3D JHTDB setting; moreover, their recovered 2D forms do not include pressure-field terms (i.e., ∇p).
✓ denotes a successful discovery, ✗ denotes a failure, and N.A. denotes an unsupported task (i.e., not applicable).

-2) We have added a brief discussion on the discovery capability of Operon and SINDy in terms of discovering NSE in Subsection 2.6 of the revised manuscript.

Revised Manuscript (Subsection 2.6):

> As listed in Table 1, Deflex detects the Navier-Stokes equation of fluid dynamics through small-scale observations, which cannot be discovered by other methods. In larger-scale random observations, Deflex extracts the velocity distribution of the fluid within the vortex flow. These formulas correspond to different manifestations of long-standing laws in physics. For complex distribution formulas like velocity distribution, most compared methods are infeasible (shown in Table 2), and only AI Feynman and Deflex manage to discover. Notably, from the discovered Navier-Stokes equation, we can further identify the term corresponding to Newton's viscosity law, which is also listed among our discoveries in Tables 1 and 2. Meanwhile, SINDy and Operon can recover a simplified form of 2D Navier–Stokes without pressure-field terms (i.e., ∇p), which leads to noticeably larger errors than Deflex, even though the simpler structure can make their discovery faster. Deflex is the only method that can recover the full 3D Navier–Stokes equation in the JHTDB setting.

Comment 4.2:

Why were spatial derivatives provided as inputs for finding the NSE? Why are these functions not simply learned from the data? The state of the system is only defined by the velocity and the pressure, so the spatial derivatives should not need to be supplied. Does the method fail if the spatial derivatives are not included?

Response to Comment 4.2:

Thank you for this insightful comment. The spatial derivatives were provided as inputs because they were already included in the original public dataset and we directly used them.

These functions are not simply learned from the data because we aim to ensure a fair comparison with the state-of-the-art methods. Please note that, the derivative features are necessary for some compared methods like Operon, which cannot learn higher-order operators like gradients by themselves, unless dedicated templates or manual feature engineering are introduced.

We agree that the state of the system is only defined by the velocity and the pressure and the spatial derivatives should not need to be supplied for Deflex. And we admit that providing these derivatives would undermine the capability validation of the end-to-end formula discovery.

In fact, our method Deflex can successfully learn these functions (e.g., NSE) from the state data (i.e., the velocity and pressure only) directly. In particular, we have conducted new experiments on the modified datasets, which remove all the derivative features. New experimental results demonstrate that Deflex can almost discover all the formulas again, although there will be a slightly higher accuracy loss and prolonged run-time due to the implicit learning of these derivative terms. It is worth mentioning that among all compared methods, Deflex is the only one can discover NSE over the datasets without derivative features. In principle, by using Lambda calculus, our method Deflex can naturally represent the higher-order operators, thus enabling the discovering of complex functions without derivative terms from the observation data. Please note that, as responded in

Response to Comment 1.1, besides spatial derivatives, we have also removed the temporal derivatives, Deflex can achieve successful discovery from the state data of fluids and particles.

To respond to this comment, we have made the following modifications in the revised manuscript and SI, which are shown as follows.

-1) We have updated Fig. 4 in the revised the manuscript to include the new experimental results that compares the discovery performance on the dataset with and without derivative features.

Revised Manuscript (Fig. 4):

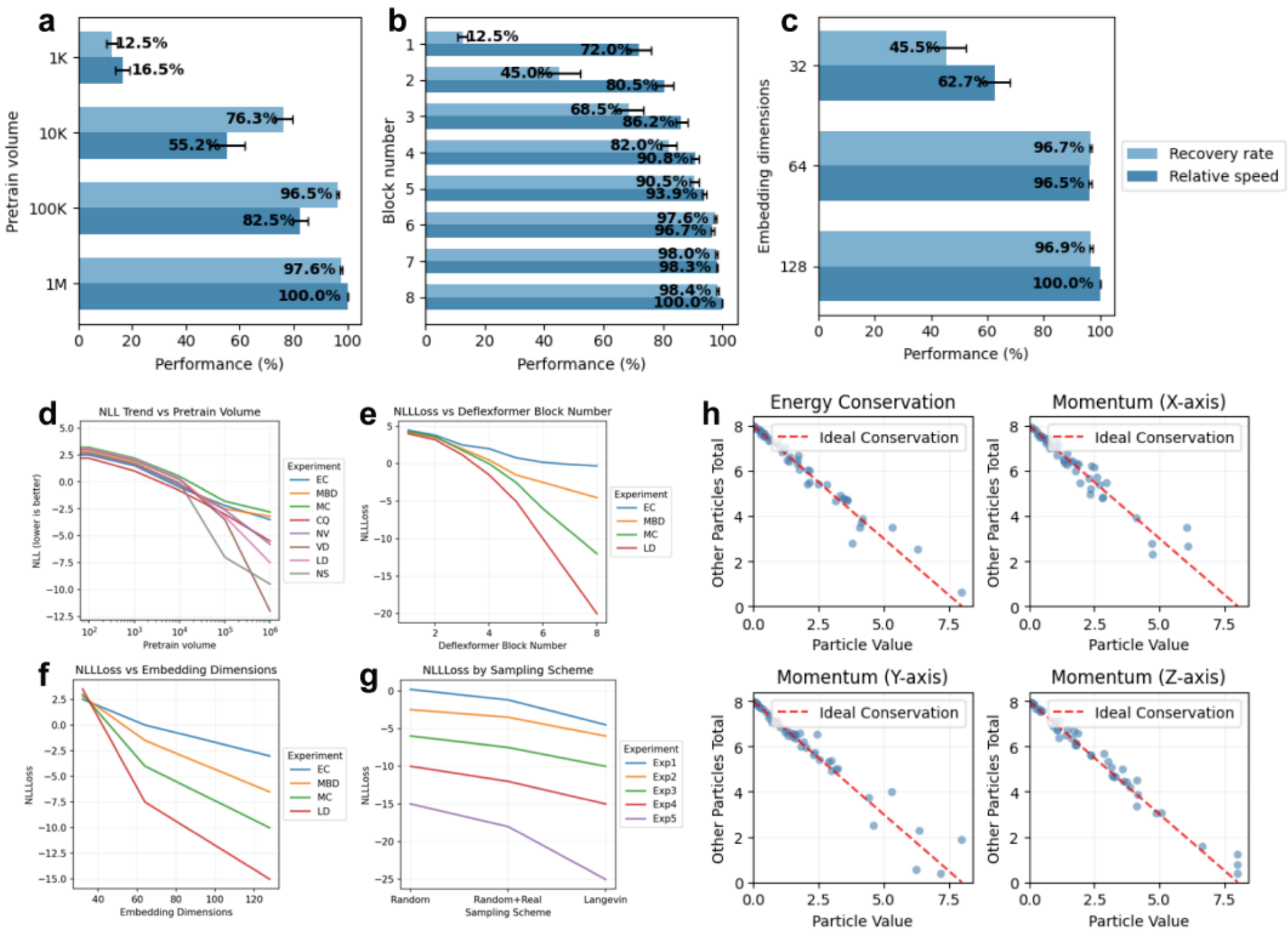

Fig. 4 Ablation studies and component analysis of Deflex. a, e, Impact of pre-training volume on formula discovery performance (recovery rate and relative speed) and NLL trend. Larger pre-training volumes significantly accelerate discovery and improve accuracy. **b**, f, Performance metrics and NLL loss with varying Deflexformer block numbers. Increasing depth improves representation power. **c**, g, Effect of embedding dimensions on performance. **d**, Ablation on derivative (gradient) features, comparing the default feature configuration against a variant where temporal-derivative and spatial-gradient channels are removed from the input. **h**, NLL loss comparison across different sampling schemes (Random, Random+Real, Langevin). Langevin sampling yields the best fitness. i, Verification of conservation laws (Energy and Momentum) learned by the Deflexformer, showing close alignment between sampled states (black dots) and ideal laws (red lines).

-2) We have also summarized the comparison results in Fig. 4 in the revised manuscript.

Revised Manuscript (Subsection 2.8):

cating that these configurations provide sufficient capacity for the tested systems. We also conducted ablation studies on the derivative (gradient) features from the datasets (Fig. 4d), finding that accuracy is mostly preserved but discovery efficiency degrades, and the complicated 3D Navier–Stokes case (JHTDB) does not reliably converge under the same time budget. Moreover, the Deflexformer effectively internalizes physical con-

3) We have also added the detailed experimental setups, results and analysis for ablation studies on

derivative features in Subsection 7.5 of the revised SI.

Revised SI (Subsection 7.5):

7.5 Ablation study on derivative (gradient) features

In the simulated spatiotemporal systems (e.g., the fluid and particle systems), the governing laws are naturally expressed with temporal derivatives (e.g., $\partial \mathbf{u}/\partial t$) and spatial gradients (e.g., $\nabla \mathbf{u}$ and ∇p). In our default feature construction for such datasets (e.g., fluid dynamics; see Supplementary Table 6), these derivative features are treated as part of the observation feature set: they are either directly provided by the simulator/dataset (e.g., JHTDB) or computed deterministically from the raw fields using standard finite-difference schemes (e.g., the cylinder wake setup).

To evaluate Deflex's end-to-end discovery capability from the raw data, we further conducted experiments over the fluid and particle systems, where all the spatial-derivative and temporal-derivative features were removed. In particular, Deflex was set up with the same training protocol, stopping criteria, and time budget as the default setting.

In these experiments, except for the 3D Navier–Stokes equation in the JHTDB setting, all discovered formulas in the previous experiments were recovered. Specifically, the 3D Navier–St equation could not be recovered in a reasonable time without the derivative features. This failure would be due to its substantially higher structural complexity. Once derivative features were removed from the observation dataset, they must be represented implicitly through difference-based expressions, which significantly increases the required expression complexity. Although such representations remain expressible within our operator set, they typically require much deeper expression trees and significantly higher computational cost, which would go beyond the search configuration (including the initial population size and runtime budget) in our experiments.

We further compare their performance in both cases. Supplementary Fig. 8 compares both the accuracy and efficiency of Deflex on the spatial-temporal systems with and without the derivative features. As shown, removing the derivative features caused an accuracy loss in both NLL and RMSE. The reason is that learning these derivatives from the raw data would introduce additional errors, increasing the overall loss. On the other hand, the runtime was prolonged due to an additional search for these derivative features.

Note that we do not compare the performance of the baselines in this ablation study. The reason is that most compared symbolic regression frameworks operate on fixed-dimensional feature vectors and do not natively support expressing operators that traverse spatiotemporal neighborhoods or perform high-order reductions over variable-sized adjacency. In particular, without these derivative features, without additional adaptations (e.g., injecting finite-difference stencil operators and explicit aggregation/traversal routines into their symbol sets, or constraining the expression space to enforce such structures), these baselines could barely learn the formulas with derivative terms.

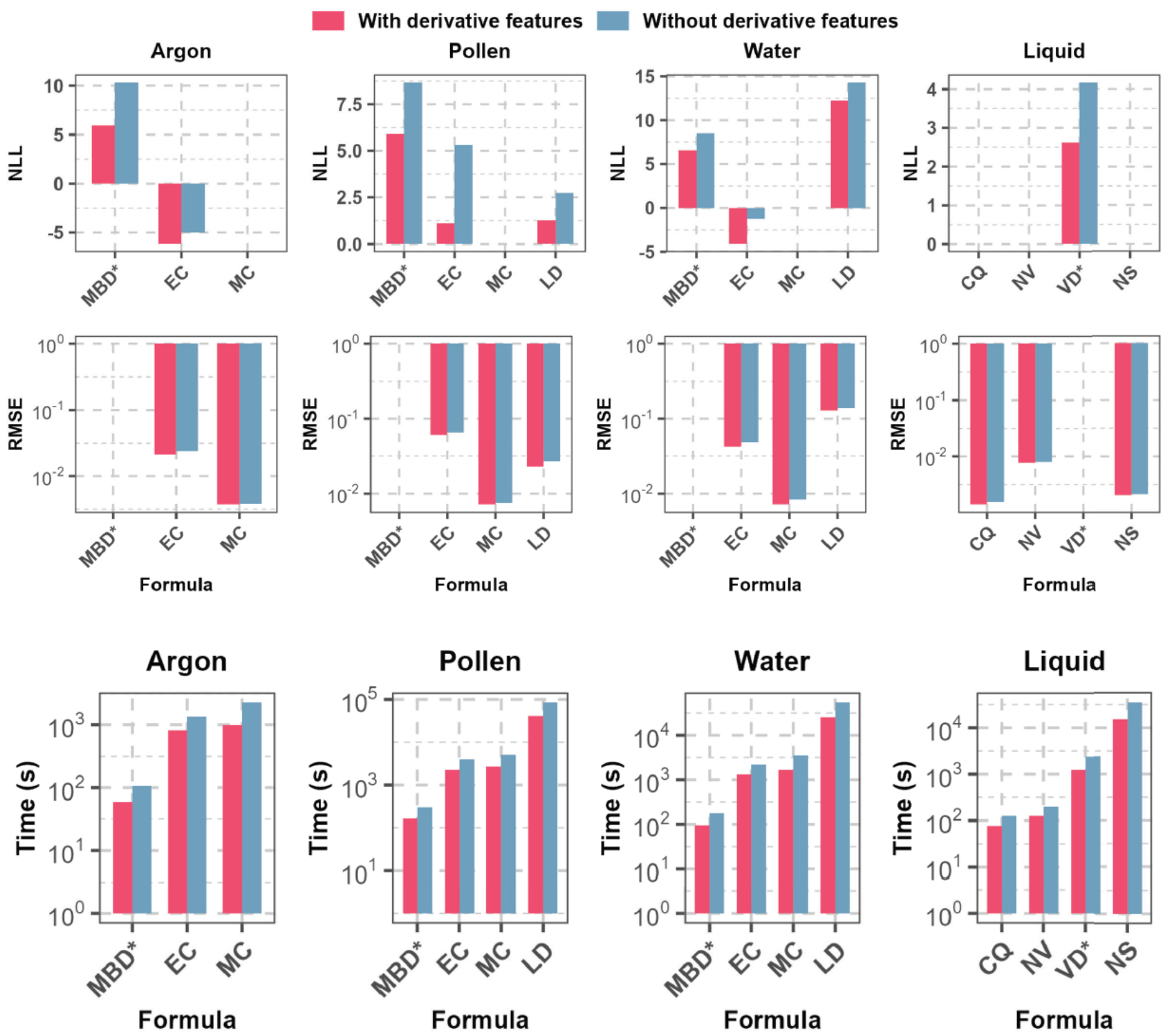

Supplementary Figure 8: Ablation study on derivative (gradient) features. We compare the discovery performance of Deflex on the datasets with and without derivative features. Top row reports NLL for distributional targets, middle row reports RMSE for equation/invariant targets, and bottom row reports runtime. Removing derivative features generally preserves accuracy but reduces efficiency; All formulas were recovered again except for the 3D Navier–Stokes equation (JHTDB), which did not reliably converge within the same time budget and stopping criteria after removing derivative features.

Comment 4.3:

In Table S8, Deflex learns the incorrect coefficients for energy conservation, Maxwell-Boltzmann, and Newtonian viscosity, whereas all of the other approaches do not get these incorrect coefficients. This appears to be a drawback of Deflex that is never explicitly stated. Why is Deflex the only method that gets these incorrect coefficients?

Response to Comment 4.3:

Many thanks for pointing out this issue. In fact, all methods, including Deflex, got imprecise coefficients due to the inherent errors of datasets and the discovery errors caused by methods themselves. We agree that we have not reported them in the formulas found by the compared methods during the results analysis in the previous manuscript. After a careful examination of the experimental results, we have added the imprecise coefficients for all equations in Supplementary Table 8 in SI, which are also shown as follows.

Revised SI (Supplementary Table 8):

Law Name	Methods	Formula Form
Energy conservation	Deflex	`E = 0.992 * mass * (vel_x^2 + vel_y^2 + vel_z^2) / (k_B * temperature)`
	Operon	`E = 1.063 * mass * (vel_x^2 + vel_y^2 + vel_z^2) / (k_B * temperature)`
	SciMED	`E = 1.052 * mass * (vel_x^2 + vel_y^2 + vel_z^2) / (k_B * temperature)`
	SINDy	`E = 1.071 * mass * (vel_x^2 + vel_y^2 + vel_z^2) / (k_B * temperature)`
	gplearn	`E = 1.078 * mass * (vel_x^2 + vel_y^2 + vel_z^2) / (k_B * temperature)`
	AI Feynman	`E = 1.049 * mass * (vel_x^2 + vel_y^2 + vel_z^2) / (k_B * temperature)`
	PySR	`E = 1.066 * mass * (vel_x^2 + vel_y^2 + vel_z^2) / (k_B * temperature)`
	DEAP	`E = 1.058 * mass * (vel_x^2 + vel_y^2 + vel_z^2) / (k_B * temperature)`
Momentum conservation	Deflex	`E = (sum(mass * vel_x) - C_x)^2 + (sum(mass * vel_y) - C_y)^2 + (sum(mass * vel_z) - C_z)^2`
	Operon	`1.063 * sum(mass(i) * vel_x(i)) = C_x, 1.063 * sum(mass(i) * vel_y(i)) = C_y, 1.063 * sum(mass(i) * vel_z(i)) = C_z`

Law Name	Methods	Formula Form
	SciMED	1.052 * sum(mass(i) * vel_x(i)) = C_x,<br>1.052 * sum(mass(i) * vel_y(i)) = C_y,<br>1.052 * sum(mass(i) * vel_z(i)) = C_z
	SINDy	1.071 * sum(mass(i) * vel_x(i)) = C_x,<br>1.071 * sum(mass(i) * vel_y(i)) = C_y,<br>1.071 * sum(mass(i) * vel_z(i)) = C_z
	gplearn	1.078 * sum(mass(i) * vel_x(i)) = C_x,<br>1.078 * sum(mass(i) * vel_y(i)) = C_y,<br>1.078 * sum(mass(i) * vel_z(i)) = C_z
	AI Feynman	1.049 * sum(mass(i) * vel_x(i)) = C_x,<br>1.049 * sum(mass(i) * vel_y(i)) = C_y,<br>1.049 * sum(mass(i) * vel_z(i)) = C_z
	PySR	1.066 * sum(mass(i) * vel_x(i)) = C_x,<br>1.066 * sum(mass(i) * vel_y(i)) = C_y,<br>1.066 * sum(mass(i) * vel_z(i)) = C_z
	DEAP	1.058 * sum(mass(i) * vel_x(i)) = C_x,<br>1.058 * sum(mass(i) * vel_y(i)) = C_y,<br>1.058 * sum(mass(i) * vel_z(i)) = C_z
Maxwell-Boltzmann (MBD)	Deflex	E = 0.511 * mass * (vel_x^2 + vel_y^2 + vel_z^2) / (k_B * temperature)
	Operon	P ~ exp(-1.063 * mass * (vel_x^2 + vel_y^2 + vel_z^2) / (2 * k_B * temperature))
	SciMED	P ~ exp(-1.052 * mass * (vel_x^2 + vel_y^2 + vel_z^2) / (2 * k_B * temperature))
	gplearn	P ~ exp(-1.078 * mass * (vel_x^2 + vel_y^2 + vel_z^2) / (2 * k_B * temperature))
	AI Feynman	P ~ exp(-1.049 * mass * (vel_x^2 + vel_y^2 + vel_z^2) / (2 * k_B * temperature))
	PySR	P ~ exp(-1.066 * mass * (vel_x^2 + vel_y^2 + vel_z^2) / (2 * k_B * temperature))

Law Name	Methods	Formula Form
	DEAP	P ~ exp(-1.058 * mass * (vel_x^2 + vel_y^2 + vel_z^2) / (2 * k_B * temperature))
MBD with potential	Deflex	E = 0.506 * mass * (vel_x^2 + vel_y^2 + vel_z^2) / (k_B * temperature) + sum(0.97 * ((pos_x(i)-pos_x(j))^2+(pos_y(i)-pos_ - 1.89 * ((pos_x(i)-pos_x(j))^2+(pos_y(i)-pos_y(j)
Continuity of quantity	Deflex	E = (vel_x_dx + vel_y_dy)^2 * 10^26
	Operon	vel_x_dx + vel_y_dy = 0
	SciMED	vel_x_dx + vel_y_dy = 0
	SINDy	vel_x_dx + vel_y_dy = 0
	gplearn	vel_x_dx + vel_y_dy = 0
	AI Feynman	vel_x_dx + vel_y_dy = 0
	PySR	vel_x_dx + vel_y_dy = 0
	DEAP	vel_x_dx + vel_y_dy = 0
Newton viscosity law	Deflex	τ_xy = -1.037 * μ * (vel_x_dy + vel_y_dx)<br>τ_xz = -1.037 * μ * (vel_x_dz + vel_z_dx)<br>τ_yz = -1.037 * μ * (vel_y_dz + vel_z_dy)
	Operon	τ_xy = -1.063 * μ * (vel_x_dy + vel_y_dx)<br>τ_xz = -1.063 * μ * (vel_x_dz + vel_z_dx)<br>τ_yz = -1.063 * μ * (vel_y_dz + vel_z_dy)
	SciMED	τ_xy = -1.052 * μ * (vel_x_dy + vel_y_dx)<br>τ_xz = -1.052 * μ * (vel_x_dz + vel_z_dx)<br>τ_yz = -1.052 * μ * (vel_y_dz + vel_z_dy)

Law Name	Methods	Formula Form
	SINDy	τ_xy = −1.071 ⋆ μ ⋆ (vel_x_dy + vel_y_dx)<br>τ_xz = −1.071 ⋆ μ ⋆ (vel_x_dz + vel_z_dx)<br>τ_yz = −1.071 ⋆ μ ⋆ (vel_y_dz + vel_z_dy)
	gplearn	τ_xy = −1.078 ⋆ μ ⋆ (vel_x_dy + vel_y_dx)<br>τ_xz = −1.078 ⋆ μ ⋆ (vel_x_dz + vel_z_dx)<br>τ_yz = −1.078 ⋆ μ ⋆ (vel_y_dz + vel_z_dy)
	AI Feynman	τ_xy = −1.049 ⋆ μ ⋆ (vel_x_dy + vel_y_dx)<br>τ_xz = −1.049 ⋆ μ ⋆ (vel_x_dz + vel_z_dx)<br>τ_yz = −1.049 ⋆ μ ⋆ (vel_y_dz + vel_z_dy)
	PySR	τ_xy = −1.066 ⋆ μ ⋆ (vel_x_dy + vel_y_dx)<br>τ_xz = −1.066 ⋆ μ ⋆ (vel_x_dz + vel_z_dx)<br>τ_yz = −1.066 ⋆ μ ⋆ (vel_y_dz + vel_z_dy)
	DEAP	τ_xy = −1.058 ⋆ μ ⋆ (vel_x_dy + vel_y_dx)<br>τ_xz = −1.058 ⋆ μ ⋆ (vel_x_dz + vel_z_dx)<br>τ_yz = −1.058 ⋆ μ ⋆ (vel_y_dz + vel_z_dy)
Velocity distribution	Deflex	E = 0.501 ⋆ mass ⋆ (vel_x^2 + vel_y^2) + (δv)^3.15
	AI Feynman	P ~ exp(−(0.5 ⋆ mass ⋆ (vel_x^2 + vel_y^2) + (δv)^p))

Law Name	Methods	Formula Form
Navier-Stokes equation (2D Cylinder)	Deflex	E = (ρ * (vel_x_dt + vel_x * vel_x_dx + vel_y * vel_x_dy) + pressure_dx − μ * (vel_x_dx_dx + vel_x_dy_dy))^2 * 1.62e21 + (ρ * (vel_y_dt + vel_x * vel_y_dx + vel_y * vel_y_dy) + pressure_dy − μ * (vel_y_dx_dx + vel_y_dy_dy))^2 * 1.62e21
	Operon	ρ * (vel_x_dt + vel_x * vel_x_dx + vel_y * vel_x_dy) + pressure_dx − 0.990 * μ * (vel_x_dx_dx + vel_x_dy_dy) = 0, ρ * (vel_y_dt + vel_x * vel_y_dx + vel_y * vel_y_dy) + pressure_dy − 0.990 * μ * (vel_y_dx_dx + vel_y_dy_dy) = 0
	SINDy	ρ * (vel_x_dt + vel_x * vel_x_dx + vel_y * vel_x_dy) − 0.992 * μ * (vel_x_dx_dx + vel_x_dy_dy) = 0, ρ * (vel_y_dt + vel_x * vel_y_dx + vel_y * vel_y_dy) − 0.993 * μ * (vel_y_dx_dx + vel_y_dy_dy) = 0
	AI Feynman	ρ * (vel_x_dt + vel_x * vel_x_dx + vel_y * vel_x_dy) − 0.985 * μ * (vel_x_dx_dx + vel_x_dy_dy) = 0, ρ * (vel_y_dt + vel_x * vel_y_dx + vel_y * vel_y_dy) − 0.981 * μ * (vel_y_dx_dx + vel_y_dy_dy) = 0
Navier-Stokes equation (3D JHTDB)	Deflex	E = (ρ * (vel_x_dt + vel_x * vel_x_dx + vel_y * vel_x_dy + vel_z * vel_x_dz) + pressure_dx − μ * (vel_x_dx_dx + vel_x_dy_dy + vel_x_dz_dz))^2 * 3.27e21 + (ρ * (vel_y_dt + vel_x * vel_y_dx + vel_y * vel_y_dy + vel_z * vel_y_dz) + pressure_dy − μ * (vel_y_dx_dx + vel_y_dy_dy + vel_y_dz_dz))^2 * 3.27e21 + (ρ * (vel_z_dt + vel_x * vel_z_dx + vel_y * vel_z_dy + vel_z * vel_z_dz) + pressure_dz − μ * (vel_z_dx_dx + vel_z_dy_dy + vel_z_dz_dz))^2 * 3.27e21

Law Name	Methods	Formula Form
	SINDy	ρ * (vel_x_dt + vel_x * vel_x_dx + vel_y * vel_x_dy + vel_z * vel_x_dz) − 0.94 * μ * (vel_x_dx_dx + vel_x_dy_dy + vel_x_dz_dz) = 0, ρ * (vel_y_dt + vel_x * vel_y_dx + vel_y * vel_y_dy + vel_z * vel_y_dz) − 0.94 * μ * (vel_y_dx_dx + vel_y_dy_dy + vel_y_dz_dz) = 0, ρ * (vel_z_dt + vel_x * vel_z_dx + vel_y * vel_z_dy + vel_z * vel_z_dz) − 0.94 * μ * (vel_z_dx_dx + vel_z_dy_dy + vel_z_dz_dz) = 0
	AI Feynman	ρ * (vel_x_dt + vel_x * vel_x_dx + vel_y * vel_x_dy + vel_z * vel_x_dz) − 0.86 * μ * (vel_x_dx_dx + vel_x_dy_dy + vel_x_dz_dz) = 0, ρ * (vel_y_dt + vel_x * vel_y_dx + vel_y * vel_y_dy + vel_z * vel_y_dz) − 0.86 * μ * (vel_y_dx_dx + vel_y_dy_dy + vel_y_dz_dz) = 0, ρ * (vel_z_dt + vel_x * vel_z_dx + vel_y * vel_z_dy + vel_z * vel_z_dz) − 0.86 * μ * (vel_z_dx_dx + vel_z_dy_dy + vel_z_dz_dz) = 0
Langevin	Deflex	E = (4.438e16 * mass * vel_x_dt + 1.003 * vel_x)^2 + (4.438e16 * mass * vel_y_dt + 1.003 * vel_y)^2 + (4.438e16 * mass * vel_z_dt + 1.003 * vel_z)^2
	Operon	4.468e16 * mass * vel_x_dt + 1.063 * vel_x = 0, 4.468e16 * mass * vel_y_dt + 1.063 * vel_y = 0, 4.468e16 * mass * vel_z_dt + 1.063 * vel_z = 0
Lévy flight (Human)	Deflex	E = 2.37 * log(sqrt((lat(time) − lat(time-1))^2 + (lon(time) − lon(time-1))^2))

Comment 4.4:

Even with the supplemental material, it is hard to decipher the Lambda Calculus sections. For example, Eq. 4 contains a substantial number of things that are never defined, like "tail" and "head", and from the brief description, I am unable to determine what "Fix" is doing. In the manuscript's current state, I do not think many readers will understand the sections on the Lambda Calculus.

Response to Comment 4.4:

We thank the reviewer for pointing out the readability issue and undefined symbols in the Lambda Calculus section.

In particular, Eq. (4) aims to demonstrate an example of a lambda expression including high-order operators, especially its capability of defining recursive functions over a variable-length input list. As the terms in Eq. (4) are commonly used functions in Lambda calculus [1], we did not define them in our original manuscript. We would like to clarify their meanings as follows. Here, $Head(xs)$ refers to the first element of a list xs with arbitrary length, $Tail(xs)$ refers to the remaining list after removing the first element, and $Null(xs)$ checks whether the list is empty. $Fix(\cdot)$ is a fixed-point operator (i.e., the Y combinator): it takes a non-recursive one-step update rule (a "generator") and turns it into a self-referential function that can call itself. It can be written as

$$Fix = \lambda g.(\lambda x.g(xx))(\lambda x.g(xx)),$$

and satisfies $Fix(G) = G(Fix(G))$. Therefore, if we write the one-step generator in Eq. (4) as

$$G(f, xs, a) = if\,Null(xs)\ then\ a\ else\ f(Tail(xs))(a + V(Head(xs))),$$

and let $F = Fix(G)$, then F is exactly the corresponding recursive aggregation function, i.e.,

$$F(xs, a) = if\,Null(xs)\ then\ a\ else\ F(Tail(xs), a + V(Head(xs))).$$

We agree these abstract terms and concepts may be difficult to follow for readers not familiar with the area. Therefore, we have conducted a thorough revision in Subsection 4.3 of the manuscript and corresponding SI to improve the readability. The main modifications are summarized as follows.

-1) We have revised the introductive paragraph of Subsection 4.3, which briefly describes the overall functions of Deflexpressor, the lambda-calculus-based symbolic regression system.

Revised Manuscript (Subsection 4.3):

4.3 Deflexpressor: SR subsystem based on lambda calculus

Traditional tree-based SR relies on first-order expression trees and manually specified higher-order primitives, which makes it difficult to learn relationships such as summation or mapping over massive interacting elements in complex systems. To this end, we present Deflexpressor, an SR system based on the extended lambda calculus, which supports various higher-order expressions while ensuring valid types and variable length of elementary inputs. In particular, Deflexpressor leverages a strict rule-based generator together with an efficient neural network-based one to generate a large number of valid candidate lambda expressions for mathematical relationships, which are then evaluated to identify Pareto-optimal expressions in terms of accuracy and conciseness.

-2) We have substantially revised the Subsubsection 4.3.1 of the manuscript, where modifications include adding a brief introduction to lambda calculus, highlighting its advantages over traditional expression trees, explaining Eq. 4 with clear visualization.

Revised Manuscript (Subsection 4.3.1):

4.3.1 Expressing relationships by lambda calculus

Lambda calculus is a formal system in mathematical logic and computer science for expressing computation based on function abstraction and application [51]. A lambda term is built from three kinds of expressions: (i) Variable x denotes a placeholder for a value or parameter; (ii) Abstraction $\lambda x.M$ defines a function with parameter x and body M; (iii) Application MN applies function M to argument N. Although there is no built-in data, the lambda calculus can encode data and control structures (e.g., recursion) as functions via Church encoding. The lambda calculus is Turing-complete and can simulate any computation based on symbol replacement. Specifically, for any expression made of variables, symbol λ, and parentheses, we can find a pattern $(\lambda x.M)N$ and replace it with M where every x is replaced by N. By repeating this process until no such pattern remains, we can obtain the final expression as the result. The basic grammar rules are provided in Section 3.1 in SI.

Since the lambda calculus is Turing-complete and natively supports function abstraction and recursion, Deflexpressor can express operations such as summation and mapping directly rather than as fixed templates. Specifically, operations over collections can be written as recursively defined functions or higher-order maps, enabling the system to learn how to aggregate interactions from data without requiring pre-specified structures. For instance, we consider a common formula $E = \sum_{i \neq j} V(r_{ij})$ that describes the aggregation of a variable number of particle interactions. Here, $V(r_{ij})$ denotes a function that calculates the pairwise interaction potential based on the distance r_{ij} between two particles indexed by i and j. Traditional SR either requires hard-coding symbols or extremely deep trees to express such a relationship. In contrast, suppose that $\mathbf{x} = [r_{ij}]_{i \neq j}$ denotes the list that enumerates these distances under any fixed ordering, Deflexpressor can naturally write the aggregation as the following Lambda expression.

$$\underbrace{\left(\underbrace{\mathrm{Fix}\left(\underbrace{\underbrace{\lambda f\,\lambda \mathrm{xs}\,\lambda a.}_{\text{Variables}}\;\underbrace{\underbrace{\text{if Null(xs) then } a}_{\text{Application w.r.t. relationship (i)}}\;\text{else}\;\underbrace{f\,(\mathrm{Tail(xs)})\,(a+V(\mathrm{Head(xs)}))}_{\text{Application w.r.t. relationship (ii)}}}_{\text{Function body describing the whole relationship (i and ii) among } f, \mathrm{xs}, a}}_{\text{Abstraction defining a function G(f, xs, a) over variables } f, \mathrm{xs}, a}\right)}_{\text{Fixed-point simulating a recursive function F(xs, a) based on G(f, xs, a)}}\right)\,\mathbf{x}\;0}_{\text{Application denoting applying recursive function F(xs, a) over parameters } \mathbf{x} \text{ and } 0} \tag{5}$$

Eq. (5) defines a recursive aggregation function F via a fixed-point construction and applies it to a variable-length list $\mathbf{x}$ and an initial accumulator 0. The underbraces decompose the term: the innermost abstraction defines a one-step update rule $G(f, \mathrm{xs}, a)$ whose body implements (i) a base case that returns a when Null(xs) and (ii) an update-and-continue step that calls f on Tail(xs) with the updated accumulator $a + V(\mathrm{Head(xs)})$. The operator $\mathrm{Fix}(\cdot)$ turns this update rule into a self-referential recursion F (formal definition in the SI), and application associates to the left, so $F\,\mathbf{x}\,0$ means $(F\,\mathbf{x})\,0$. See SI for a step-by-step expansion and an explicit walk-through of the bracketed decomposition. This demonstrates that the system constructs the algorithm of summation from scratch, covering cases where predefined summation templates are inapplicable or insufficient.

Besides strong expressiveness, another merit of the lambda calculus is that its relatively unified structure enables intuitive comparison of the complexity of formulas. In particular, we can directly use the expression length as the complexity metric $R(f)$ (in Eq. 3) in Pareto selection, enabling an explicit trade-off between data fitness and conciseness.

-3) We have also added a basic introduction of lambda calculus in Subsection 3.1 of SI, added a detailed walk-through explanation of Eq. 4 in Subsection 3.2 of SI.

Revised SI (Section 3):

3 Lambda calculus-based symbolic regression

In our work, we use a symbolic regression framework, Deflexpressor, to identify concise lambda calculus-based mathematical patterns. Lambda calculus, introduced by Alonzo Church in the 1930s [4], is a formal system in mathematical logic and computer science for expressing computation based on function abstraction and application.

3.1 Basic introduction to lambda calculus

Lambda calculus represents computation using only functions. Its basic building blocks are (i) defining a function, and (ii) applying a function to an input. Although there are no built-in representations for numbers, booleans, loops, or data structures, they all can be encoded using functions (often called Church encodings [5]).

A lambda term is built from three syntactic forms:

- x (variable): a placeholder denoting a parameter.
- $\lambda x.\, M$ (abstraction): a function with parameter x and body M.
- $M\ N$ (application): applying function M to argument N.

For example, $\lambda x.\, x$ represents the identity function $f(x) = x$, and $(\lambda x.\, x)\ y$ denotes applying function $f(x) = x$ to argument y, which reduces to y.

Two standard conventions remove the ambiguity of computing rules:

- Application is left-associative: $M\ N\ P$ means $(M\ N)\ P$.
- Abstraction extends to the rightmost: $\lambda x.\, M\ N$ means $\lambda x.\, (M\ N)$.

Computation is performed by rewriting terms according to a small set of rules. The key rule is β-reduction (function application):

$$(\lambda x.\, M)\ N \ \to_\beta \ M[x := N]. \tag{8}$$

meaning that N is substituted for each free occurrence of x in M (renaming bound variables when needed to avoid capture). Two additional, commonly used equivalences are: α-conversion (renaming bound variables, e.g., $\lambda x.\, x \equiv \lambda y.\, y$) and η-conversion (extensionality, e.g., $\lambda x.\, M\ x \equiv M$ when x is not free in M).

Since there is no built-in support, recursions can be represented using a fixed-point combinator such as the Y combinator [5]:

$$Y \;=\; \lambda f.\, (\lambda x.\, f(x\,x))(\lambda x.\, f(x\,x)). \tag{9}$$

which satisfies $Y\,F = F\,(Y\,F)$ for any F. Operationally, it turns a non-recursive "one-step update rule" F into a self-referential function.

Lambda calculus is widely used as a foundation for functional programming language semantics and for studying computability and type systems. Programs can be expressed as functions and evaluated by repeated substitution, and higher-level constructs (data, branching, recursion) can be encoded within this minimal core. In this work, we regard each lambda expression as an explicit, compositional function built from abstraction and application that maps structured observations (and optional global context) to a scalar, enabling aggregation over variable-sized entity collections within the expression itself.

3.2 Detailed walk-through of the fixed-point aggregation example

We revisit the aggregation example in the main text, $E = \sum_{i \neq j} V(r_{ij})$, where $V(\cdot)$ denotes a pairwise interaction potential and r_{ij} denotes the distance between particles i and j. To connect this summation to a variable-length input, we collect all pair distances into a list $\mathbf{x} = [r_{ij}]_{i \neq j}$ under any fixed ordering. The following lambda expression implements the aggregation as an executable recursion over $\mathbf{x}$ with an accumulator initialized as 0:

$$E = \underbrace{\Bigg(\underbrace{\mathrm{Fix}\Big(\underbrace{\underbrace{\lambda f\, \lambda \mathrm{xs}\, \lambda a}_{\text{Variables}}.\, \underbrace{\underbrace{\text{if } \mathrm{Null}(\mathrm{xs}) \text{ then } a}_{\text{Application w.r.t. relationship (i)}} \;\text{ else }\; \underbrace{f\;(\mathrm{Tail}(\mathrm{xs}))\;\big(a + V(\mathrm{Head}(\mathrm{xs}))\big)}_{\text{Application w.r.t. relationship (ii)}}}_{\text{(D) Function body describing the whole relationship (i) and (ii) among } f,\, \mathrm{xs},\, a}}_{\text{(C) Abstraction defining a function G(f, xs, a) over variables } f,\, \mathrm{xs},\, a} \Big)}_{\text{(B) Fixed-point simulating a recursive function F(xs, a) based on G(f, xs, a)}} \Bigg)\; \mathbf{x}\; 0}_{\text{(A) Outermost application applying recursive function F(xs, a) over parameters } \mathbf{x} \text{ and } 0}\;. \tag{10}$$

We explain the notations, terms, and computational logic in the above lambda-calculus expression from the inside underbraces to the outside ones as follows.

- Function body (D): the expression (D) denotes a typical conditional sentence of the "if–then–else" style in the lambda calculus, which defines the body of a function. Within it, the relationship (i) returns a if $\mathrm{Null}(\mathrm{xs})$ is true, and relationship (ii) continues by calling f on $\mathrm{Tail}(\mathrm{xs})$ with updated accumulator $a + V(\mathrm{Head}(\mathrm{xs}))$. specifically, $\mathrm{Head}(\mathrm{xs})$ returns the first element of xs, $\mathrm{Tail}(\mathrm{xs})$ returns the remaining list of xs after removing the first element, and $\mathrm{Null}(\mathrm{xs})$ tests whether the list xs is empty. They are predefined functions that are commonly used in lambda calculus.

- Abstraction (C): the abstraction underbrace has a form of $\lambda f\ \lambda \mathrm{xs}, \lambda a.G$ defines a function $G(f, \mathrm{xs}, a)$ with three parameters, where f is the continuation, xs is the remaining list, and a is the current accumulator. As we can see, the inner term (D) indeed describes the logic of the function $G(f, \mathrm{xs}, a)$.
- Fixed-point operation (B): $\mathrm{Fix}(\cdot)$ is a commonly used fixed-point operator in the lambda calculus. Specifically, a fixed-point operation in the form of $\mathrm{Fix}(\lambda f \lambda x.M)$ turns function $M(f, x)$ into a self-referential recursion function $F(x) = M(F(x))$. In the above example, it produces $F(\mathrm{xs}, \mathrm{a}) = G(F(\mathrm{xs}, \mathrm{a}))$ from the inner abstraction (C), which denotes the function $G(f, \mathrm{xs}, \mathrm{a})$.
- Application (A): the whole expression (A) is an application in the form of $F\ \mathbf{x}\ 0$, which means applying a function F constructed from (B) to the inputs $\mathbf{x}$ and 0. $\mathbf{x} = [r_{ij}]_{i \neq j}$ is the variable-length list of pair distances under any fixed ordering. 0 denotes that the accumulator is initialized as 0. In particular, after (B),(C), and (D) are composed, F represents

$$F(\mathrm{xs}, a) = \text{if Null(xs) then } a \text{ else } F(\mathrm{Tail(xs)},\ a + V(\mathrm{Head(xs)})). \tag{11}$$

In the lambda calculus, applications are left-associative, so $F\ \mathbf{x}\ 0$ means $(F\ \mathbf{x})\ 0$. Finally, given $\mathbf{x} = [x_1, x_2, \ldots]$, $F\ \mathbf{x}\ 0 = F\ [x_2, \ldots]\ (0 + V(x_1)) = F\ [x_3, \ldots]\ (0 + V(x_1) + V(x_2)) = \cdots$, which just matches the expression of $\sum_k V(x_k)$.

-4) We have further revised Subsubsection 4.3.2 of the manuscript, which highlights the motivation of extending lambda calculus, adds more explicit explanations to the detailed examples.

Revised Manuscript (Subsubsection 4.3.2):

4.3.2 Extended lambda calculus with types and arrays

The pure lambda calculus is Turing complete, but it has two limitations in our complex system setting. The first is that it may generate meaningless expressions and even non-terminating reductions, which would lead to the low efficiency of the symbolic regression process in Deflexpressor. The second is that data structures like lists or arrays are often encoded as nested higher-order functions, which leads to significantly high complexity in data traversal. To this end, in Deflexpressor, we adopt an extended lambda calculus with types and arrays.

A type specifies what kind of value a sub-expression denotes (e.g., a real number, a vector, or a function), and therefore constrains how expressions can be composed. Specifically, we enforce the following type constraints. (i) Type compliance in the applications. For example, an application $(M\,N)$ is valid only when M denotes a function whose input type matches the type of N. Instead, if $M : \mathbb{R}$ denotes a real-valued number, then $(M\,N)$ will be rejected. (ii) Type requirements for the operands of operators. For instance, expression $M = 1 + (\lambda x.x)$ will be identified as invalid because $\lambda x.x$ (i.e., $f(x) = x$) belongs to a function type, whereas $+$ expects two operands with the real-valued type. Type constraints on input-output format. For example, many tasks require a real-valued function on an n-dimensional state, i.e., $f : \mathbb{R}^n \to \mathbb{R}$. In such cases, a well-typed function term can be used, whereas a constant will be rejected in Deflexpressor. For instance, $E = \sum_{i \neq j} V(r_{ij})$ requires a mapping from a variable-length list of pair distances $\mathbf{x} = [r_{ij}]_{i \neq j}$ to a scalar energy. Eq. (5) has exactly this form (a function that consumes $\mathbf{x}$ and returns E), thus being a type-valid expression. In Deflexpressor, we implement a type inference engine based on the classical type inference algorithm W [52] (see SI for details), which can automatically infer the type of each candidate expression, and then keep only type-valid candidates.

Variable-sized data structures, such as lists, arrays, and vectors, are essential to represent various data in complex systems. However, encoding such structures as functions in pure lambda calculus would lead to extra workload of searching the encoding

itself rather than the relationship of encoded data. To adapt the framework for scientific discovery, we predefine array $\text{Array}[\tau]$ as a built-in primitive data type, and provide $O(1)$-time access and basic operators (e.g., indexing and size). For instance, we treat the pair distances r_{ij} in $E = \sum_{i \neq j} V(r_{ij})$ as an array $\mathbf{x} = [r_{ij}]_{i \neq j}$. This allows the aggregation to be written in the same form as Eq. (5), letting the search focus on learning $V(\cdot)$ and the aggregation structure rather than on data representation details (formal definitions in SI).

-5) We have also revised Subsubsections 4.3.3 and 4.3.4 of the manuscript. In revised Subsections 4.3.3, we have added necessary symbols and examples for explaining the technical details. In revised Subsections 4.3.3, we have unified the symbols and formulas for improving the readability.

Revised Manuscript (Subsubsections 4.3.3 and 4.3.4):

ensure validity. For example, a subtree mutation can replace the local update term $V(\text{Head}(\text{xs}))$ in Eq. (5) with $V'(\text{Head}(\text{xs}))$ without changing the recursion structure. β-reduction further simplifies candidates by evaluating function applications, e.g., $((\lambda x.x)\ y) \to y$ (formal rules in SI). Secondly, relying solely on a neural network can

To accelerate this process without imposing hard constraints, we employ a heuristic initialization strategy that generates the seed expressions performing various aggregations over variable-length inputs. Specifically, these seed expressions are written in the form $\lambda\mathbf{x}.\ \text{Reduce}(\text{kernel}, \mathbf{x})$, which denotes the recursive procedure of updating an aggregation state s by traversing a variable-length input list $\mathbf{x} = [x_1, \ldots, x_N]$ as

follows

$$s \leftarrow \begin{cases} x_1, i = 1 \\ \text{kernel}(s, x_i), i > 1 \end{cases} \tag{6}$$

where $\text{kernel}(s, x_i)$ is a learnable binary operator that updates the aggregation state s using an element x_i of the list $\mathbf{x}$. In particular, the kernel is a randomly generated expression tree that defines the interaction logic between the aggregation state s and each data element x_i of a variable-length list. For example, in $E = \sum_{i \neq j} V(r_{ij})$, we can define a kernel as $\text{kernel}(s, r) = s + V(r)$ and take $\mathbf{x} = [r_{ij}]_{i \neq j}$ as the list. Then the function $\text{Reduce}(\text{kernel}, \mathbf{x})$ can yield the desired aggregation, i.e., it stands for computing E by iteratively applying $s \leftarrow s + V(r)$ over all pairwise distances r_{ij} in $\mathbf{x}$ (with the recursive procedures realized via $\text{Fix}(\cdot)$ as in Eq. (5)). This initialization serves as a soft inductive bias that prioritizes aggregation operations over massive elements, encouraging SR to focus immediately on discovering local interaction laws (encoded in the kernel) while fitting the data. Meanwhile, since $\lambda\mathbf{x}.\ \text{Reduce}(\text{kernel}, \mathbf{x})$

-6) We finally proofread the whole manuscript to ensure all terms and notations are defined with clear meanings.

Reference to the response to the comment:

[1] R. Nederpelt and H. Geuvers, Type Theory and Formal Proof: An Introduction. Cambridge University Press, 2014.

Comment 4.5:

What do you mean that the expression in [10] is more concise, but may not be Pareto-optimal? The NSE is the same regardless of how it is represented. Also, you wrote that the "...new experimental setups and results have been added in both manuscript...", but I did not see where these new SINDy results were added in the manuscript.

Response to Comment 4.5:

We thank the reviewer for this comment. As for the statement “the expression in [10] is more concise, but may not be Pareto-optimal”, we mean that although the expression discovered by SINDy in [10] is more concise, its accuracy may not be better than Deflex's on our experiment dataset. Specifically, in our experiments, SINDy could recover the 2D Navier–Stokes equations but missed a pressure term, which was correctly discovered by Deflex.

We agree with the reviewer that there is only one ground truth of NSE expression. And according to our definition of successful discovery that "the formulas of invariants are considered successful recovery if they can be expressed as the correct formula with an additional stochastic residual term", SINDy in [10] indeed should be regarded as being able to find NSE, which we have admitted in the previous response. However, it should be noted that, SINDy could only discover NSE in 2D fluids and could not in 3D cases.

Regarding our last response "new experimental setups and results have been added in both manuscript", we are sorry for our mistake of not correctly updating the new results in the last revision. In the revision, we have made the following modifications to update the corresponding results.

-1) We have updated Table 2 in the revised manuscript to mark the successful discovery of NSE by SINDy [10]. In addition, we have added notes to clarify the limitation in 2D fluids.

Revised Manuscript (Table 2):

Table 2 Comparison of methods on discovery capability

Rules	Deflex	Operon	SciMED	SINDy	gplearn	AI Feynman	PySR	DEAP
Energy conservation	✓	✓	✓	✓	✓	✓	✓	✓
Momentum conservation	✓	✓	✓	✓	✓	✓	✓	✓
MBD	✓	✓	✓	N.A.	✓	✓	✓	✓
MBD with potential	✓	N.A.	N.A.	N.A.	✗	N.A.	N.A.	✗
Continuity of quantity	✓	✓	✓	✓	✓	✓	✓	✓
Newton viscosity law	✓	✓	✓	✓	✓	✓	✓	✓
Velocity distribution	✓	✗	✗	N.A.	✗	✓	✗	✗
Navier-Stokes equation	✓	✓[1]	✗	✓[1]	✗	✗	✗	✗
Langevin	✓	✗	✗	N.A.	✗	✗	✗	✗
Lévy flight (Human)	✓	✗	✗	N.A.	✗	✓	✓	✓
Lévy flight (Avian)	✓	✗	✗	N.A.	✗	✓	✓	✓
Power law	✓	✗	✗	N.A.	✗	✓	✓	✓

Operon and SINDy can recover a 2D Navier–Stokes form (cylinder wake) but fail in the 3D JHTDB setting; moreover, their recovered 2D forms do not include pressure-field terms (i.e., ∇p).
✓ denotes a successful discovery, ✗ denotes a failure, and N.A. denotes an unsupported task (i.e., not applicable).

-2) We have added the discovered formula of NSE by SINDy [10] in Supplementary Table 8 in the revised SI.

Revised SI (Supplementary Table 8):

Law Name	Methods	Formula Form
Navier-Stokes equation (2D Cylinder)	Deflex	E = (ρ * (vel_x_dt + vel_x * vel_x_dx + vel_y * vel_x_dy) + pressure_dx − μ * (vel_x_dx_dx + vel_x_dy_dy))^2 * 1.62e21 + (ρ * (vel_y_dt + vel_x * vel_y_dx + vel_y * vel_y_dy) + pressure_dy − μ * (vel_y_dx_dx + vel_y_dy_dy))^2 * 1.62e21
	Operon	ρ * (vel_x_dt + vel_x * vel_x_dx + vel_y * vel_x_dy) + pressure_dx − 0.990 * μ * (vel_x_dx_dx + vel_x_dy_dy) = 0, ρ * (vel_y_dt + vel_x * vel_y_dx + vel_y * vel_y_dy) + pressure_dy − 0.990 * μ * (vel_y_dx_dx + vel_y_dy_dy) = 0
	SINDy	ρ * (vel_x_dt + vel_x * vel_x_dx + vel_y * vel_x_dy) − 0.992 * μ * (vel_x_dx_dx + vel_x_dy_dy) = 0, ρ * (vel_y_dt + vel_x * vel_y_dx + vel_y * vel_y_dy) − 0.993 * μ * (vel_y_dx_dx + vel_y_dy_dy) = 0
	AI Feynman	ρ * (vel_x_dt + vel_x * vel_x_dx + vel_y * vel_x_dy) − 0.985 * μ * (vel_x_dx_dx + vel_x_dy_dy) = 0, ρ * (vel_y_dt + vel_x * vel_y_dx + vel_y * vel_y_dy) − 0.981 * μ * (vel_y_dx_dx + vel_y_dy_dy) = 0

Navier-Stokes equation (3D JHTDB)	Deflex	`E = (ρ * (vel_x_dt + vel_x * vel_x_dx + vel_y * vel_x_dy + vel_z * vel_x_dz) + pressure_dx - μ * (vel_x_dx_dx + vel_x_dy_dy + vel_x_dz_dz))^2 * 3.27e21 + (ρ * (vel_y_dt + vel_x * vel_y_dx + vel_y * vel_y_dy + vel_z * vel_y_dz) + pressure_dy - μ * (vel_y_dx_dx + vel_y_dy_dy + vel_y_dz_dz))^2 * 3.27e21 + (ρ * (vel_z_dt + vel_x * vel_z_dx + vel_y * vel_z_dy + vel_z * vel_z_dz) + pressure_dz - μ * (vel_z_dx_dx + vel_z_dy_dy + vel_z_dz_dz))^2 * 3.27e21`

Comment 4.6:

In Table 1, the Newton viscosity law is presented for all components of the viscous stress tensor, but the supplemental table S8 only shows one component of this tensor. Were the other components found and not presented in the supplemental material (the response to 4.15 says this table is a complete list of all unique formulas discovered)? Part of my confusion is that this is the only equation not presented as an energy with Deflex, and it remains unclear why this is the case.

Response to Comment 4.6:

Thank you for this comment. Indeed, the other components could be found but not presented in SI. In particular, all the components are almost the same. This component is not presented as an energy function because it is not directly found, but derived from the discovered Navier-Stokes equation, which has been explained in our **Response to Comment 4.3 in the previous revision**.

To respond to this comment, we have made the following modifications in both the revised manuscript and SI.

-1) We have revised Subsection 2.6 of the manuscript to highlight the discovery of Newton viscosity law from the Navier-Stokes equation.

Revised Manuscript (Subsection 2.6):

> As listed in Table 1, Deflex detects the Navier-Stokes equation of fluid dynamics through small-scale observations, which cannot be discovered by other methods. In larger-scale random observations, Deflex extracts the velocity distribution of the fluid within the vortex flow. These formulas correspond to different manifestations of long-standing laws in physics. For complex distribution formulas like velocity distribution, most compared methods are infeasible (shown in Table 2), and only AI Feynman and Deflex manage to discover. Notably, from the discovered Navier-Stokes equation, we can further identify the term corresponding to Newton's viscosity law, which is also listed among our discoveries in Tables 1 and 2. Meanwhile, SINDy and Operon can recover a simplified form of 2D Navier–Stokes without pressure-field terms (i.e., ∇p), which leads to noticeably larger errors than Deflex, even though the simpler structure can make their discovery faster. Deflex is the only method that can recover the full 3D Navier–Stokes equation in the JHTDB setting.

-2) We have revised the Supplemental Table 8 in SI to show the complete results of our discovery, which are also shown as follows.

Revised SI (Supplemental Table 8):

Newton viscosity law	Deflex	`τ_xy = -1.037 * μ * (vel_x_dy + vel_y_dx)`<br>`τ_xz = -1.037 * μ * (vel_x_dz + vel_z_dx)`<br>`τ_yz = -1.037 * μ * (vel_y_dz + vel_z_dy)`
	Operon	`τ_xy = -1.063 * μ * (vel_x_dy + vel_y_dx)`<br>`τ_xz = -1.063 * μ * (vel_x_dz + vel_z_dx)`<br>`τ_yz = -1.063 * μ * (vel_y_dz + vel_z_dy)`
	SciMED	`τ_xy = -1.052 * μ * (vel_x_dy + vel_y_dx)`<br>`τ_xz = -1.052 * μ * (vel_x_dz + vel_z_dx)`<br>`τ_yz = -1.052 * μ * (vel_y_dz + vel_z_dy)`

Comment 4.7:

The ablation study was useful for understanding the significance of the pretraining, but I have a couple of questions here. How is recovery speed defined? You explain that with a pre-trained model, it takes 5,000 steps for over 80% recovery rate, and from scratch, it requires 20,000 steps. It is unclear from this statement which approach is faster because this does not account for the pretraining time. If you account for the pretraining time, is it still faster to use the pretrained model? And, to clarify, is it correct that the timing in Figure 3 accounts for every step (including the pretraining) in the Deflex process?

Response to Comment 4.7:

Many thanks for this comment. The recovery speed is defined by the time cost of post-training, inference of Deflexformer, and hierarchical symbolic regression.

For once application of Deflex, training from scratch is faster but results in lower recovery rate. The reason is that pre-training over a large amount of samples, drawn from the generated equations by Deflexpressor, would significantly increase the time cost than directly training from scratch. However, by pre-training over synthesized datasets, Deflex can incorporate the prior knowledge of correct formulas, which can also greatly improve the recovery rate than training from scratch. This means that, for once application of Deflex, if we account for the pretraining time, it is not faster to use the pretrained model.

However, as Deflex block only needs pretraining once but can be used for unlimited number of times, pretraining is not only time efficiency but also provides better discovery rate for different systems in the long run. In this case, even if we account for the pretraining time, it is faster to use the pretrained model for multiple systems.

Thus, to clarify, the calculated time in Figure 3 does not account for the pre-training time. The reason is that this part of time is highly related to the studied system. In particular, the pre-training of

Deflexformer block is not counted because it is independent of any specific complex system and requires only pre-training once in an offline mode and then can be used without any limit.

Unless otherwise specified, the Deflex block in our experiments was fully pre-trained with 10K sampled data, which ensures a minimized fitting error. This cost around 45.6 minutes without any speedup techniques, which however, can almost maximize recovery efficiency when Deflex is applied to any complex system.

To respond to this comment, we have clarified this in Subsection 2.2 of the revised manuscript, which is shown as follows.

Revised Manuscript (Subsection 2.2):

> erable error in NLL or RMSE. Note that the run-time of Deflex is measured from applying Deflex on the observation dataset, including post-training and inference of Deflexformer, and hierarchical symbolic regression for formula discovery. It excludes the time cost of the Deflexformer block pre-training, which was conducted offline over 10,000 sampled pairs and independent of any studied system. A discovery is consid-

Comment 4.8:

In your response, you said that if no global information exists, it is introduced as noise. Is this in the manuscript or the SI? I could not find it when searching, but I might have just missed it?

Response to Comment 4.8:

Many thanks for your comment and effort for reviewing. We are very sorry for not explicitly including this in our previous manuscript. As mentioned in our previous manuscript, the system features in the observation dataset may optionally include some global features characterizing the system from a macroscopic level (e.g., a system-wide temperature). However, these were not clearly defined throughout the manuscript. To respond to this comment, we have made the following modifications to clarify this in the revised manuscript, which are shown as follows.

-1) We have rewritten the problem formulation in Subsection 4.1 to highlight that the features may include both the element-level and global-level ones, and so does the corresponding observation dataset. Specifically, the observation data X consists of the element-wise state dataset S and the global context V, i.e., X=[S; V] where the global context V is optional.

Revised Manuscript (Section 4.1):

> ### 4.1 Problem formulation
>
> We consider describing a complex system by a set of measurable features $\mathbf{x}$, which normally include the element-level ones $\mathbf{s}$ and optionally the global-level ones $\mathbf{v}$, i.e., $\mathbf{x} = [\mathbf{s}; \mathbf{v}]$. Specifically, the element-level features $\mathbf{s}$ are state variables that describe the local, dynamic condition of individual components (or elements) within the system (e.g., a particle's position and velocity), which are time-varying. The global-level features $\mathbf{v}$ may characterize the system from a macroscopic level (e.g., a system-wide temperature) and is considered independent of both element amount and time. In particular, Suppose we can obtain an observation dataset $\mathbf{X} = [\mathbf{S}; \mathbf{V}]$ from the system, where $\mathbf{S} \in \mathbb{R}^{NTD_s}$ denotes the D_s-dimensional state $\mathbf{s}$ of N elements within a

time period of T, and the optional $\mathbf{V} \in \mathbb{R}^{D_v}$ represents the D_v dimensional global information $\mathbf{v}$. Note that, except for the category of global or elementary features, we have no prior knowledge about the relationships among the individual features.

-2) We have revised Subsubsection 4.4.1 of the manuscript to clarify its input data. Specifically, the observation data X is fed into the input layer of Deflexformer. When no global feature v is included in x and no global context is given in X, then we set a dummy feature and draw some random noise to manually generate a global context dataset V.

Revised Manuscript (Subsubsection 4.4.1):

$D_{model} = 64$ is the model dimension. For some observations of a complex system, $\mathbf{X}$ may only include the element-level state information $\mathbf{S}$ without the global information $\mathbf{v}$. In such a case, we set a dummy global feature $\mathbf{v}$ and randomly generate a dummy global context dataset $\mathbf{V}$ by drawing some random noise over $\mathbf{v}$ (see SI for details).

Note that, designed for a much more complicated SR problem over observation data of complex systems with multiple scales, Deflexformer can also be directly used for classical SR tasks (e.g., applied to the AI Feynman dataset in Section 2.7), which correspond to a single scale without considering element-level dynamics. Here, the SR dataset is input as the global context, and the element-level representation is left empty at the input layer.

-3) We have revised Subsection 8.1 of SI to add details about how Deflex processes the input data.

Revised SI (Subsection 8.1):

8.1 Data representation and Deflexformer architecture

We denote the observation data as $\mathbf{X} = \{x_i\}_{i=1}^{N}$, where each x_i comprises n elements observed over t time frames. In our formulation, each observation may include both element-level states S and an optional global context V characterizing the system at a macroscopic level (e.g., a system-wide temperature), i.e., $x = [S; V]$ with V possibly absent; when V is unavailable, we introduce global slots as fixed Gaussian padding tokens (sampled once and reused) rather than resampling noise at every inference. Importantly, the elementwise and temporal axes are not mandatory: when a task provides only global descriptors (or does not benefit from element/time factorization), the model can operate with an empty elementwise tensor and treat the input entirely as global tokens. The Deflexformer network approximates an energy function $\mathcal{E}_\Theta$:

Algorithm 5 Deflexformer Forward Sweep

Input: Observation x, block count k, element width d, local width d_ℓ, scale count S, range R
Output: Intermediate sequence $\mathcal{R} = (\mathcal{R}_0, \dots, \mathcal{R}_k)$, encoded scalar e_x

```
                                                    ▷ Implements F_fwd(x; k, d, d_ℓ, S, R)
v_0 ← EMBED(x, d, S, R)                             ▷ Fourier multiscale embedding of raw input
(P_0, G_0) ← STATEINIT(v_0, d_ℓ)                    ▷ Split into element/global tensors (with P_0 possibly empty)
R_0 ← (P_0, G_0)
for i = 0 → k − 1 do
    (P_{i+1}, G_{i+1}) ← F_block^(i)(P_i, G_i)      ▷ Apply Deflexformer block i
    R_{i+1} ← (P_{i+1}, G_{i+1})
end for
g ← F_proj(G_k, 2S)                                 ▷ Select leading global channels
e_x ← EMBEDINV(g, 2S, S, R, 1)                      ▷ Invert embedding to scalar energy
return R, e_x
```

linear projections. Consistent with Algorithm 2, elementwise embeddings use zero padding for unused channels, while the global context is initialised as follows: when auxiliary global descriptors are available, we embed them using the same Fourier multiscale map and project them to initialise G_0; otherwise, we treat the global context as Gaussian padding tokens and initialise G_0 once as $G_0 \sim \mathcal{N}(0, I)$, keeping this padding fixed across forward passes rather than resampling it at every inference. In purely symbolic regression settings without an explicit notion of entities or time (e.g., SR over common tabular datasets), we place all input features into the global descriptor set and let the element-wise tensor be empty (so the forward sweep reduces to transformations over G). For static observations without temporal sequences, we treat inputs as a single frame ($t = 1$), and the temporal-mixing component becomes a degenerate case. Successive applications of k Deflexformer blocks $\mathcal{E}^0, \dots, \mathcal{E}^{k-1}$ yield intermediate repre-

Comment 4.9:

Below Eq. 8 you write "Malkov" instead of "Markov"

Response to Comment 4.9:

Many thanks for pointing out this typo. We have carefully proofread the whole manuscript to eliminate the typos and grammar errors in this revision.

Revised Manuscript (Subsubsection 4.4.3):

> and adopt the Markov Chain Monte Carlo (MCMC) method with Langevin sampling [53, 54]. While computing the partition function $Z_{\boldsymbol{\theta}}$ itself is intractable, its gradient $\nabla_{\boldsymbol{\theta}} \log Z_{\boldsymbol{\theta}}$ can be efficiently estimated via MCMC sampling, which is sufficient for gradient-based optimization (see SI for detailed methodology and parameter setups). Crucially, this approach discovers probability distributions directly from raw data points via likelihood maximization, rather than fitting predefined probability density functions (PDFs) to pre-computed statistics.

Comment 4.10:

I do not see where ND is defined in 4d,e,f. Is this supposed to be VD?

Response to Comment 4.10:

Thanks for pointing out this issue. ND stands for Newton dynamics, which specifically denotes momentum conservation in this work. To respond, we have replaced it with "MC" defined above, and revised the legend in Fig. 4, which is shown as follows.

Revised Manuscript (Fig. 4):

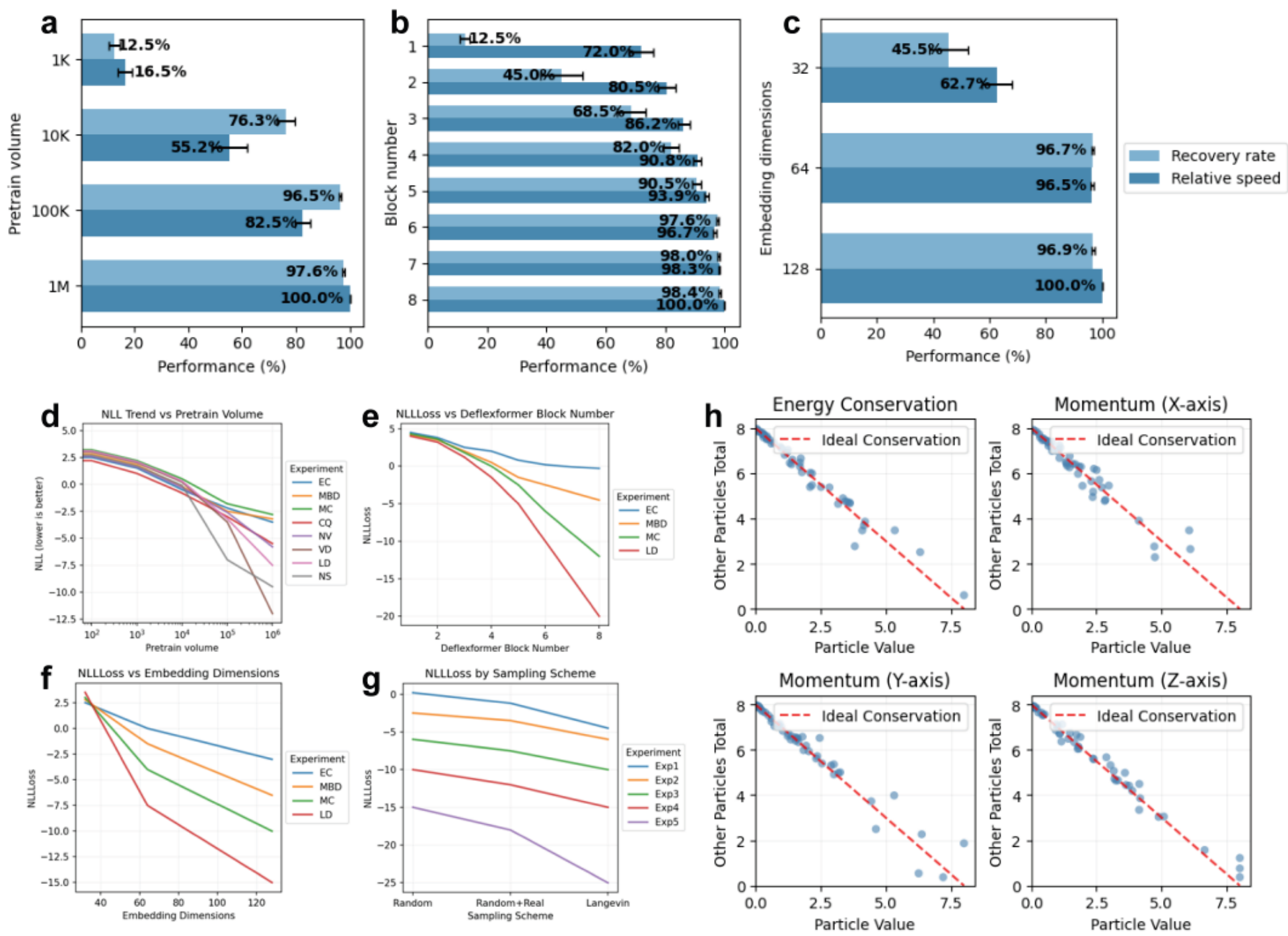

Fig. 4 Ablation studies and component analysis of Deflex. a, **e**, Impact of pre-training volume on formula discovery performance (recovery rate and relative speed) and NLL trend. Larger pre-training volumes significantly accelerate discovery and improve accuracy. **b**, **f**, Performance metrics and NLL loss with varying Deflexformer block numbers. Increasing depth improves representation power. **c**, **g**, Effect of embedding dimensions on performance. **d**, Ablation on derivative (gradient) features, comparing the default feature configuration against a variant where temporal-derivative and spatial-gradient channels are removed from the input. **h**, NLL loss comparison across different sampling schemes (Random, Random+Real, Langevin). Langevin sampling yields the best fitness. **i**, Verification of conservation laws (Energy and Momentum) learned by the Deflexformer, showing close alignment between sampled states (black dots) and ideal laws (red lines).

We have also double checked all the figures and tables to ensure correct presentation.

Overall Thoughts

This work presents a method for discovering equations from data through a combination of deep learning and symbolic regression. The authors refer to this approach as Deflex. This method consists of training deflexformer blocks (modified transformers) to find function approximations for energy models, and then using deflexpressor with symbolic regression to fit the deflexformer latent functions. An advantage of this approach is that it allows for the symbolic regression to solve a series of subproblems for each of the deflexformer blocks, as opposed to solving the entire symbolic regression straight from data. The method is validated on many example problems, including: Argon particles, pollen particles, human mobility, bird migration, water particles, flow around a cylinder, and on a benchmark database.

Overall, this paper presents a new method for equation discovery that yields impressive results. The authors do an excellent job of validating the approach across a variety of problems and of comparing the approach to a variety of other methods. The main shortcomings are in the presentation of Deflex and in the justifications for why it works. In the following section, I list the major and minor comments I had while reading the paper.

Major Comments

1. I have a few concerns and questions about the fluid dynamics problem:

   (a) Citation [10] shows that SINDy finds the Navier-Stokes Eq. (NSE) with and without noise. Why are you unable to find the correct equation using SINDy in this paper?

   (b) SINDy is typically designed as a regression problem for the output du/dt. How are you setting up the problem such that SINDy can be used to find the continuity equation and the Newtonian constitutive equation?

   (c) How is the Newtonian constitutive equation found using these methods? With the norm of continuity and NSE, the value should go to 0, which is what is shown. But how can the shear stress be found like this? Does deflex just find different groupings of terms in the NSE? For example, why wouldn't the equation for the total derivative also come out of this analysis ($D\mathbf{u}/Dt = \partial\mathbf{u}/\partial t + \mathbf{u} \cdot \nabla\mathbf{u}$)?

   (d) How do the large-scale observations work for this problem? I do not understand what is meant by: "In larger-scale random observations, Deflex extracts the velocity distribution of the fluid within the vortex flow." Also, what is the δ in the velocity distribution equation?

   (e) The details of what is happening in the simulation are not very important here. Only the fact that it is a numerical solution to the NSE. However, the simulation appears not to be very high-quality.

The boundary conditions have a major influence on the flow due to the small domain. This means the results will differ from typical cylinder flow results. It would be useful to report the boundary conditions and the initial condition. Is this a startup flow? Lastly, I do find it odd to use simple periodic shedding when the example in Fig. 1b is turbulence, especially when trying to highlight the importance of multiscale flows (which is only the case for a turbulent flow). A much more interesting case study would have been to consider data from the Johns Hopkins turbulence database.

2. I have many questions about the details of the architecture and training of Deflex:

    (a) It is unclear what makes up the element and embedding dimensions of P. For example, in particle motion, the time frame, the mass, position, and velocity are embedded into 64 dimensions. How is this done? And, are the elements here supposed to represent the dimension of the batch size, or is there a separate batch dimension not included?

    (b) Are all of the equations for a single system found in one pass of training the deflexformer through multiple symbolic regression passes? Or, is everything retrained for each equation?

    (c) It is challenging to understand the choices in the deflexpressor. Was there extensive manual tuning on how to select the types of mutations? Is this how you chose to add the new styles of mutation? Also, how does the autoregressive neural network work in this framework? How is this NN train?

    (d) The training process for the deflexformer is unclear. Is it also using the NLL loss? If so, how do you get f out of the deflexformer? Figure 3d makes it seem like each deflexformer block has a scalar output. However, I thought that there would be some layer after the final deflexformer block that would generate f. Or is there another loss used directly with P_K, G_K for training the deflexformer?

    (e) Do you pretrain a single deflexformer block or many different ones?

    (f) How close are the deflexformer blocks to the original equations they are pretrained on? This is an important question because it determines if the deflexformer blocks are actually beneficial. It seems like they are unnecessary if they are not learning new functions.

    (g) In Fig. 2, it is not clear where the self-attention network (2b) lies in (2a). Furthermore, it is hard to decipher what is happening in the grey boxes (e.g., element-wise self attention) without showing more details of the equations.

    (h) Why is the global context needed, and what is the purpose of adding random noise for this context?

(i) In 4.2, f is shown as taking one argument $\mathbf{x}$, which makes sense to me. How in 4.5 is f able to take two arguments $\mathbf{h}_i$ and $\mathbf{h}_j$? The paragraph discussing how to find the Pareto-optimal set with the Reduce process is difficult to follow. It would be helpful to add more or rephrase this section.

3. Are the equations presented in Table 1 the only equations discovered from Deflex? Or were additional equations discovered that were not presented?

Minor Comments

1. On pg 3, DNN is used before it is defined.
2. Figure 1 and the comments like "...mimicking the process scientists use to break down intricate systems to make the approach efficient for highly complex formulas" seem very out of place. These connections are loose at best.
3. On pg 6, there is a typo "Here,Since".
4. It would be useful to show examples of some of the probabilities used to compute the EMD in Fig. 4c. Adding further clarification here would also be helpful. I do not understand how you are getting the different scales here, especially for cases like the NS.
5. In Eq. 3 are g and f flipped in the inequality for L?
6. Pg. 15 has a typo where you write "global representation P_{k-1}"
7. The abbreviation "NLL" is used before it is defined.
